# Deriving a Mutation Index of Carcinogenicity Using Protein Structure and Protein Interfaces

**DOI:** 10.1371/journal.pone.0084598

**Published:** 2014-01-15

**Authors:** Octavio Espinosa, Konstantinos Mitsopoulos, Jarle Hakas, Frances Pearl, Marketa Zvelebil

**Affiliations:** 1 Breakthrough Breast Cancer Research Centre, Institute of Cancer Research, London, United Kingdom; 2 UK Cancer Therapeutics Unit, The Institute of Cancer Research, London, United Kingdom; 3 Translational Drug Discovery Group, School of Life Sciences, University of Sussex, Brighton, United Kingdom; University of Rome, Italy

## Abstract

With the advent of Next Generation Sequencing the identification of mutations in the genomes of healthy and diseased tissues has become commonplace. While much progress has been made to elucidate the aetiology of disease processes in cancer, the contributions to disease that many individual mutations make remain to be characterised and their downstream consequences on cancer phenotypes remain to be understood. Missense mutations commonly occur in cancers and their consequences remain challenging to predict. However, this knowledge is becoming more vital, for both assessing disease progression and for stratifying drug treatment regimes. Coupled with structural data, comprehensive genomic databases of mutations such as the 1000 Genomes project and COSMIC give an opportunity to investigate general principles of how cancer mutations disrupt proteins and their interactions at the molecular and network level. We describe a comprehensive comparison of cancer and neutral missense mutations; by combining features derived from structural and interface properties we have developed a carcinogenicity predictor, InCa (Index of Carcinogenicity). Upon comparison with other methods, we observe that InCa can predict mutations that might not be detected by other methods. We also discuss general limitations shared by all predictors that attempt to predict driver mutations and discuss how this could impact high-throughput predictions. A web interface to a server implementation is publicly available at http://inca.icr.ac.uk/.

## Introduction

Many cancers arise as a result of the acquisition of a series of fixed DNA sequence abnormalities, termed mutations, which ultimately confer a growth advantage upon the cells in which they have occurred [1], [2], [3], [4]. These mutations can have several impacts on the gene in or near which they reside. Mutations that contribute to disease initiation or progression, often by altering the protein product directly, are termed “driver” mutations, whereas those mutations that are a result of the inherent genetic instability of the cancer, confer no selective advantage to the cell and do not contribute to disease progression are termed “passenger” mutations.

With the advent of Next Generation Sequencing (NGS) the identification of mutations in the genomes of healthy and diseased tissues has become commonplace providing a new avenue to discover potential genotypes underlying the molecular causes of cancers [5]. Key to this endeavor is the ability to determine which mutations are contributing to the disease process. The most common mutational event in cancer that changes the protein product is a missense substitution, where usually a single base substitution changes the protein product by a single amino acid. However the consequence of these mutations still remains challenging to predict.

There is a large body of work documenting the consequences of inherited missense mutations, as they comprise a large part of the repertoire of human disease variants as evidenced in the OMIM [6] and HGMD databases [7]. Studies show that disease-associated mutations commonly impact protein folding, protein stability, and protein-protein interactions (PPIs) [8] thus altering protein function. What is key to determining the molecular and hence biological impact of a mutation, is its location within the protein structure and the molecular function of residues affected [9]. Many studies have shown that both the evolutionary conservation of the mutated residue, the severity of difference in physiochemical properties of the substitution and the structural attributes of the residues involved, are all indicative of the disruption of the protein, with more “diverse” substitutions resulting in disease [10]. This also includes inherited missense mutations within cancer predisposition genes such as those in BRCA1 [11], [12].

More recently, studies have compared the conservation of somatically acquired cancer mutations with neutral missense mutations suggesting [13] [14] that both the evolutionary structural and functional conservation of the mutated residue and its local environment, combined with the severity of the substitution discriminate between cancer associated and neutral mutations.

Sequence and protein structure have often been used to predict whether non-synonymous single nucleotide polymorphisms (nsSNPs) could be disease causing, as well as assisting in ranking or prioritising candidates for experimental validation. Sequence conservation has been used to predict which mutations would not be tolerated within a protein, and are often used as a proxy to identify disease-causing mutations [15], [16], [17], [18], [19], [20], [21], [22]. Similarly, protein structure has been used for estimating how disruptive a missense mutation maybe [18], [19], [23], [24], [25], [26], [27], [28]. Recently Reva et al. [29] successfully used comprehensive multiple sequence alignment of proteins to create a functional impact (FI) metric to score amino acid substitutions. Other methods include filter-based algorithms to prioritize pathogenic mutations [30].

While several structure-based predictors exist for estimating general pathogenic effects of missense mutations these are not specifically oriented towards cancer mutations and do not benefit from the comprehensive structural detail of protein interfaces. From a structural perspective the structural impact of a driver mutation is dependent on whether the protein is an oncogene or tumour suppressor. The structural consequences of mutations within tumour suppressors, where protein function is ablated, are often similar to those in inherited diseases, in that they impact on protein stability and folding. Mutations can also disrupt active sites or ligand binding sites, whether directly by occurring in or near the site or indirectly by destabilising the site's structure, will also be severely detrimental to the protein's function. In contrast mutations in oncogenes, where the protein is activated, are found in loops and unstructured regions of proteins and not in the protein core [31], [32].

When considering the impact of a mutation, protein-protein interfaces are also important regions of the protein to consider since they are responsible for mediating protein interactions within the cell. Protein interfaces have discernible characteristics such as complementarity in shape and electrostatic charge and the presence of hydrophobic patches [33], [34]. Hydrophilic residues are more frequent in interfaces facilitating transient interactions, whereas interfaces in more permanent subunit associations in complexes often have hydrophobic patches.

Recent analyses on protein structures in the Protein Data Bank Europe (PDBe) [35], [36] have revealed a comprehensive set of protein interfaces which were deposited in the Protein Interfaces, Surfaces and Assemblies (PISA) database [37]. Coupled with genome-wide NGS mutation data, this gives the opportunity of exploring how mutations manifest structural defects in proteins and therefore provide useful insights into how they may cause cancer genome phenotypes. Examples of interface mutations that disrupt protein-protein interactions have been previously documented [38] and the value of molecular-level annotation of proteins with respect to interfaces has recently been demonstrated [39] by predicting the effect of mutations using interface proximity and offering explanations for pleiotropy and locus heterogeneity in terms of mutation location with respect to interfaces.

With the increasing amounts of sequence and mutation data from NGS experiments, there is an accompanying need to develop better ranking and prediction tools to assess and characterize cancer mutations computationally. Here, we describe a new predictor, InCa (Index of Carcinogenicity), based on criteria derived from a large scale analysis of cancer driver mutations from the COSMIC database and the HapMap mutations in the 1000 Genomes (1k) project. We show that by focusing on structure and interface information, our parameters can be used to obtain similar or better prediction than previous methods that predict severity of mutations in cancer data sets based on structure and sequence conservation, such as those described in [40] and [22] and detect mutations that are not detected by some of these methods. When we compare predictions of InCa and CHASM [22], another cancer-specific missense driver mutation predictor, we also find that relevant mutations are detected by both methods that are not detected by the other. We discuss general caveats affected by all current prediction methods and how they affect predictions of driver mutations in the context of cancer biology.

## Methods

### Protein structure data and protein interface data

Protein interfaces were obtained from PISA and assembled into an in-house database which was further expanded with data from the Structure Integration with Function, Taxonomy and Sequence (SIFTS) initiative database [35] allowing translation of PDB to UniProt coordinates. PISA interfaces with complexation significance scores (CSSs) of zero and an interface area of <400 Å were considered as ambiguous and excluded from our analysis. Using the PISA quaternary structure definitions we computed intra- and inter-molecular minimum atomic contacts for residues, as well as the centre of gravity for each interface. Relative accessible and buried surface areas (ASA and BSA) were calculated from the PISA absolute ASA and BSA values as the fraction of the accessible area of the respective amino acid in the tripeptide Gly-X-Gly [41]. Secondary structure DSSP [42] assignments were obtained from SIFTS and simplified to three states: helix, H, beta sheet/strand, E, and coil, C. Non-human PDB interfaces were filtered out using PDB chain to taxonomy mappings (ftp://ftp.ebi.ac.uk/pub/databases/msd/sifts/text/pdb_chain_taxonomy.lst). To obtain background distributions of amino acid frequencies in our data set, we used a non-redundant human list of proteins from NCBI (ftp://ftp.ncbi.nih.gov/mmdb/nrtable/nrpdb.060111) with p-value for similarity cutoff of 10e-80. The UniProt database [43] was parsed and the annotations for post-translational modifications and disulphide bonds were added to the residue data. A further comprehensive set of post-translational modifications were obtained from Phosphosite [44]. We included acetylation, methylation, O-GlcNAc modification, phosphorylation, sumoylation and ubiquitination. Only the sites obtained via high throughput mass spectrometry were taken to reduce any investigation bias.

### Neutral mutations, mutations in cancer genomes and driver mutations

Non-synonymous SNPs from non-diseased individuals were downloaded from the 1000 Genomes (1k) Project [45]. Those from somatic cancer genomes were downloaded from COSMIC version 64 [46]. The mutations in both studies were annotated to transcripts in the Ensembl database [47], [48] by alignment to the reference human genome. The mutations were sorted with respect to protein topological area by their interface area and CSS (if the residue is in an interface), relative accessible surface area (relative ASA) and buried surface area (relative BSA) (Table S1).

Driver mutations were taken from [22], which is a mutation list from COSMIC in putative oncogenes and tumour suppressor genes identified using a variant of the 20/20 rule [49]. We obtained the most recent list from the author's url (http://wiki.chasmsoftware.org/index.php/CHASM_DL) downloaded on 21.12.2012.

Out full data frame of mutations is available in Dataset S1.

### Biases in frequency of mutation classes and physicochemical dissimilarity of residues

Overrepresentation of mutations classes was calculated by constructing contingency tables and applying Fisher's exact tests correcting for multiple testing using false-discovery rate. Details are provided in supplementary methods in Text S1. Details of physicochemical dissimilarity for mutations are given in supplementary methods in Text S1 and Table S2.

## Results

### 1000 Genomes and driver mutation comparison overview

Our compiled list of neutral and cancer driver mutations mapping to proteins with structure comprised 2412 unique single nucleotide polymorphisms from the 1000 Genomes (1k) project and 3808 unique mutations from cancer drivers. These were mapped to 1207 and 57 unique proteins (UniProt IDs) respectively. 3 mutations and 23 proteins overlapped in the two data sets (Figure S1 A–B).

The most frequently mutated proteins with driver mutations with were p53, PTEN, EGFR, CDKN2A and PIK3CA, all of which are highly studied oncogenes or tumour suppressors. Conversely, among the proteins most frequently mutated with neutral mutations were immune system proteins like HLA class II histocompatibility antigen, HLA-DPB1 (P04440). The amount of mutations for the top genes with the most neutral mutations and most driver mutations are shown in Table S3. The average for drivers and neutral proteins were 2.0 and 54.2 respectively, and the mode 1 and 5 respectively. The distribution of mutations per protein had a longer tail for driver proteins (Figure S2).

### Cancer driver mutations are less conservative than neutral mutations

To compare the neutral and driver mutations, it was necessary to ensure that there was no underlying bias in the observed amino acid frequencies in 1k and COSMIC. The normalised frequencies were very similar in the two datasets and were also highly correlated with those observed for both UniProt and the PDB as a whole (Figure S3). There were two exceptions, with both Ser and Pro residues being slightly under-represented in both the structurally constrained datasets.

We calculated several measures to compare of the nature and the conservation of the mutations in driver and neutral datasets. Cancer driver mutations exhibited significantly higher physicochemical differences between the wild-type residue and the mutant, than neutral mutations, suggesting that in general they exhibit less conservative substitutions (Figure S4). This observation was supported by the lower BLOSUM substitution scores, and the lower Dayhoff substitution scores demonstrated by the driver mutations, indicating that these mutations were less conservative than neutral mutations.

Driver mutations also exhibited higher functional impact (FI) scores [29], supporting the hypothesis that driver mutations are both less conservative (Figure S4) and occur in both functionally and structurally conserved regions of the protein. However, in a number of cases the FI score could not be calculated because the mutation fell outside a region of the requisite multiple sequence alignment. There were also several 1k mutations where the FI score was significantly high. Further analyses are required to determine whether these mutations will disrupt the protein function leading to a pathogenic impact, or whether these are false positive results. Either way, the development of alternative methods to predict the carcinogenicty of mutations is important.

To ensure these distribution differences were not due to a biased artefact in the data for driver mutations having more extensive interface or interaction partner annotation, we obtained the corresponding distributions for the reduced set of 23 proteins in the intersection of proteins with driver mutations and proteins with neutral mutations. Using this set reduced the number of mutations from 5500 to 1677, a 30% reduction. We observed the same differences in distribution, with only two cases being below statistical significance at the 0.05 level, namely FI scores for mutations in buried residues and mean distance to interface for mutations in interface areas (Figure S5). However, because this reduced set greatly reduces the number of mutations available for analysis, we suspect this loss of significance is likely due to insufficient data.

### Driver biases in topology, secondary structure and amino acid composition

As we wanted to incorporate structural features in our prediction algorithm we first investigated which structural parameters to include. Several studies have suggested that cancer-causing mutations preferentially occur in particular locations within a protein structure, for instance temperature-sensitive (TS) mutations often occur in buried regions of the protein. We investigated these biases in topology of mutations as a whole and investigated whether they occurred at an interface, on the protein surface or were buried and we also recorded their secondary structure (helix (H), sheet (E), or coil (C)).

In general, mutated residues occurred less frequently in buried positions and more frequently in surface accessible positions (Figure S6 A,C). This tendency was even greater for the neutral mutations alone. Driver mutations occurred slightly more frequently in interfaces. Interestingly, driver mutations occurred more often in coils and beta sheets whereas neutral mutations occurred less frequently in α-helices (Figure S6 B,D). In both datasets arginine was the most frequently mutated residue despite its highly redundant codon usage. We further calculated the normalised frequency and observed/expected ratio of the corresponding mutation classes for the reduced set of 23 proteins containing both neutral and driver mutations. We observed the same differences, with driver mutations occurring more frequently in interfaces (Figure S7).

To further investigate the differences in the distribution of driver and neutral mutations, the data was partitioned by secondary structure, topology and mutated wild type residue. Several biases were discernible in the type of amino acid mutated, particularly when the data was partitioned by secondary structure type (Figure S8): tryptophan residues were more often mutated in drivers, as well as buried cysteine residues in beta sheets and coils, buried hydrophilic residues (aspartate, glutamate, histidine and phenylalanine) and interface glycine residues.

Several types of amino acid substitutions were enriched in drivers (Figure S9, S10). Table 1 summarises the top 10 significantly enriched mutation types in each category. Both glycine to valine and glutamate to glycine substitutions were enriched in interface areas and leucine to proline mutations in interface helices. These mutations were all predicted to be deleterious from their physicochemical parameters. In particular proline is known to be a “helix breaker” [50], [51] and such mutations to a proline residue within a helix are likely to have a considerable structural impact. Some amino acid substitutions were never observed in either data sets (Figure S9). We found that amino acid substitutions requiring two or more nucleotide substitutions (using values from the genetic code matrix) were never observed in neutral mutations, whereas in drivers, several cases of different amino acid substitutions requiring two or more nucleotide substitutions were observed. However, in general, amino acid substitutions requiring only 1 nucleotide substitution were more prominent and the non-observed amino acid substitutions corresponded mostly to those requiring 2–3 nucleotide substitutions.

**Table 1:** Summary of top mutation types in each category enriched in drivers.

secondary structure	buried	interface	surface
coil	T→I		
sheet	C→R,L→R		
helix	L→R		
any	C,H,L,W,Y,L→P,L→R,V→D	G,L,G→E,L→P,L→R,G→V,R→P	Y,L→R,G→V

Conversely, alanine isoleucine and valine residues were mutated more frequently in the neutral dataset, especially on the protein surface (Figure S8), suggesting that when these residues are located on the surface of the protein they can tolerate mutations with little detrimental effects. Buried valine to isoleucine mutations were also enriched in the neutral dataset probably facilitated by their similarity in physicochemical properties (Figure S9B).

Driver mutations were significantly enriched in interface electrostatic bonds and post-translational modifications (Table 2). The enrichment of cysteine mutations in drivers prompted us to examine whether they could disrupt disulphide bond formation. In the majority of cases, mutated cysteines did not participate in disulphide bond formation and when they did, there was no statistically significant enrichment in driver, indicating that the mutation defects cannot be attributed to their loss. Interestingly the immediate vicinity (5 Å radius) of mutated buried cysteine drivers was highly enriched in cysteine residues (Figure S11), suggesting that the change in physicochemical properties may itself contribute to the mutation severity, or that the proteins in our dataset do not for disulphide bonds. This would be the case if many proteins in our data set are located to the cytoplasm, as opposed to localised to organelles and excretion, where disulphide bond formation occurs following their synthesis in the endoplasmic reticulum.

**Table 2:** Contingency tables for post-translational modifications, disulphide bonds and electrostatic (h-bonds and salt bridges) interface bonds in mutated residues.

	NO PTM	PTM	NO SS	SS	NO interface bond	interface bond
neutral	2387	25	2409	3	1987	425
driver	3019	69	3082	6	2077	1011
p	0.0007117		0.7398		<2.2e-16	

P-values are calculated with a Fisher's test with a two-sided alternative hypothesis.

### Driver mutations are located near protein interfaces

Driver mutations occurred closer to interface binding sites than neutral mutations (Figure S3) suggesting that mutations that interface disruption may be a factor in cancer pathogenicity. To investigate if the position of the mutation within the interface was important we divided mutated residues into groups depending on their relative accessible surface area (ASA) both in the monomeric- unbound state, and their corresponding accessibility when contributing to a multimer. We analysed these accessibilities and compared their frequency in drivers and 1k datasets (Table 3). Mutations occurring in interfaces in multimers that were in partially accessible residues in the monomer, were enriched in the driver dataset, suggesting that these were the most deleterious residue positions when combined in an interface. We calculated the same ratios for the intersection set of 23 proteins in the intersection. We observed the same pattern with partially accessible or buried residues in interfaces being enriched in driver mutations.

**Table 3:** Area propensities of mutations by accessibility.

area and accessibility	normalised frequency of driver/neutral in full set	normalised frequency of driver/neutral in intersection set
interface, <10% accesssible	3.63	11.12
interface, 10–30% accesssible	2.52	4.49
interface, >30% accesssible	1.26	1.52
buried, <10% accesssible	1.79	1.47
surface, 10–30% accesssible	1.55	2.38
surface, >30% accesssible	0.87	0.90

For each area, the percentages denote the relative ASA. Ratios denote the driver/neutral fraction. Values for the set of 23 proteins which contain both neutral and driver mutations is shown on the right.

We further analysed the driver and neutral mutations by also taking into account the residue hydropathy changes between wild type and mutant (Table 4).Hydropathy transitions (hydrophilic to hydrophobic and vice versa) were enriched in drivers, with the enrichment becoming stronger for residues of lower accessibility. The only notable exception was the hydrophobic to hydrophilic interface mutations, which showed slight enrichment in neutral mutations. It is possible that such mutations may be better tolerated in adjoining exposed hydrophilic interface patches.

**Table 4:** Ratios of COSMIC/1k of normalised hydrophobicity propensities by accessibility.

	PhiPhi	PhiPho	PhoPhi	PhoPho
interface, <10% accesssible	5.47	6.48	4.73	3.32
interface, 10–30% accesssible	3.05	4.23	4.19	2.28
interface, >30% accesssible	1.28	2.13	2.20	1.50
buried	3.13	3.25	3.10	1.44
surface, 10–30% accesssible	1.93	2.34	2.46	1.51
surface, >30% accesssible	0.90	1.39	1.45	1.11

“Phi” denotes hydrophilicity and “Pho” denotes hydrophobicity.

Together these data indicate that the disruptions that cause the cancer phenotype in interface areas are likely to occur from mutations that are buried or partially accessible in the monomeric unit. This effect is exacerbated when the residue hydropathy is altered, suggesting that partially exposed interface residue mutations in drivers may act primarily by distorting the protein interface shape. This effect may be more deleterious than the loss of hydrophobic contacts or electrostatic interactions potentially imposed by mutations found in highly accessible interface residues.

### Driver mutations disrupt electrostatic interactions across interfaces

**Figure 1:**
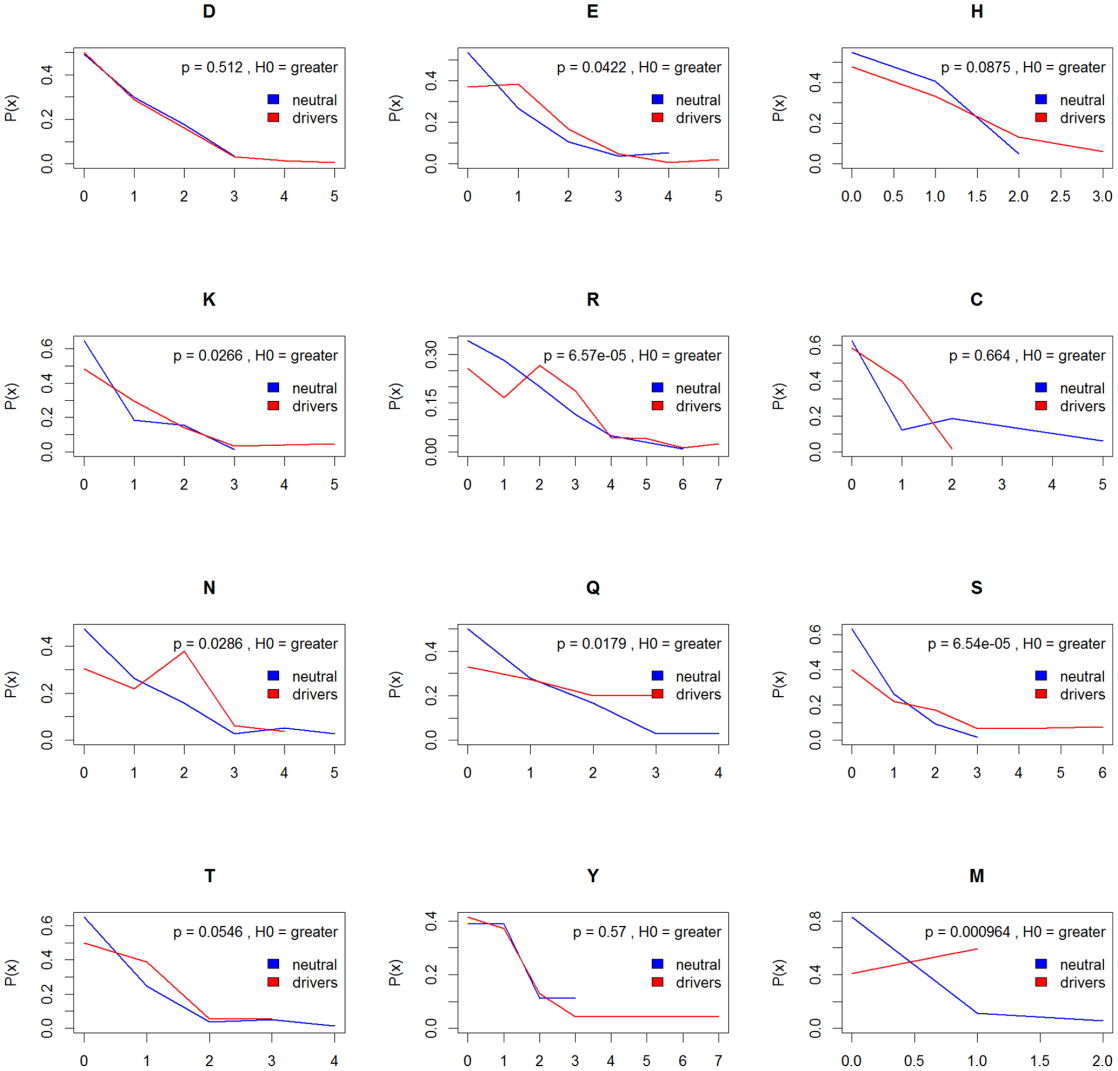
Hydrogen bond enrichment in interface residues for charged and polar residues in driver mutations. Densities (denoted P(x)) are shown for mutations of each amino acid in both sets with their associated p-values comparing 1k and drivers with a two-sample Wilcoxon test using a one-sided alternative hypothesis.

Formation of hydrogen bonds and salt bridges across the opposing sites of the interface plays a pivotal role in interface stabilisation [52]. To investigate whether mutations in drivers more often occurred in interface residues critical for electrostatic interactions, we calculated the maximum and mean hydrogen and salt-bridge bonds for each mutated wild type residue side chain. Compared to drivers, neutral mutations occurred in interface residues enriched for non-hydrogen bonded amino acids and in the majority of cases, the proportion of residues forming one or more hydrogen bonds, was significantly lower (Figure 1).

Driver mutations often occurred in amino acids that contribute a higher number of hydrogen bonds across the interface. To a large extent, these patterns were observed for salt bridges too but differences were statistically significant for glutamic acid and histidine only (Figure S12).

### Compositional differences between the drivers and neutral mutational microenvironments

A previous study [9] has shown that structural disruption by a mutation of the local environment correlates with the pathogenicity of a mutation. These include whether a mutation cause a steric clash, introduces a cavity in the protein, and estimate the local change in stability of a protein. Studies have also indicated that these metrics are dependent on both conformation and resolution of the protein structure, so we developed a “fuzzy” packing metric to describe the mutated residue's microenvirionment, which consisted of the normalised frequency of each amino acid residue in the vicinity of the mutated residue within a 5 Å radius. This metric was calculated on both the isolated monomer, and the PISA derived multimers so that we could capture information on both inherent and the interfacial microenvironments. Where more than structure was available, data from all the available structures were combined.

Although overall, neither drivers nor neutral proteins and their interfaces exhibited global compositional differences, for several of the 20 amino acids there were statistically significant compositional differences between the driver and the neutral interior and interfacial microenvironments (Figure S13, as well as Dataset S2 for full listings). We observed instances of polar or charged residues being in the vicinity of mutated polar or charged residues significantly more often in drivers, suggesting that disruption of electrostatic interactions or electrostatic patches of proteins are important in contributing to the cancer phenotype. We further fine-tuned the interface compositional analysis by subgrouping substitutions by wild type and mutant amino acid. While the differences did not reach significance, we observed several substitutions that represented a loss of electrostatic interactions in the context of the neighbouring residues. In several instances, such as mutations of phenylalanine to serine substitutions (Figure S14A) it is possible that cation-pi interactions with arginine residues on the opposite side of the interface may be abolished. Such interactions have been previously shown to be important binding contributors in protein-protein interfaces [53]. Glycine to valine mutations (Figure S14B) may be deleterious because they not only increase the volume of the side chain, but also abolish the conformational flexibility that is unique to glycine, potentially introducing a more widely felt interface distortion around the mutated residue. Full listings of microenvironments for each substitution and area are provided in Dataset S3.

### Model for predicting carcinogenicity (drivers) and comparison with other predictors

Having established which structural parameters may contribute to a mutation's carcinogenicity, we created a model, Index of Carcinogenicity (InCa), using a random forest algorithm [54], to predict whether a mutation induces a cancer phenotype. The parameters used and their contributing significance are listed in Table S4.

**Figure 2:**
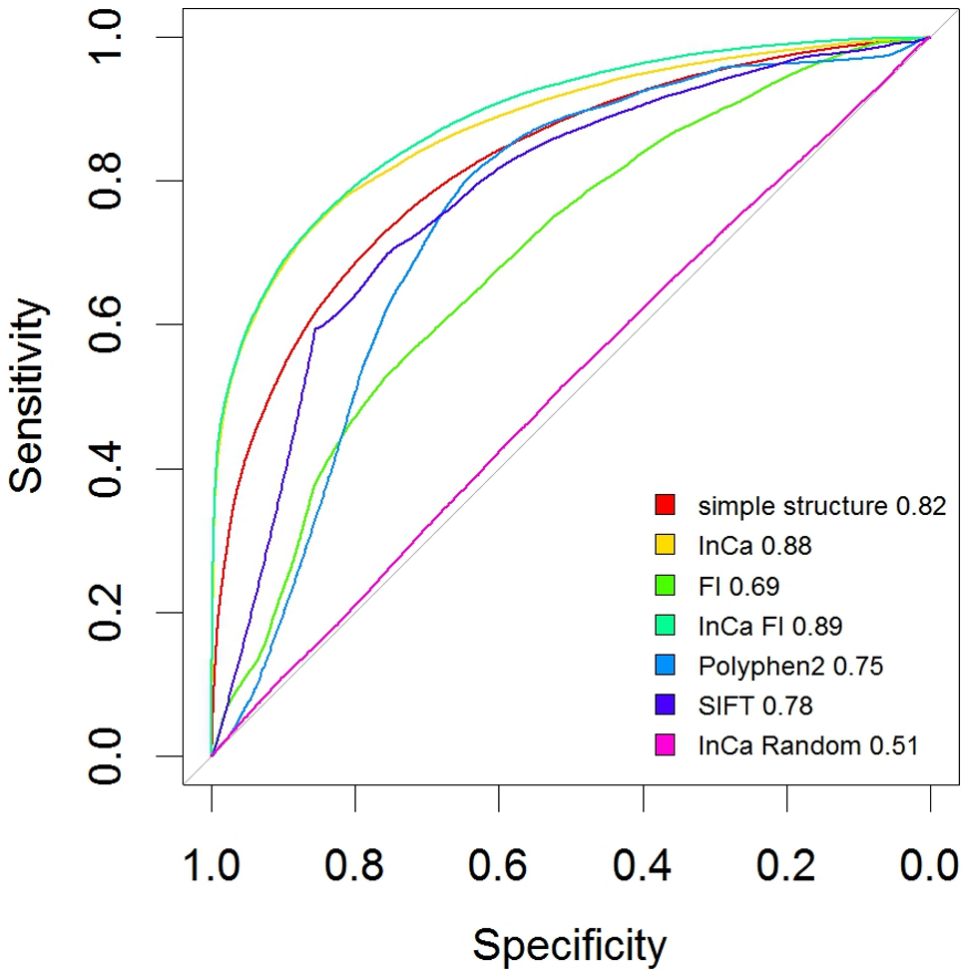
ROC curve for InCa and other mutation predictors. Shown are the standard metrics averaged using 10% randomly withheld annotations and 100 repetitions at each point. “simple structure” denotes a model without using frequency profiles of neighbouring residues. A model denoted “InCa FI” is a combined model using FI as a parameter. AUC values are shown next to the names.

We performed a 5 fold cross-validation with 100 iterations, where 20% of mutations were randomly withheld at each iteration and used the remainder as a training to train the model. The model gave an area under the curve (AUC) of 0.88. The optimal cut-off (which maximizes the distance to the identity (diagonal) line) was 0.54 and at this threshold, the specificity was 0.83 and sensitivity 0.77, which is in a similar range to other cancer-specific methods that train on COSMIC subsets and neutral mutations such as CHASM [22]. The AUC shows InCa performs better than Polyphen and SIFT (the receiver operating characteristic (ROC) curve is shown in Figure 2). The model denoted “simple structure” excludes the microenvironment parameters and a lower AUC in this model shows that microenvironment parameters confer predictive contribution. Full prediction metrics for all methods are given in Table 5.

**Table 5:** ROC curve and prediction parameters for optimal thresholds in all tested methods.

	optimal threshold	specificity	sensitivity	accuracy	tn	tp	fn	fp	npv	ppv	1-specificity	1-sensitivity	1-npv	AUC
simple structure	0.53	0.77	0.72	0.74	4125	4845	1880	1250	0.69	0.79	0.23	0.28	0.31	0.82
InCa	0.54	0.83	0.77	0.80	4424	5225	1550	901	0.74	0.85	0.17	0.23	0.26	0.88
FI	0.67	0.74	0.54	0.63	3969	3680	3089	1362	0.56	0.73	0.26	0.46	0.44	0.69
InCa FI	0.56	0.85	0.75	0.79	4495	5106	1722	777	0.72	0.87	0.15	0.25	0.28	0.89
Poly-phen2	0.53	0.65	0.80	0.73	3463	4189	1062	1835	0.77	0.70	0.35	0.20	0.23	0.75
SIFT	0.52	0.75	0.70	0.73	3582	3319	1441	1173	0.71	0.74	0.25	0.30	0.29	0.77
InCa Random	0.52	0.44	0.59	0.52	2320	4012	2803	2965	0.45	0.58	0.56	0.41	0.55	0.51

To ensure that the predictive capacity of our model held despite possible inherent data bias in driver proteins, we re-ran the same prediction assessment iterations on the reduced set of 23 proteins. The InCa AUC dropped to 0.751 and there was a drop in performance of the other predictors, but because this was greatly reduced training set, we concluded our predictive parameters were independent of biases in interface annotations (Figure S15).

We calculated a conservative InCa threshold, based on the cross-validation InCa scores for mutations in the randomly withheld sets, below which 99% of the neutral mutations lied, as 0.778. This was used as the cutoff for prediction of a driver.

### Application of predictor on COSMIC mutations not present in the driver list

**Figure 3:**
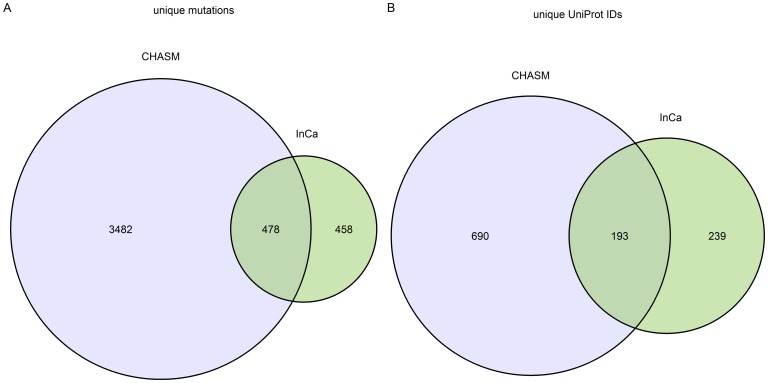
Unique mutations and proteins predicted as drivers by InCa and by CHASM from mutations in COSMIC that were not in the training driver set.

To compare our method to CHASM, we applied InCa and CHASM to the mutations in COSMIC that were not in the driver mutation list. This mutation list consisted of 31471 mutations in 3353 unique proteins. We used the FDR threshold supplied by the authors, 0.2, to determine which mutations were classed as drivers. We retained all mutations with a CHASM FDR score below 0.2 and InCa score above 0.778. This resulted in 478 mutations predicted as drivers by both programs, 458 predicted by InCa only and 3482 mutations predicted by CHASM only (Figure 3). 193 proteins contained mutations predicted as drivers by both programs. 239 proteins had mutations predicted as drivers in InCa only and 690 proteins had some mutations predicted as drivers by CHASM only. 27052 mutations did not score high enough to be predicted as drivers by either InCa or CHASM.

We further explored the list of proteins in the InCa only set with DAVID [55], manual literature mining and inspection. We found that 216/239 (90%) of proteins had functional associations with cancer (Table S5). We deduced that a significant amount of relevant mutations are detected by InCa and CHASM that are not detected by the other.

**Figure 4:**
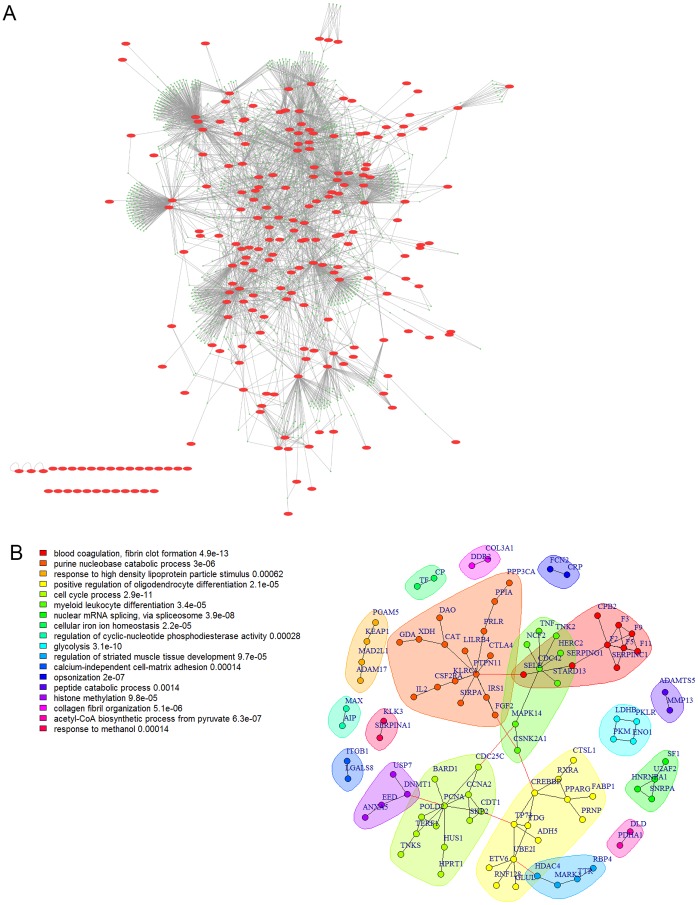
Protein-protein interaction network of genes containing mutations predicted as drivers by InCa only. A) 1-hop network for the proteins containing mutations predicted as drivers in InCa only. The original proteins are shown as red ellipses. Connecting proteins are shown as green circles. 29 of 215 proteins are in a giant component. B) Induced network of connections between original proteins only excluding orphans. The network contains 94 proteins and optimal communities are colour coded. The legend show the top enriched GO BP term in each community.

To further explore these proteins, we created an induced protein-protein interaction network (PPIN) using the ROCK web server [56]. 215 proteins were mapped and we found that by taking a 1-hop network, 195 of 215 of the proteins (91%) were connected in a large connected component (Figure 4A). Communities from this network were enriched in GO BP terms that are characteristic of cancer functions (Table S6). The network connecting only the list proteins directly contained 217 proteins and still gave a large connected component containing of 74 proteins. The network excluding orphan nodes is shown in Figure 4B. Several visually discernible communities were apparent. A few of these were enriched in canonical cancer functions like signal transduction, cell proliferation and DNA metabolism, but also in coagulation and RNA processing. Blood coagulation was recently found to have important contributions to cancer pathogenesis [57], [58]. Similarly, RNA processing also has been recently shown to be involved in cancer pathogenesis [59], [60]. Community membership is listed in Table S7.

### Investigation of BARD1 mutation

**Figure 5:**
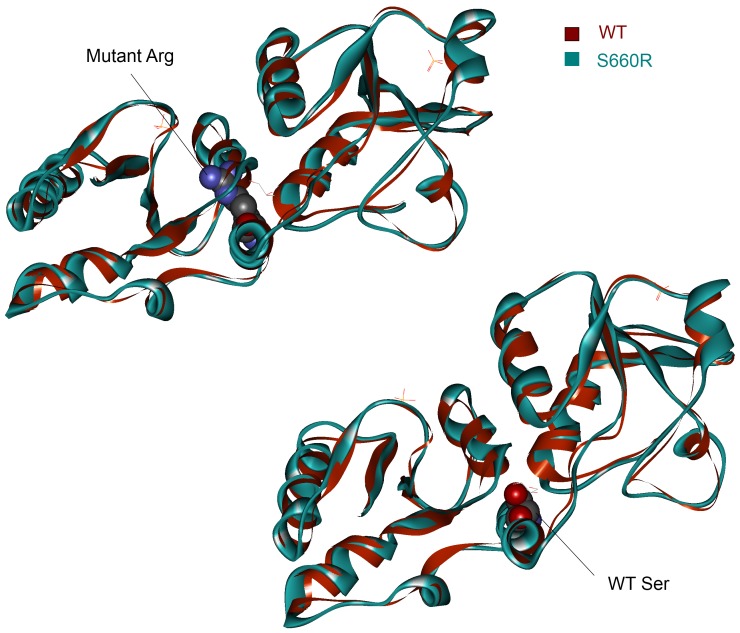
Energy minimisation of the S660R mutation in BARD1. The top panel shows the mutant Arg residue in the mutant structure causing a steric clash with the helix opposite. The wild-type (WT) structure is shown in red overlaid with the mutatnt structure in blue. The bottom panel shows the WT Ser residue.

BARD1 has been previously characterised as an important contributor to breast and ovarian cancer [61]. We characterised the BARD1 S660R mutation in more detail (Figure 5). By doing an energy minimisation on the mutated structure, we found that the effect is similar to a previously documented C645R mutation that destabilises the BRCT1 fold [62]. The arginine residue cannot be accommodated and produces a similar effect to the C645R and we therefore speculate this could be the mechanism that contributes to its carcinogenicity.

### Domain characterisation of INCA and CHASM driver mutation predictions

To investigate the distribution of domains in the InCa and CHASM driver mutation predictions, we plotted these and compared them (Fig. S16 and Table S8). We found that CHASM mutations are enriched in kinase domains whereas InCa mutations are more evenly spread out throughout several Pfam domains. This might suggest that sequence-based predictors like CHASM might have a prediction bias for certain genes or domains that is less pronounced in structure-based predictors.

### Application of InCa to a lung adenocarcinoma data set

To further show the applicability if InCa to NGS mutation data sets, we parsed the mutations from a recent lung adenocarcinoma study [63] and applied InCa on the missense mutations. The study contained 7659 missense mutations. We excluded mutations in our training and testing sets; of the remaining mutations, 622 mapped to structures and obtained InCa scores. 16 were predicted as carcinogenic and were all in genes that were associated with cancer in the literature (Table S9). The top scoring mutation was in BRAF. Several of these mutations occurred in functional domains or regions, such as the RNA-recognition motif (RRM1) domain of SNRPA and the inhibitor TIMP2 binding region of MMP2. The latter two proteins form a complex highly associated in other cancers [64], [65].

## Discussion

We have performed an in-depth structural analysis of missense mutations in both driver mutations from COSMIC and neutral mutations in 1k. Using these data we have derived parameters for a new mutation carcinogenicity predictor that is based on structural and protein interface parameters.

In both datasets it was surface residues that were more often mutated. This is an expected finding for 1k since surface residue mutations may play a key role in evolutionary diversification with low immediate impact on protein structure and function, whereas protein core mutations tend to have a much more severe effect on protein structure and stability. Driver mutations found in surface residues may be detrimental for a number of reasons. Partially exposed side chains of surface residues were more enriched in driver mutations, possibly because they have the potential to cause significant local structural deformation, coupled with the fact that such substitutions were in principle non-conservative. In addition, large areas on the protein surface may serve as yet unidentified interface forming sites for transient or less specific interactions, particularly considering the very high protein density in the cell, and hence may be more constrained in tolerating physicochemically dissimilar residues. In agreement with our findings, it was recently reported that driver mutations are clustered on surface patches [14].

We observed specific biases in driver for mutated residues and their resulting amino acid substitutions. Driver mutations were enriched in mutated glycine and tryptophan residues as well as buried cysteines. Interestingly, driver mutations occurred less frequently on α-helices and more often on coils compared to 1k, implying a more subtle effect than simple secondary structure perturbation. Talavera et al. also observe that cancer-related mutations have an overall tendency to occur near specific amino acids possibly due to a positional bias for proximity to surface residues and therefore hydrophilic neighbours [14], although it is difficult to compare the data directly as different distance cut offs were used. We found that buried cysteines often mutated to tyrosines and tryptophan and tyrosine residues often mutated to serine and arginine respectively. Driver mutations are also commonly found in bond-forming residues in protein binding interfaces, which may contribute to signaling aberrations and lesions responsible for the cancer phenotype.

Our findings are generally consistent with the hypothesis that interface disruptions are a significant factor in generating cancer phenotypes. The amino acid residues neighbouring mutation sites, both on the same molecule as well as its binding partner, displayed significant compositional biases across the driver and 1k datasets (Figures S12, S13 and Dataset S2, S3). The physicochemical compatibility of the substitution as well as loss of electrostatic contacts with the surrounding residues often linked driver mutations to more adverse interface binding defects.

Using sequence and structural parameters that included residue neighbourhood, interface electrostatic interactions and the sequence conservation as expressed by the FI score [29], we constructed a predictive additive model that discriminates between cancer-associated and neutral mutations. While it has been shown that cancer related mutations occur more often in conserved residues [14], we showed that using additional structural information such as neighbouring residues and interface electrostatic bond information yields better prediction performance and that performance is slightly increased from the inclusion of the FI score. The performance of the predictor indicates that all these parameters are important for assessing cancer mutations and in that respect a simple examination of primary sequence conservation around the mutated residue may lead to the conclusion that cancer mutations can occur at any position in the protein [14], although there are clear mutational hotspots defined by higher structure orders.

While the caveat of our method is that structural information is required, we found several instances where FI scores are unavailable, presumably due to insufficient sequence information or size of protein families required for the computation. For these cases, our structural method can still be used to predict cancerous character.

A comprehensive analysis of neighbouring residues of candidate mutations would give value for discerning future potential cancer mutations. With the advent of structural genomics initiatives, it will be increasingly practical to investigate structures of uncharacterized proteins if they are relevant and more data will be available. While structural predictors have been used extensively in previous studies [23], [25], we show that added value can be obtained from structural information from comprehensive analyses such as those in PISA and information of neighbouring residues of the mutation.

In this work we focused on the classification of missense mutations as their effect on protein function is more difficult to interpret. While the 1000 Genomes project derives data from non-diseased individuals it may contain a number of mutations that can drive or predispose to cancer later in life. While in its current form InCa may be subject to these issues, it may be possible to fine tune its resolving power with interaction network perturbation analysis features particularly as the cancer phenotype often is the result of multiple signaling lesions [66].

The observation that CHASM predictions were enriched in kinase domains suggests there could be a degree of gene-centricity that is created by the datasets used for training and the method. We also observed instance where FI scores gave false positives, such as the BRAF D594V mutation, which decreases ERK stimulation [67] but has a high FI score (4.46). The mutation also occurred in our training data set and therefore highlighted that all predictors trained on non-experimentally validated and characterised drivers may potentially suffer from a few false positives in the training set. Our results that InCa and CHASM detect different mutations, albeit with large overlap, suggest that several different methods should be used when determining mutation carcinogenicity and that shortcomings of each individual methods in isolation should be considered. While all current methods for predicting the effects of single mutations are powerful, the effect of mutations is often combinatorial and so the context of each mutation should be taken into account for better biological interpretation.

## Supporting Information

Dataset S1 **Data fame of mutations with all parameters.**
(ZIP)

Dataset S2 **Neighbouring residue profiles for mutations classed by WT residue.**
(ZIP)

Dataset S3 **Neighbouring residue profiles for mutations classed by substitution.**
(ZIP)

Figure S1 **Unique mutations and proteins in the cancer driver and neutral datasets.**
(TIF)

Figure S2 **Distributions of amount of mutations per protein.**
(TIF)

Figure S3 **Background distribution of amino acid frequencies in 1000 Genomes and COSMIC.** A) Normalised frequencies (denoted P(x) for density) of amino acids in each set. The frequencies are ordered according to average value. B) Normalised frequencies for each sample divided by area. C) Pearson correlation coefficients of each set pair. Smooth trendlines are overlaid in red on plots in the bottom left part of the panel. D) as C) for each sample divided by area. The dataset each series denotes is described below. PDB, NR, Hsa: All non-redundant human crystallised sequences 1k: 1000 Genomes set of non-redundant human crystallised sequences COSMIC: Cosmic set of non-redundant human crystallised sequences Uniprot: Entire Uniprot sequences Uniprot PDB: Entire Uniprot sequences which have PDB entries Uniprot 1k: Entire Uniprot sequences in the 1000 Genomes set Uniprot COSMIC: Entire Uniprot sequences in Cosmic Uniprot 1k and COSMIC: Entire Uniprot sequences in Cosmic and 1000 Genomes.
(TIF)

Figure S4 **Mutation severity in neutral and driver mutations by physicochemical change of substitution, mutational permissiveness according to BLOSUM 62, Dayhoff, FI and distance to interface.** The first row shows plots of change in amino acid physiochemical character incurred by the substitution. The driver mutations show a greater change in physiochemical character, thus presumably incurring a greater disruption to protein stability/function. The second row shows boxplots of mutation substitution severity according to the amino acid substitution values in BLOSUM 62 (EBI). The 1k mutations hover around 0, whereas the driver mutations have less permitted mutability. Rows 3 and 4 show that same using Dayhoff (EBI) (see text) and FI scores. Rows 5 and 6 show minimum and mean distances to interfaces. Because unique residues can have multiple PDB files and each PDB file can have many interfaces, there are several distances from each residue to each interface. The proximity of driver mutations to the interface suggests that cancer mutations tend to disrupt interfaces.
(TIF)

Figure S5 **Mutation severity in neutral and driver mutations by physicochemical change of substitution, mutational permissiveness according to BLOSUM 62, Dayhoff, FI and distance to interface, using the reduced set of 23 proteins with both neutral and driver mutations.**
(TIF)

Figure S6 **Propensities in mutations split by area and 2ry structure separately.** A) Normalised frequency of occurrences of mutations in each area. Cancer mutations occur more frequently in buried and interface areas than neutral mutations. B) Normalised frequency of occurrences of mutations in secondary structures. Most carcinogenic mutations occur in coils and beta sheets and less in helices. There is a small but significant difference (Fisher's test with a two-sided alternative hypothesis) between the driver and 1k samples in both cases. C) Fractions of observed normalised frequency to expected normalised frequency (all residues in proteins) for each area. D) Fractions of observed normalised frequency to expected normalised frequency for each secondary structure.
(TIF)

Figure S7 **Propensities in mutations split by area and 2ry structure separately, using the reduced set of 23 proteins with both neutral and driver mutations.**
(TIF)

Figure S8 **Enriched mutations in area, secondary structure and WT residue comparing neutral and driver mutations.** Red denotes enriched classes in drivers and blue denotes enriched classes in neutral mutations. A) Enrichment in driver mutations divided by area and WT residue (*s*_o_). B) Enrichment in driver mutations divided by area, secondary structure and WT residue (*s*_o_).
(TIF)

Figure S9 **Heatmaps of normalised substitution frequencies and enrichment comparing neutral and driver mutations.** Red denotes enriched classes in drivers and blue denotes enriched classes in neutral mutations. A) Driver/neutral fraction of normalised frequencies for mutations by area and substitution. B) Statistically overrepresented substitution frequencies by area (*s*_o_).
(TIF)

Figure S10 **Heatmaps of normalised substitution frequencies and enrichment comparing neutral and driver mutations for mutation classes separated by area and secondary structure.** Red denotes enriched classes in drivers and blue denotes enriched classes in neutral mutations. A) driver/neutral fraction of normalised frequencies for mutations by area and substitution. B) Statistically overrepresented substitution frequencies by area (*s*_o_).
(TIF)

Figure S11 **Neighbouring residue profile of targeted wild-type buried Cys mutations in the 5 Å vicinity.**
(TIF)

Figure S12 **Salt bridge enrichment in interface residues for charged residues targeted by COSMIC mutations.** Densities (denoted P(x)) are shown for mutations of each amino acid in both sets with their associated p-values comparing 1k and COSMIC with a two-sample Wilcoxon test using a one-sided alternative hypothesis.
(TIF)

Figure S13 **Neighbouring residue profiles of mutated interface residues in the 5 Å vicinity.** The 5 Å neighbourhood profiles are shown grouped by mutated wild-type residue and by area. Shown are signed p-values using a two-sample Wilcoxon test. Red denotes enrichment in drivers and blue denotes enriched classes in neutral mutations.
(TIF)

Figure S14 **Neighbouring residue profiles for F→S mutations and G→V mutations in the 5 Å vicinity of the muatations.** Profiles of neighbouring amino acid residues on the mutated side of the interface are denoted by their codes and those on the opposite side of the interface are denoted with “Opp”. Neighbouring residues of interface phenylalanine mutations in neutral mutations and drivers. Normalised (relative) frequencies (P(x)) are shown for each amino acid for 0–5 Å. The “Opp” suffix denotes the molecule on the opposite side of the interface to the mutated molecule.
(TIF)

Figure S15 **ROC curve for Inca and other mutation predictors using the reduced set of 23 proteins with both neutral and driver mutations.**
(TIF)

Figure S16 **Pfam domain distribution in InCa and CHASM predicted driver mutations.** A) InCa only. B) CHASM only.
(TIF)

Table S1 **Definitions of topological areas.**
(XLS)

Table S2 **Physicochemical properties of amino acids.**
(XLS)

Table S3 **Top mutated proteins for neutral mutations and driver muations.**
(XLS)

Table S4 **Model parameters and importance in InCa.** The %incMSE is the increasing in mean of the error of a tree (mean square error (MSE)) for regression and misclassification in the forest when the observed values of this variable are randomly permuted in the “out of bag” samples. The IncNodePurity is the total decrease in node impurities from splitting on the variable, averaged over all trees.
(XLS)

Table S5 **Cancer functions for proteins containing mutations that were predicted as drivers by InCa only.**
(XLS)

Table S6 **Enriched GO BP terms in communities of the protein-protein interaction network of proteins containing mutations that were predicted as drivers by InCa only.**
(XLS)

Table S7 **Community membership of proteins in the protein-protein interaction network of proteins containing mutations that were predicted as drivers by InCa only.**
(XLS)

Table S8 **Pfam domain counts for genes from COSMIC mutations not in drivers that were predicted as drivers by InCa only and CHASM only.**
(XLS)

Table S9 **InCa scores and predictions for the lung adenocarcinoma data set.**
(XLS)

Text S1 **Supporting methods.**
(DOC)
