# Supplementary figures and images for: Deriving a Mutation Index of Carcinogenicity Using Protein Structure and Protein Interfaces (part 1 of 3)
Source: PLoS One. 2014 Jan 15;9(1):e84598. doi: 10.1371/journal.pone.0084598 (PMC3893166; doi:10.1371/journal.pone.0084598)

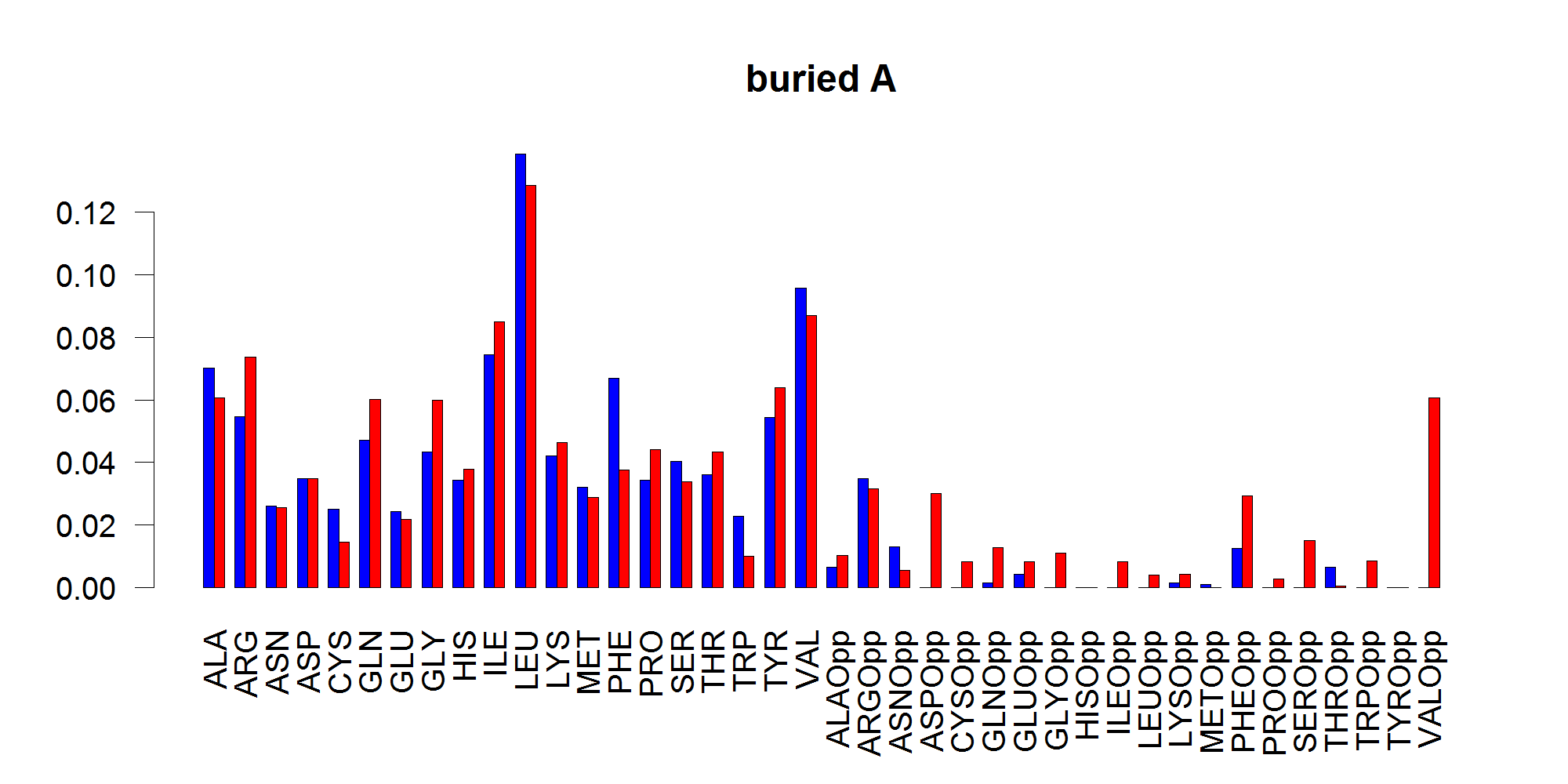

Supplement: Dataset S2 — Neighbouring residue profiles for mutations classed by WT residue. (ZIP) [file pone.0084598.s002.zip › neighbour_1/buried_A.tif]

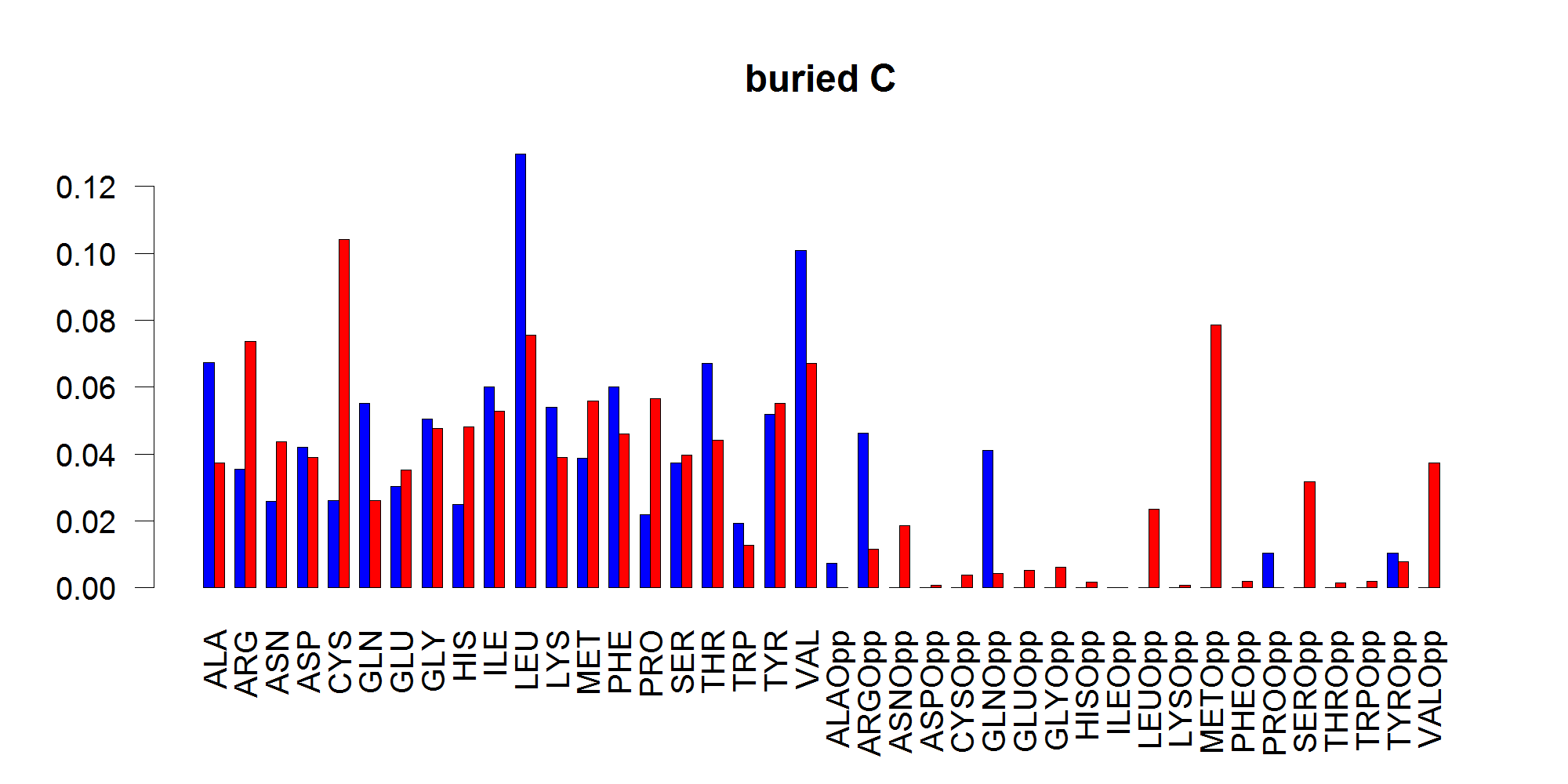

Supplement: Dataset S2 — Neighbouring residue profiles for mutations classed by WT residue. (ZIP) [file pone.0084598.s002.zip › neighbour_1/buried_C.tif]

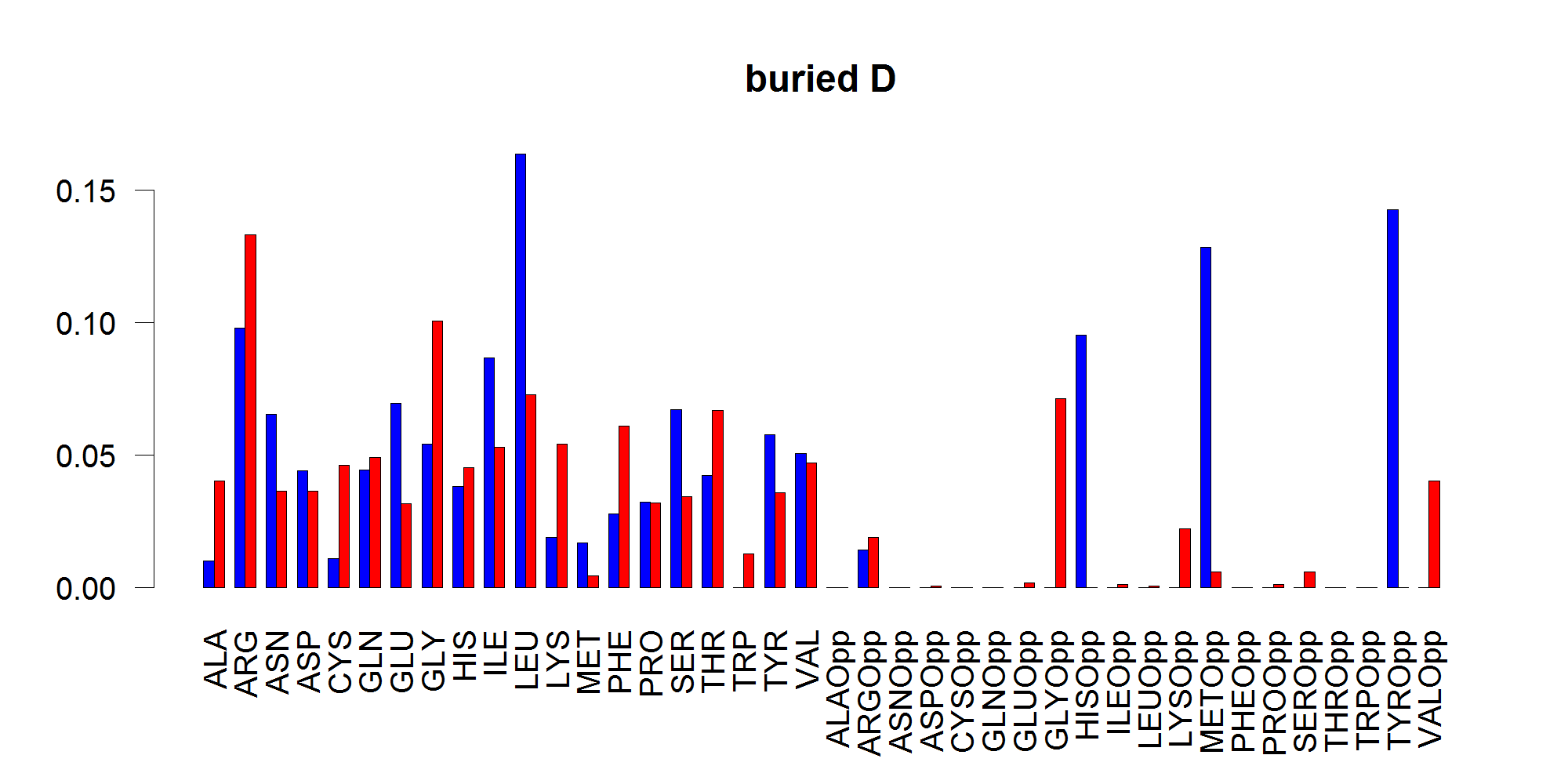

Supplement: Dataset S2 — Neighbouring residue profiles for mutations classed by WT residue. (ZIP) [file pone.0084598.s002.zip › neighbour_1/buried_D.tif]

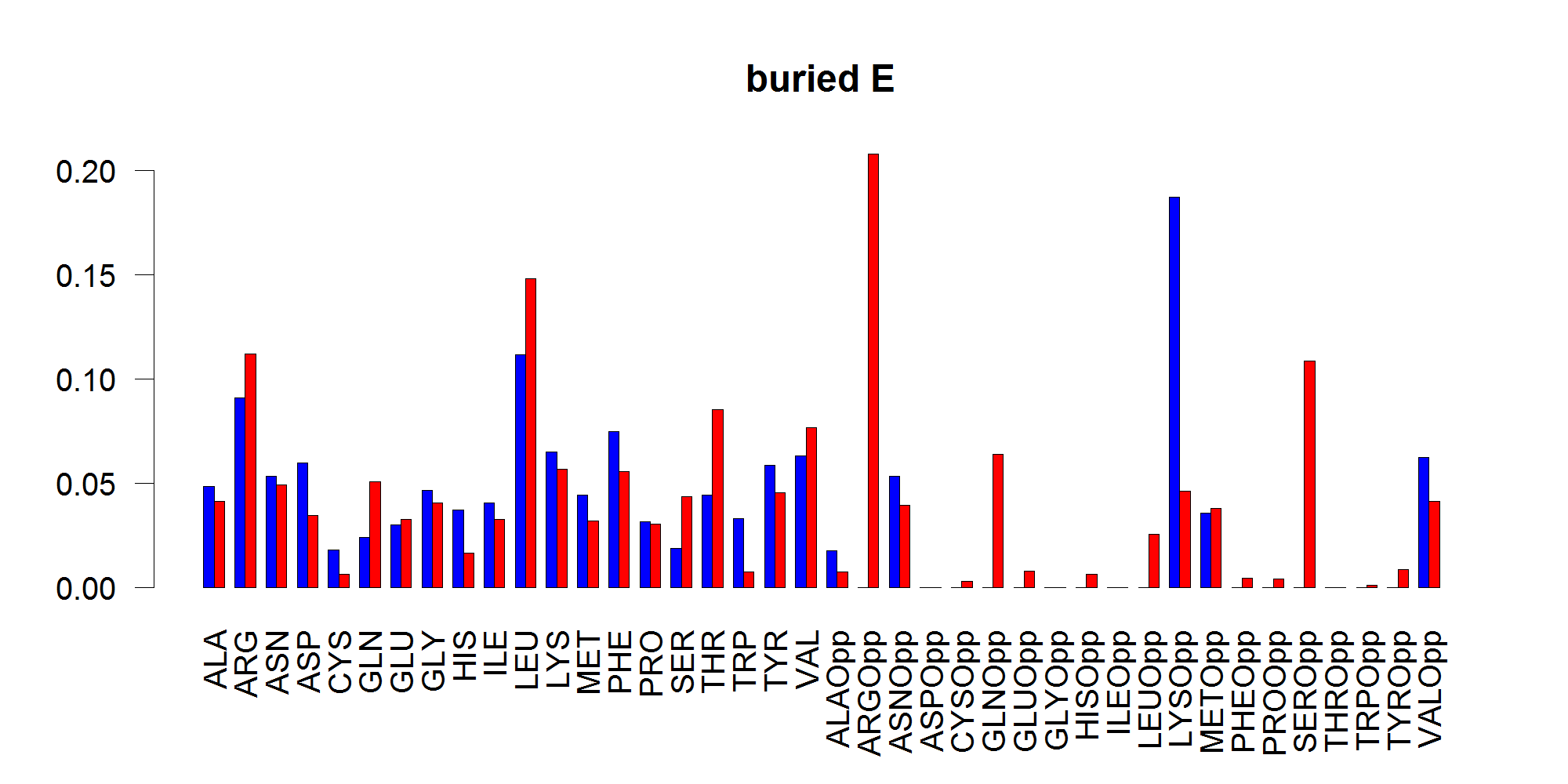

Supplement: Dataset S2 — Neighbouring residue profiles for mutations classed by WT residue. (ZIP) [file pone.0084598.s002.zip › neighbour_1/buried_E.tif]

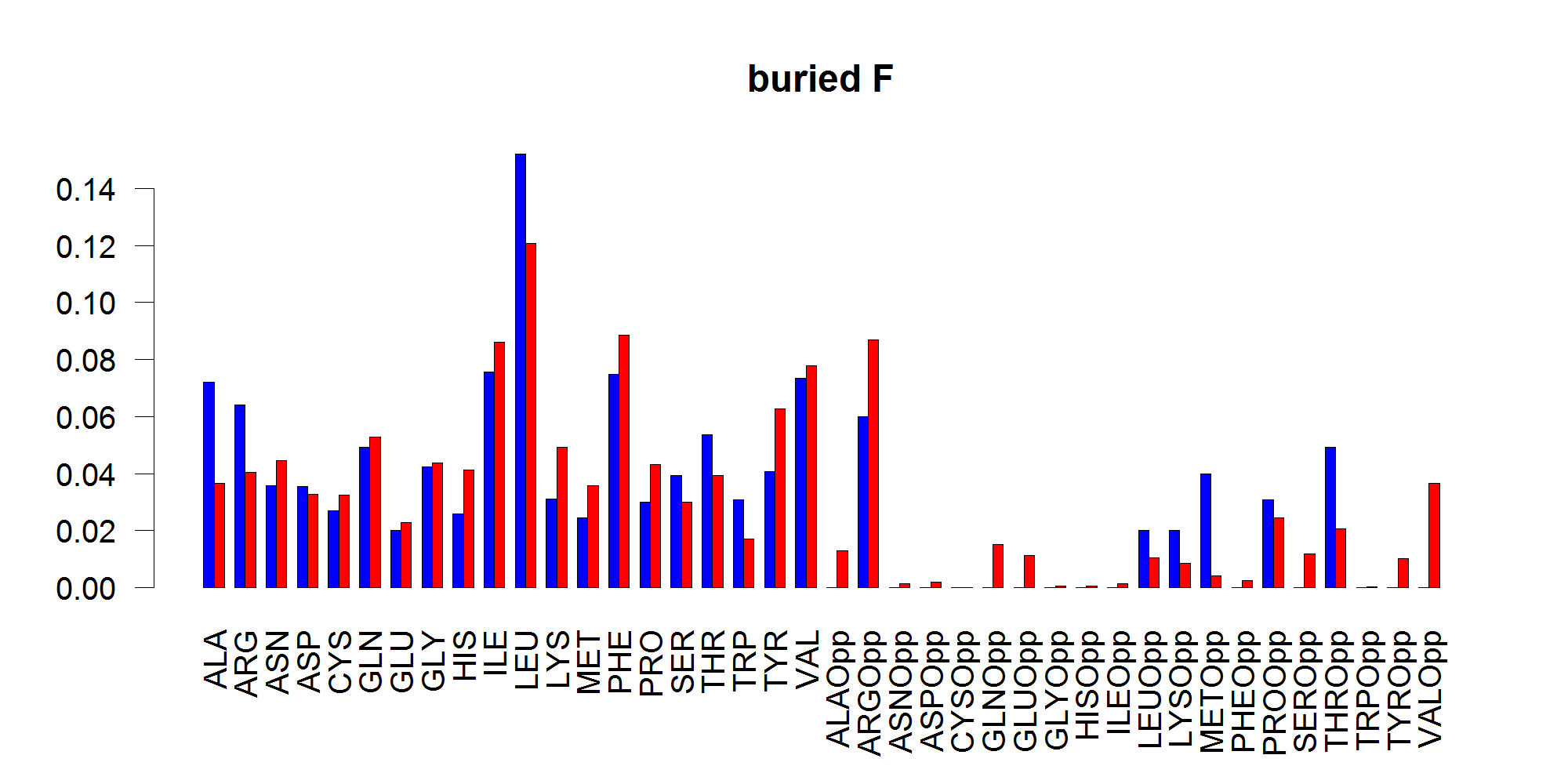

Supplement: Dataset S2 — Neighbouring residue profiles for mutations classed by WT residue. (ZIP) [file pone.0084598.s002.zip › neighbour_1/buried_F.tif]

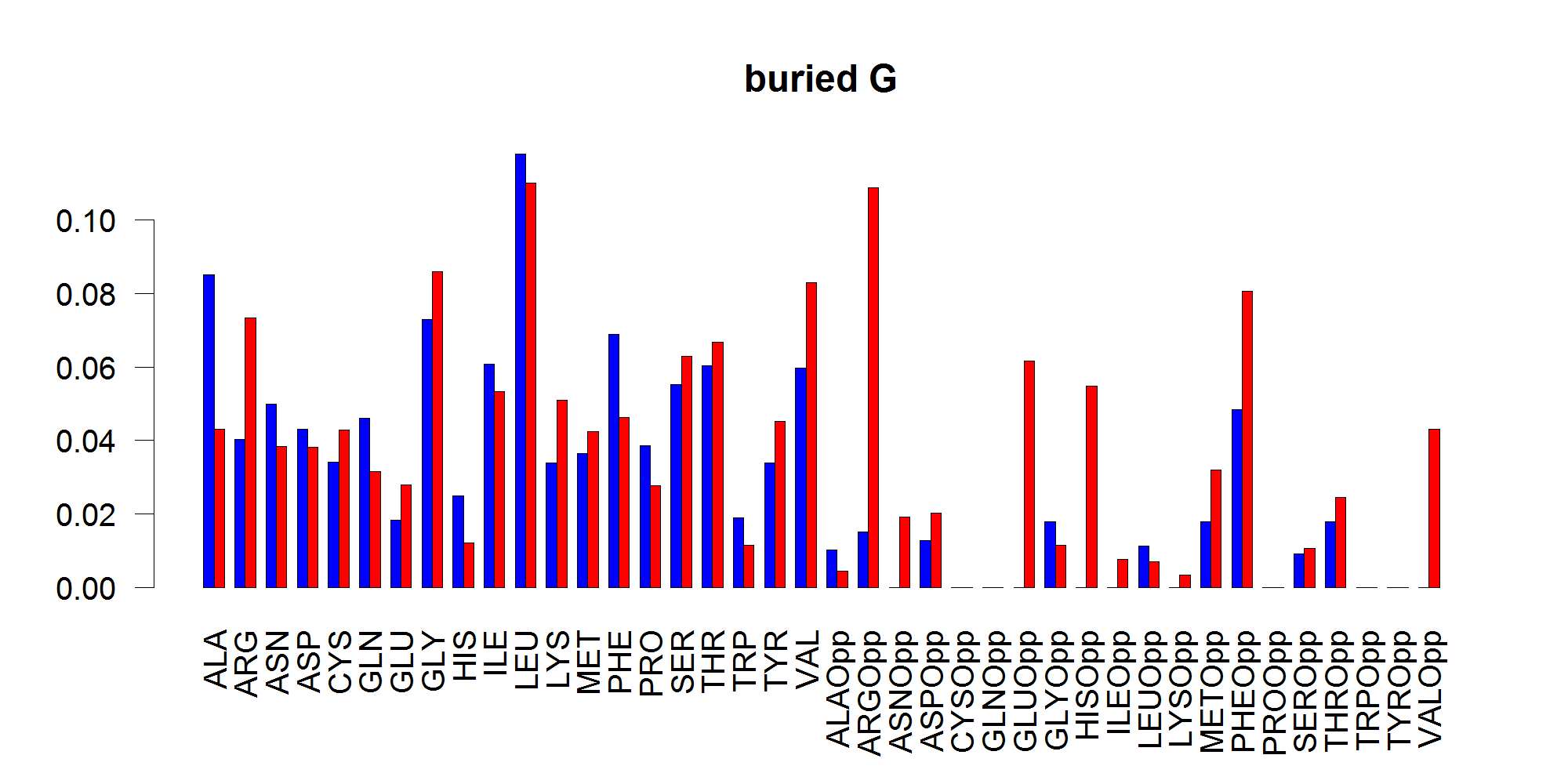

Supplement: Dataset S2 — Neighbouring residue profiles for mutations classed by WT residue. (ZIP) [file pone.0084598.s002.zip › neighbour_1/buried_G.tif]

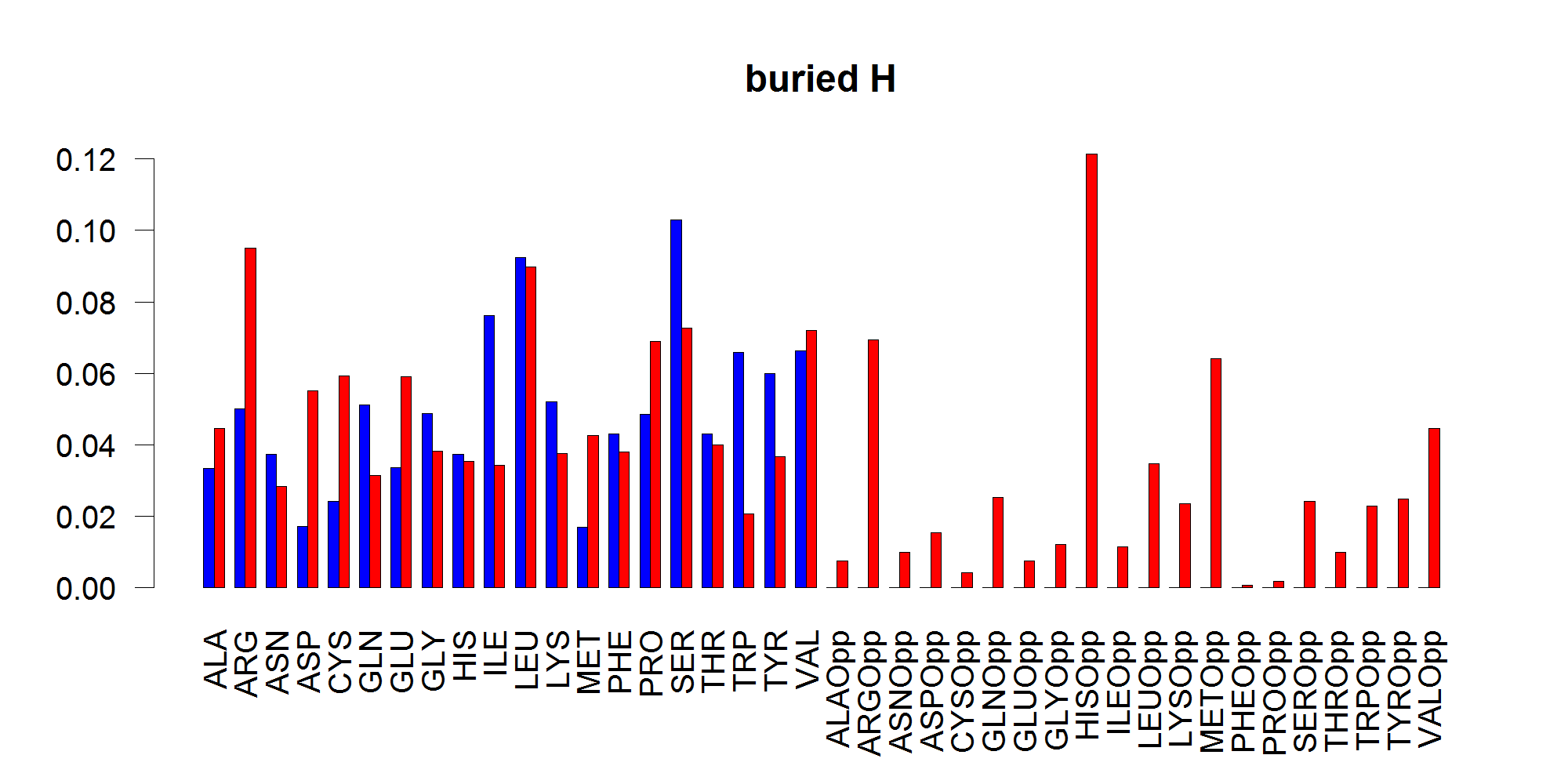

Supplement: Dataset S2 — Neighbouring residue profiles for mutations classed by WT residue. (ZIP) [file pone.0084598.s002.zip › neighbour_1/buried_H.tif]

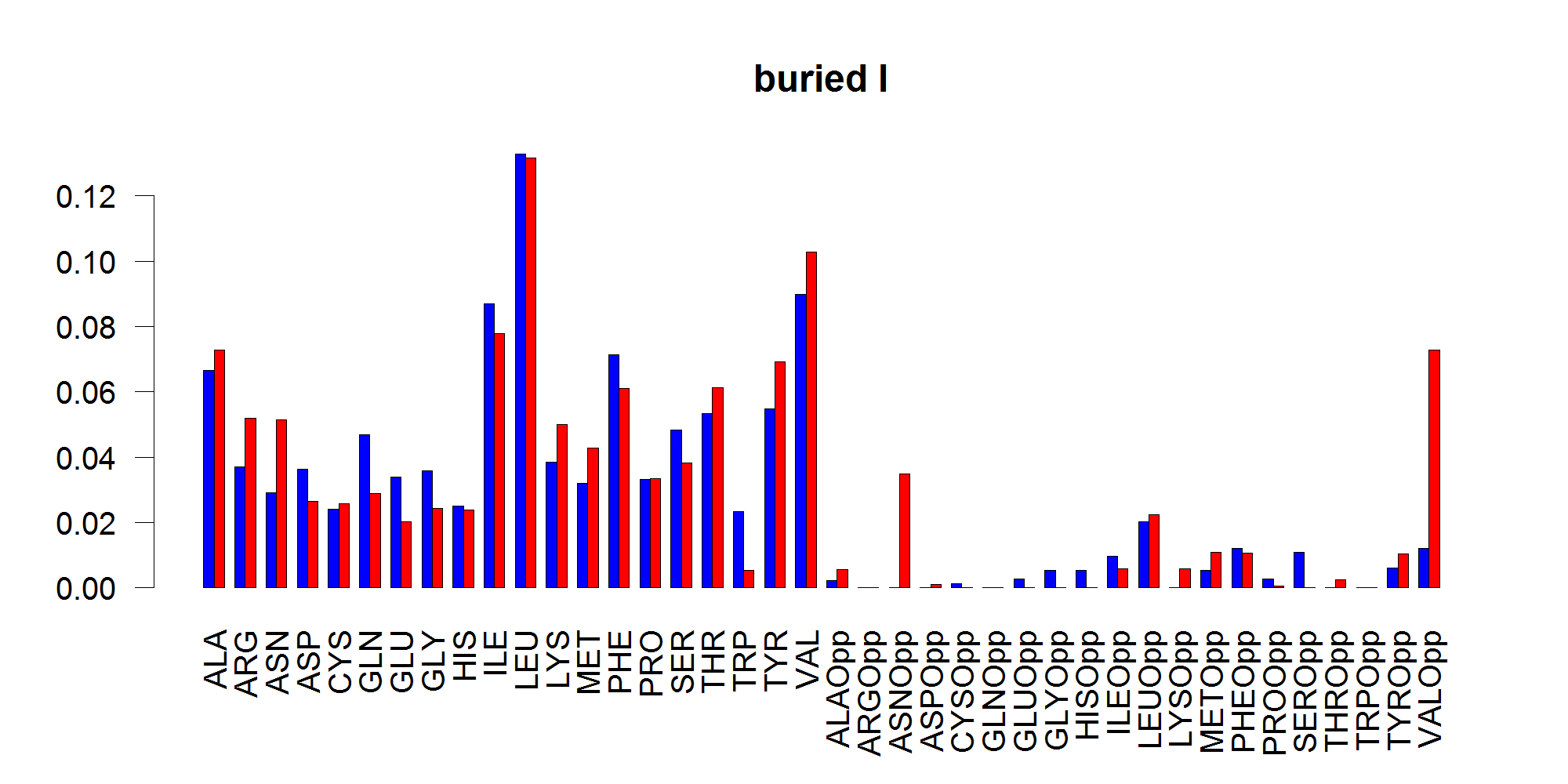

Supplement: Dataset S2 — Neighbouring residue profiles for mutations classed by WT residue. (ZIP) [file pone.0084598.s002.zip › neighbour_1/buried_I.tif]

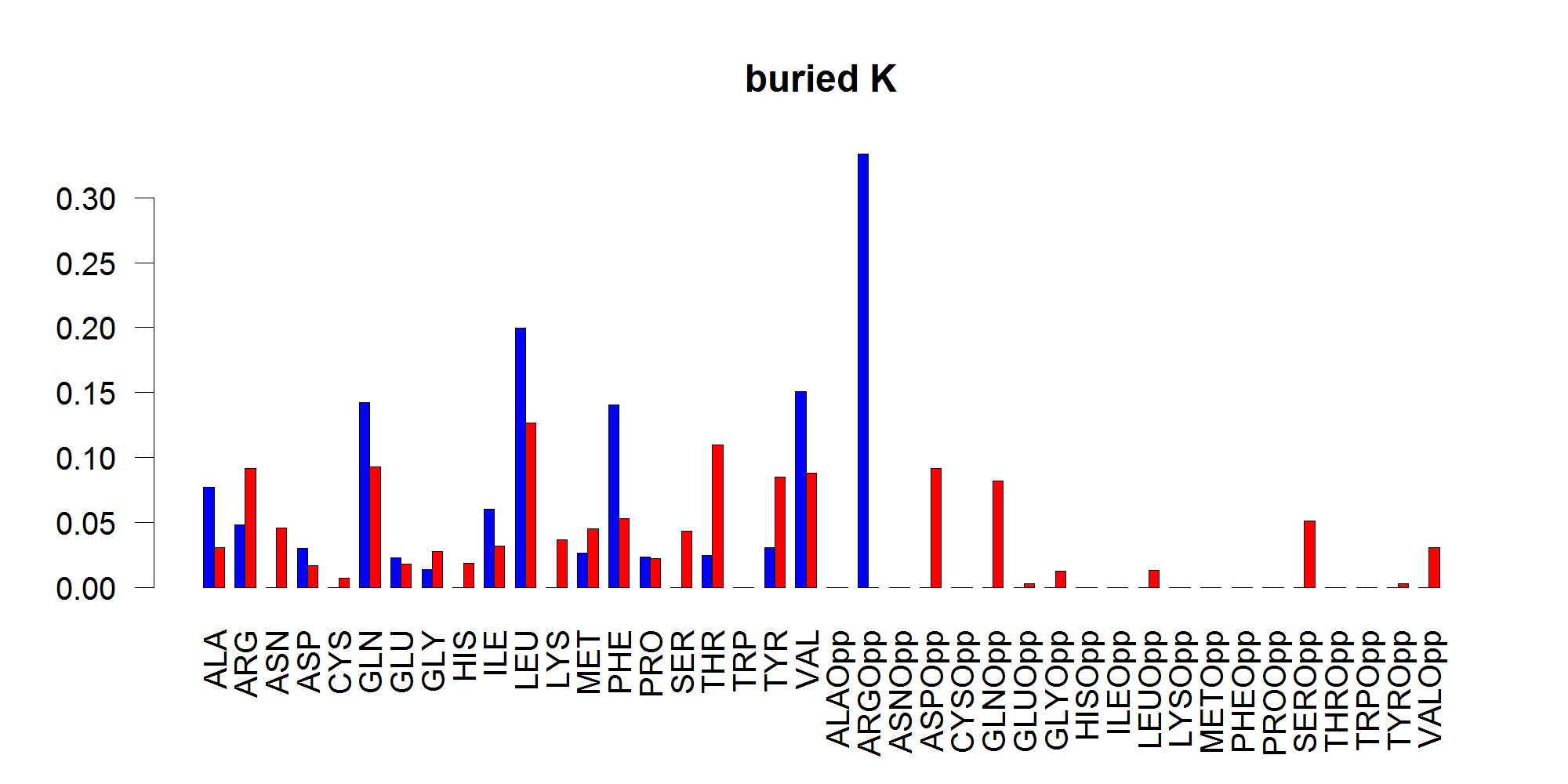

Supplement: Dataset S2 — Neighbouring residue profiles for mutations classed by WT residue. (ZIP) [file pone.0084598.s002.zip › neighbour_1/buried_K.tif]

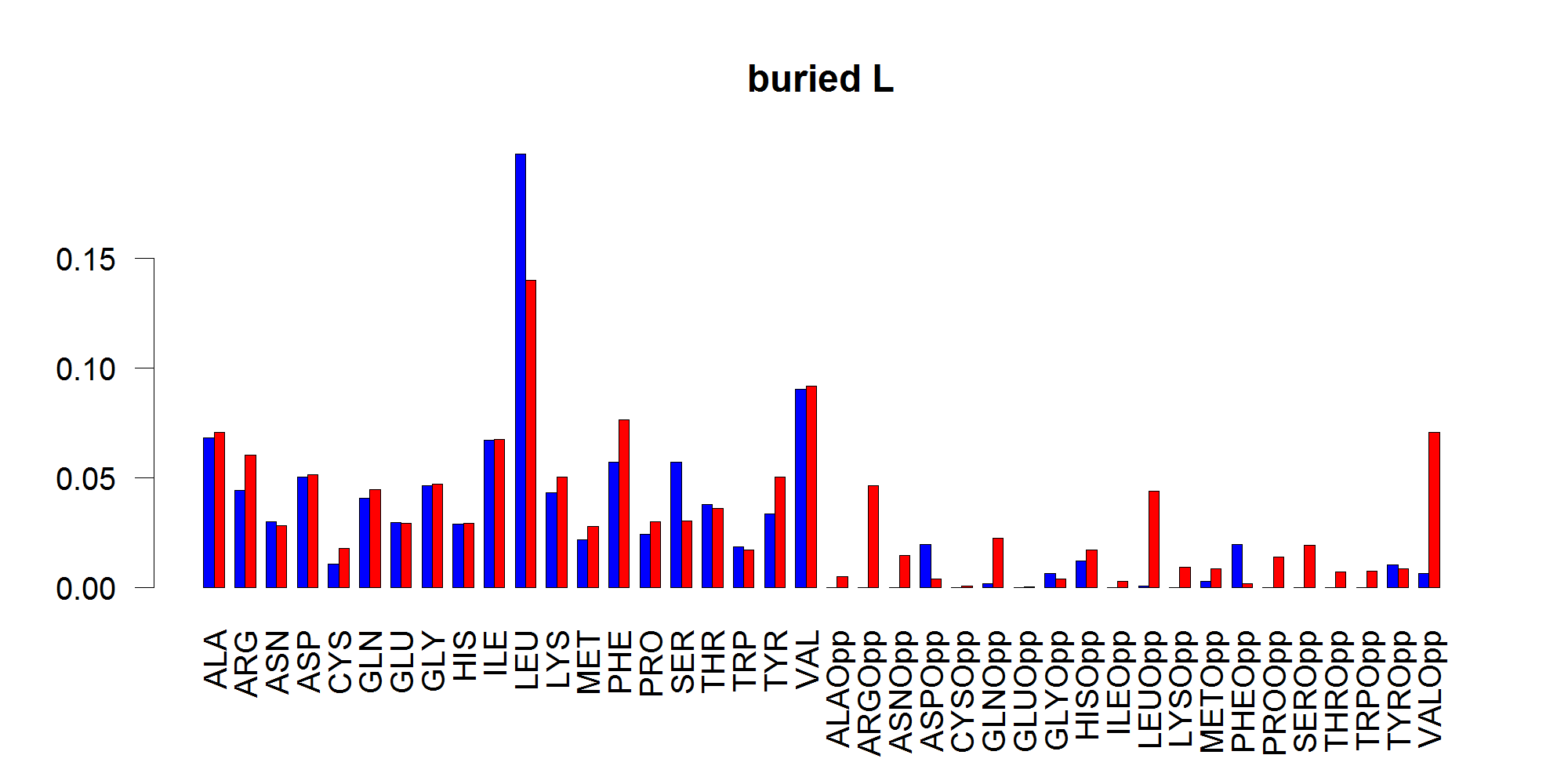

Supplement: Dataset S2 — Neighbouring residue profiles for mutations classed by WT residue. (ZIP) [file pone.0084598.s002.zip › neighbour_1/buried_L.tif]

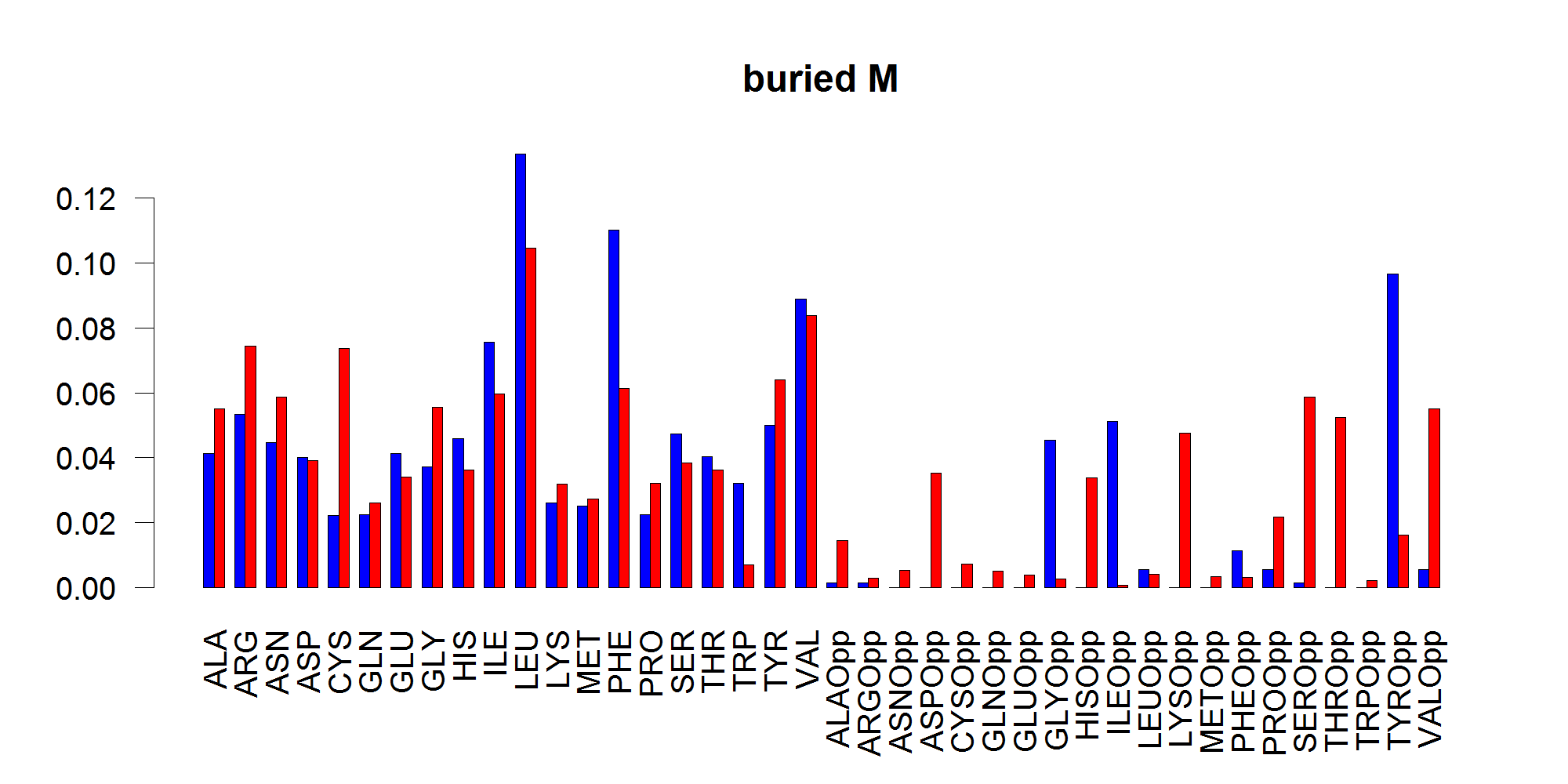

Supplement: Dataset S2 — Neighbouring residue profiles for mutations classed by WT residue. (ZIP) [file pone.0084598.s002.zip › neighbour_1/buried_M.tif]

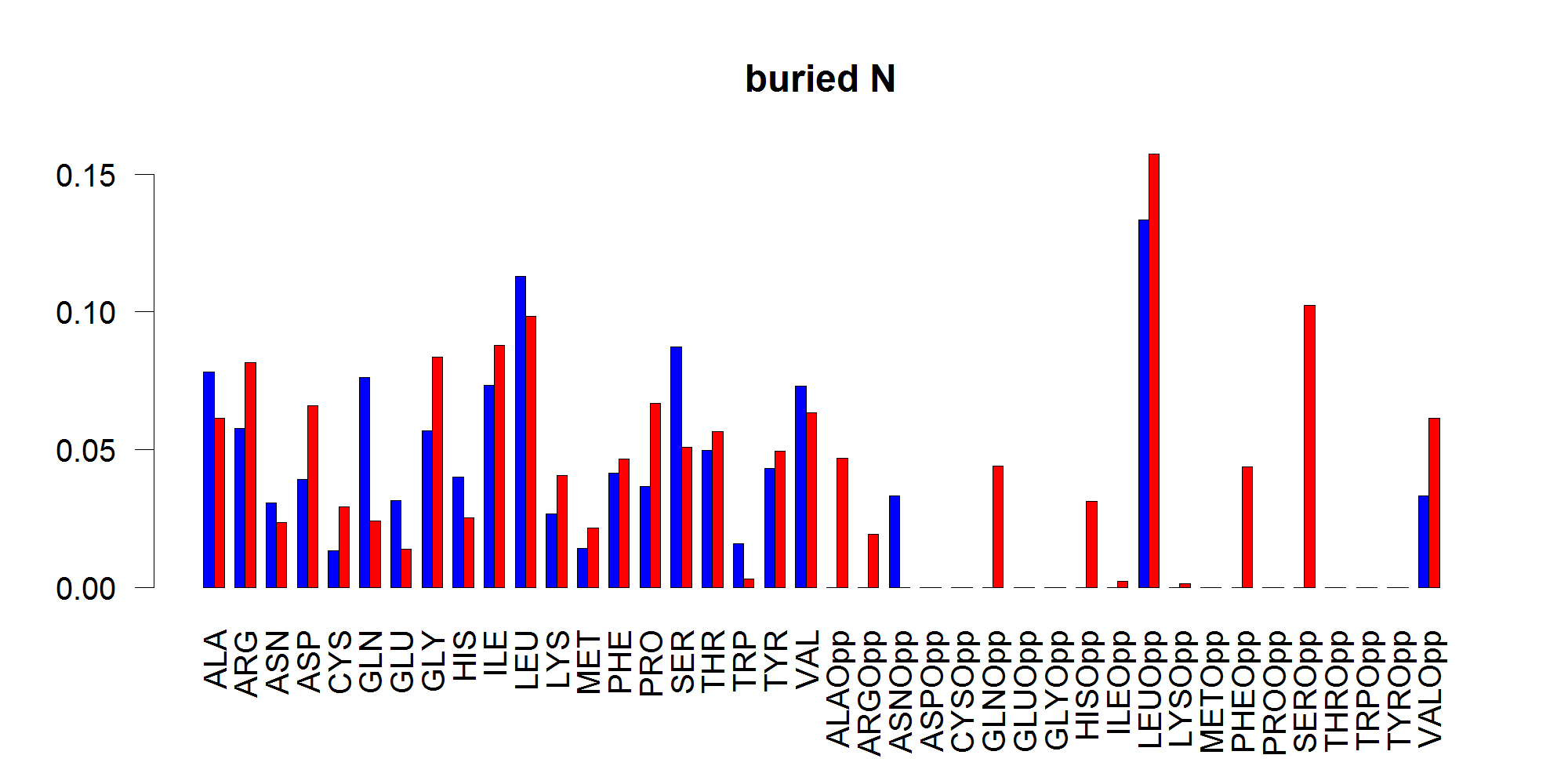

Supplement: Dataset S2 — Neighbouring residue profiles for mutations classed by WT residue. (ZIP) [file pone.0084598.s002.zip › neighbour_1/buried_N.tif]

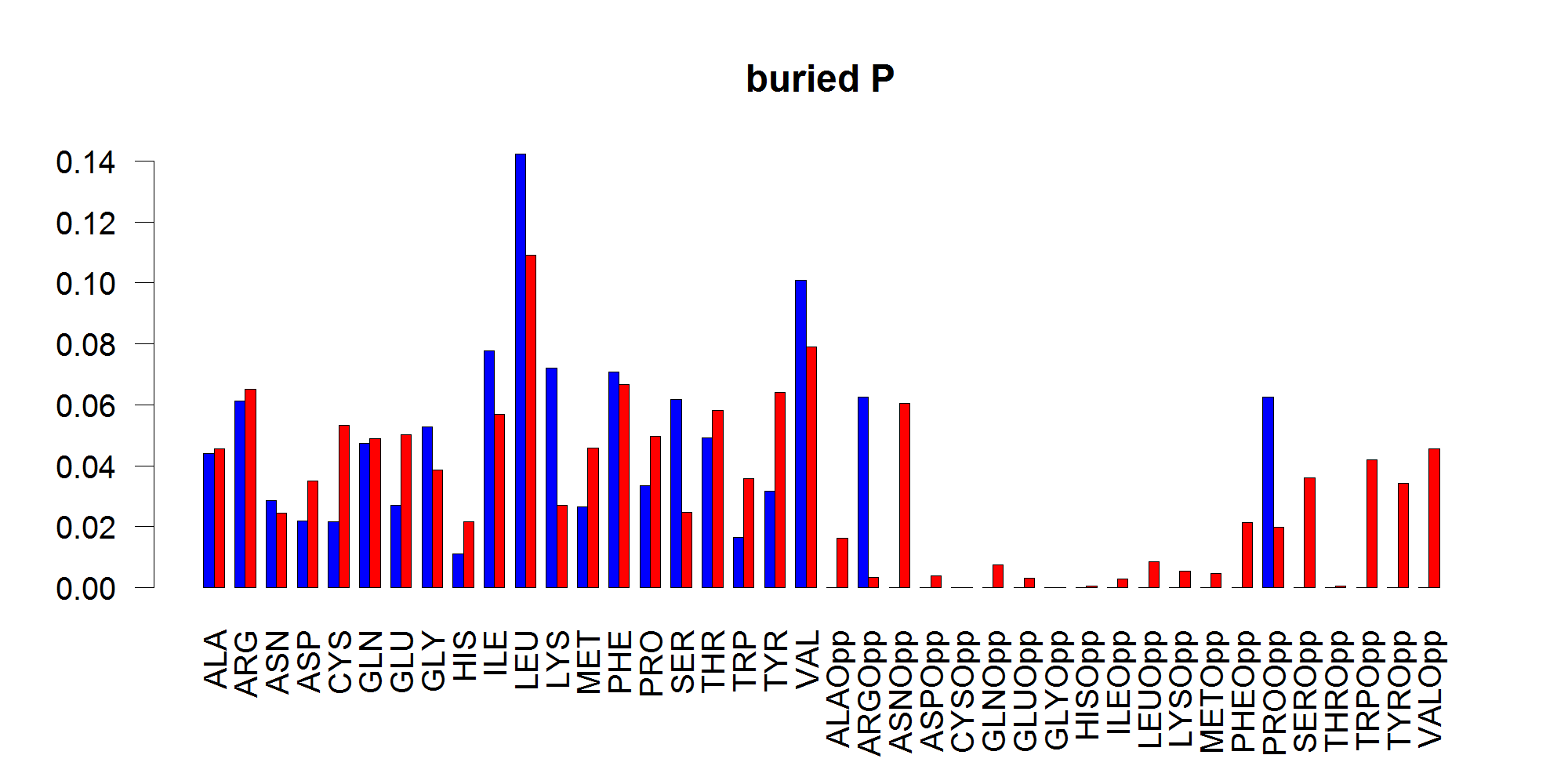

Supplement: Dataset S2 — Neighbouring residue profiles for mutations classed by WT residue. (ZIP) [file pone.0084598.s002.zip › neighbour_1/buried_P.tif]

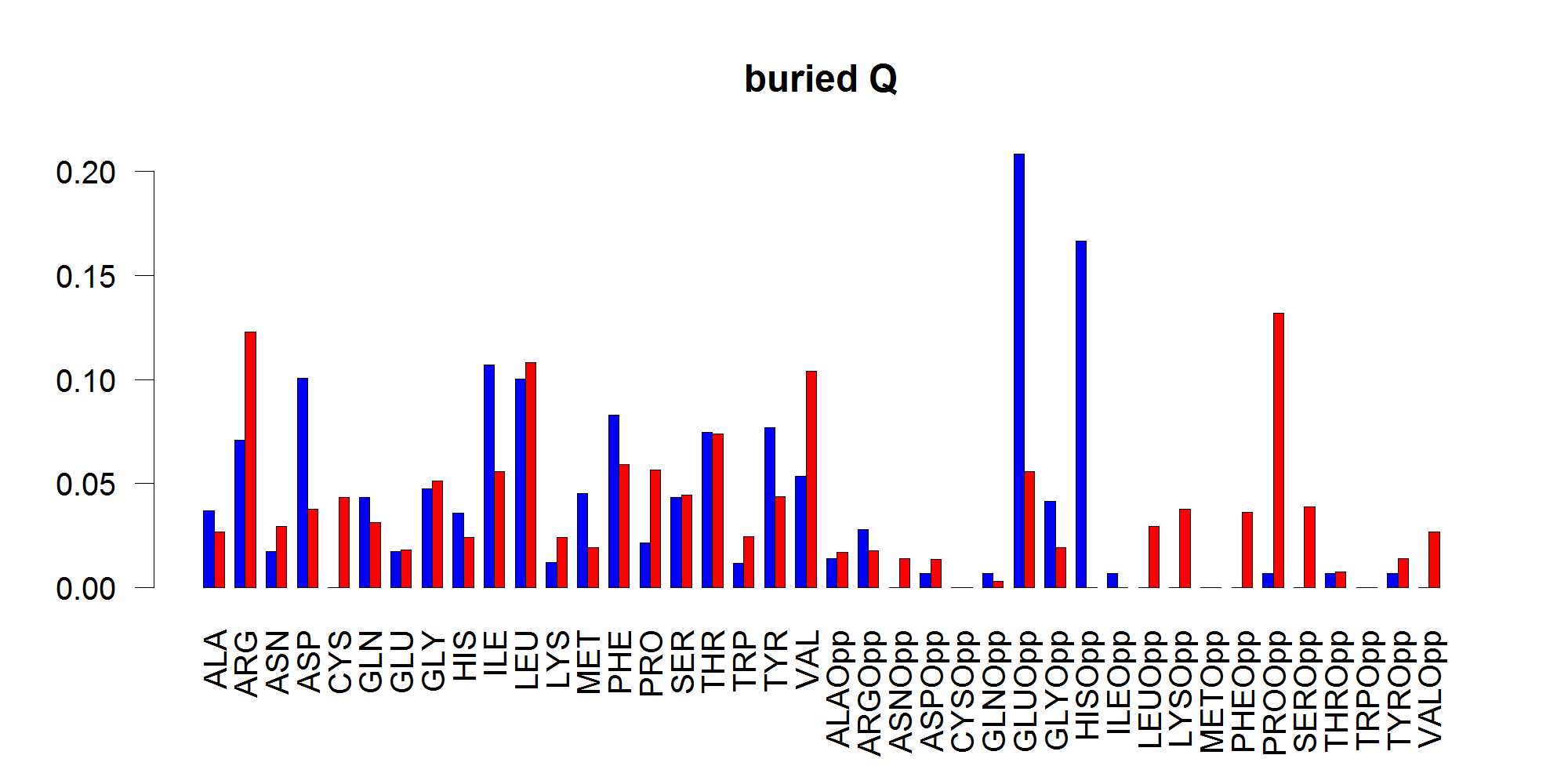

Supplement: Dataset S2 — Neighbouring residue profiles for mutations classed by WT residue. (ZIP) [file pone.0084598.s002.zip › neighbour_1/buried_Q.tif]

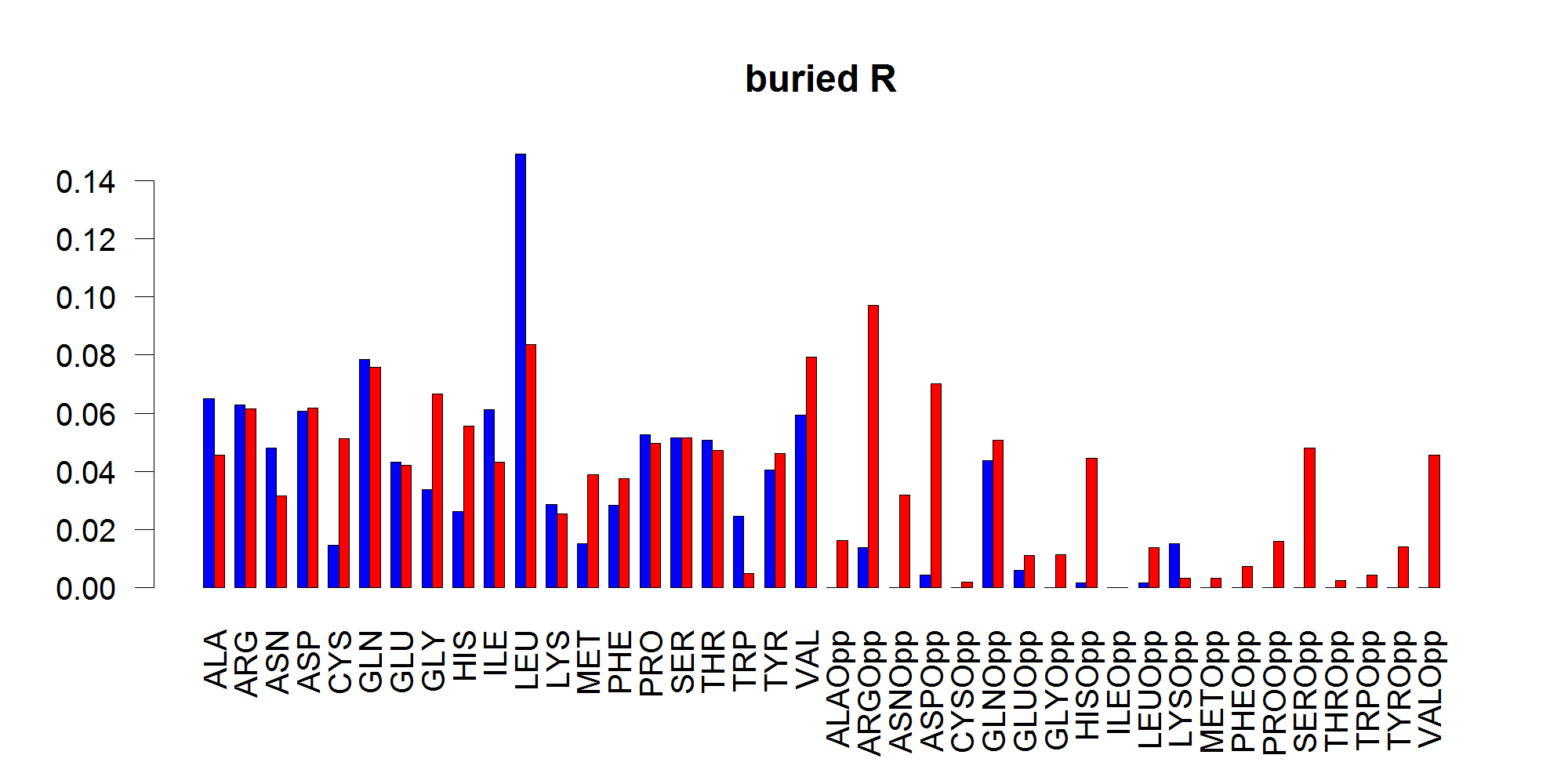

Supplement: Dataset S2 — Neighbouring residue profiles for mutations classed by WT residue. (ZIP) [file pone.0084598.s002.zip › neighbour_1/buried_R.tif]

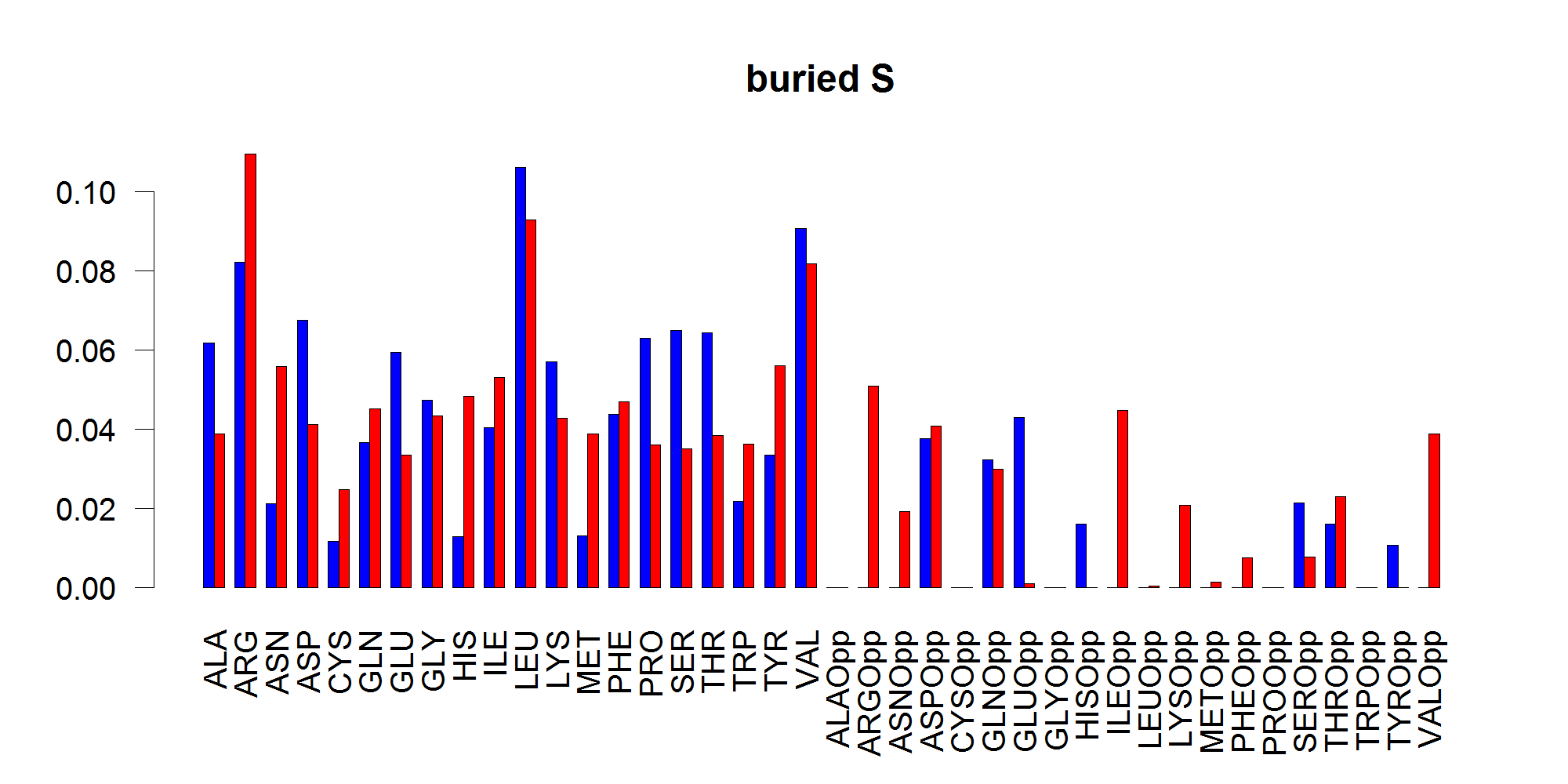

Supplement: Dataset S2 — Neighbouring residue profiles for mutations classed by WT residue. (ZIP) [file pone.0084598.s002.zip › neighbour_1/buried_S.tif]

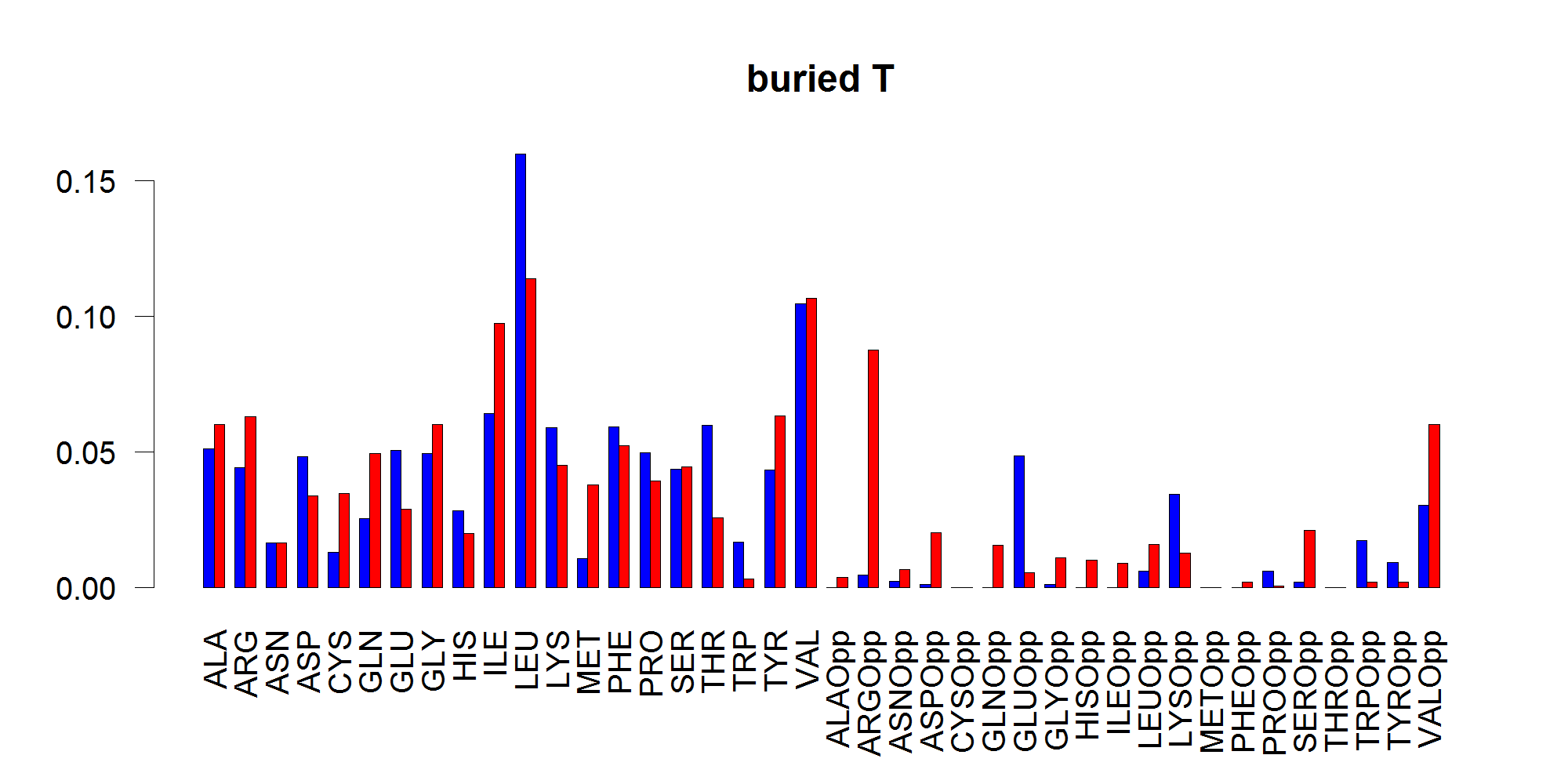

Supplement: Dataset S2 — Neighbouring residue profiles for mutations classed by WT residue. (ZIP) [file pone.0084598.s002.zip › neighbour_1/buried_T.tif]

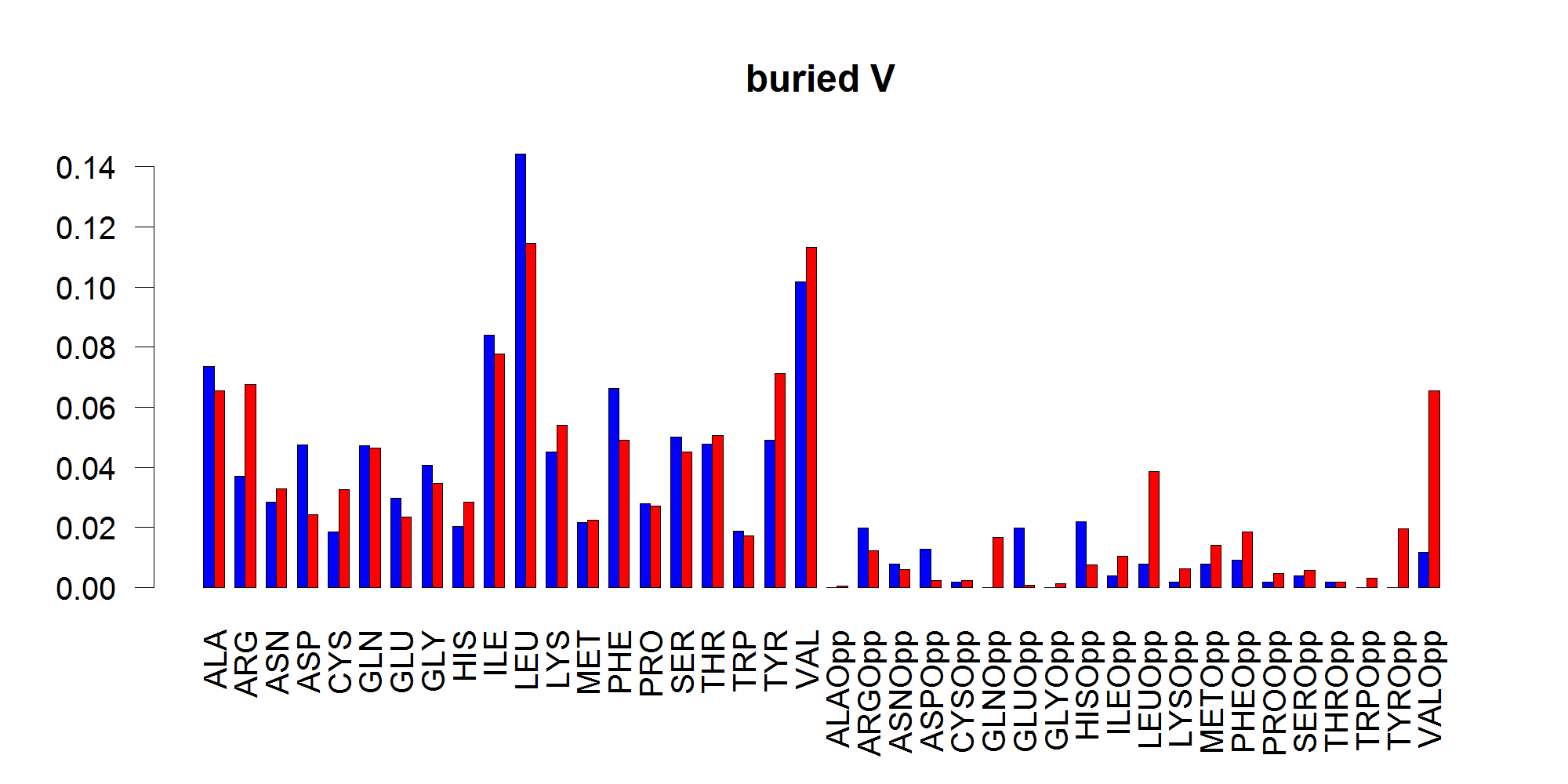

Supplement: Dataset S2 — Neighbouring residue profiles for mutations classed by WT residue. (ZIP) [file pone.0084598.s002.zip › neighbour_1/buried_V.tif]

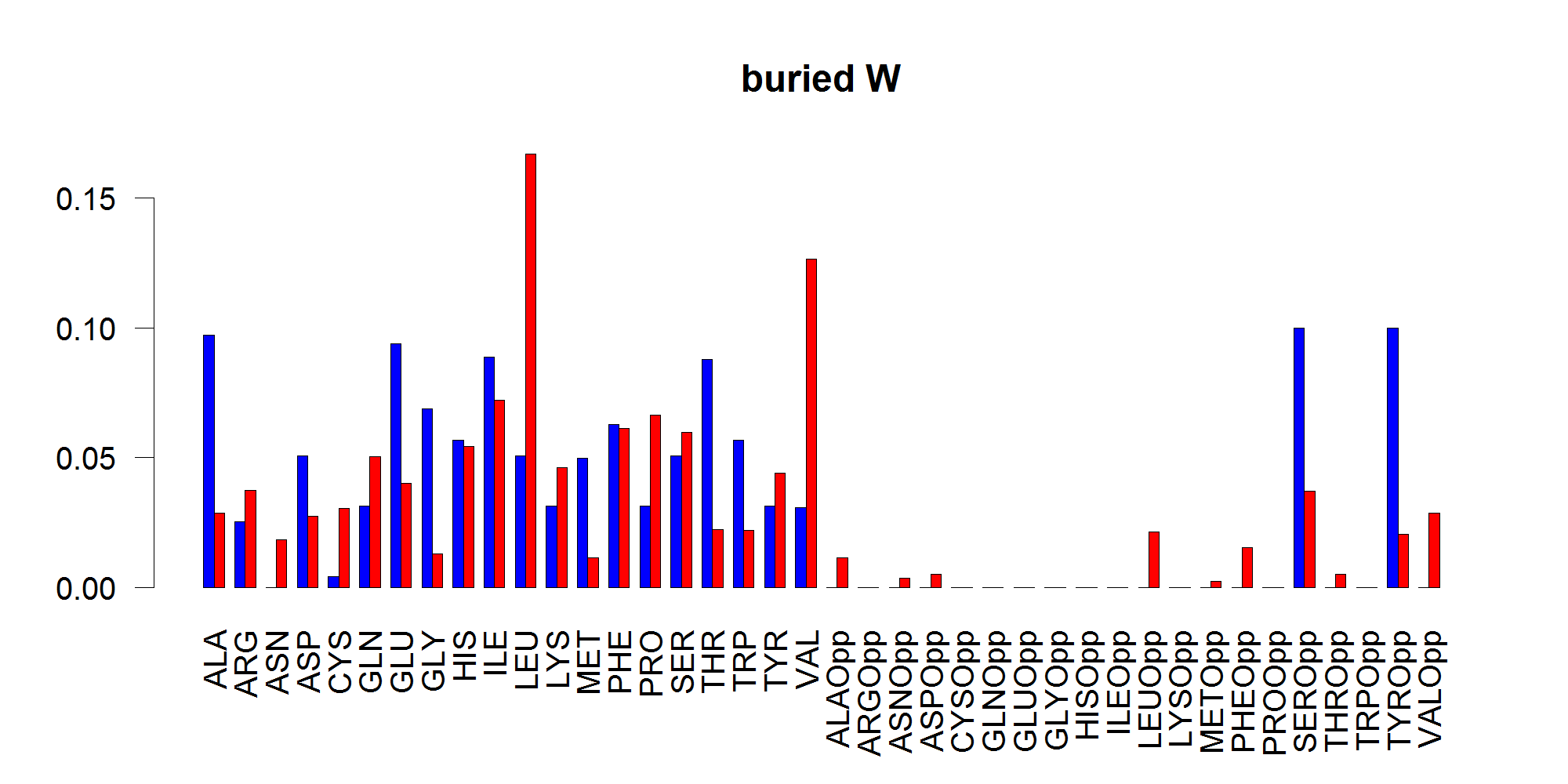

Supplement: Dataset S2 — Neighbouring residue profiles for mutations classed by WT residue. (ZIP) [file pone.0084598.s002.zip › neighbour_1/buried_W.tif]

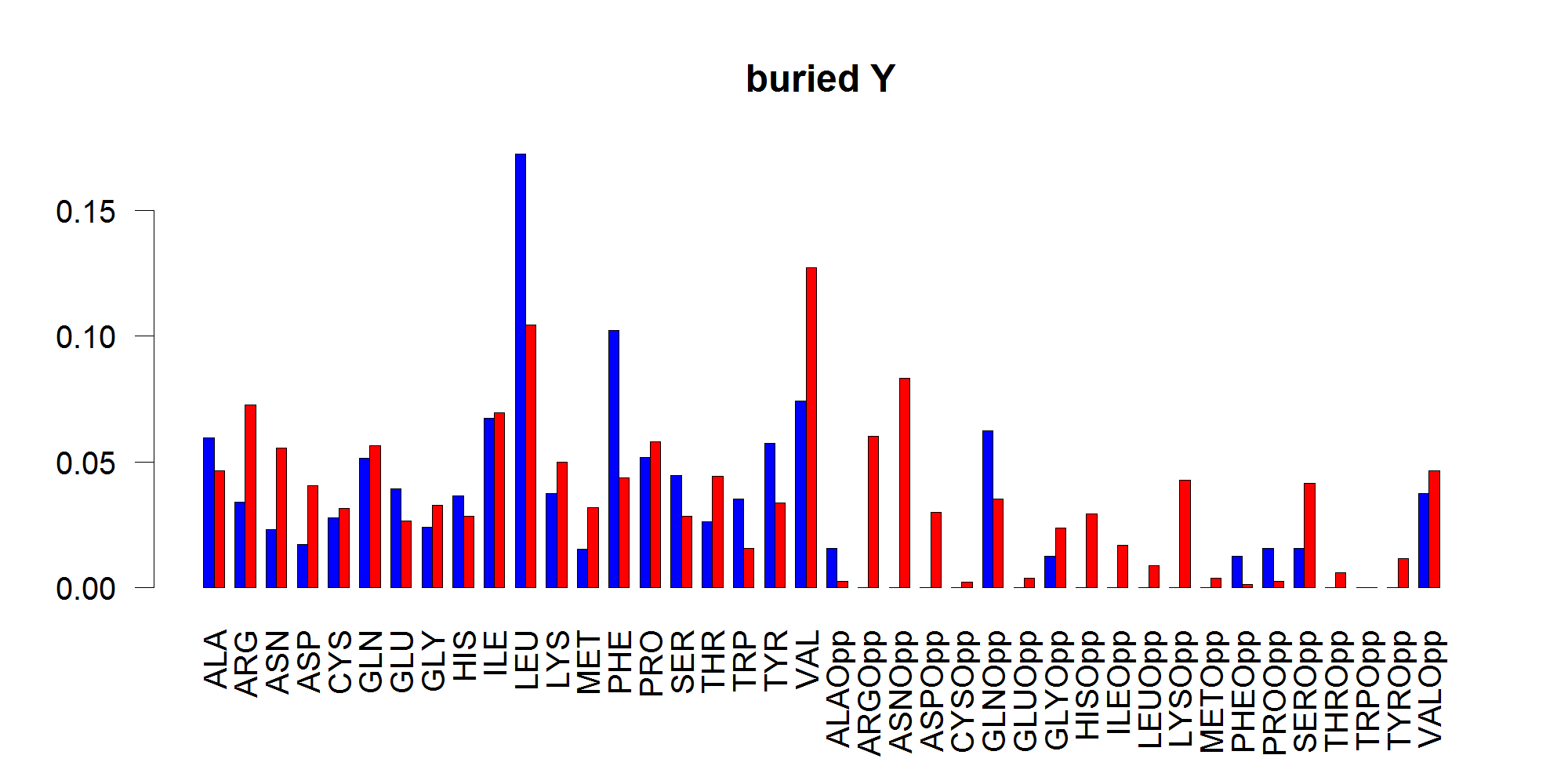

Supplement: Dataset S2 — Neighbouring residue profiles for mutations classed by WT residue. (ZIP) [file pone.0084598.s002.zip › neighbour_1/buried_Y.tif]

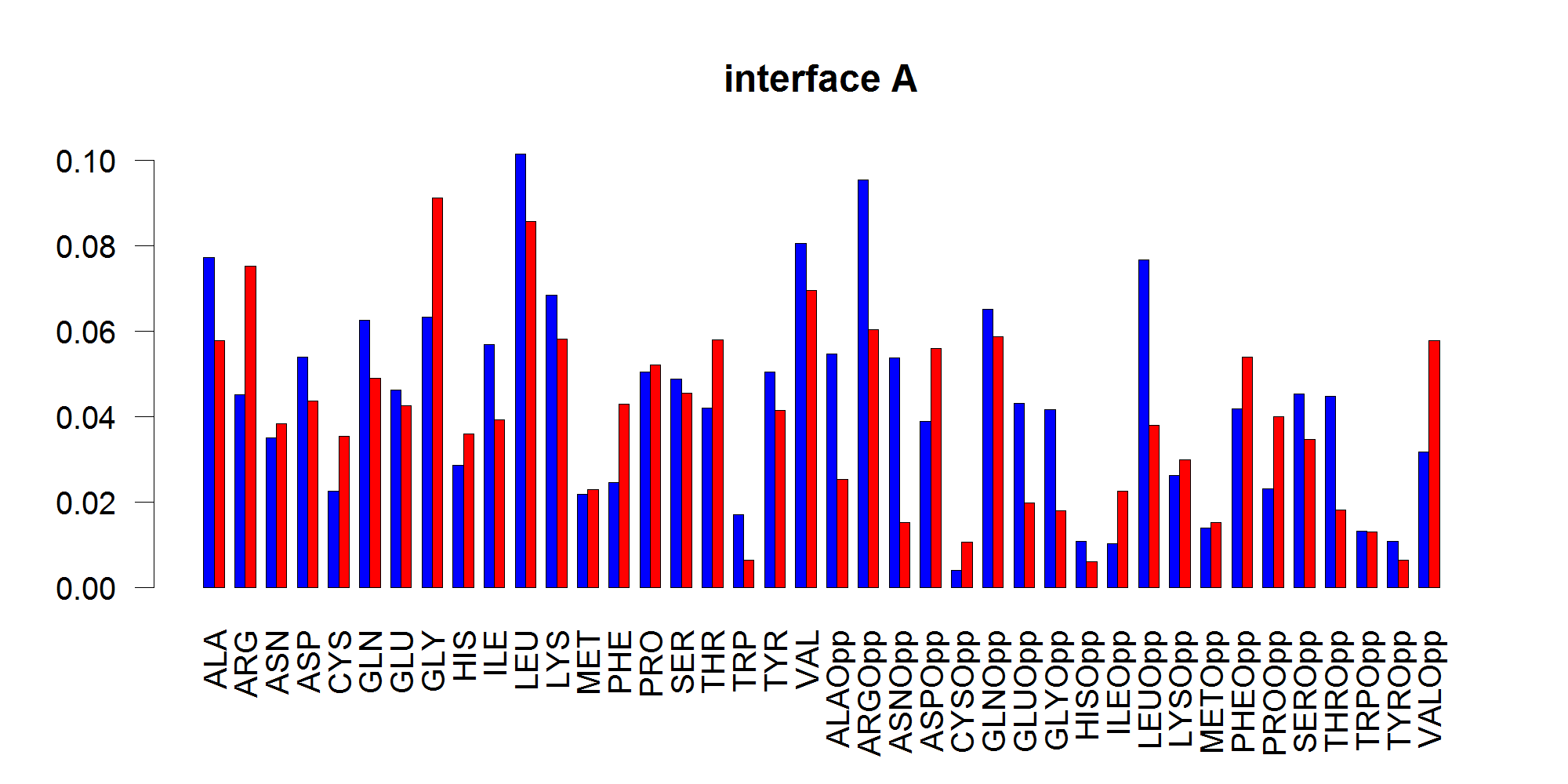

Supplement: Dataset S2 — Neighbouring residue profiles for mutations classed by WT residue. (ZIP) [file pone.0084598.s002.zip › neighbour_1/interface_A.tif]

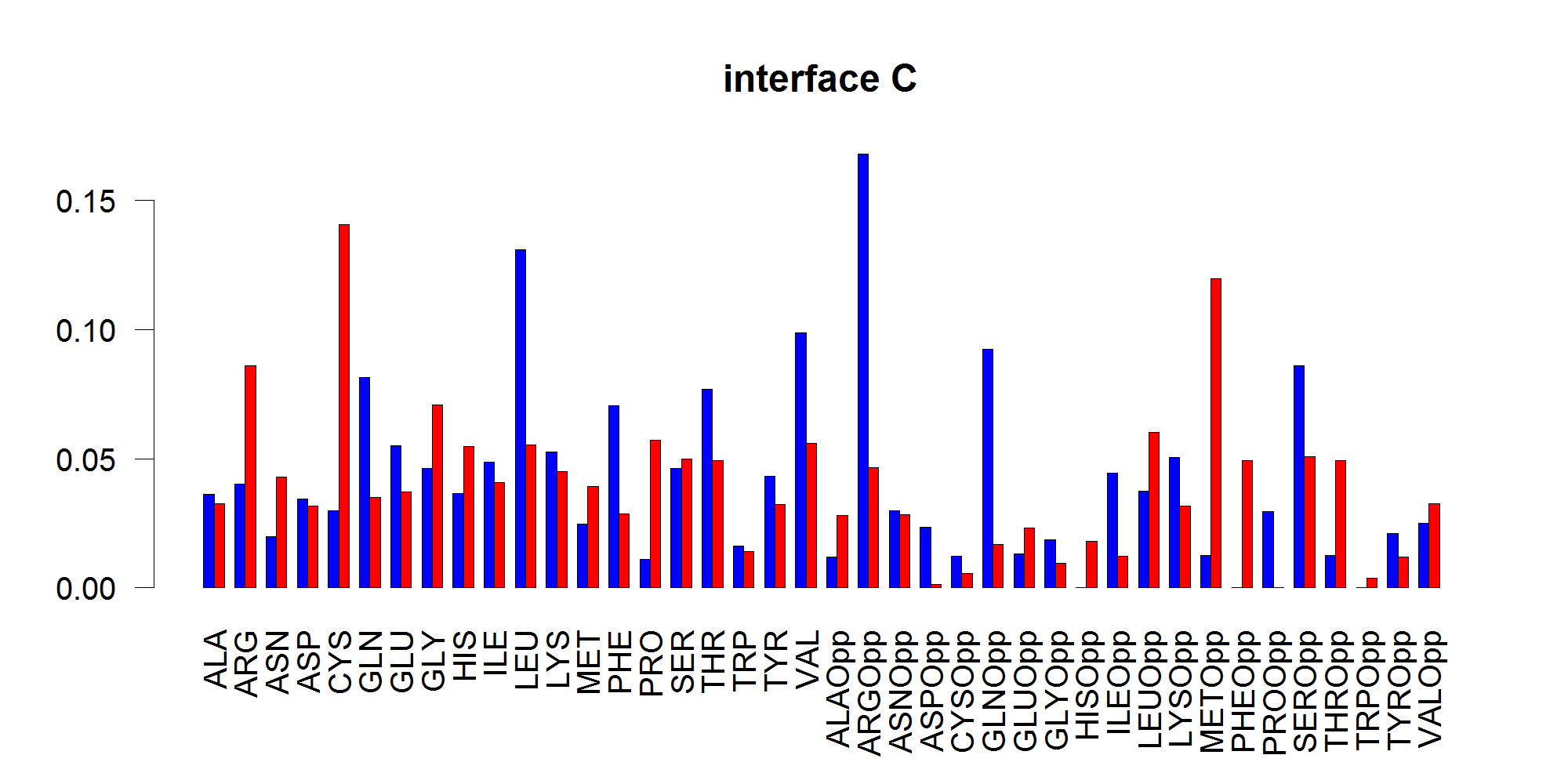

Supplement: Dataset S2 — Neighbouring residue profiles for mutations classed by WT residue. (ZIP) [file pone.0084598.s002.zip › neighbour_1/interface_C.tif]

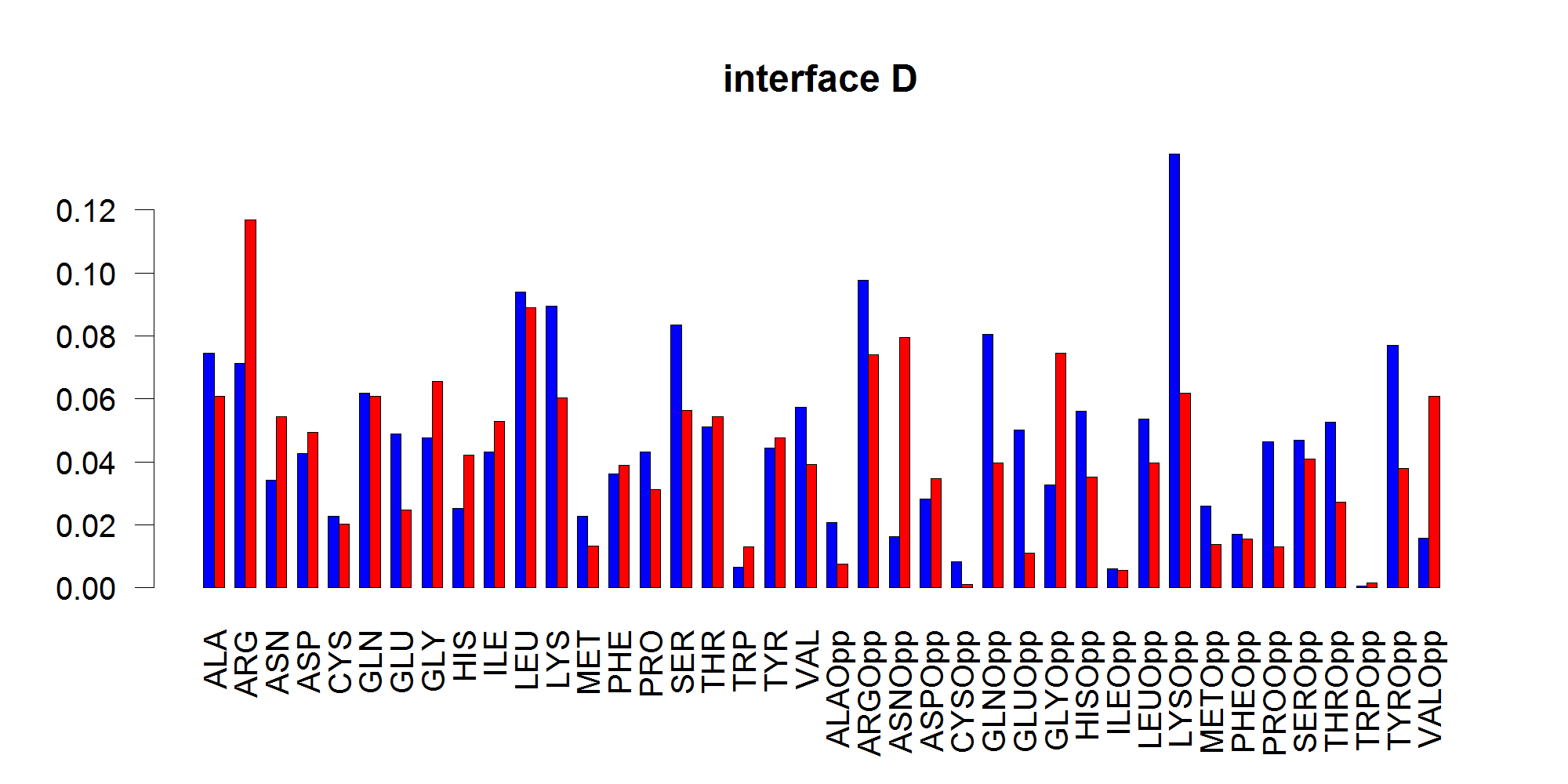

Supplement: Dataset S2 — Neighbouring residue profiles for mutations classed by WT residue. (ZIP) [file pone.0084598.s002.zip › neighbour_1/interface_D.tif]

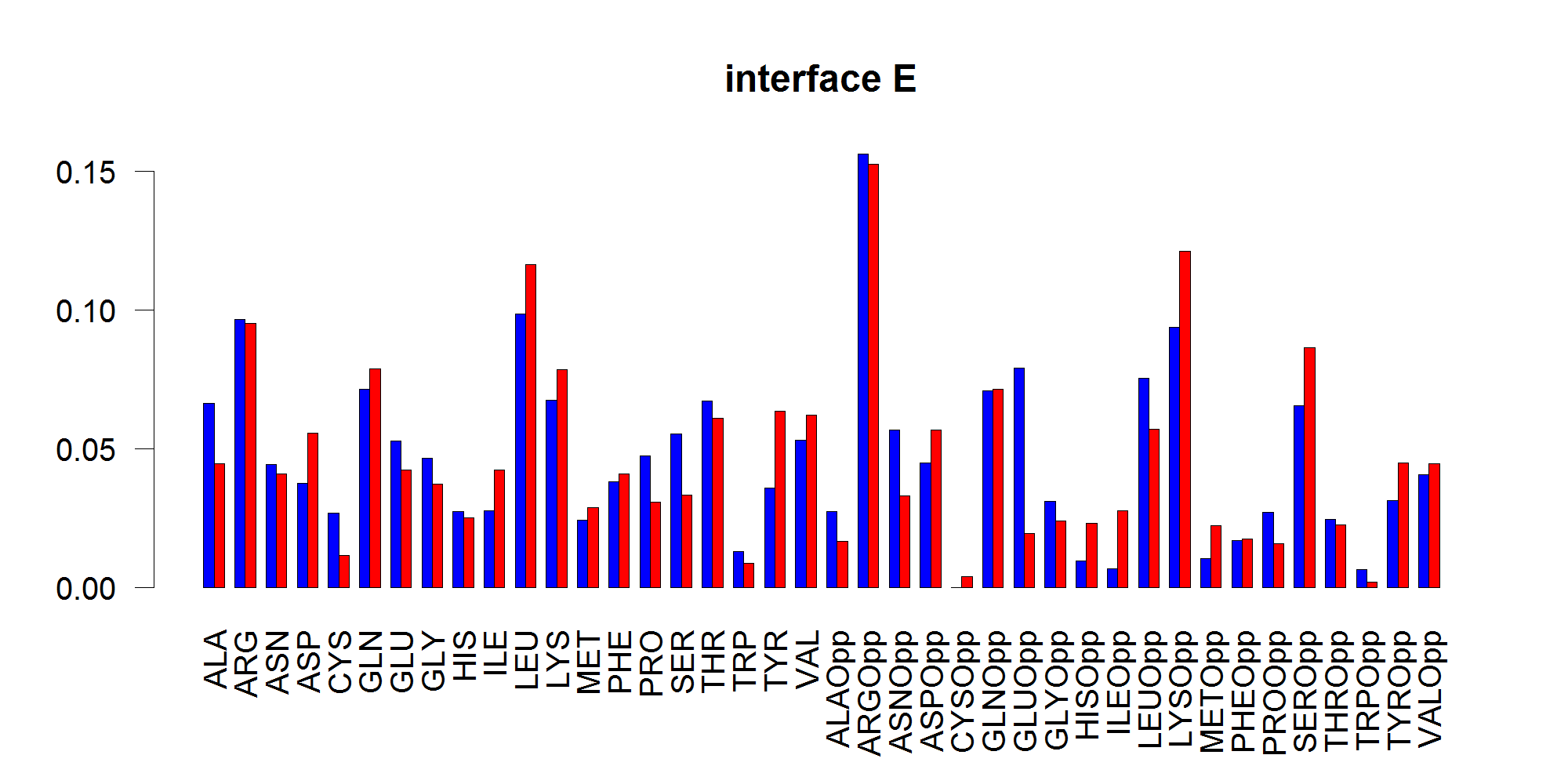

Supplement: Dataset S2 — Neighbouring residue profiles for mutations classed by WT residue. (ZIP) [file pone.0084598.s002.zip › neighbour_1/interface_E.tif]

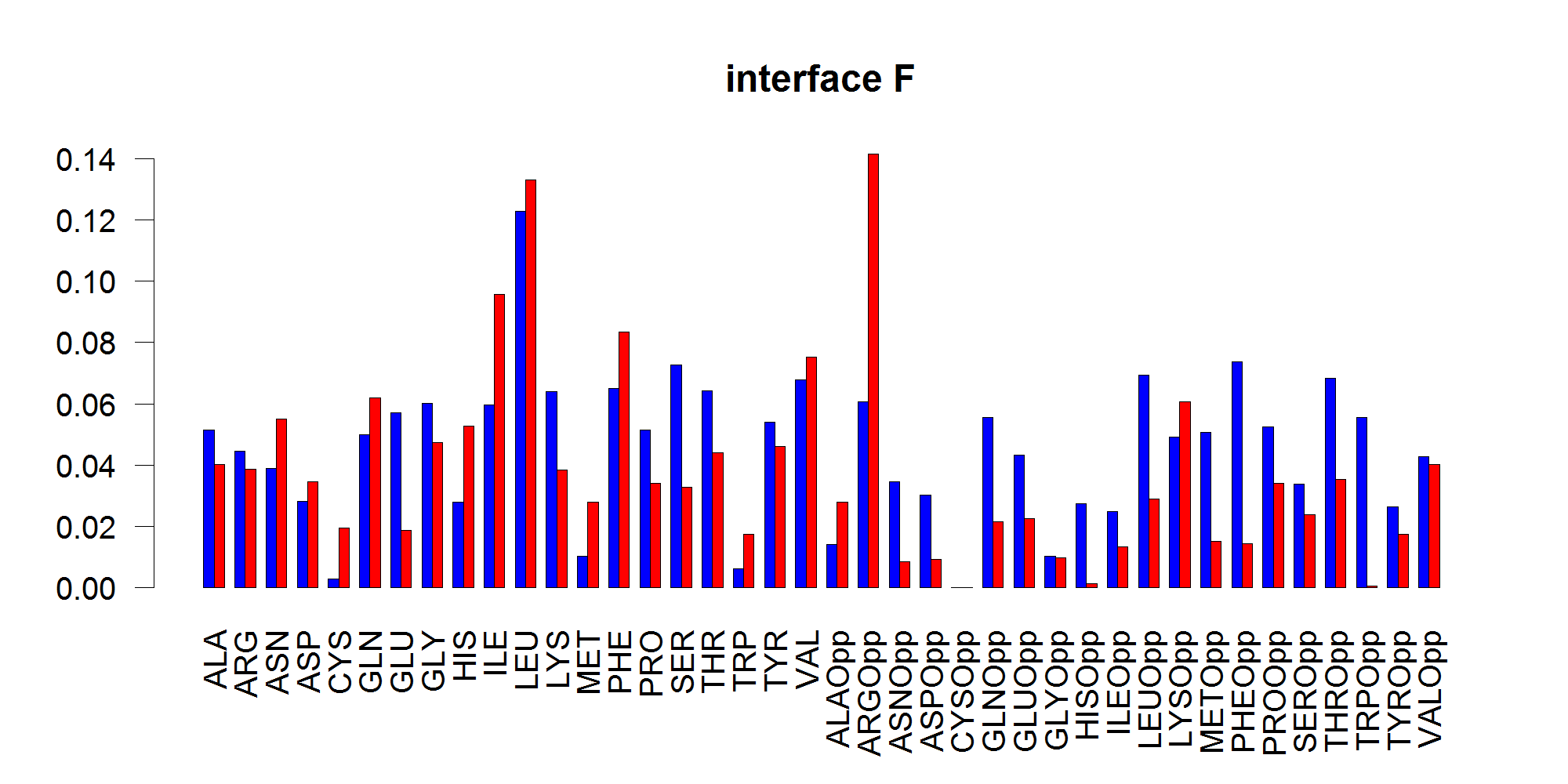

Supplement: Dataset S2 — Neighbouring residue profiles for mutations classed by WT residue. (ZIP) [file pone.0084598.s002.zip › neighbour_1/interface_F.tif]

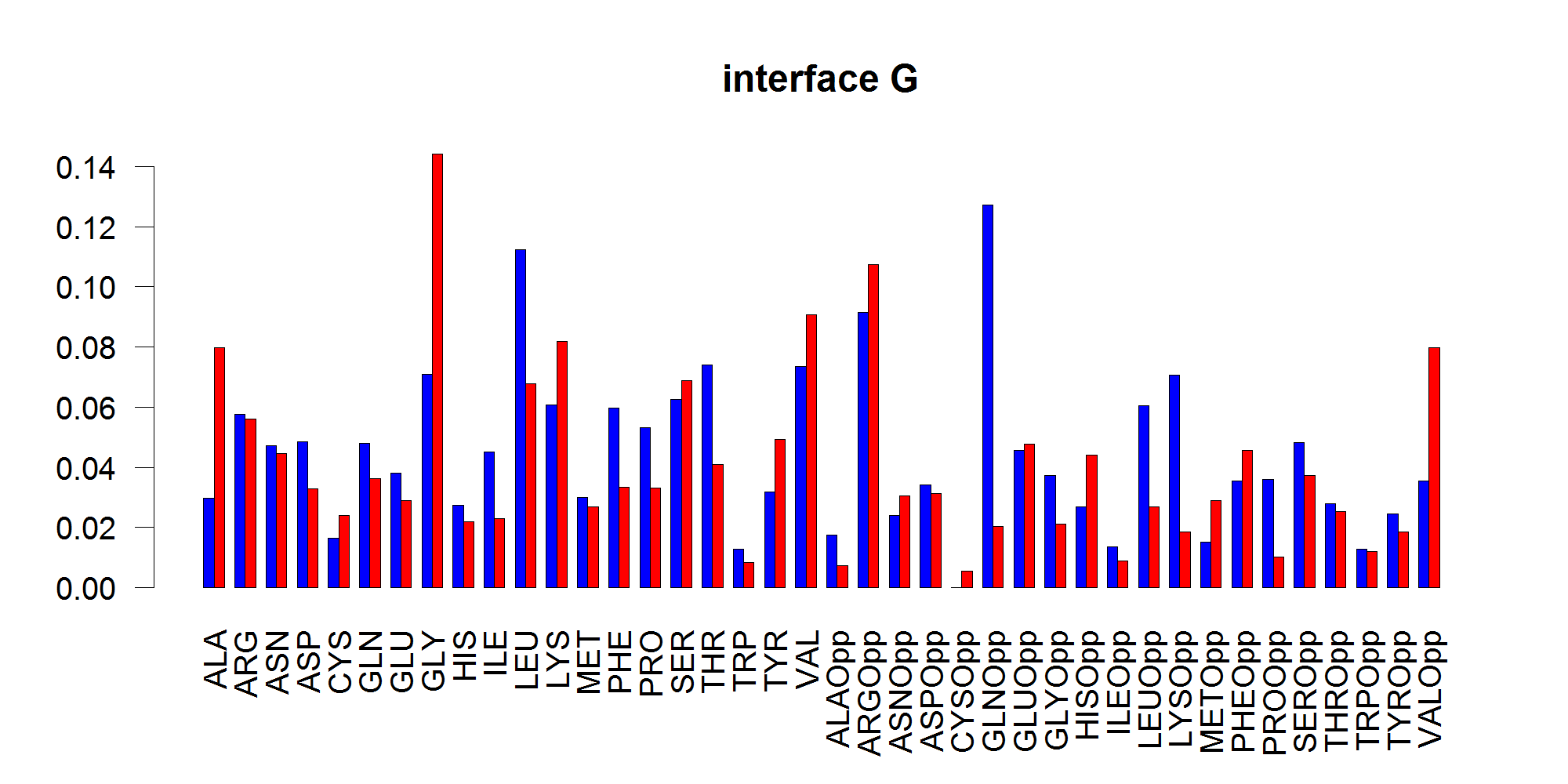

Supplement: Dataset S2 — Neighbouring residue profiles for mutations classed by WT residue. (ZIP) [file pone.0084598.s002.zip › neighbour_1/interface_G.tif]

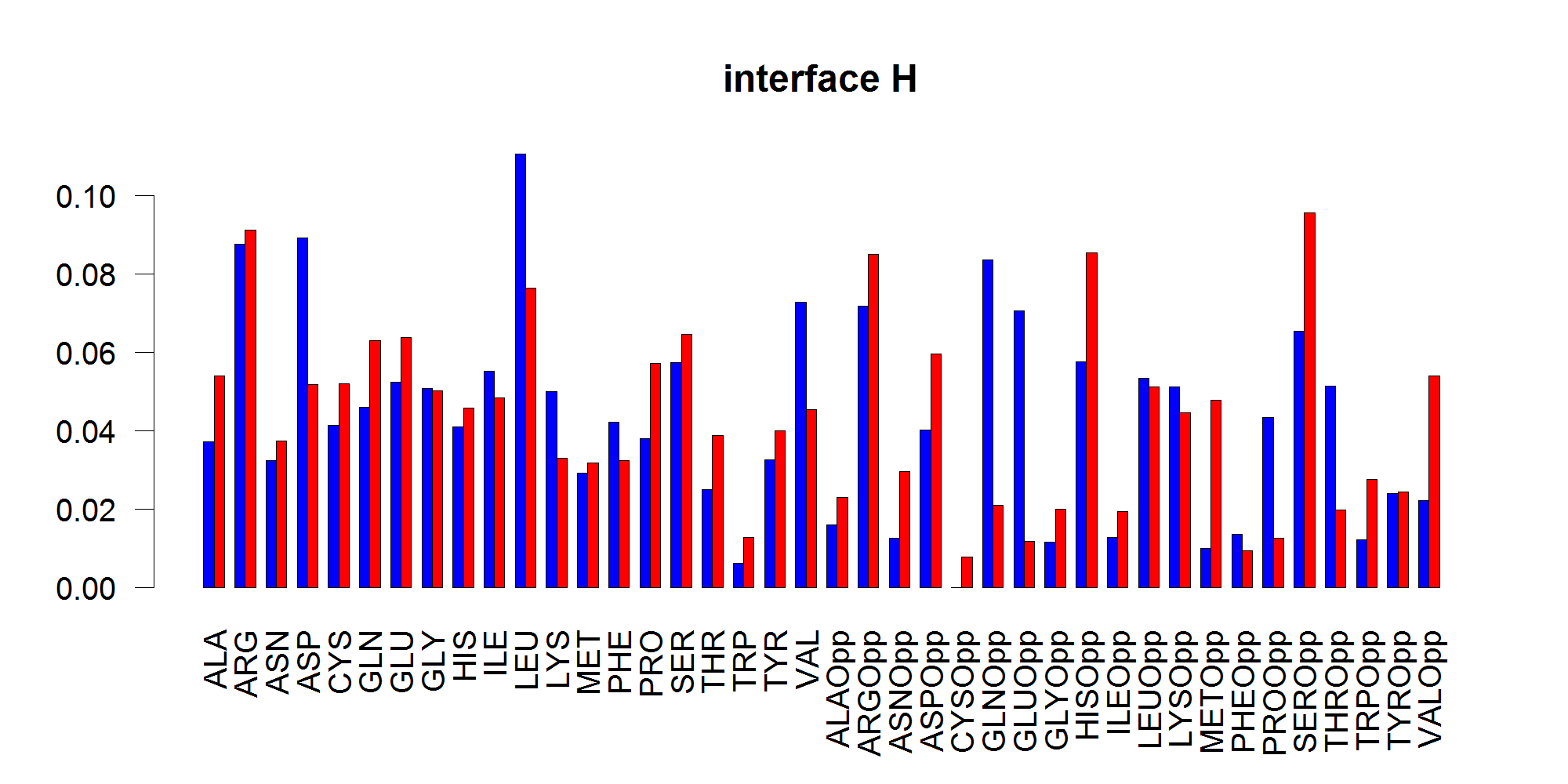

Supplement: Dataset S2 — Neighbouring residue profiles for mutations classed by WT residue. (ZIP) [file pone.0084598.s002.zip › neighbour_1/interface_H.tif]

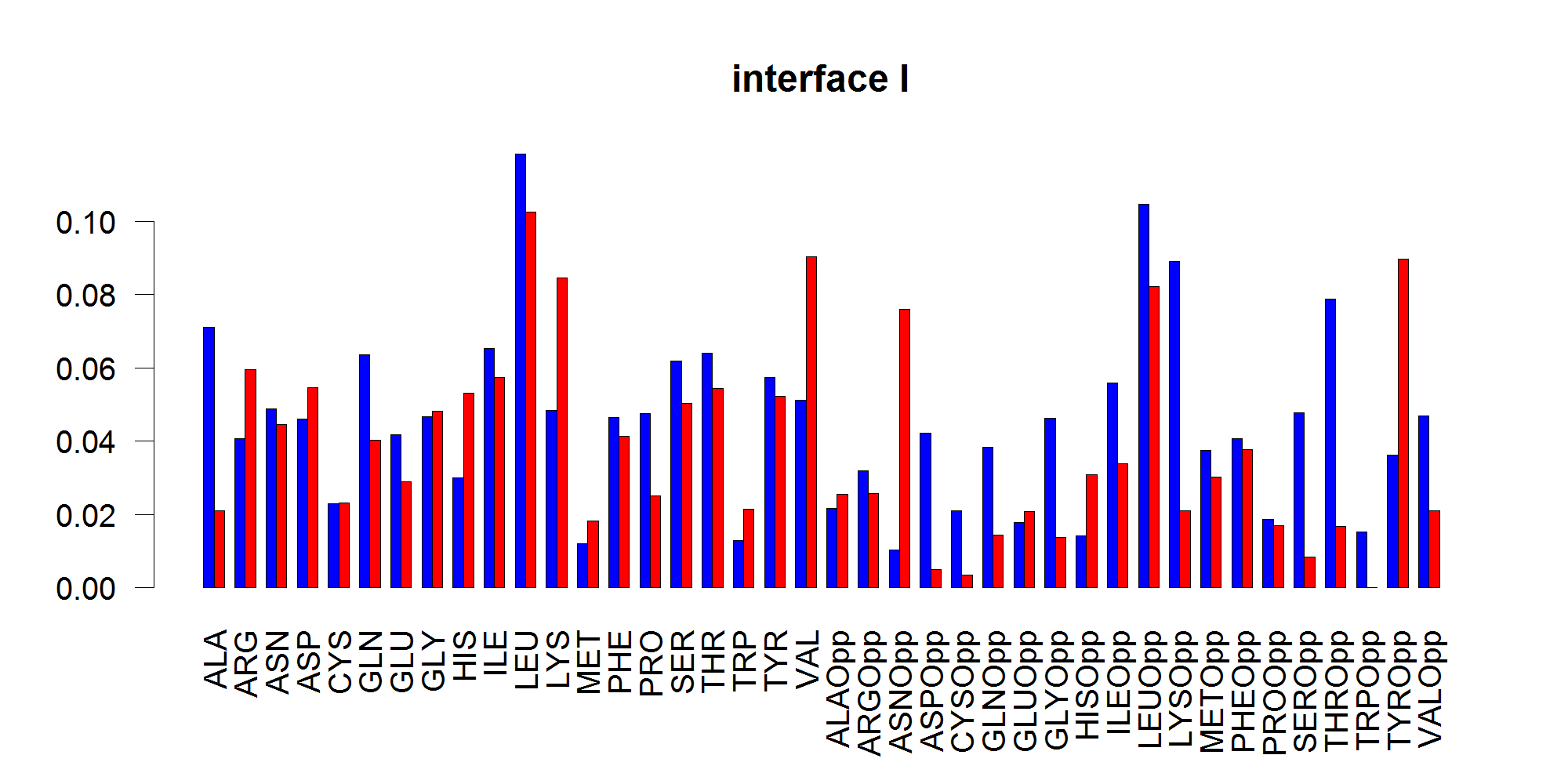

Supplement: Dataset S2 — Neighbouring residue profiles for mutations classed by WT residue. (ZIP) [file pone.0084598.s002.zip › neighbour_1/interface_I.tif]

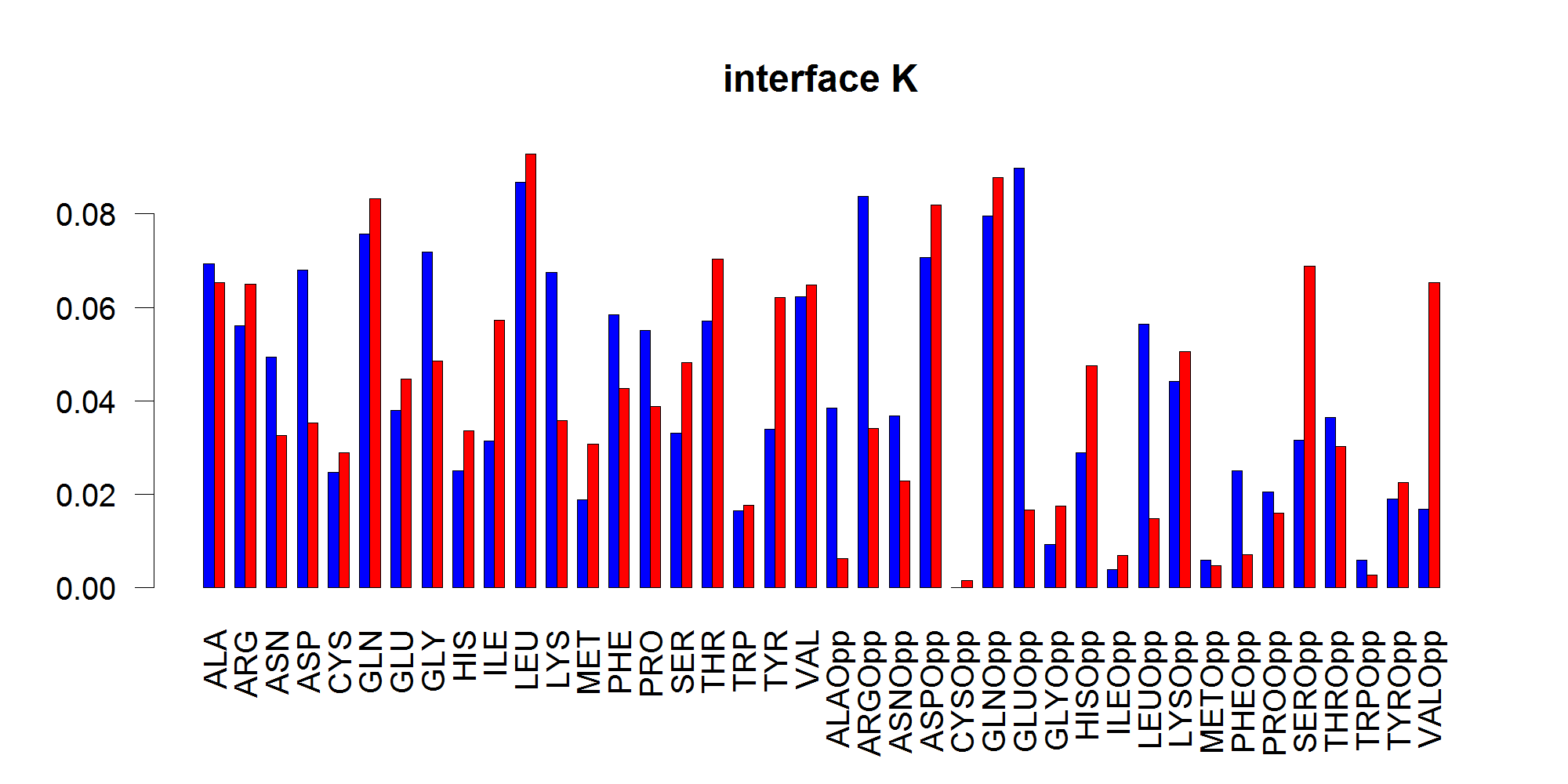

Supplement: Dataset S2 — Neighbouring residue profiles for mutations classed by WT residue. (ZIP) [file pone.0084598.s002.zip › neighbour_1/interface_K.tif]

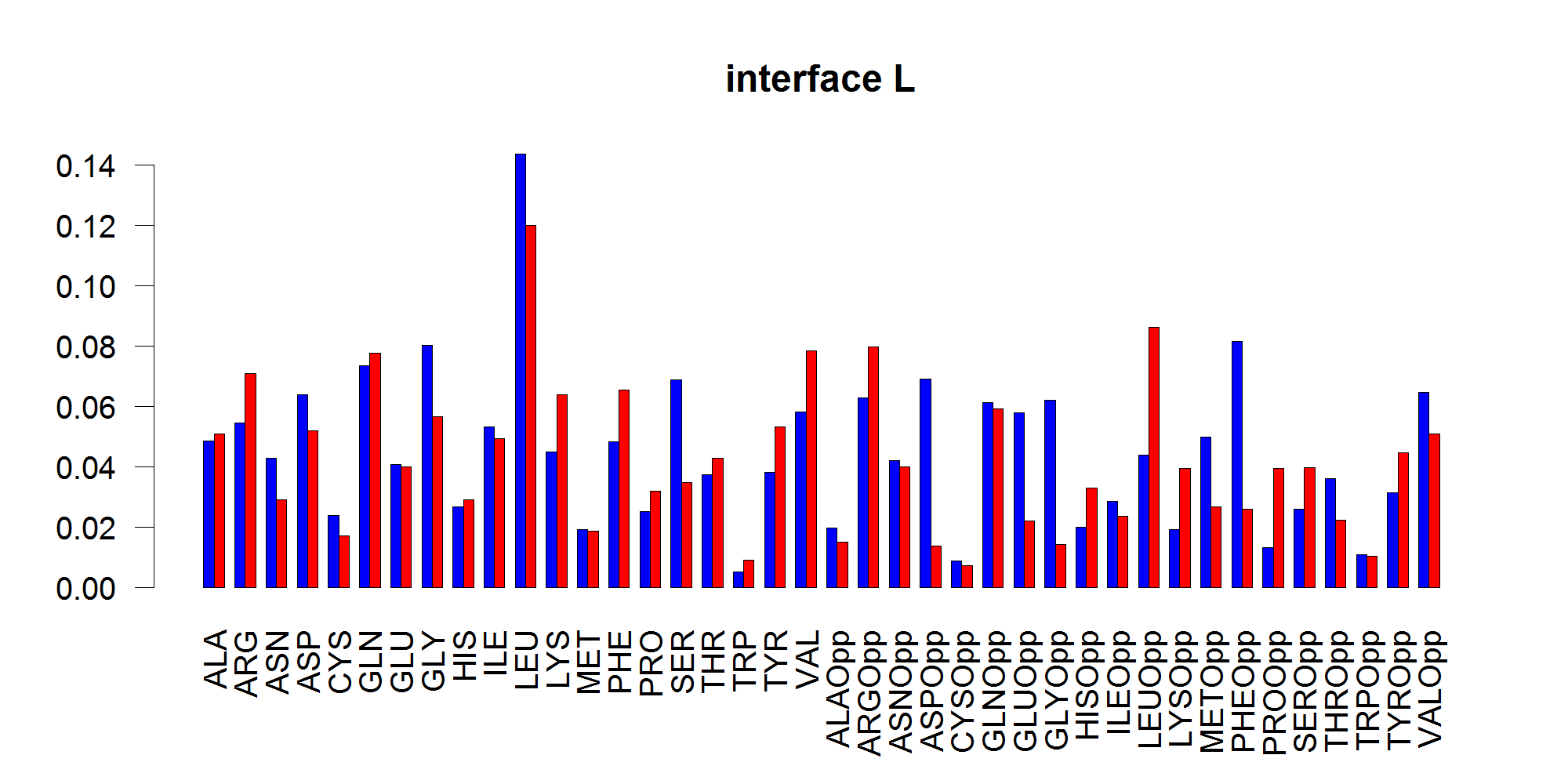

Supplement: Dataset S2 — Neighbouring residue profiles for mutations classed by WT residue. (ZIP) [file pone.0084598.s002.zip › neighbour_1/interface_L.tif]

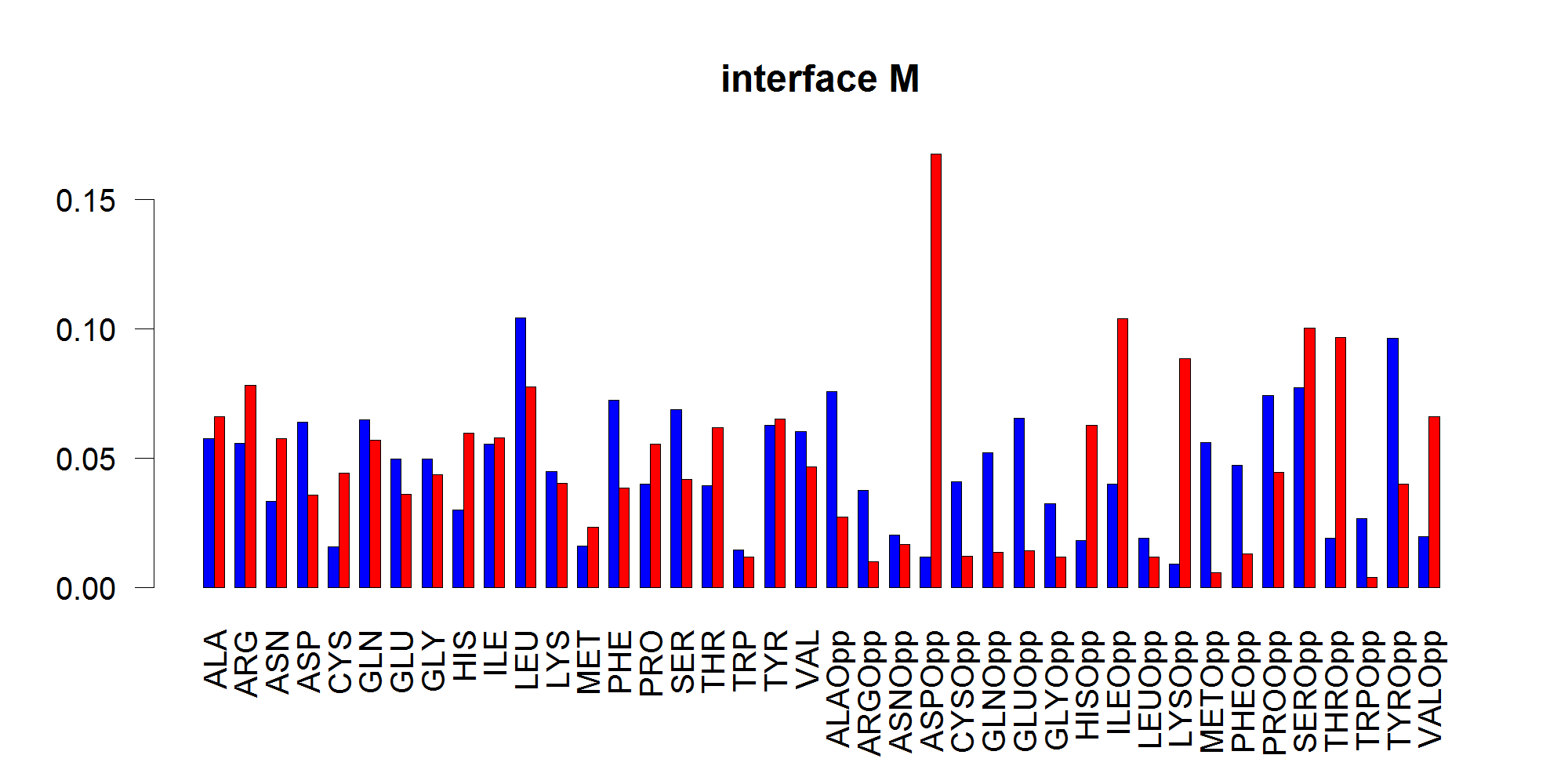

Supplement: Dataset S2 — Neighbouring residue profiles for mutations classed by WT residue. (ZIP) [file pone.0084598.s002.zip › neighbour_1/interface_M.tif]

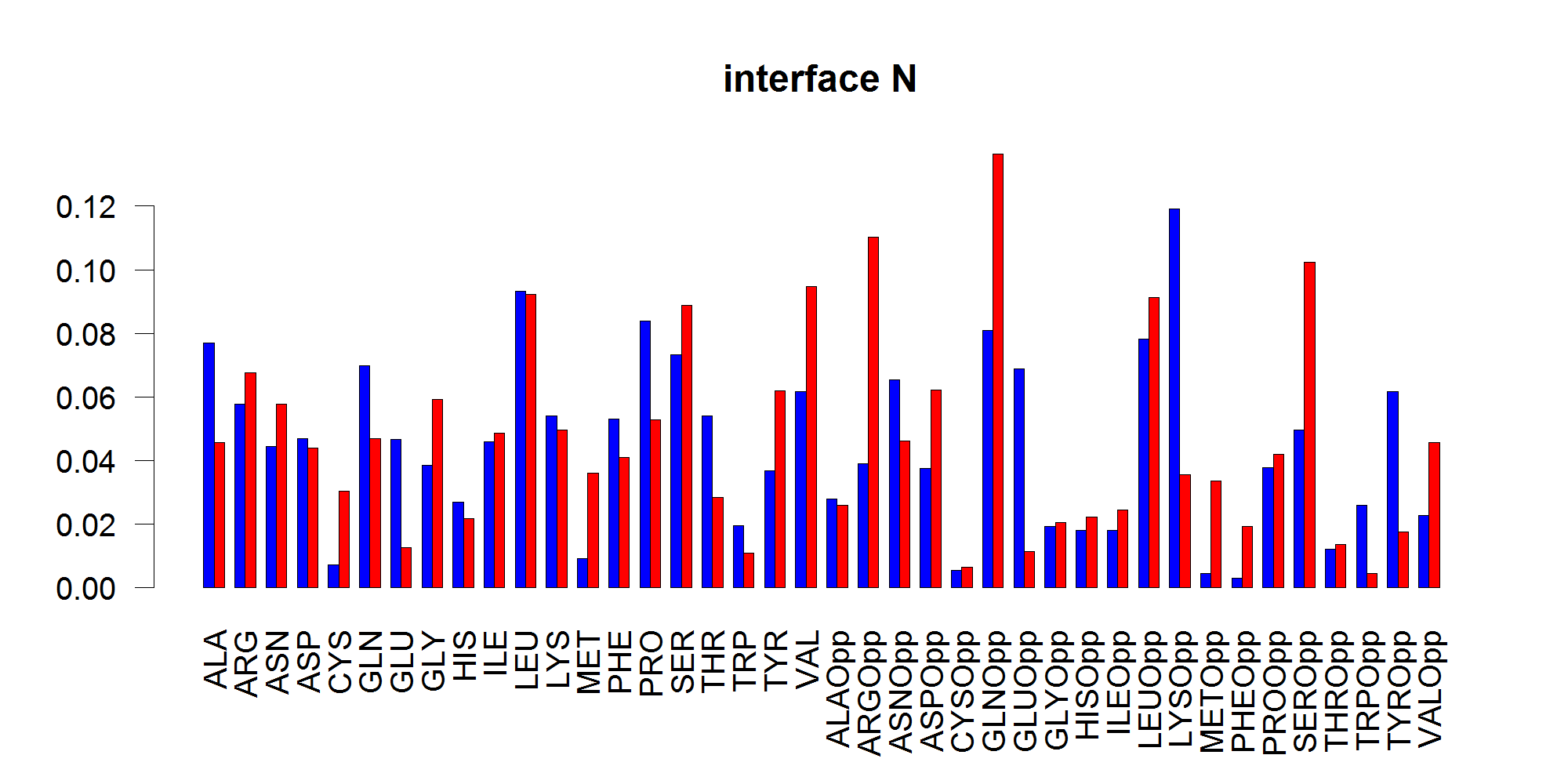

Supplement: Dataset S2 — Neighbouring residue profiles for mutations classed by WT residue. (ZIP) [file pone.0084598.s002.zip › neighbour_1/interface_N.tif]

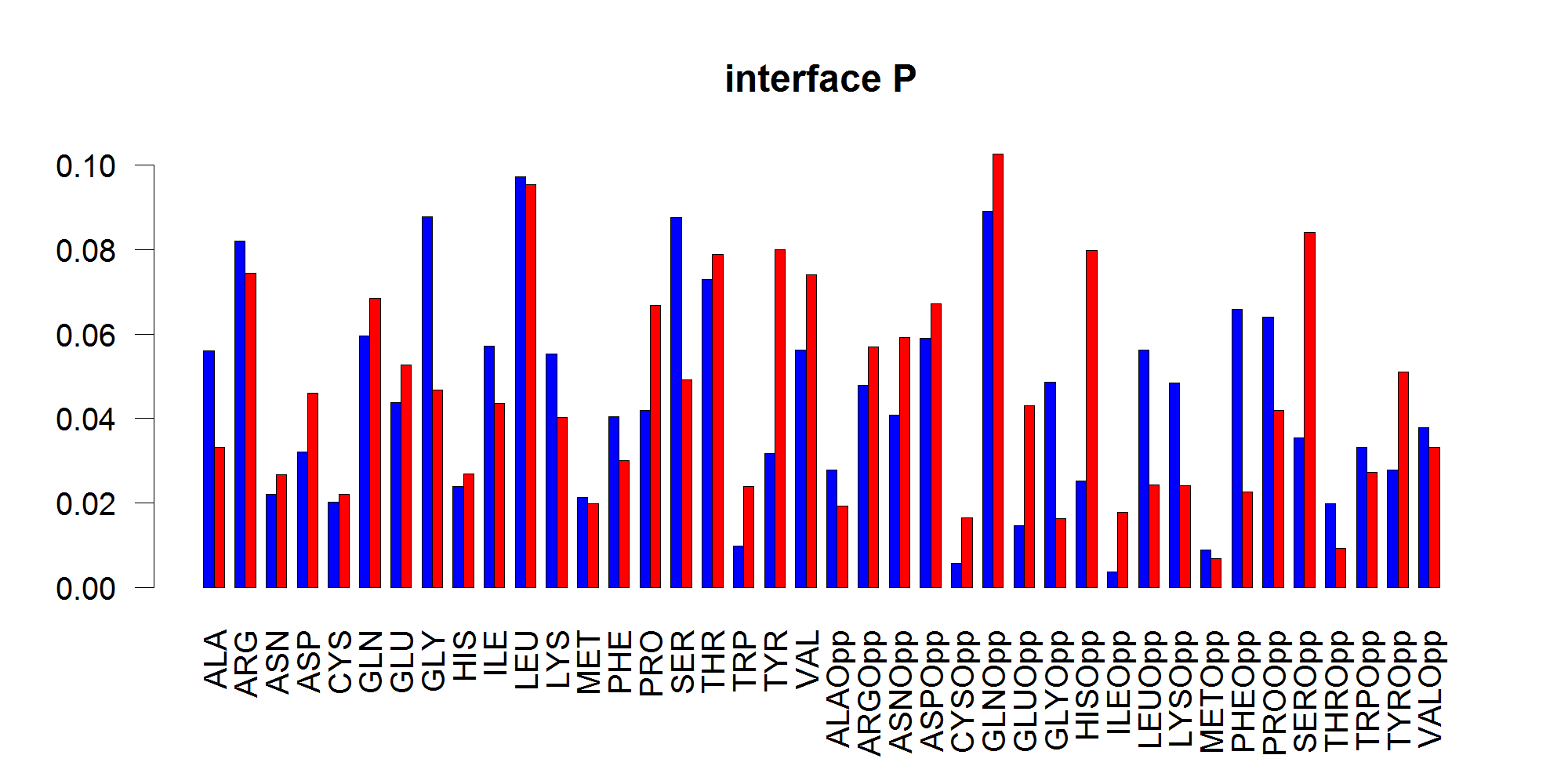

Supplement: Dataset S2 — Neighbouring residue profiles for mutations classed by WT residue. (ZIP) [file pone.0084598.s002.zip › neighbour_1/interface_P.tif]

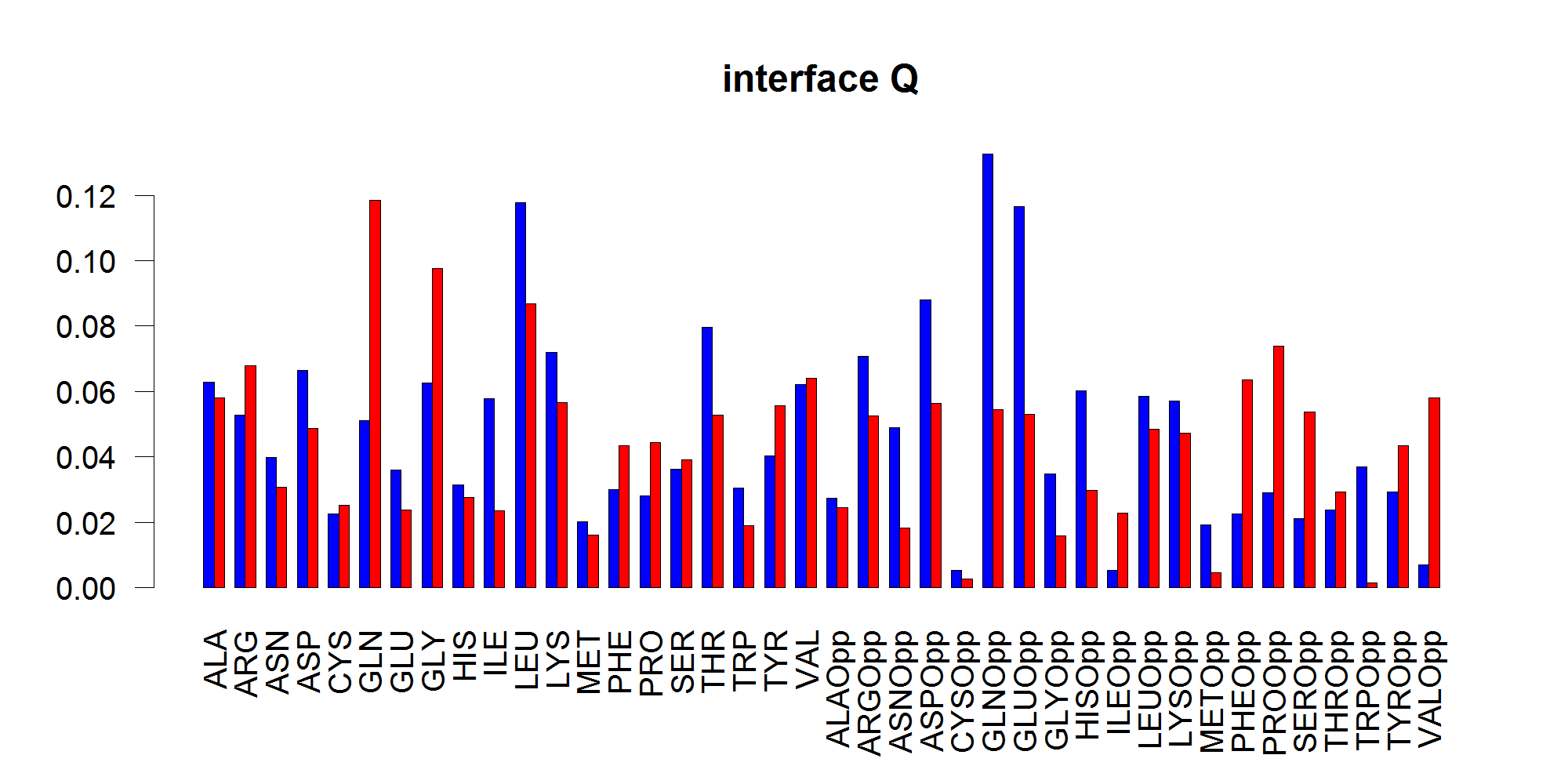

Supplement: Dataset S2 — Neighbouring residue profiles for mutations classed by WT residue. (ZIP) [file pone.0084598.s002.zip › neighbour_1/interface_Q.tif]

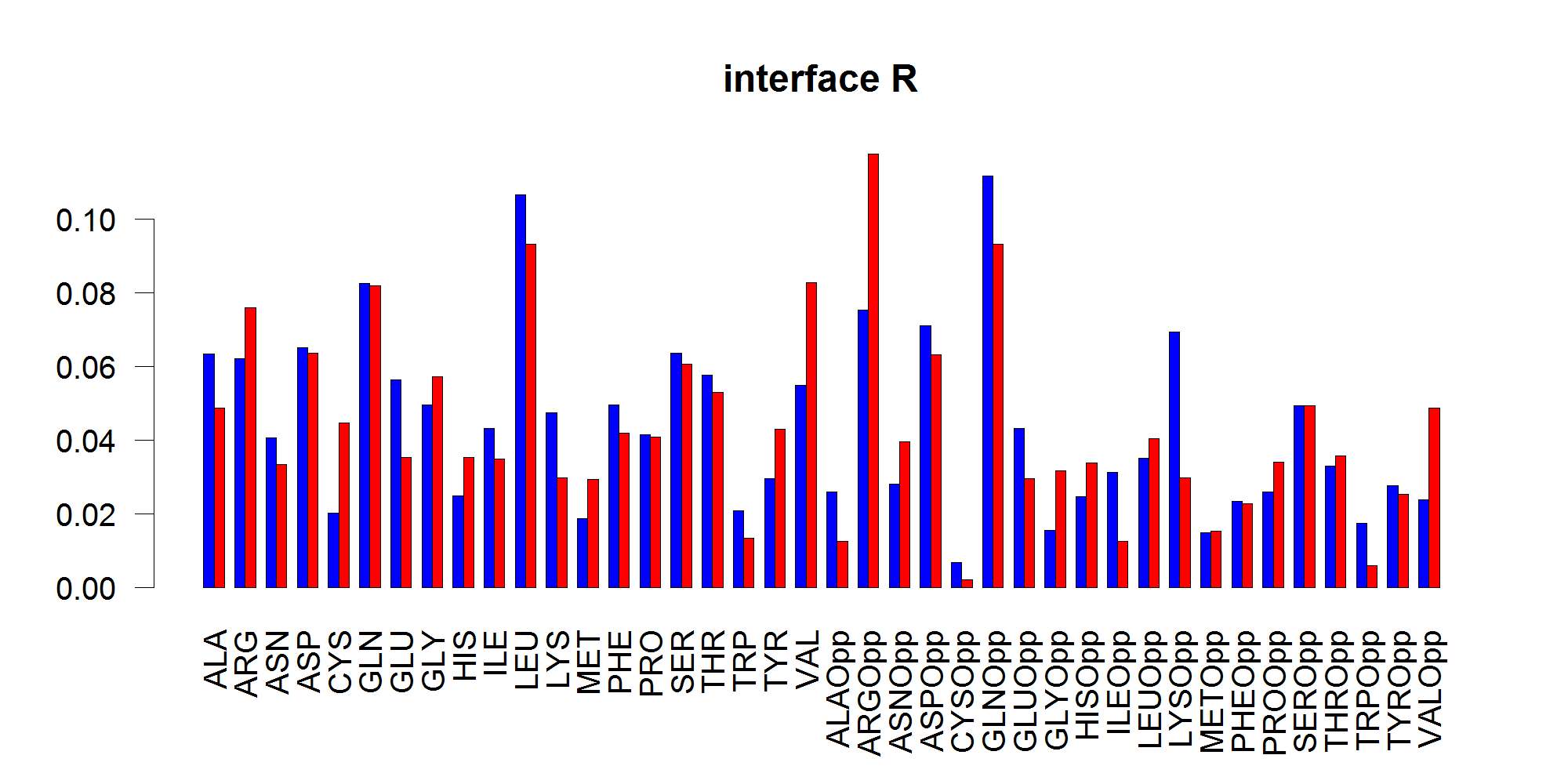

Supplement: Dataset S2 — Neighbouring residue profiles for mutations classed by WT residue. (ZIP) [file pone.0084598.s002.zip › neighbour_1/interface_R.tif]

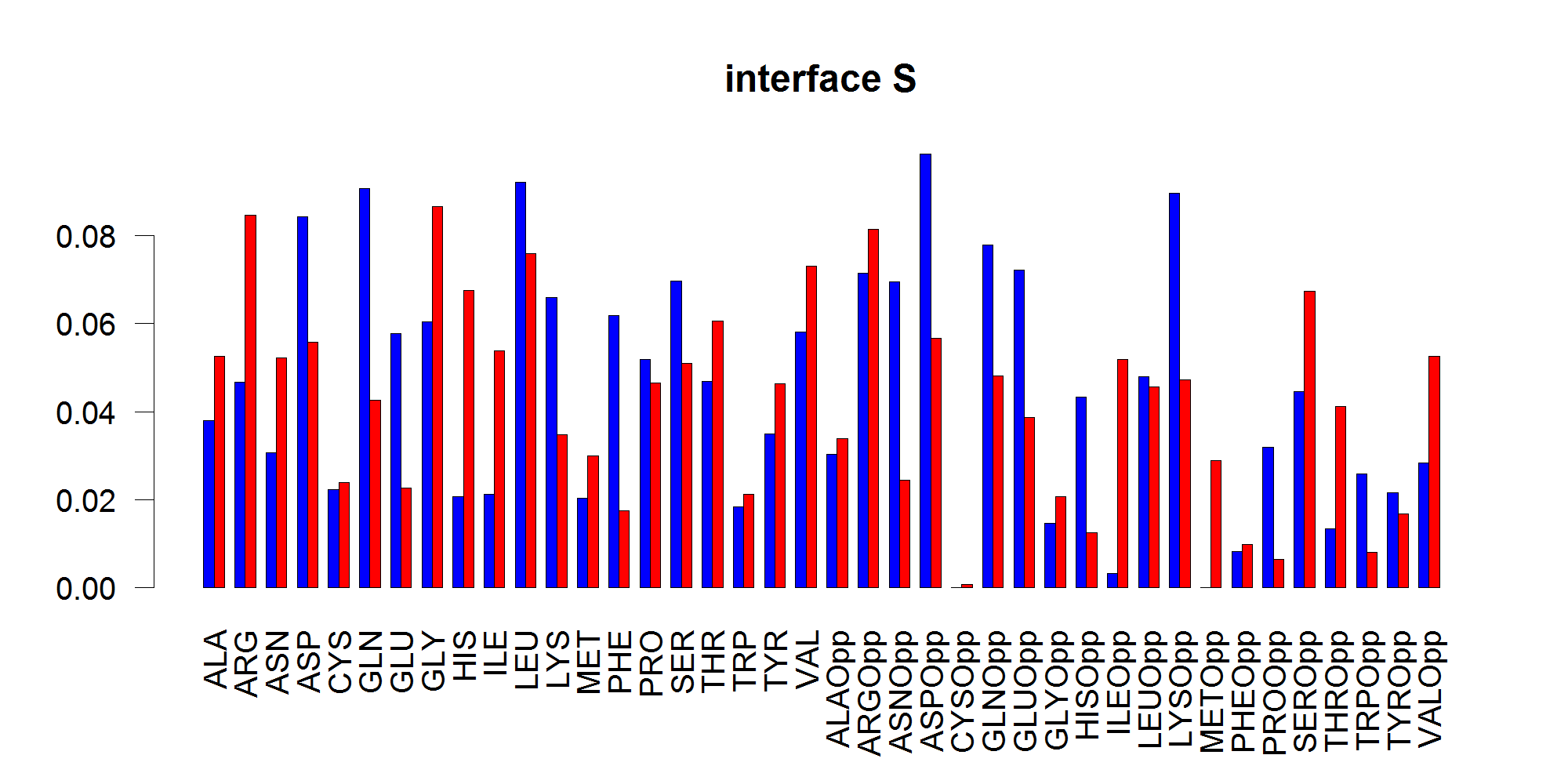

Supplement: Dataset S2 — Neighbouring residue profiles for mutations classed by WT residue. (ZIP) [file pone.0084598.s002.zip › neighbour_1/interface_S.tif]

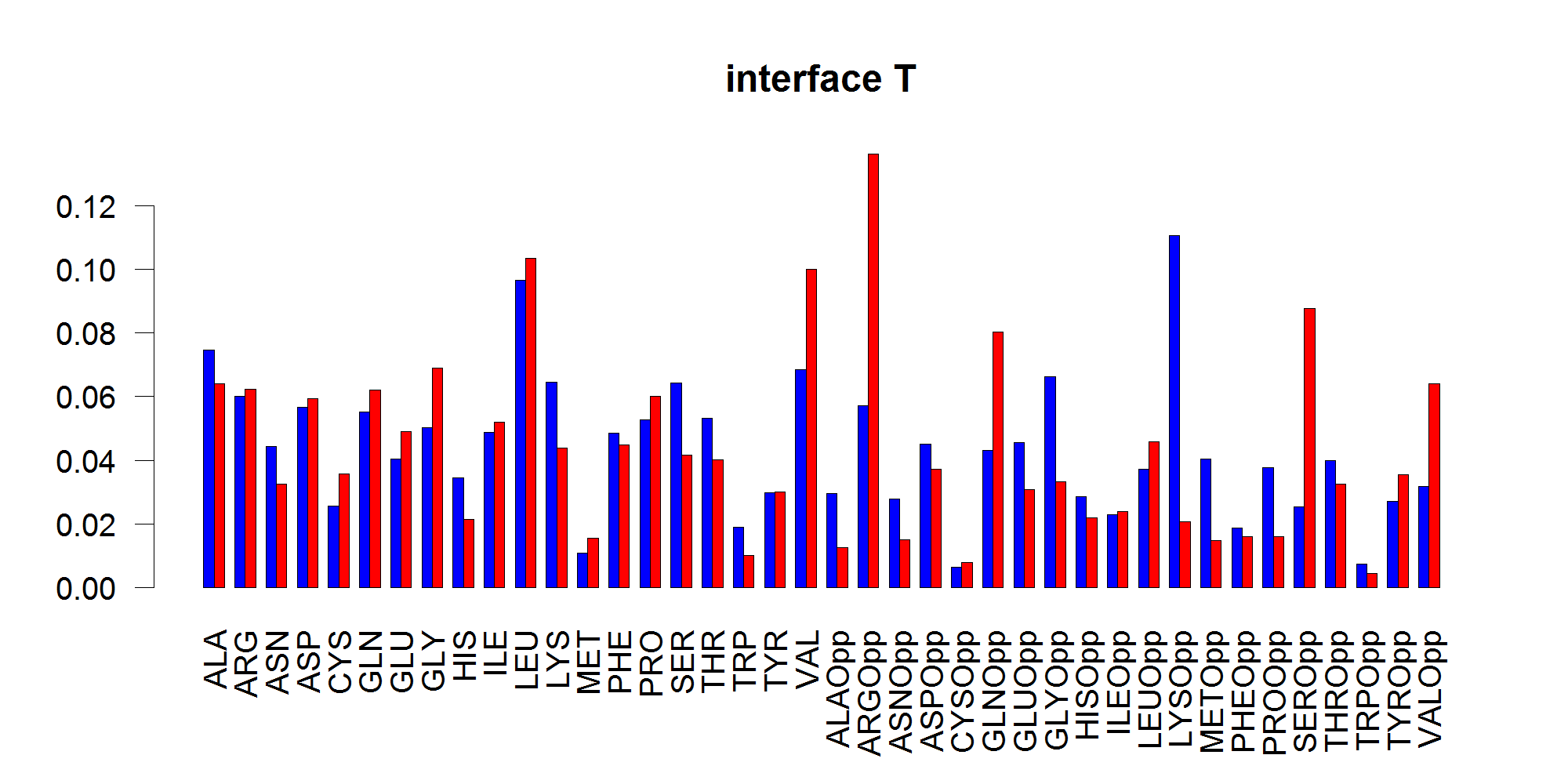

Supplement: Dataset S2 — Neighbouring residue profiles for mutations classed by WT residue. (ZIP) [file pone.0084598.s002.zip › neighbour_1/interface_T.tif]

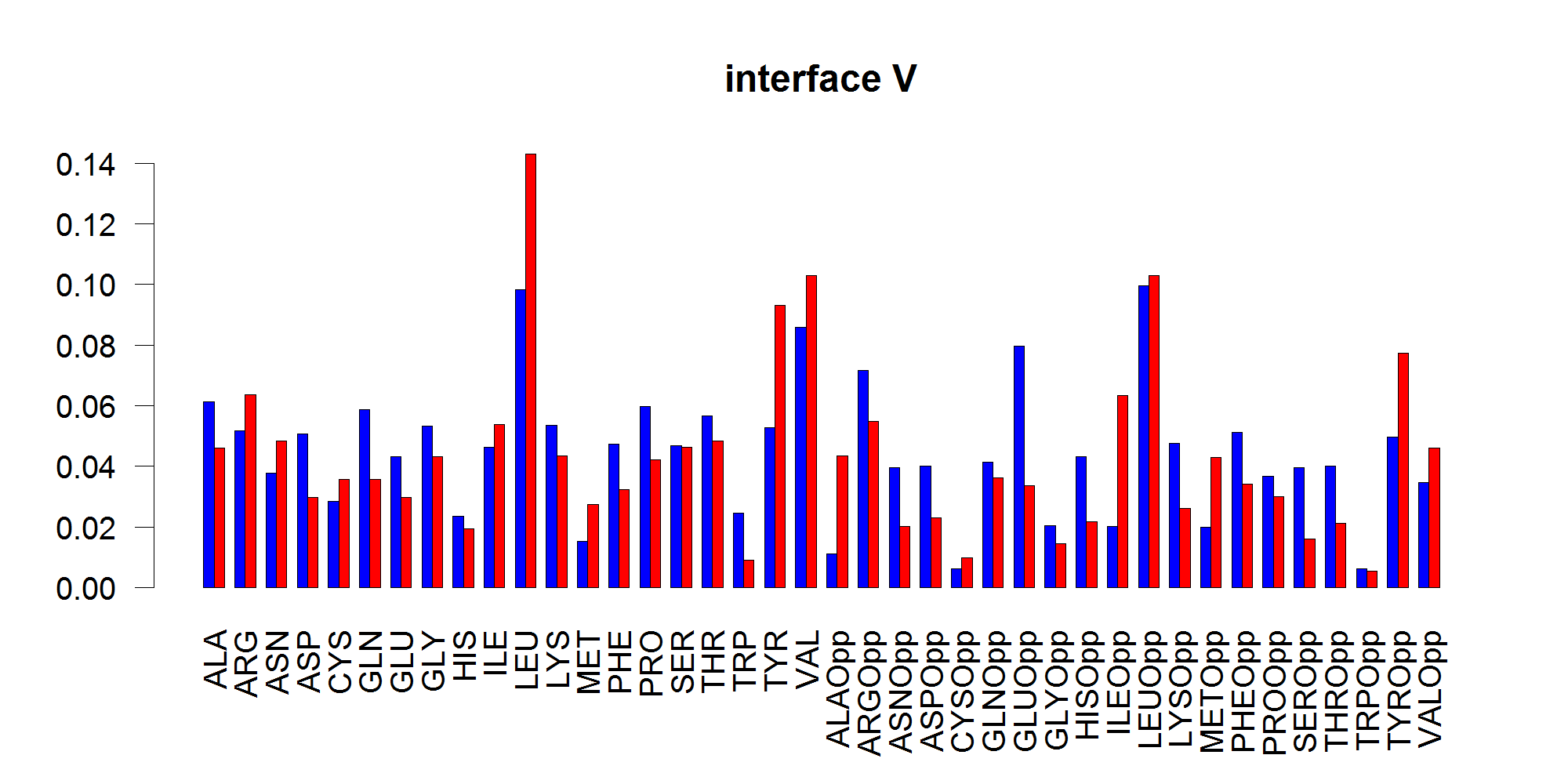

Supplement: Dataset S2 — Neighbouring residue profiles for mutations classed by WT residue. (ZIP) [file pone.0084598.s002.zip › neighbour_1/interface_V.tif]

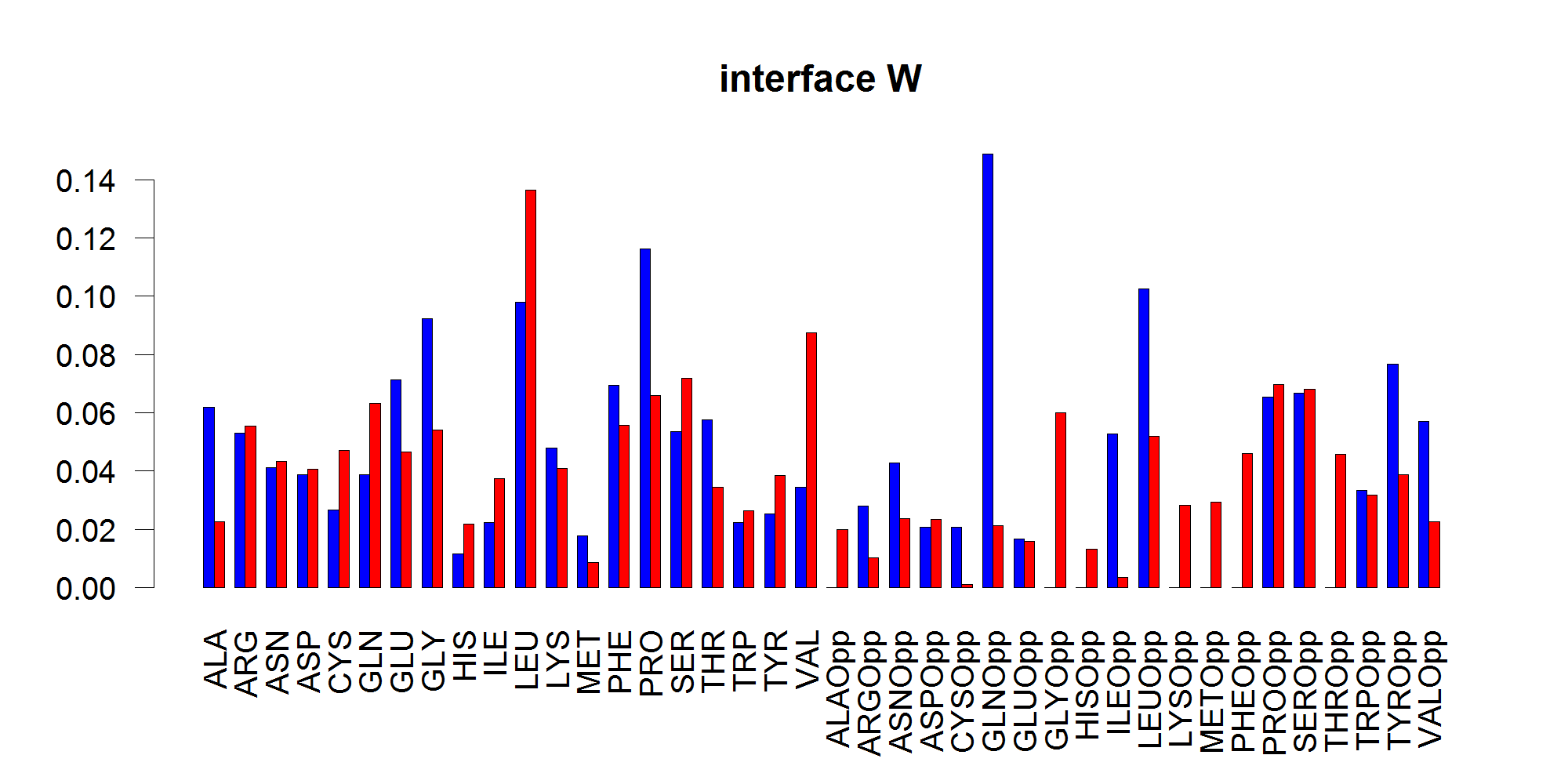

Supplement: Dataset S2 — Neighbouring residue profiles for mutations classed by WT residue. (ZIP) [file pone.0084598.s002.zip › neighbour_1/interface_W.tif]

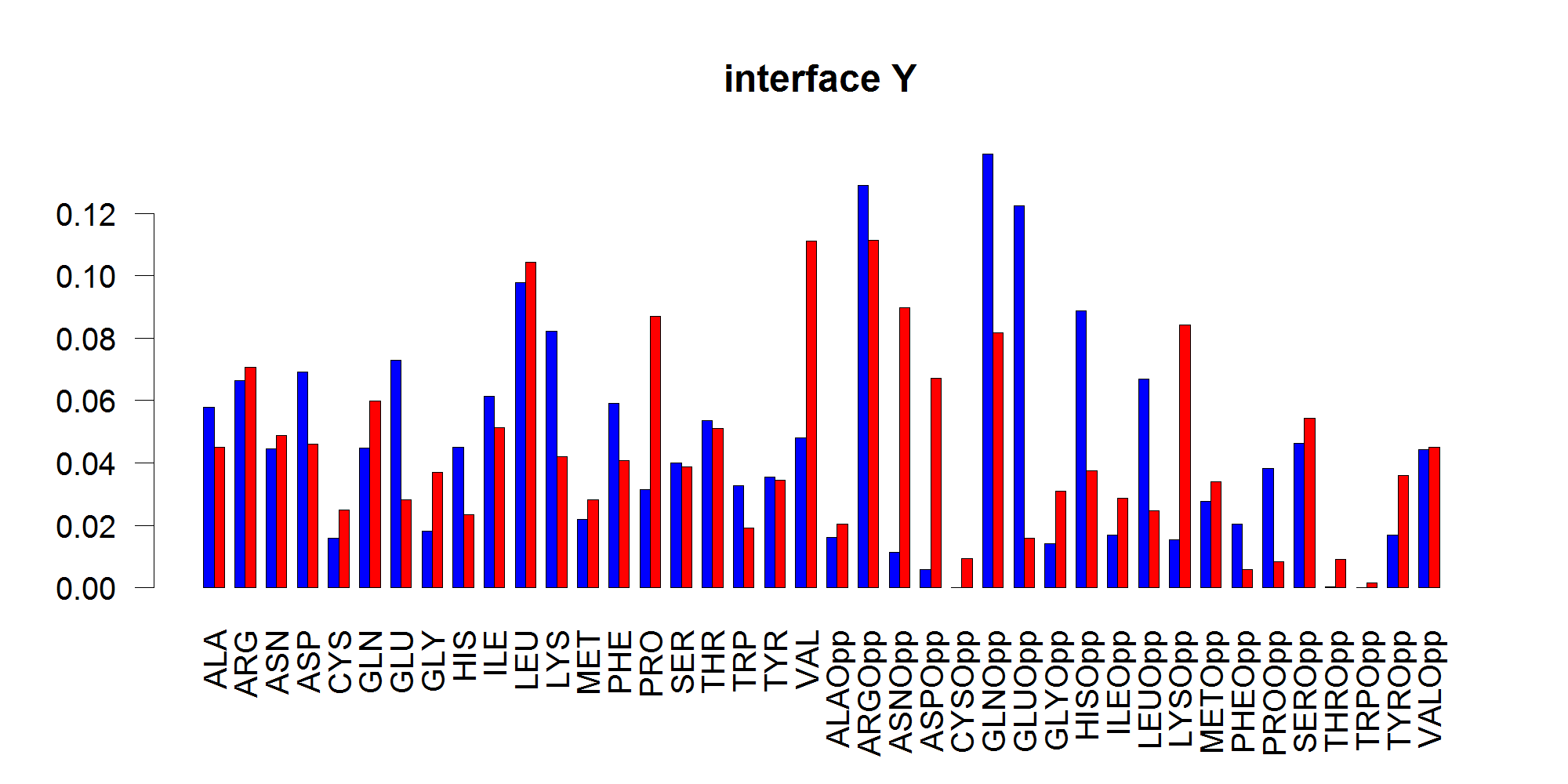

Supplement: Dataset S2 — Neighbouring residue profiles for mutations classed by WT residue. (ZIP) [file pone.0084598.s002.zip › neighbour_1/interface_Y.tif]

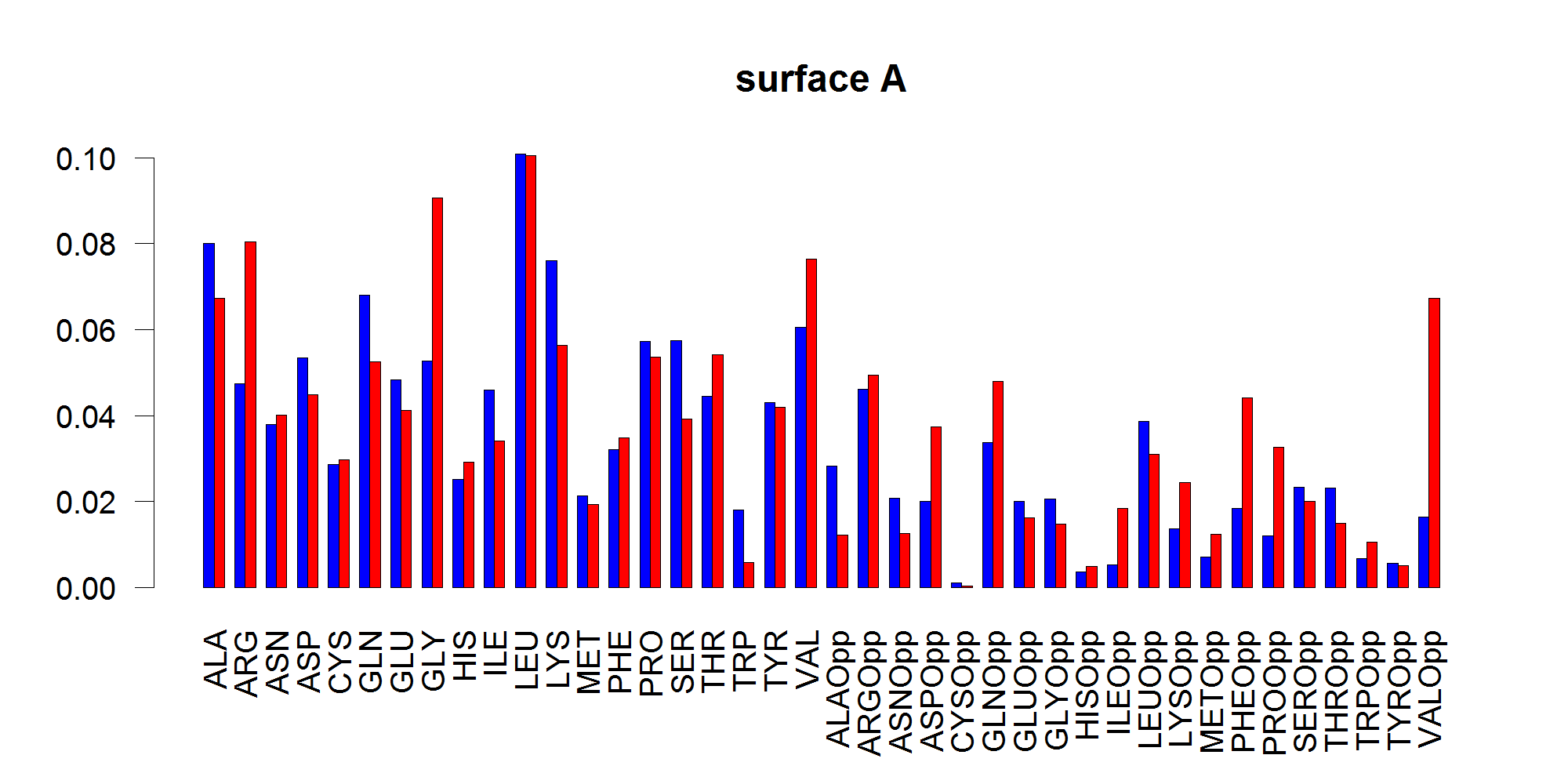

Supplement: Dataset S2 — Neighbouring residue profiles for mutations classed by WT residue. (ZIP) [file pone.0084598.s002.zip › neighbour_1/surface_A.tif]

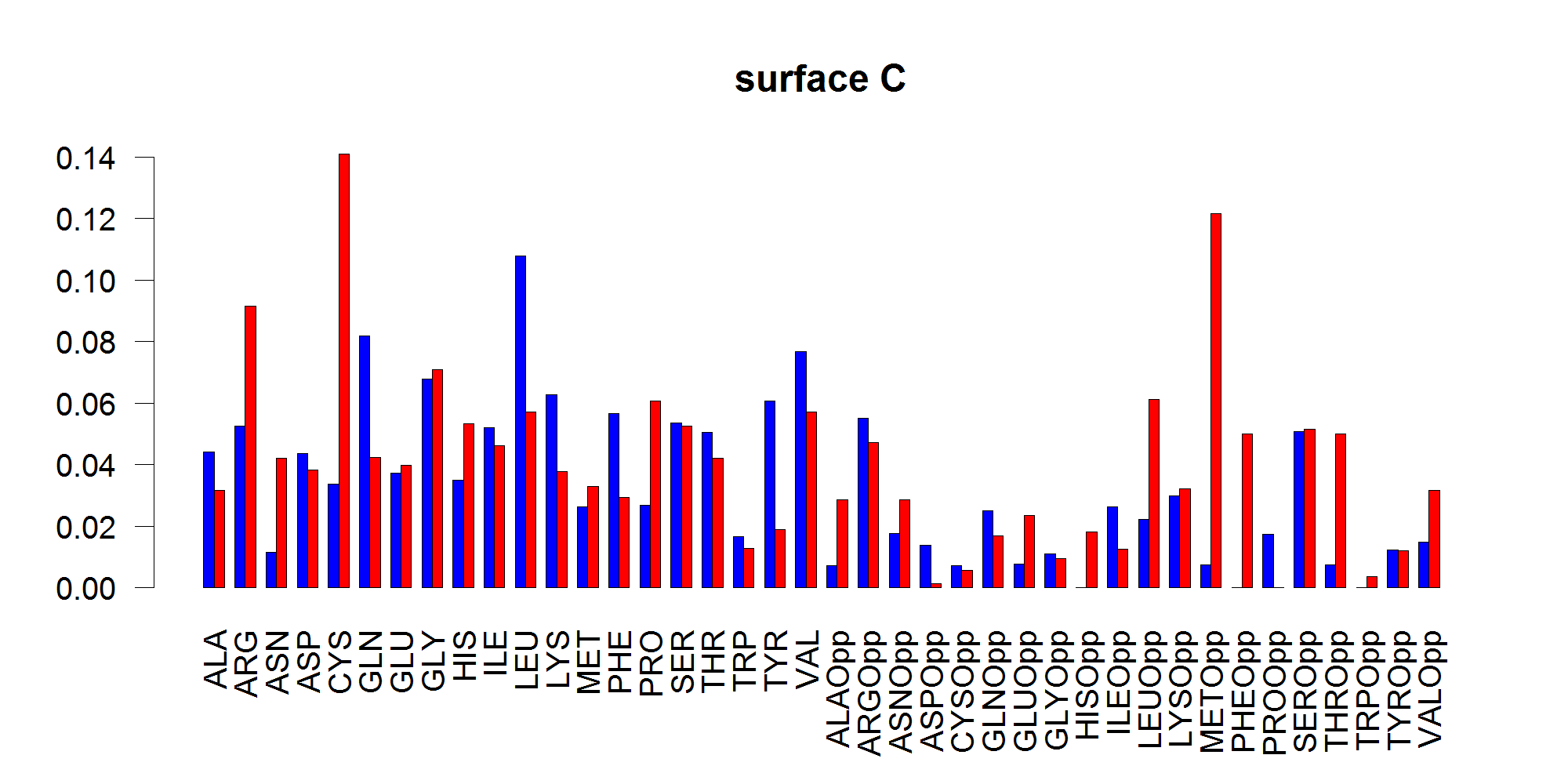

Supplement: Dataset S2 — Neighbouring residue profiles for mutations classed by WT residue. (ZIP) [file pone.0084598.s002.zip › neighbour_1/surface_C.tif]

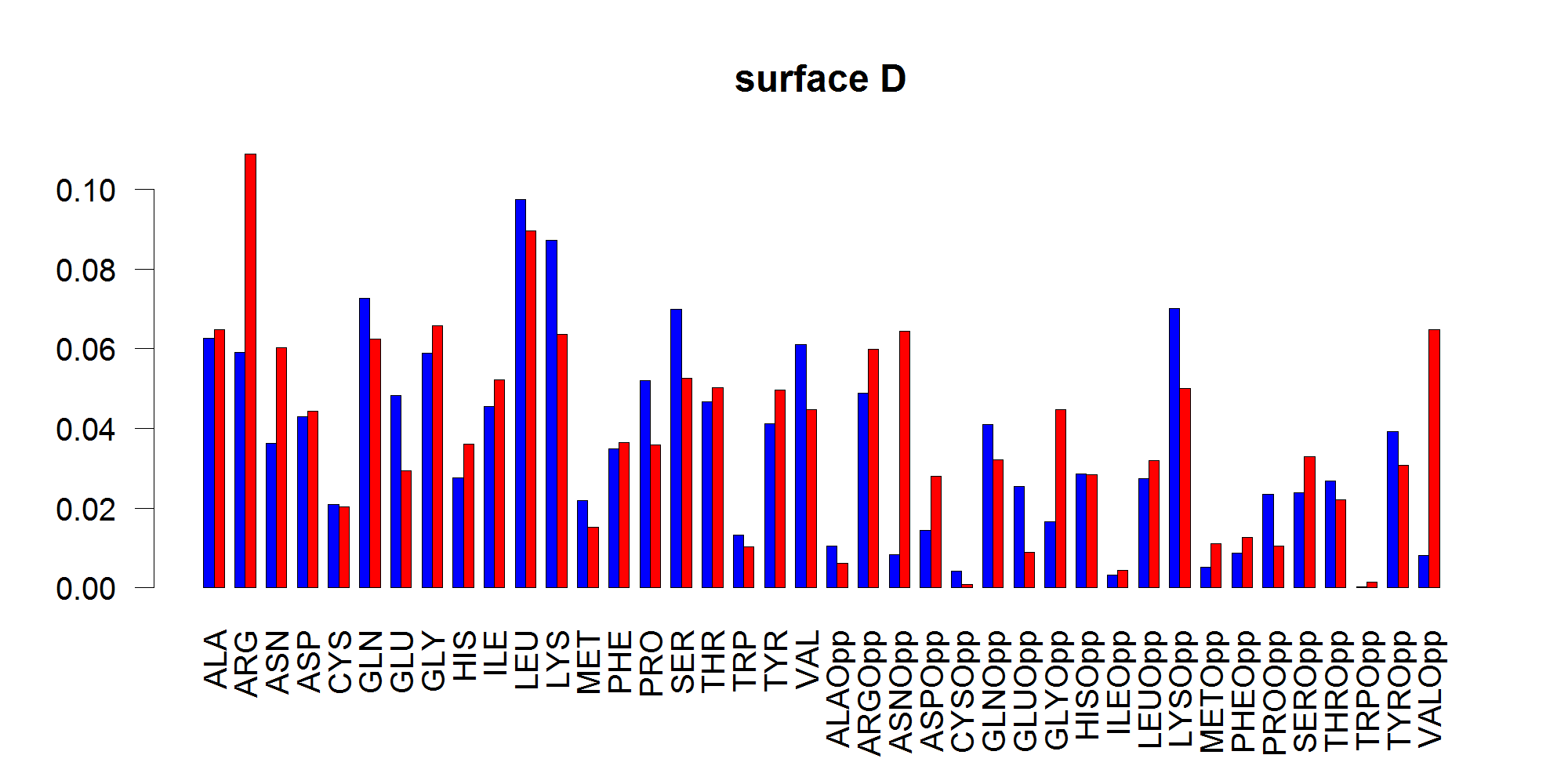

Supplement: Dataset S2 — Neighbouring residue profiles for mutations classed by WT residue. (ZIP) [file pone.0084598.s002.zip › neighbour_1/surface_D.tif]

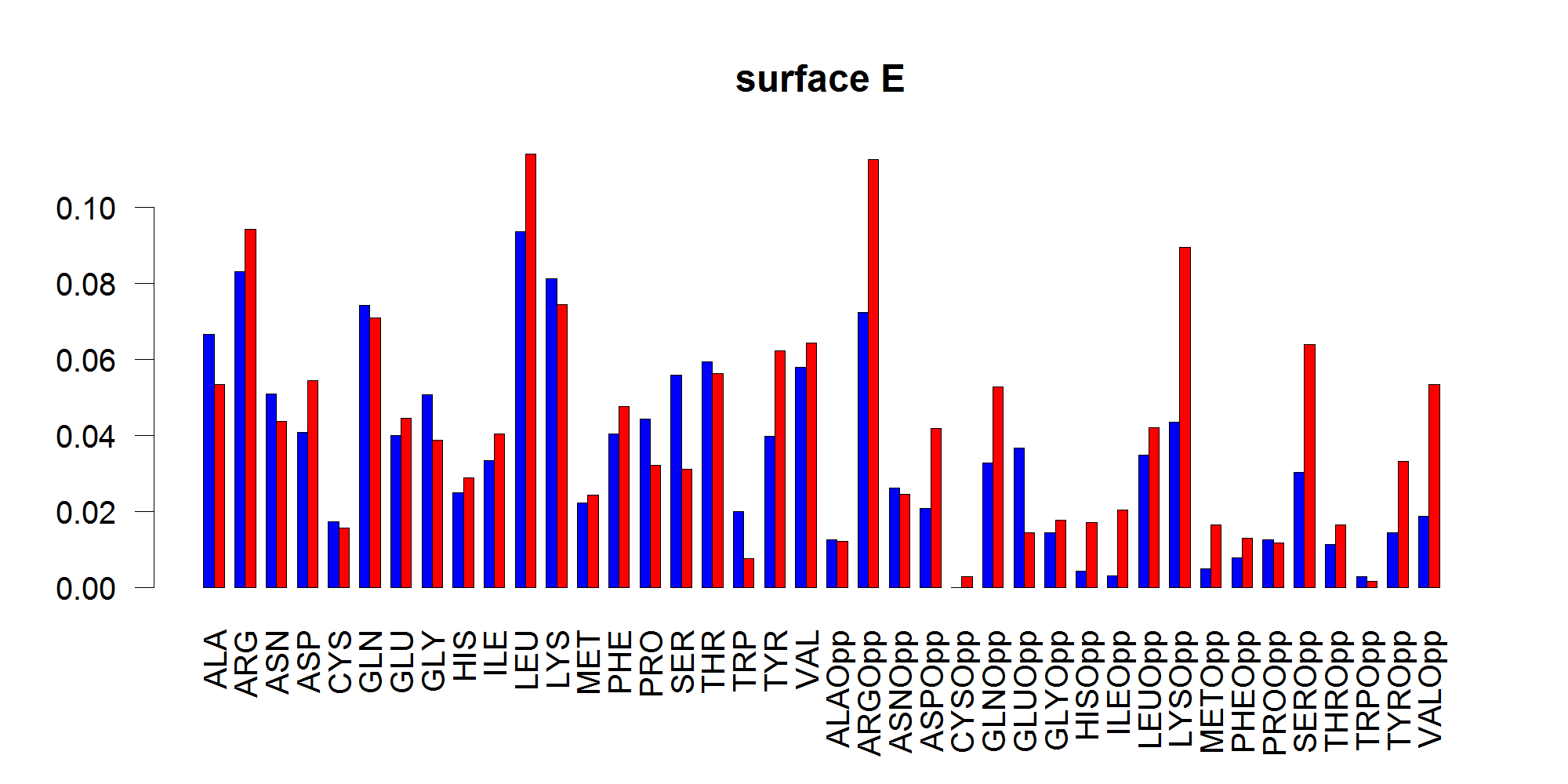

Supplement: Dataset S2 — Neighbouring residue profiles for mutations classed by WT residue. (ZIP) [file pone.0084598.s002.zip › neighbour_1/surface_E.tif]

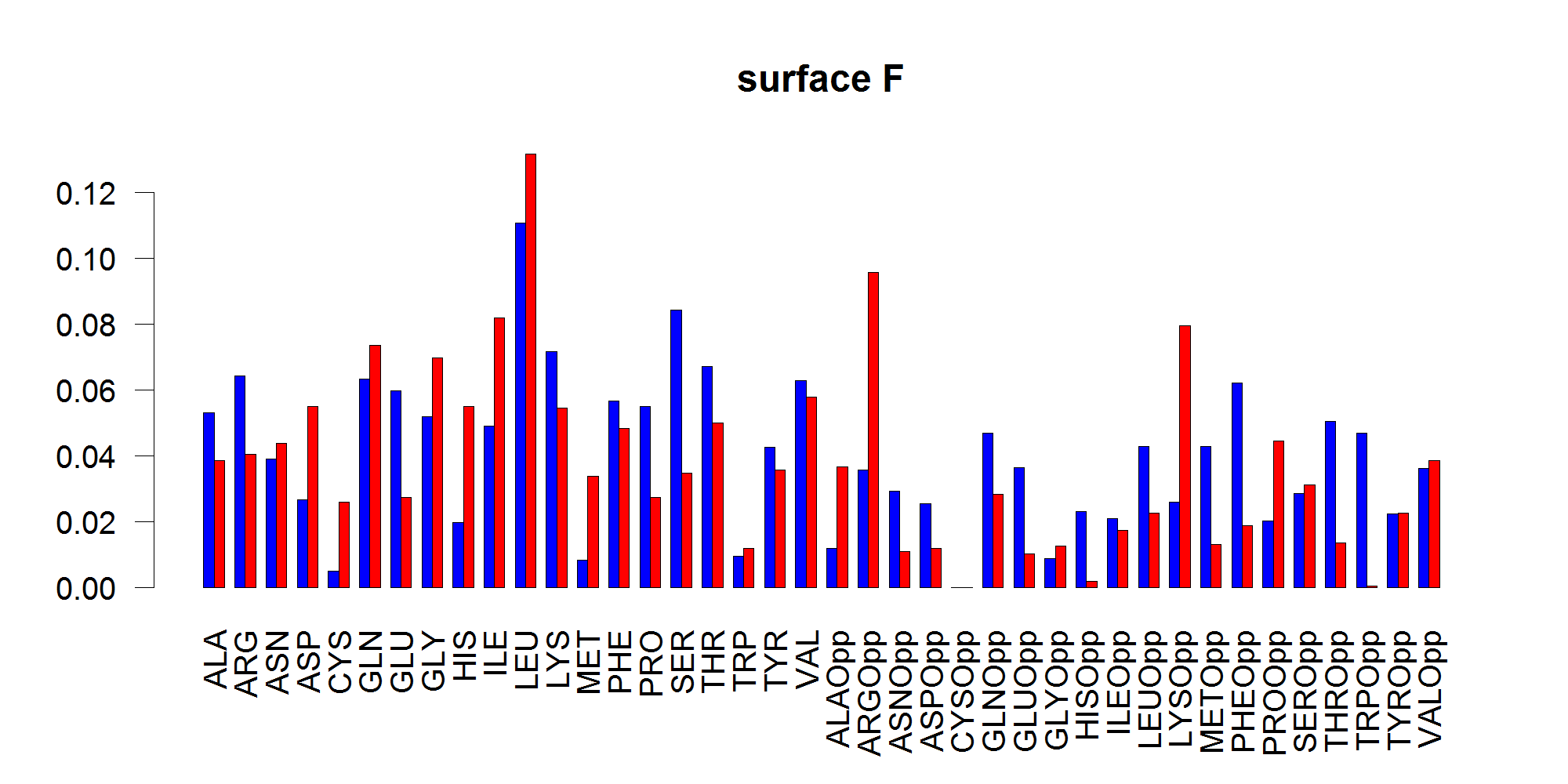

Supplement: Dataset S2 — Neighbouring residue profiles for mutations classed by WT residue. (ZIP) [file pone.0084598.s002.zip › neighbour_1/surface_F.tif]

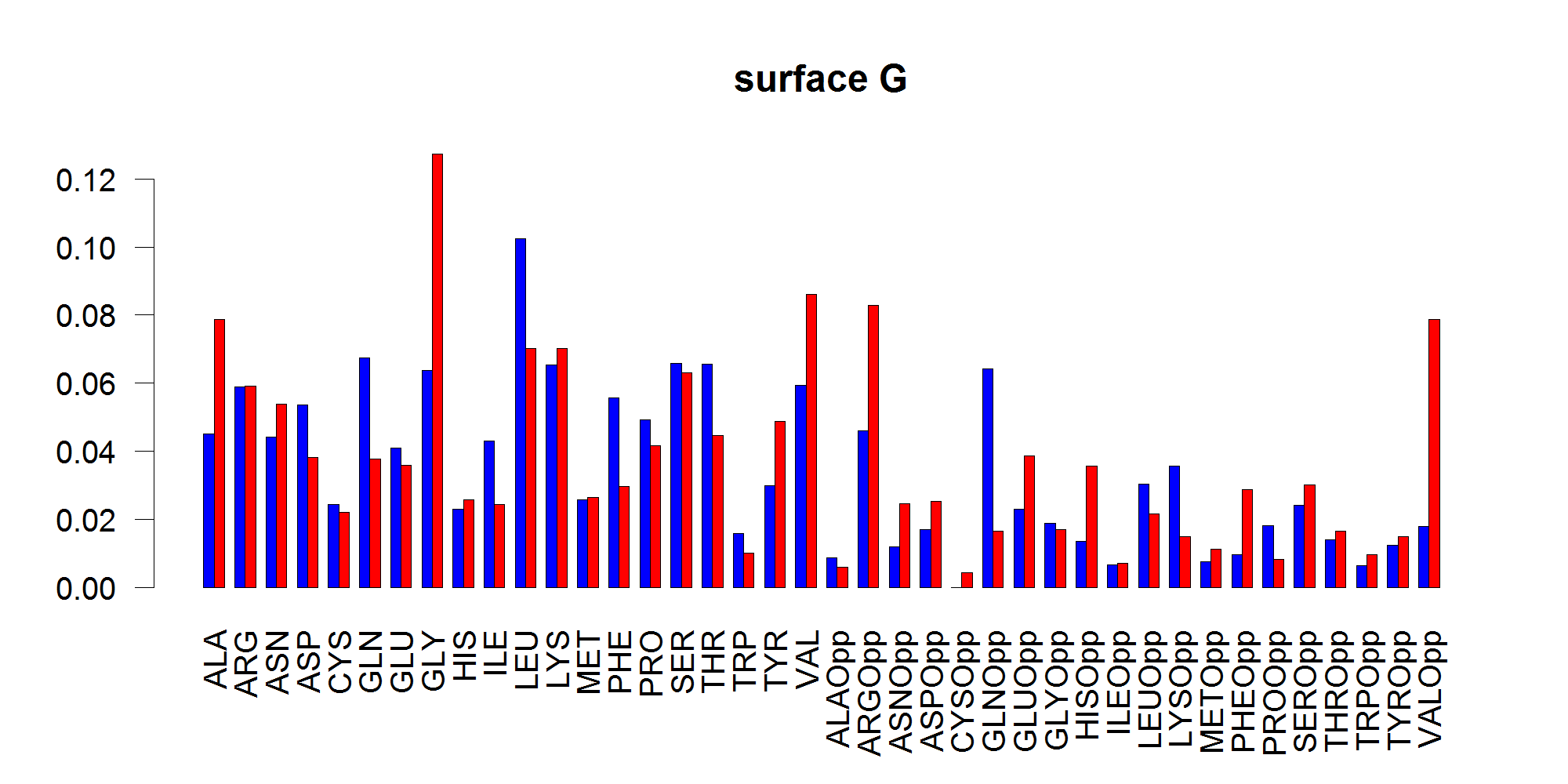

Supplement: Dataset S2 — Neighbouring residue profiles for mutations classed by WT residue. (ZIP) [file pone.0084598.s002.zip › neighbour_1/surface_G.tif]

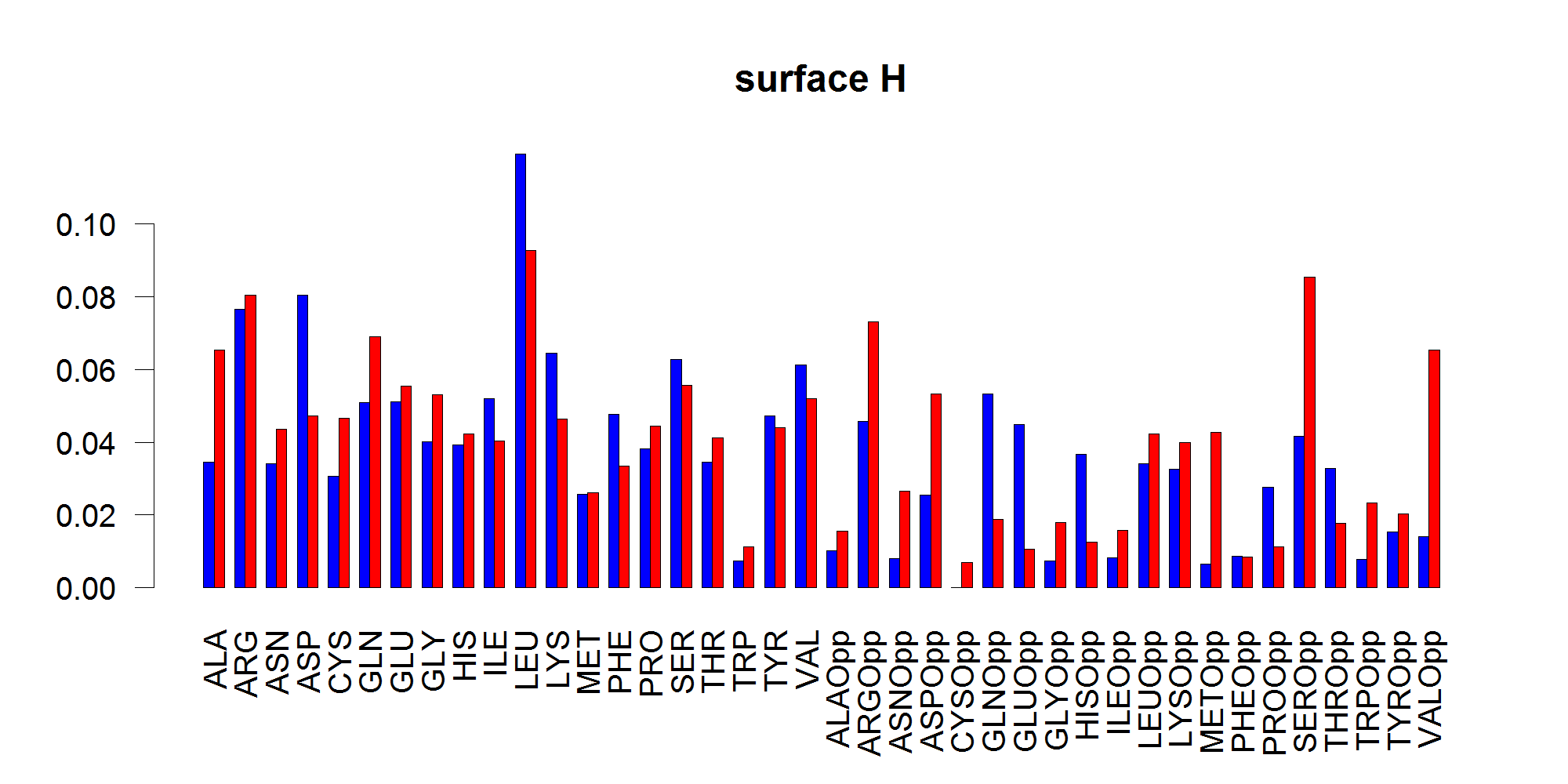

Supplement: Dataset S2 — Neighbouring residue profiles for mutations classed by WT residue. (ZIP) [file pone.0084598.s002.zip › neighbour_1/surface_H.tif]

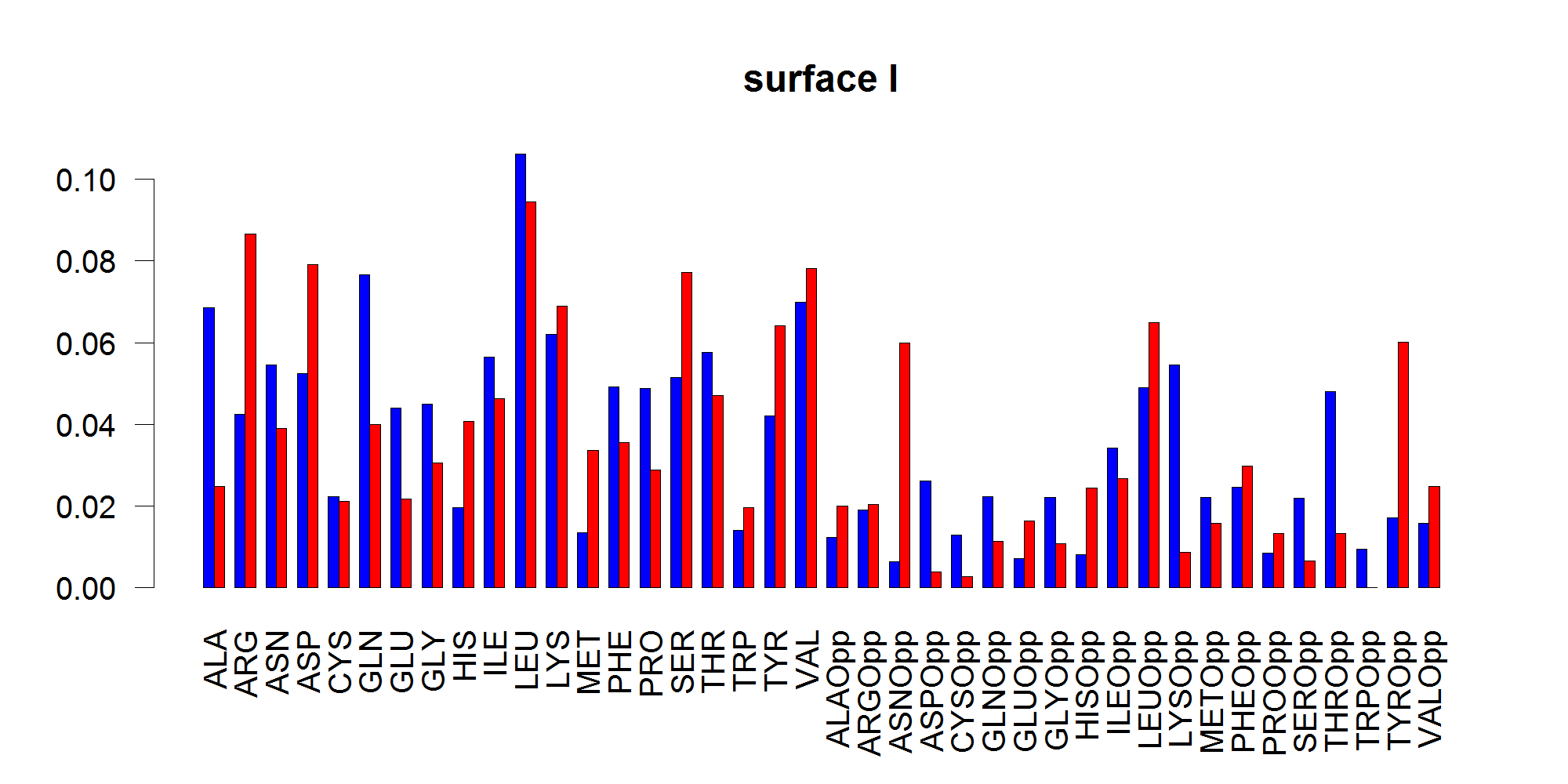

Supplement: Dataset S2 — Neighbouring residue profiles for mutations classed by WT residue. (ZIP) [file pone.0084598.s002.zip › neighbour_1/surface_I.tif]

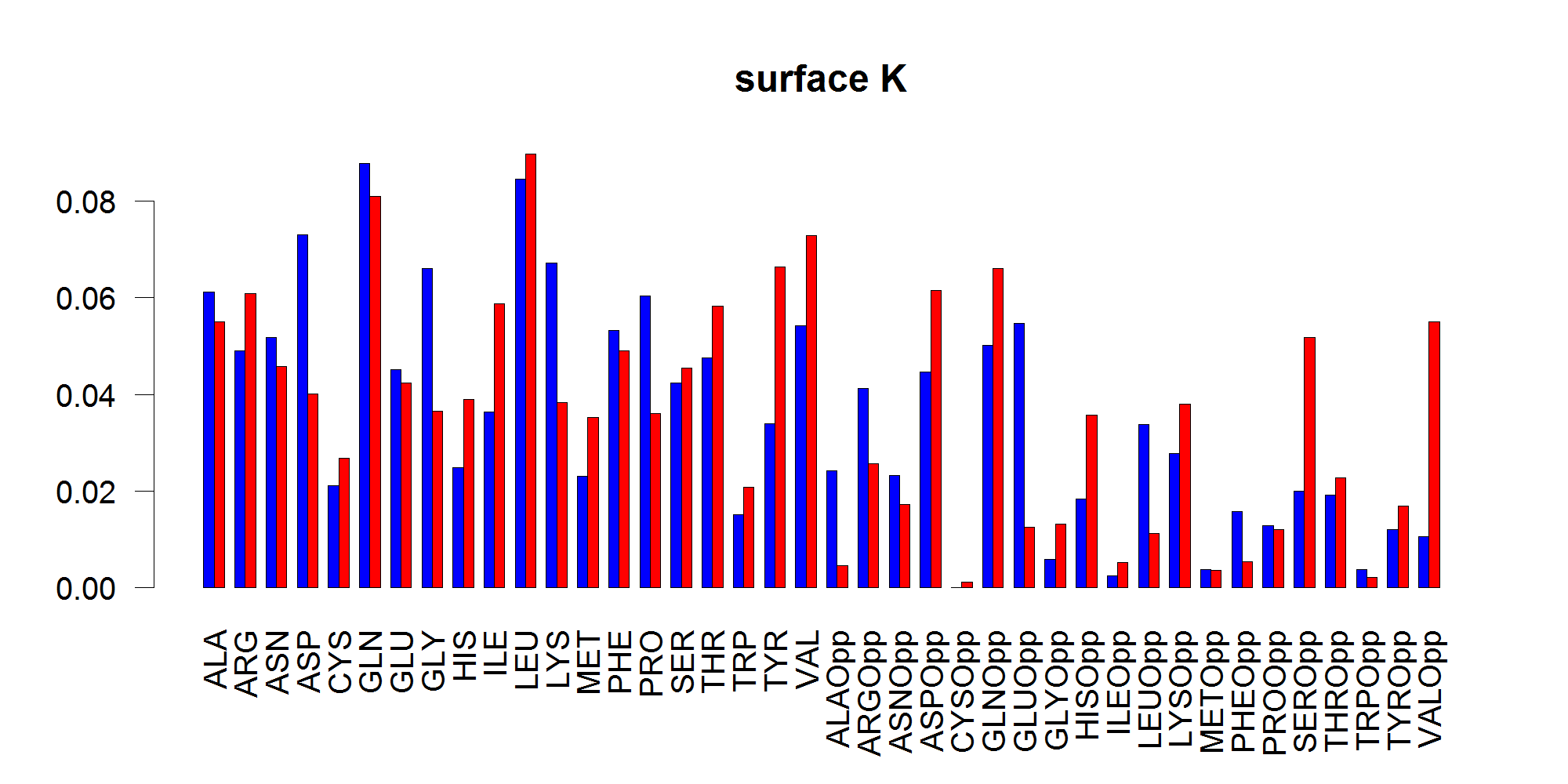

Supplement: Dataset S2 — Neighbouring residue profiles for mutations classed by WT residue. (ZIP) [file pone.0084598.s002.zip › neighbour_1/surface_K.tif]

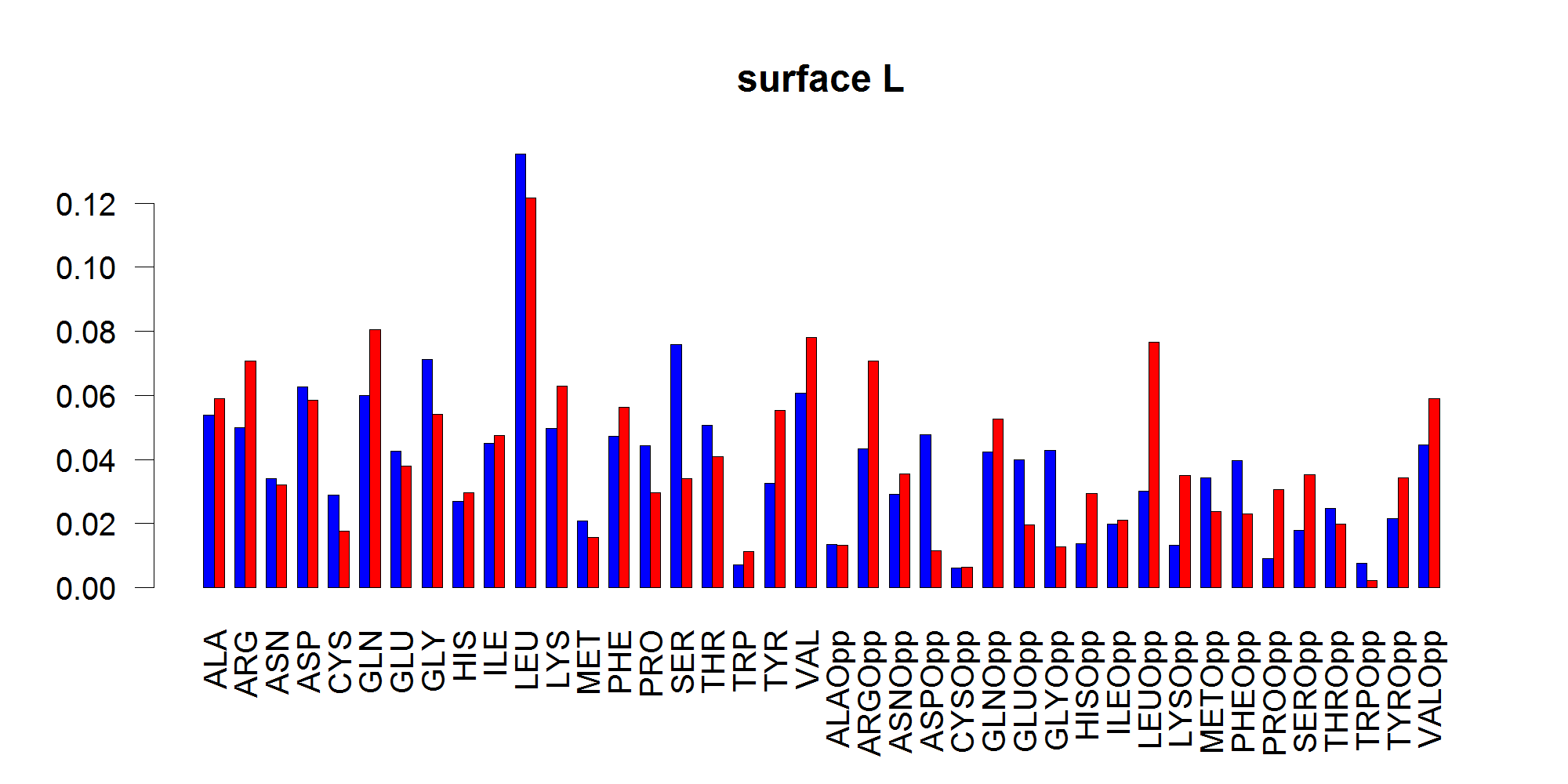

Supplement: Dataset S2 — Neighbouring residue profiles for mutations classed by WT residue. (ZIP) [file pone.0084598.s002.zip › neighbour_1/surface_L.tif]

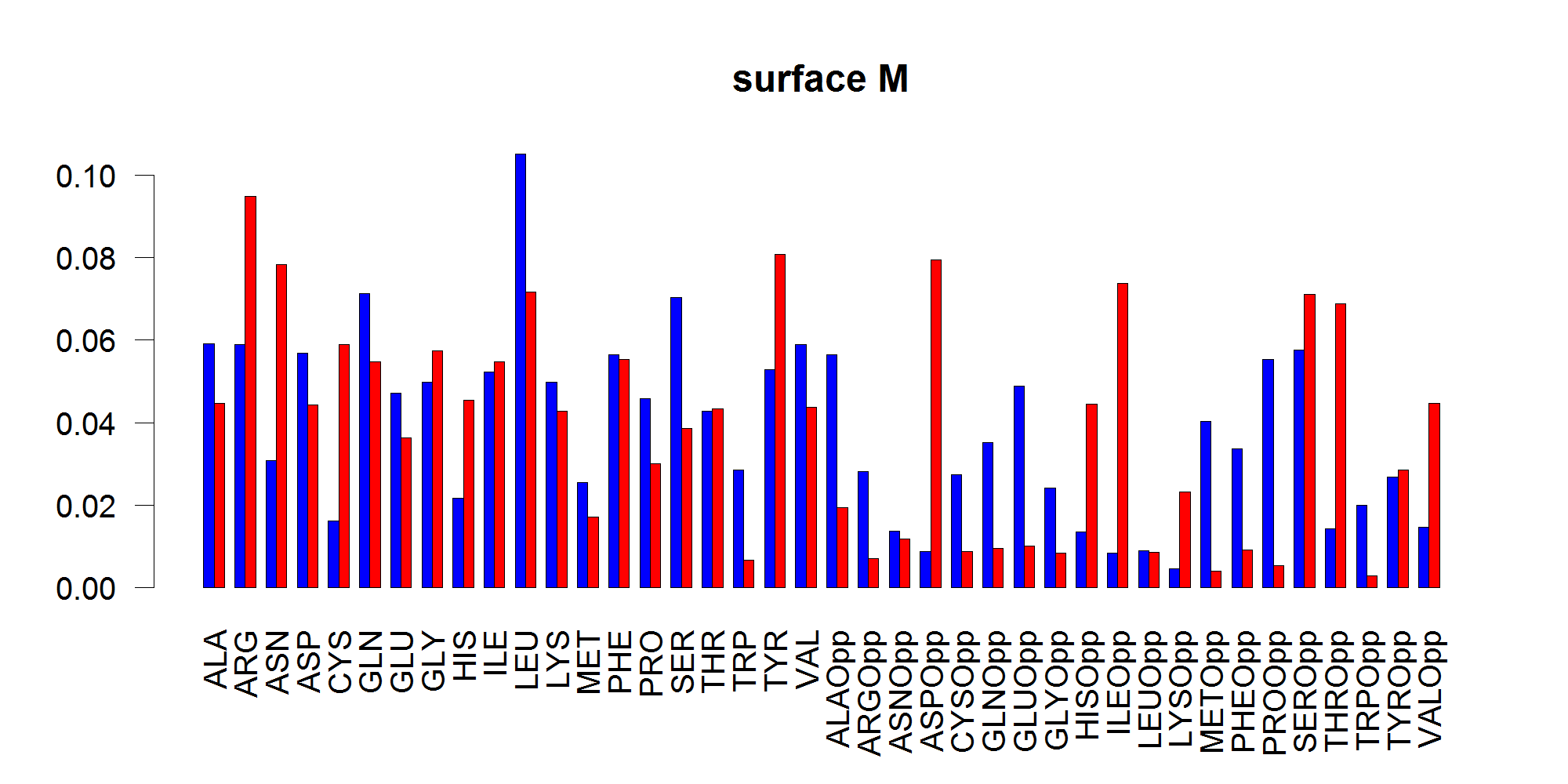

Supplement: Dataset S2 — Neighbouring residue profiles for mutations classed by WT residue. (ZIP) [file pone.0084598.s002.zip › neighbour_1/surface_M.tif]

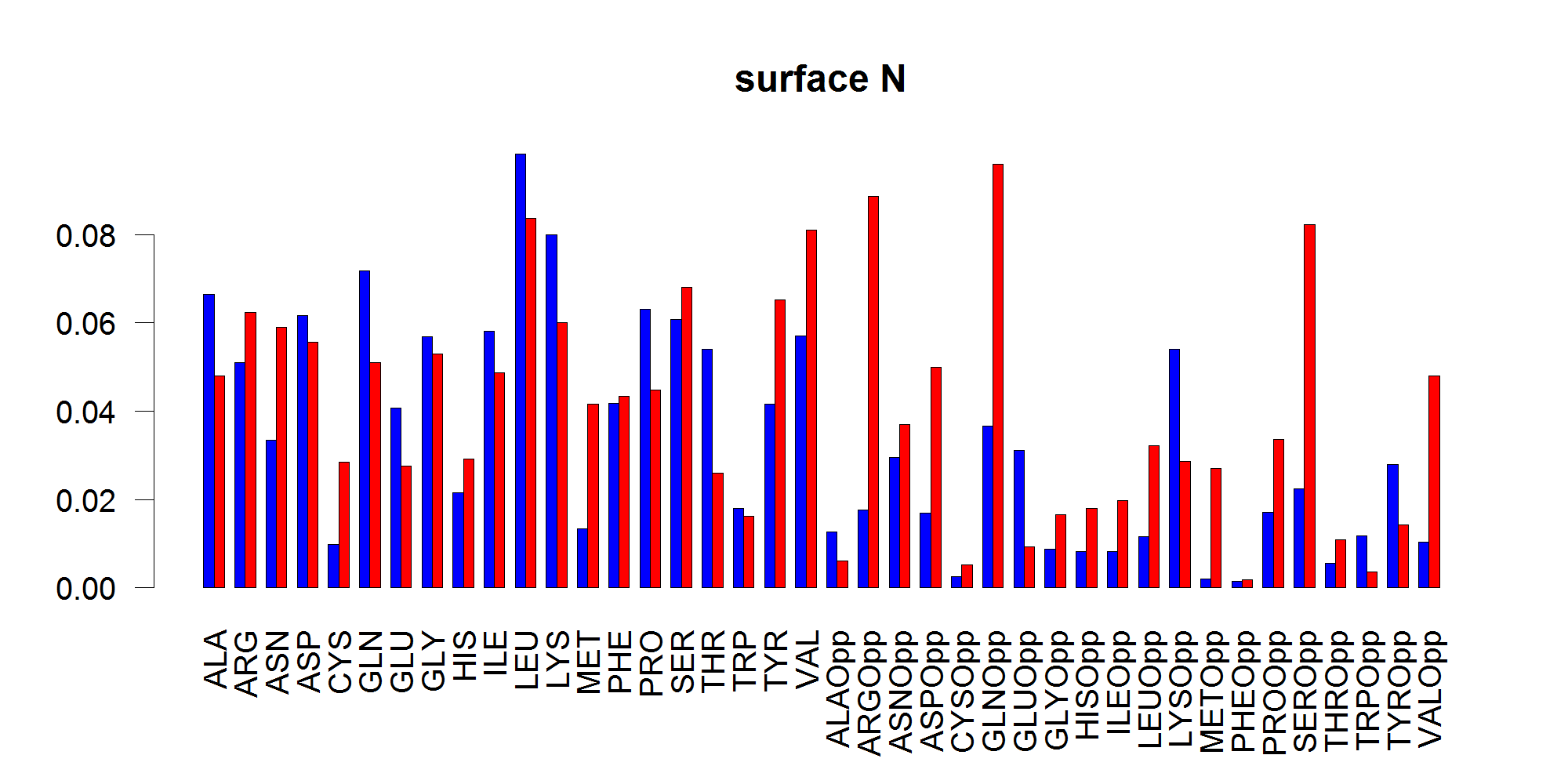

Supplement: Dataset S2 — Neighbouring residue profiles for mutations classed by WT residue. (ZIP) [file pone.0084598.s002.zip › neighbour_1/surface_N.tif]

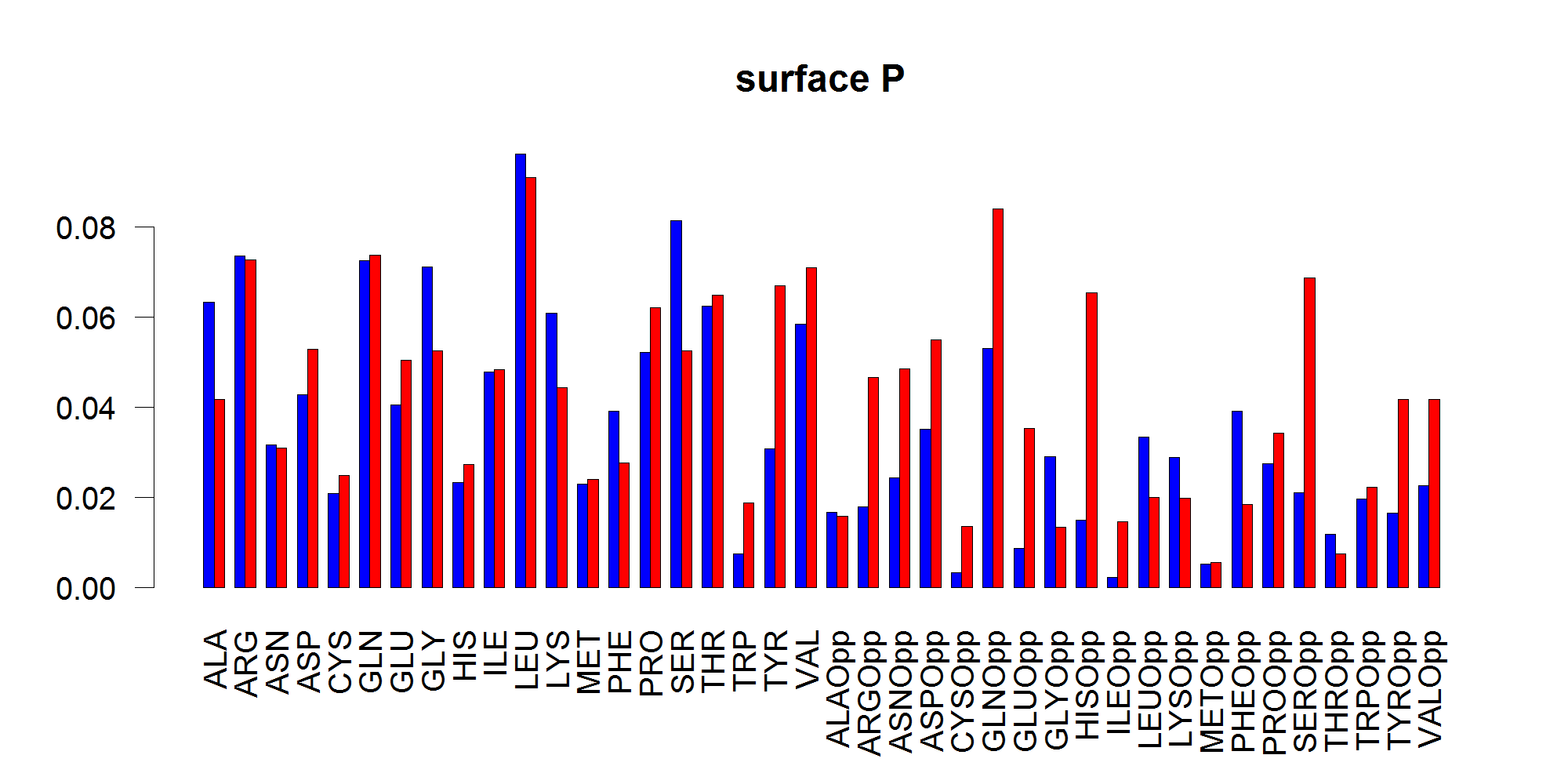

Supplement: Dataset S2 — Neighbouring residue profiles for mutations classed by WT residue. (ZIP) [file pone.0084598.s002.zip › neighbour_1/surface_P.tif]

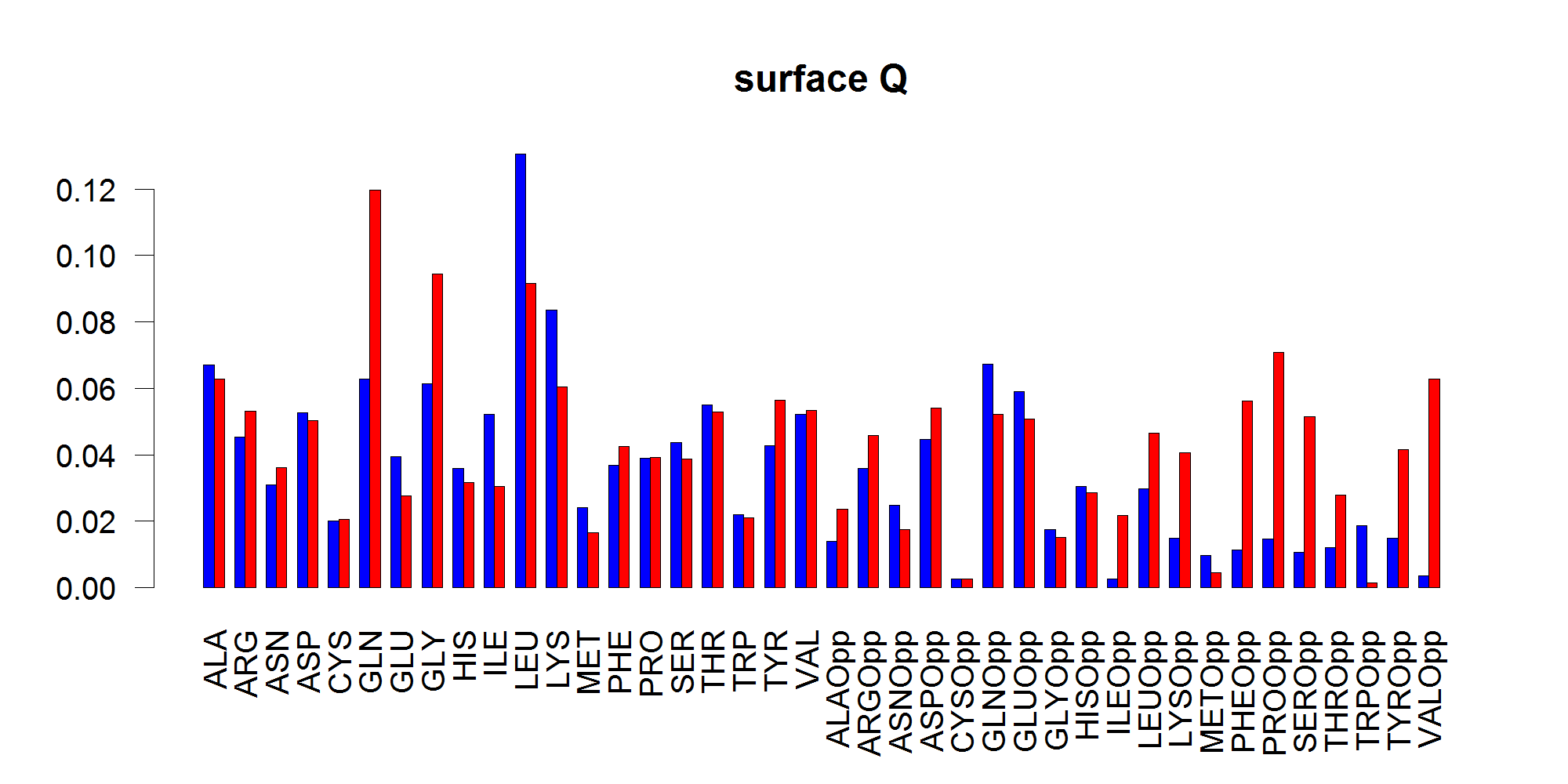

Supplement: Dataset S2 — Neighbouring residue profiles for mutations classed by WT residue. (ZIP) [file pone.0084598.s002.zip › neighbour_1/surface_Q.tif]

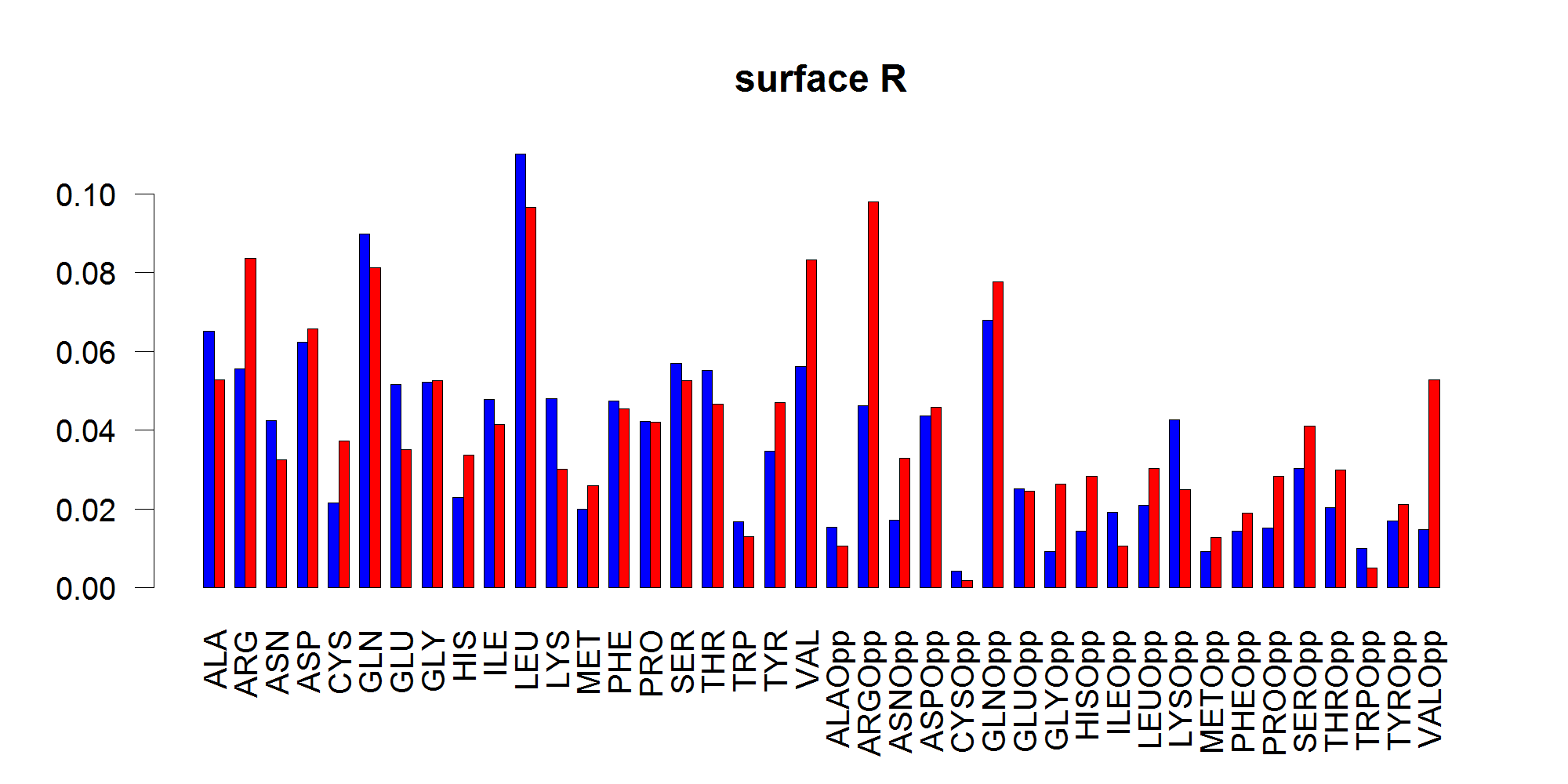

Supplement: Dataset S2 — Neighbouring residue profiles for mutations classed by WT residue. (ZIP) [file pone.0084598.s002.zip › neighbour_1/surface_R.tif]

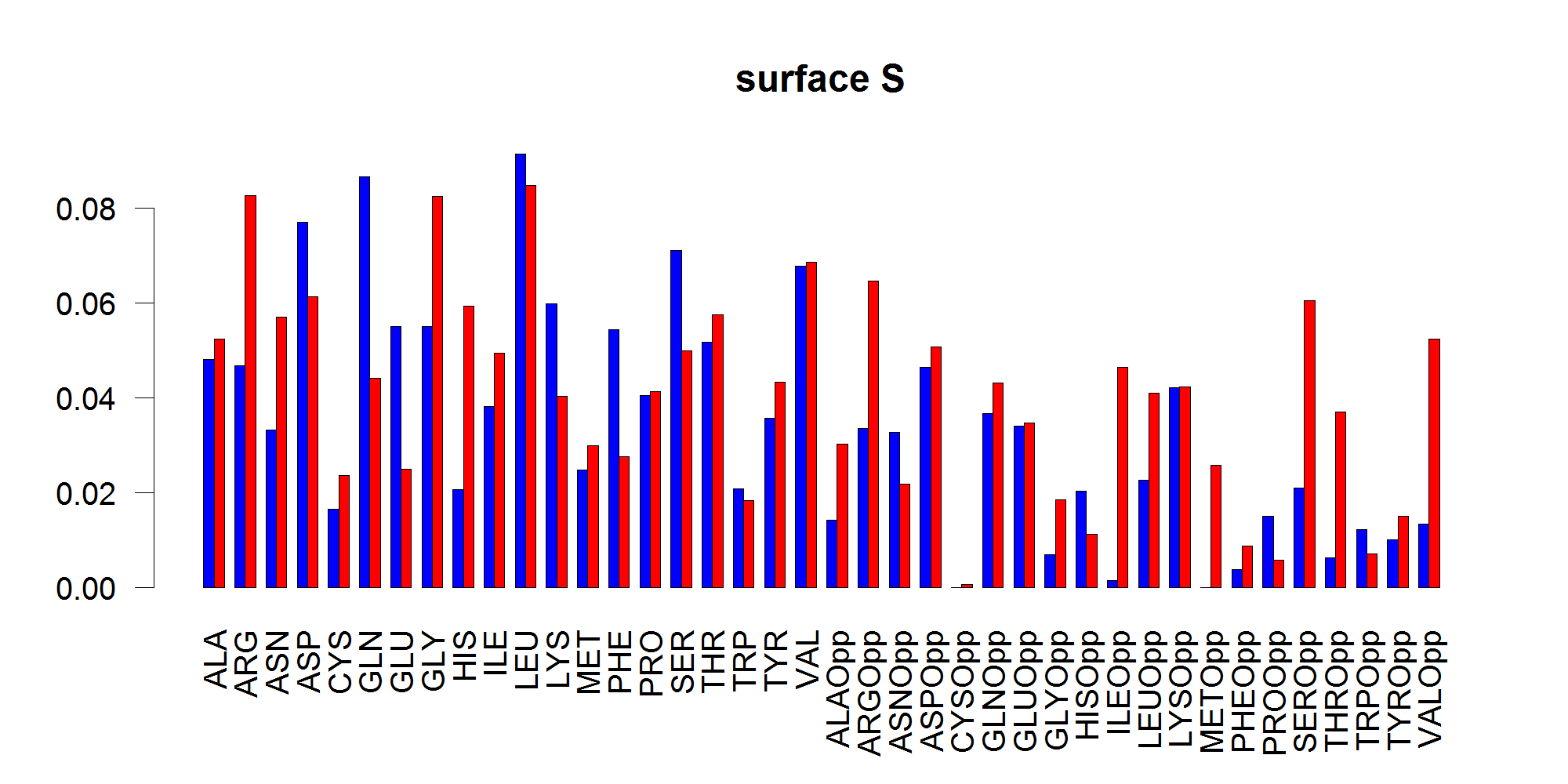

Supplement: Dataset S2 — Neighbouring residue profiles for mutations classed by WT residue. (ZIP) [file pone.0084598.s002.zip › neighbour_1/surface_S.tif]

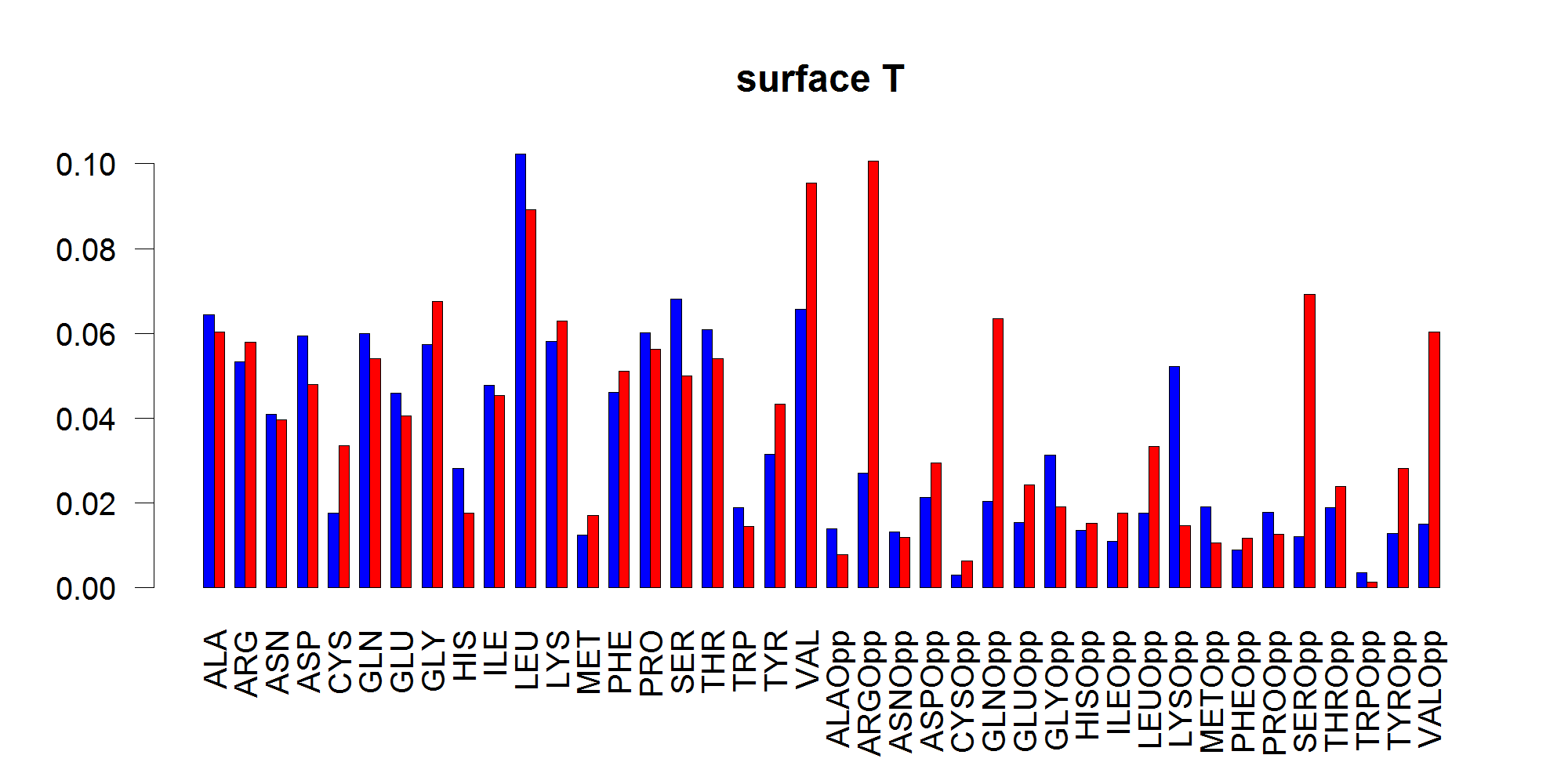

Supplement: Dataset S2 — Neighbouring residue profiles for mutations classed by WT residue. (ZIP) [file pone.0084598.s002.zip › neighbour_1/surface_T.tif]

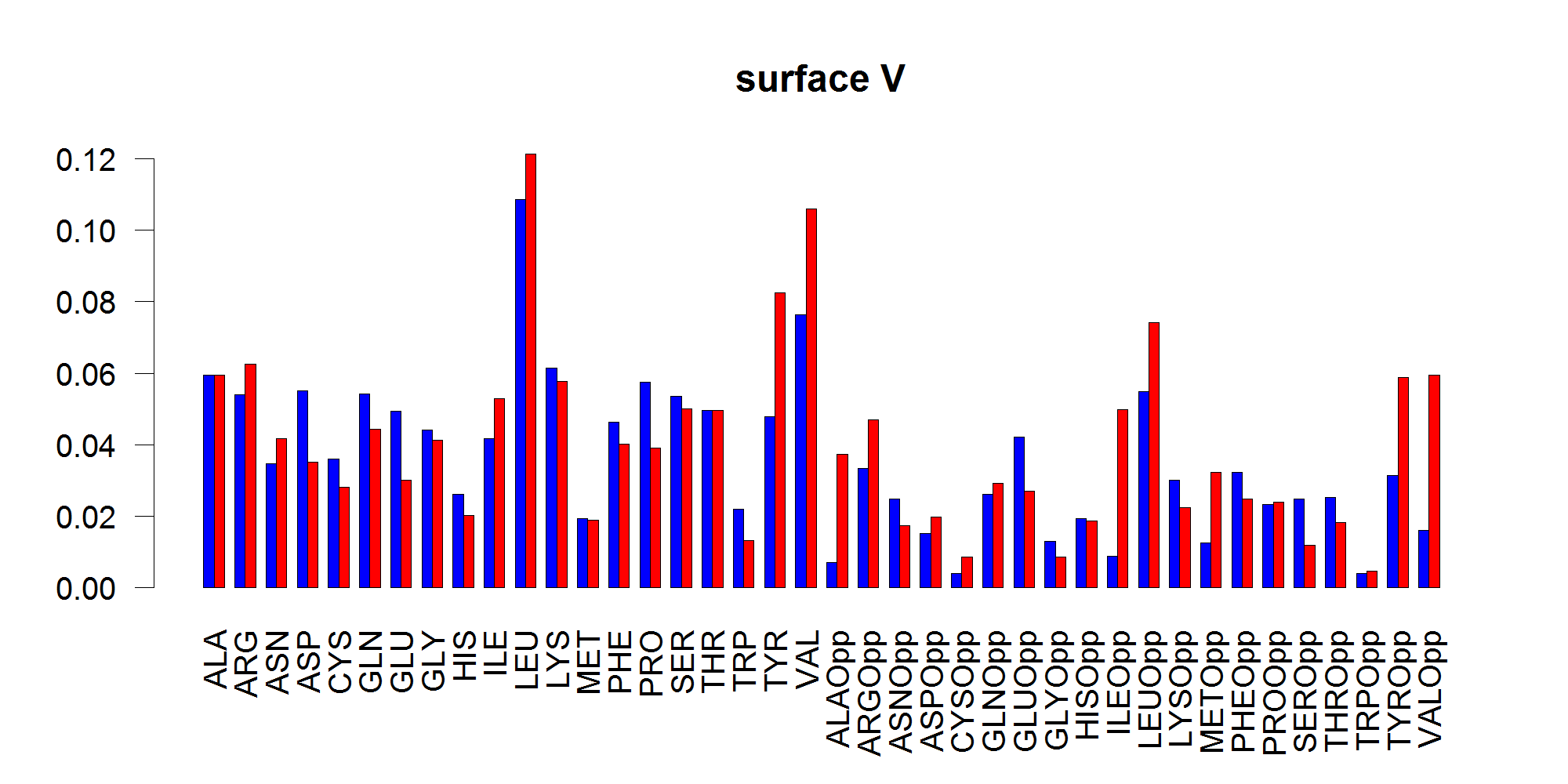

Supplement: Dataset S2 — Neighbouring residue profiles for mutations classed by WT residue. (ZIP) [file pone.0084598.s002.zip › neighbour_1/surface_V.tif]

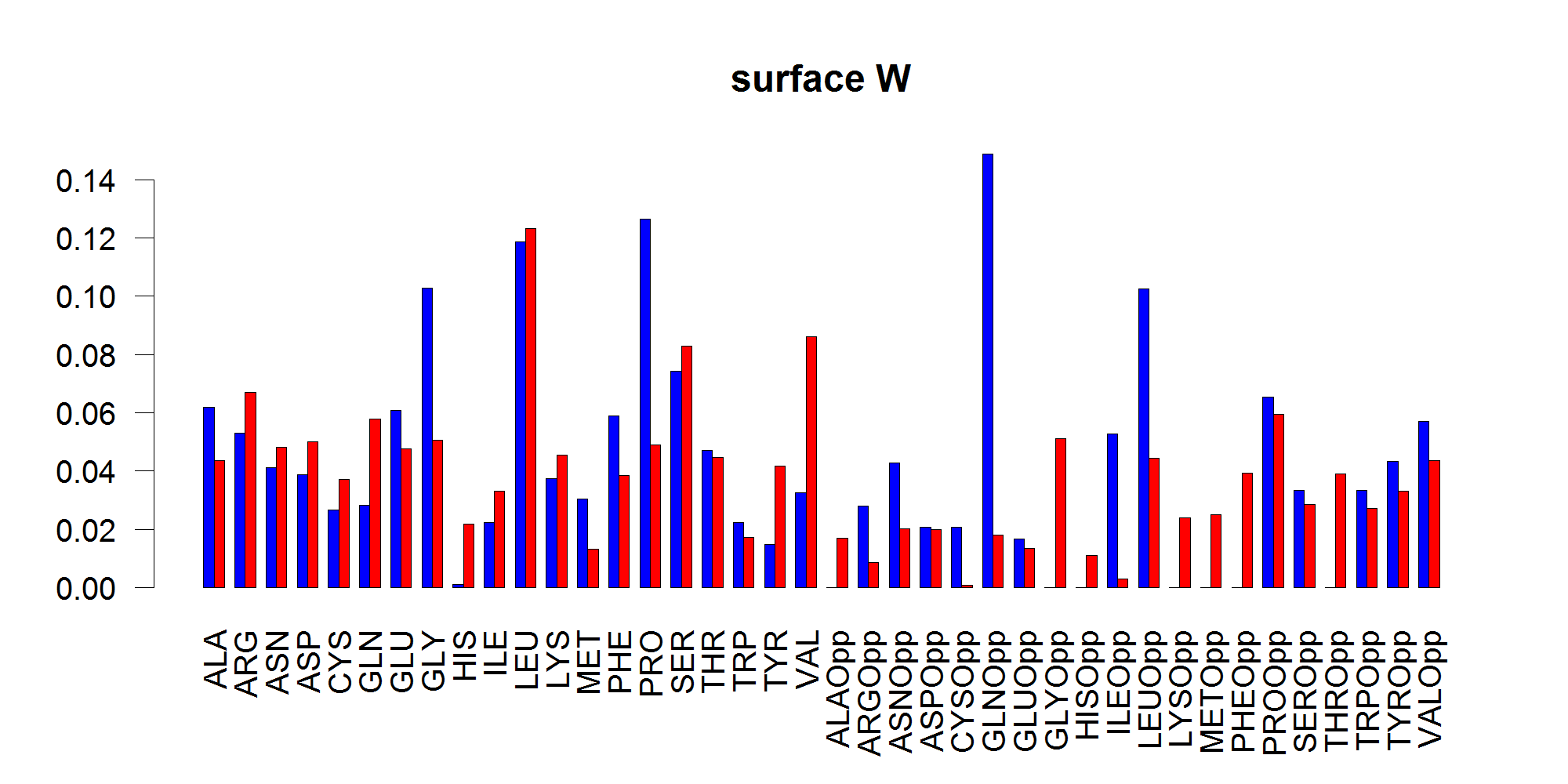

Supplement: Dataset S2 — Neighbouring residue profiles for mutations classed by WT residue. (ZIP) [file pone.0084598.s002.zip › neighbour_1/surface_W.tif]

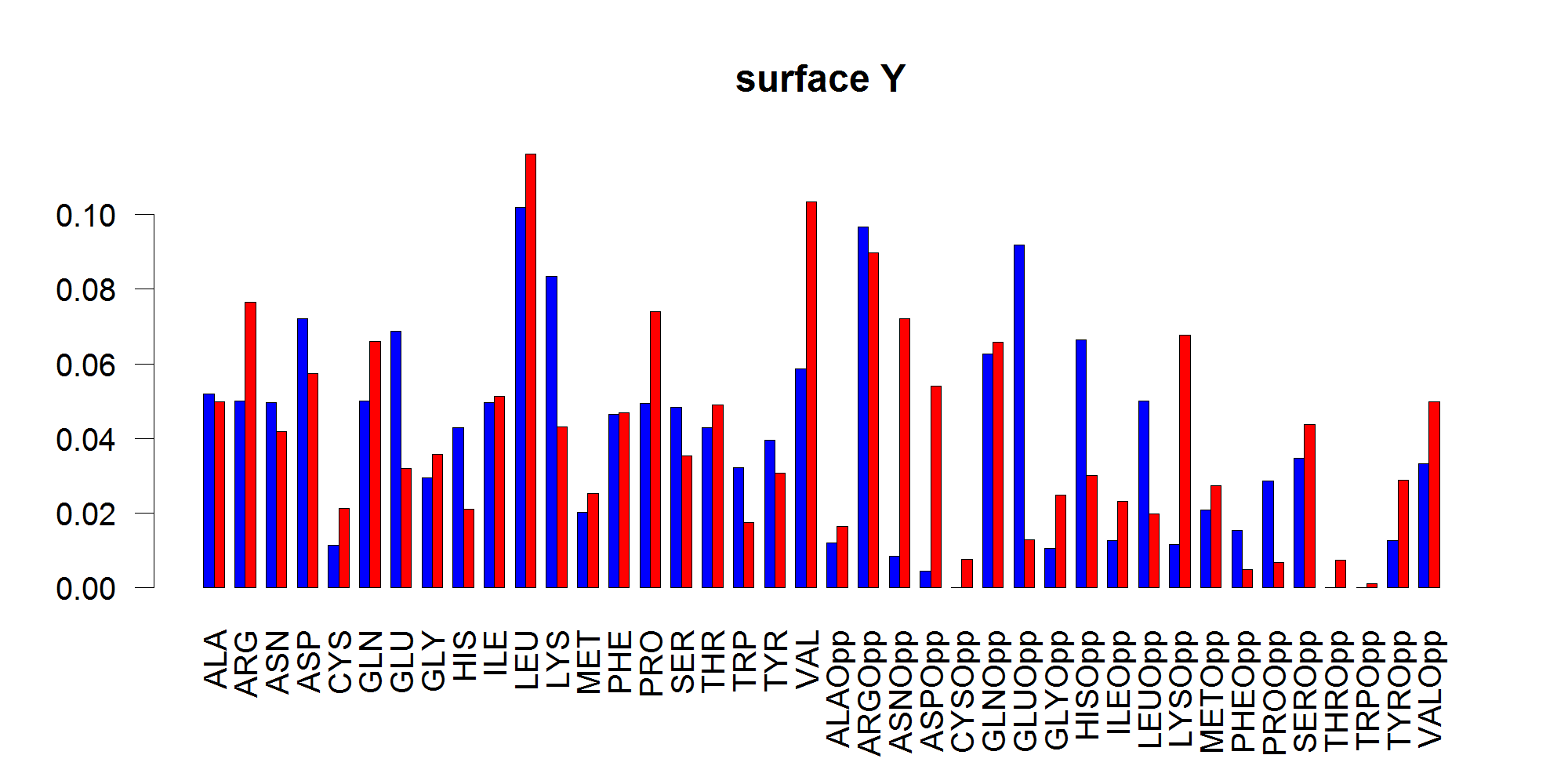

Supplement: Dataset S2 — Neighbouring residue profiles for mutations classed by WT residue. (ZIP) [file pone.0084598.s002.zip › neighbour_1/surface_Y.tif]

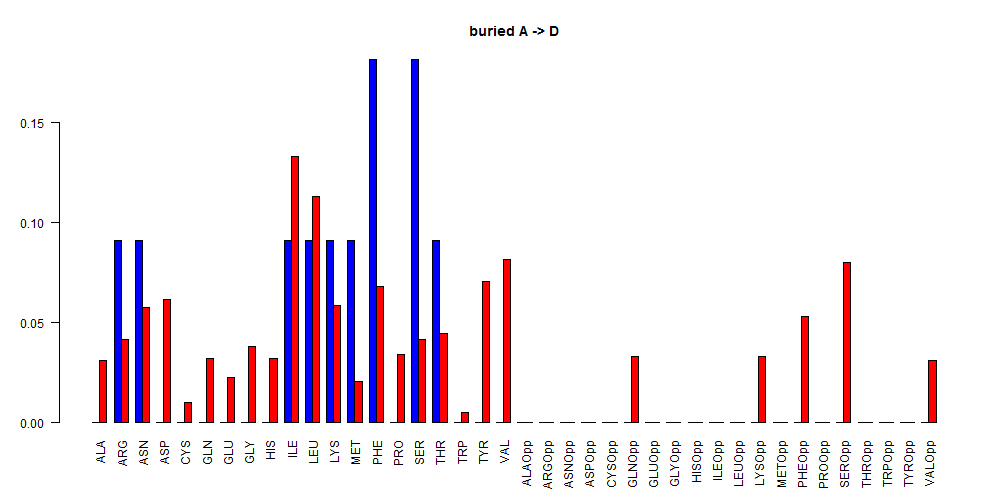

Supplement: Dataset S3 — Neighbouring residue profiles for mutations classed by substitution. (ZIP) [file pone.0084598.s003.zip › neighbour_2/buried_A_D.tif]

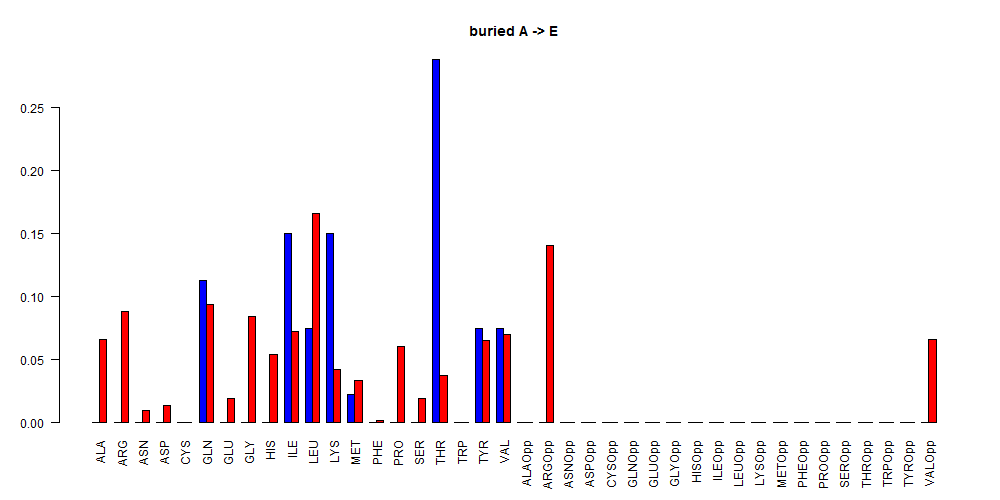

Supplement: Dataset S3 — Neighbouring residue profiles for mutations classed by substitution. (ZIP) [file pone.0084598.s003.zip › neighbour_2/buried_A_E.tif]

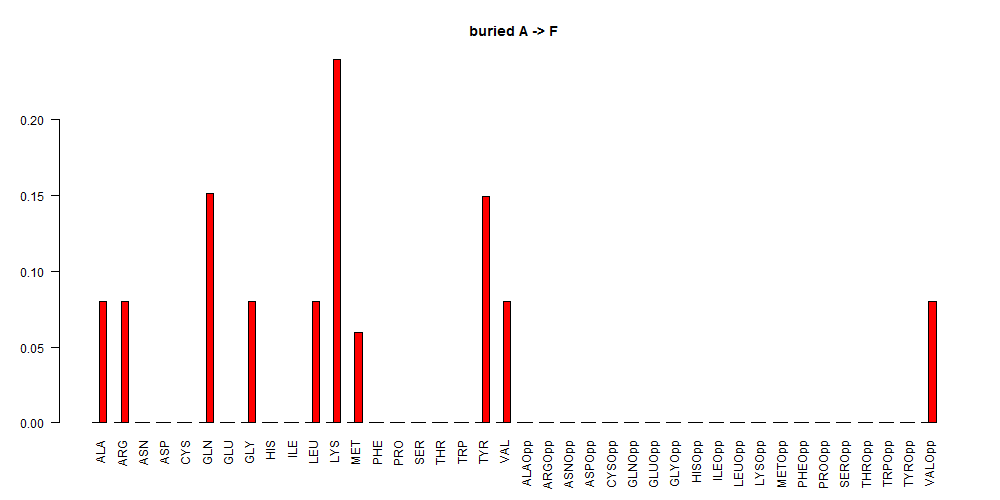

Supplement: Dataset S3 — Neighbouring residue profiles for mutations classed by substitution. (ZIP) [file pone.0084598.s003.zip › neighbour_2/buried_A_F.tif]

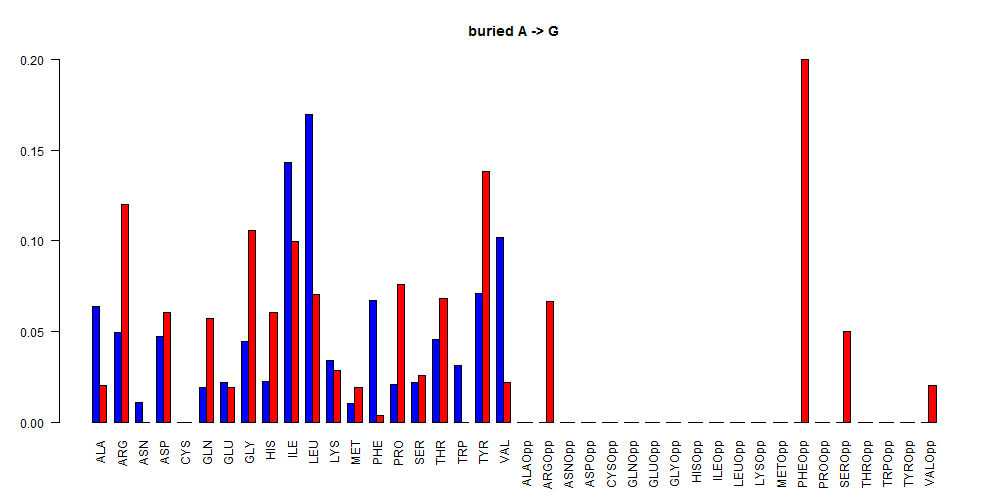

Supplement: Dataset S3 — Neighbouring residue profiles for mutations classed by substitution. (ZIP) [file pone.0084598.s003.zip › neighbour_2/buried_A_G.tif]

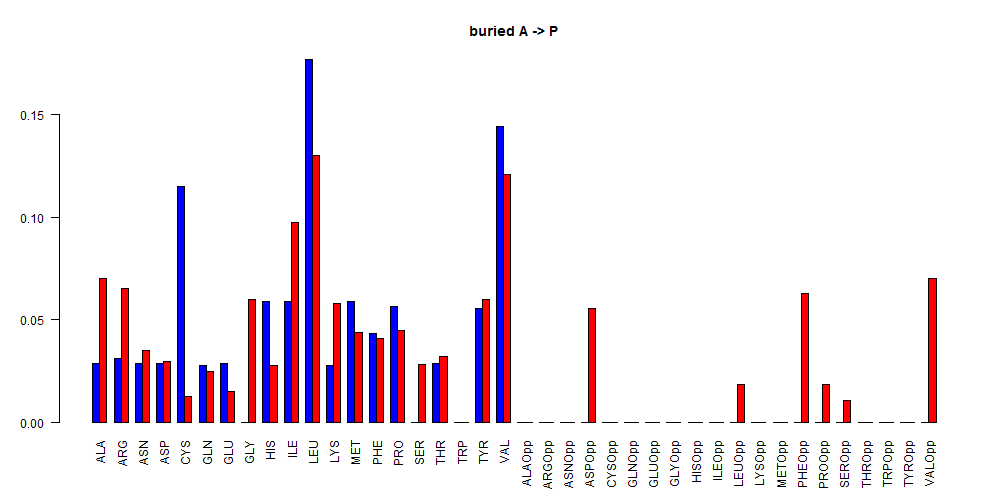

Supplement: Dataset S3 — Neighbouring residue profiles for mutations classed by substitution. (ZIP) [file pone.0084598.s003.zip › neighbour_2/buried_A_P.tif]

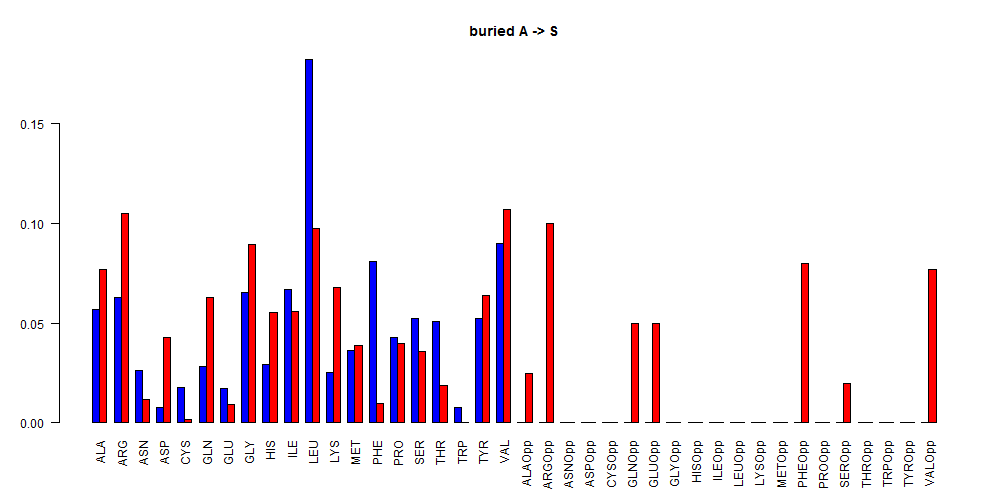

Supplement: Dataset S3 — Neighbouring residue profiles for mutations classed by substitution. (ZIP) [file pone.0084598.s003.zip › neighbour_2/buried_A_S.tif]

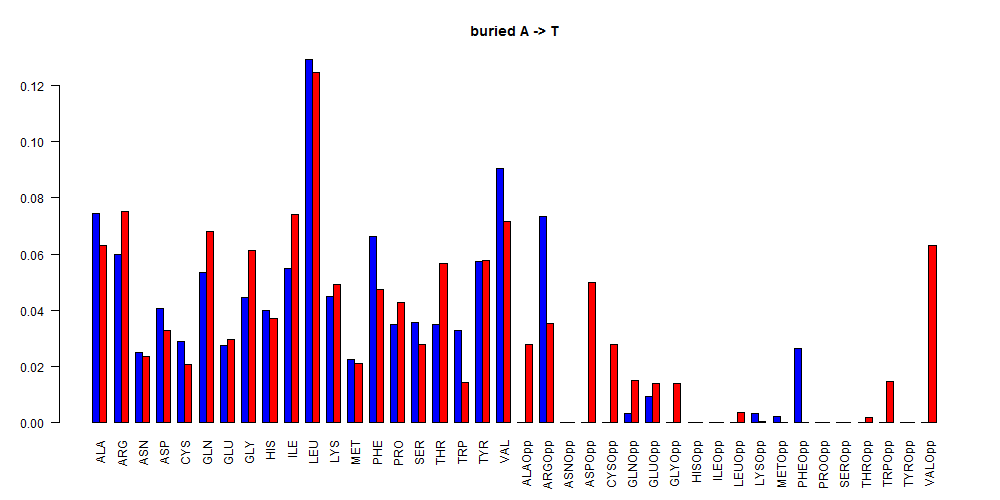

Supplement: Dataset S3 — Neighbouring residue profiles for mutations classed by substitution. (ZIP) [file pone.0084598.s003.zip › neighbour_2/buried_A_T.tif]

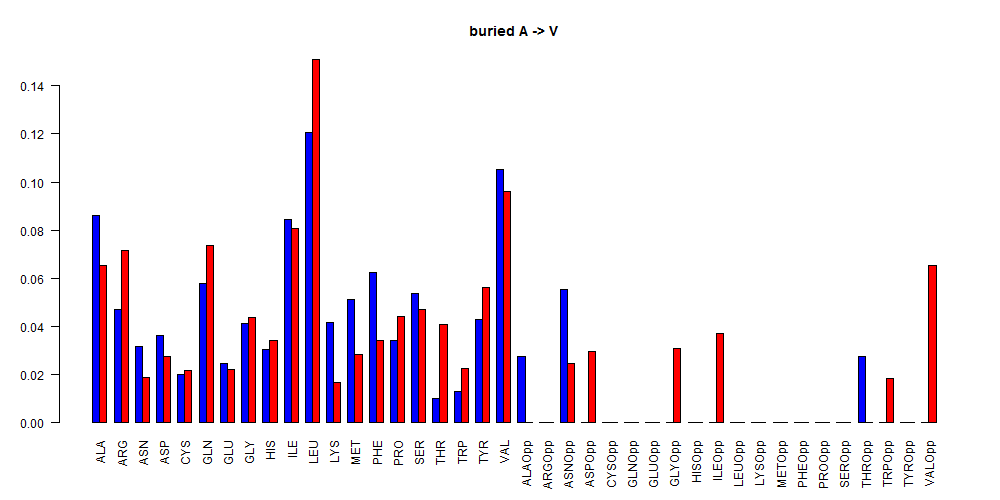

Supplement: Dataset S3 — Neighbouring residue profiles for mutations classed by substitution. (ZIP) [file pone.0084598.s003.zip › neighbour_2/buried_A_V.tif]

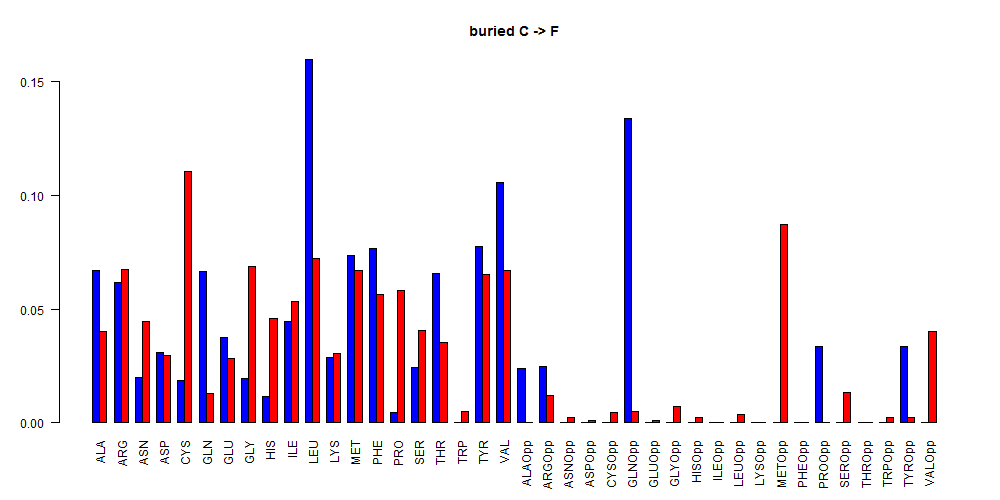

Supplement: Dataset S3 — Neighbouring residue profiles for mutations classed by substitution. (ZIP) [file pone.0084598.s003.zip › neighbour_2/buried_C_F.tif]

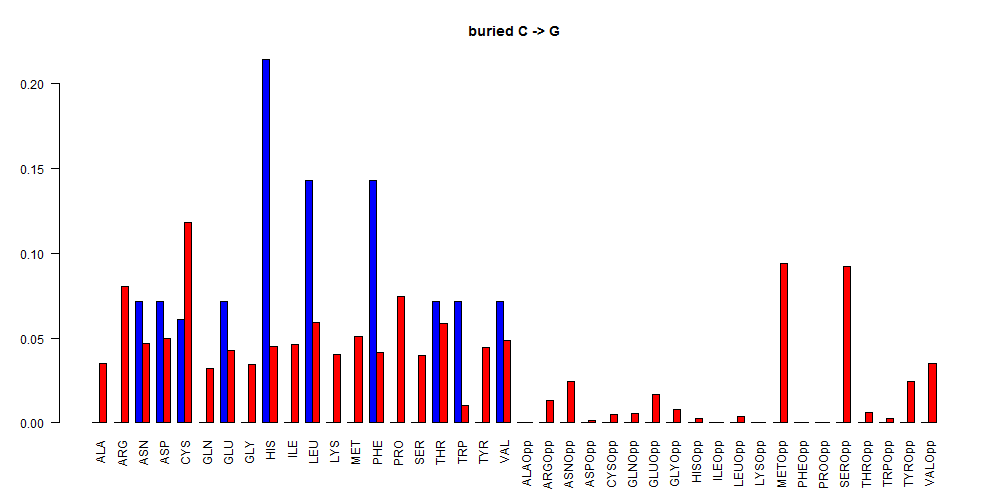

Supplement: Dataset S3 — Neighbouring residue profiles for mutations classed by substitution. (ZIP) [file pone.0084598.s003.zip › neighbour_2/buried_C_G.tif]

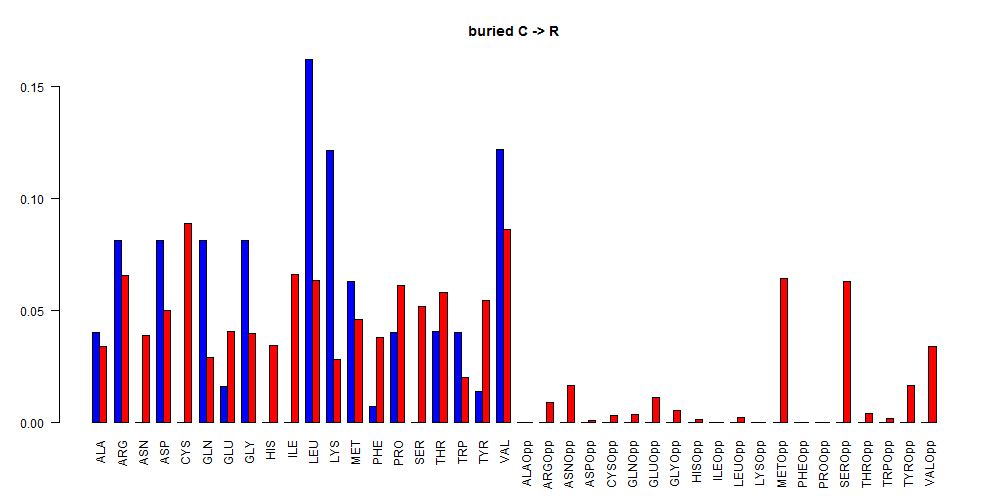

Supplement: Dataset S3 — Neighbouring residue profiles for mutations classed by substitution. (ZIP) [file pone.0084598.s003.zip › neighbour_2/buried_C_R.tif]

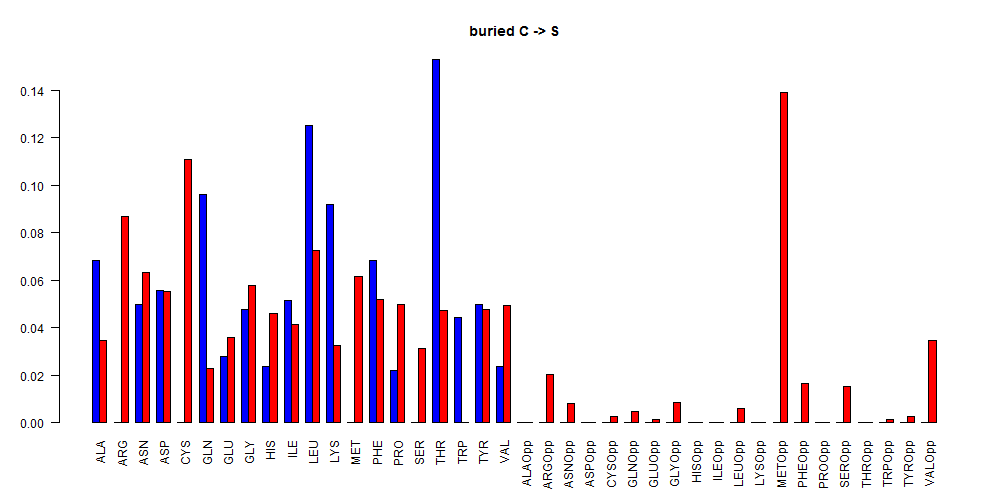

Supplement: Dataset S3 — Neighbouring residue profiles for mutations classed by substitution. (ZIP) [file pone.0084598.s003.zip › neighbour_2/buried_C_S.tif]

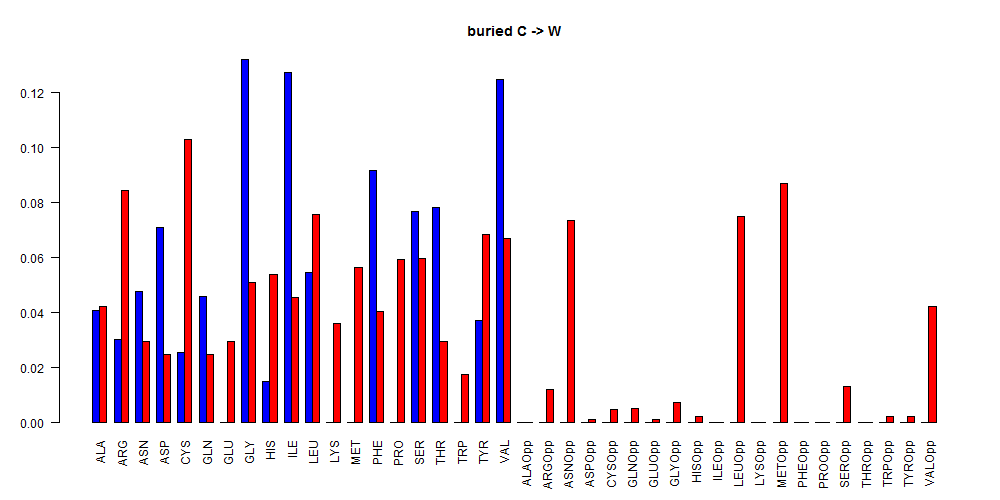

Supplement: Dataset S3 — Neighbouring residue profiles for mutations classed by substitution. (ZIP) [file pone.0084598.s003.zip › neighbour_2/buried_C_W.tif]

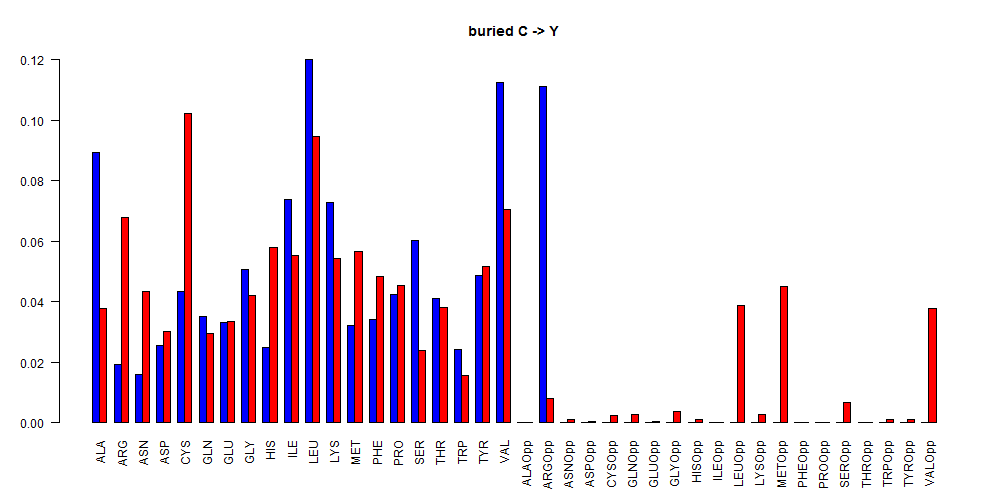

Supplement: Dataset S3 — Neighbouring residue profiles for mutations classed by substitution. (ZIP) [file pone.0084598.s003.zip › neighbour_2/buried_C_Y.tif]

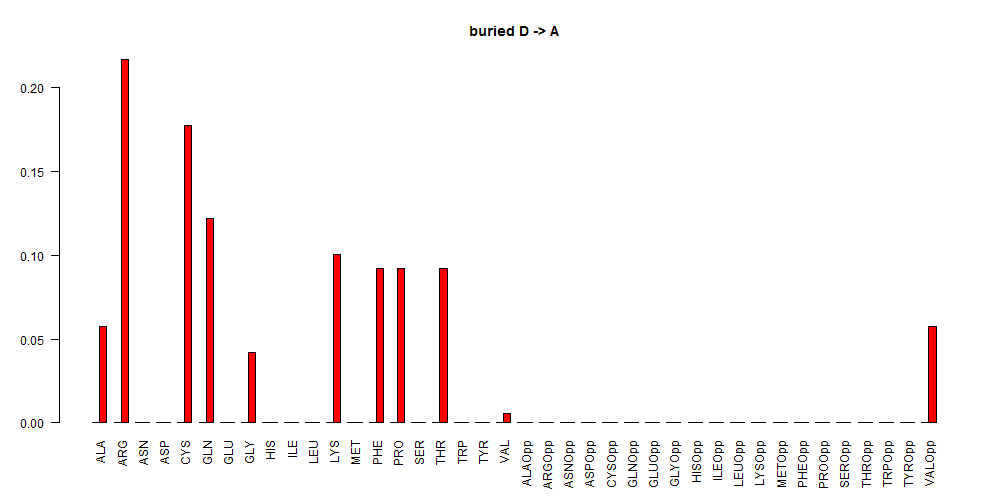

Supplement: Dataset S3 — Neighbouring residue profiles for mutations classed by substitution. (ZIP) [file pone.0084598.s003.zip › neighbour_2/buried_D_A.tif]

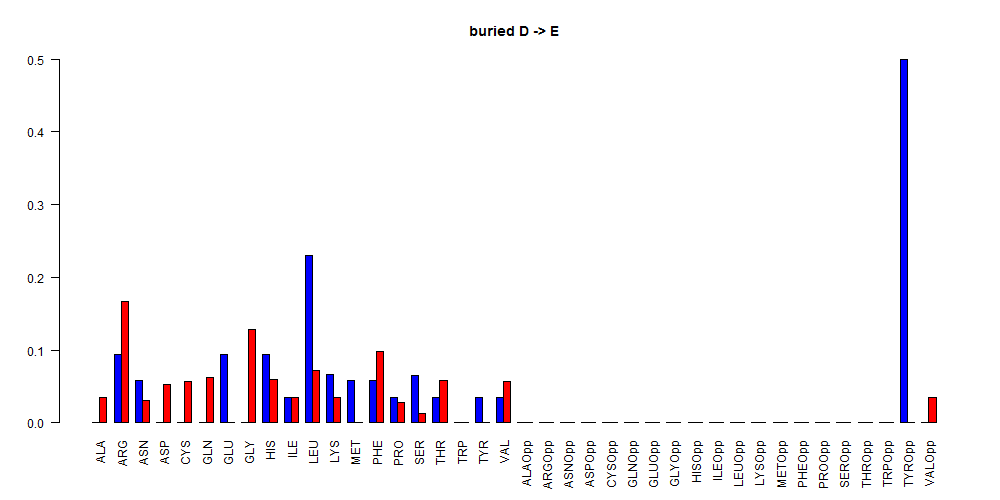

Supplement: Dataset S3 — Neighbouring residue profiles for mutations classed by substitution. (ZIP) [file pone.0084598.s003.zip › neighbour_2/buried_D_E.tif]

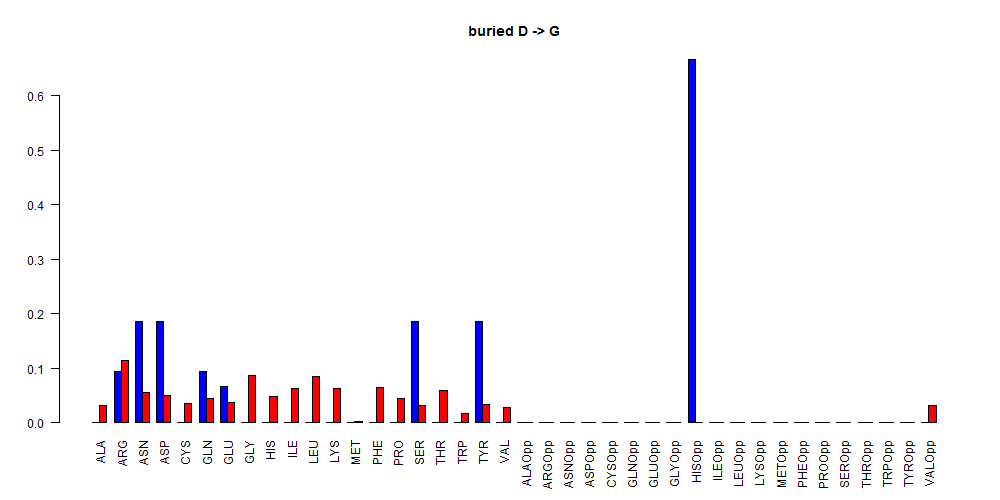

Supplement: Dataset S3 — Neighbouring residue profiles for mutations classed by substitution. (ZIP) [file pone.0084598.s003.zip › neighbour_2/buried_D_G.tif]

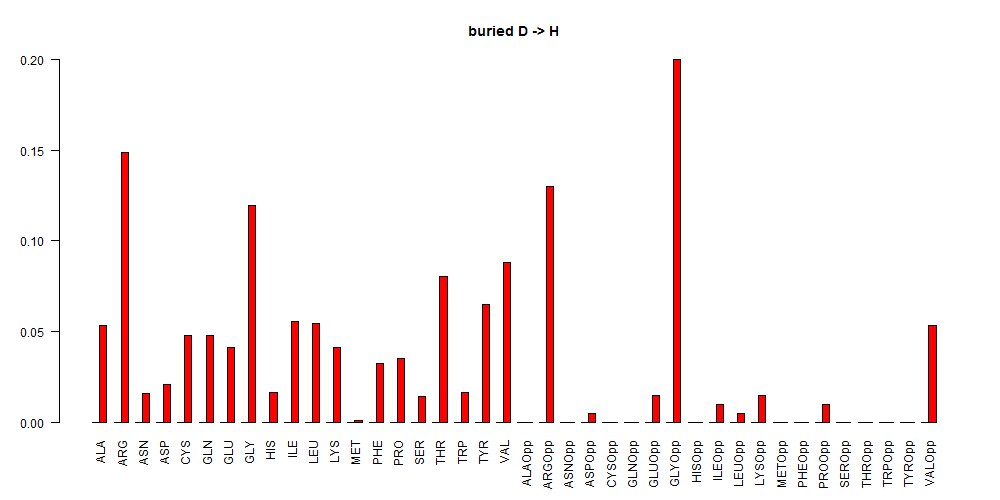

Supplement: Dataset S3 — Neighbouring residue profiles for mutations classed by substitution. (ZIP) [file pone.0084598.s003.zip › neighbour_2/buried_D_H.tif]

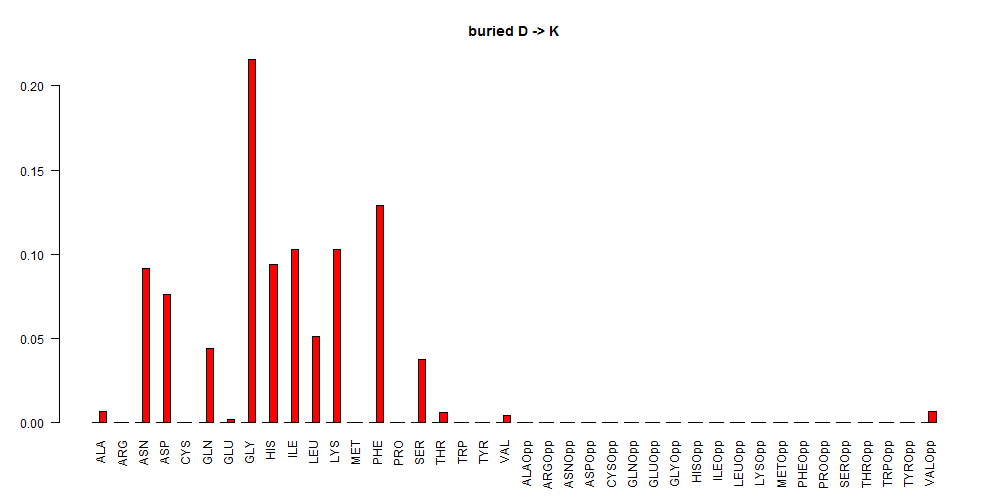

Supplement: Dataset S3 — Neighbouring residue profiles for mutations classed by substitution. (ZIP) [file pone.0084598.s003.zip › neighbour_2/buried_D_K.tif]

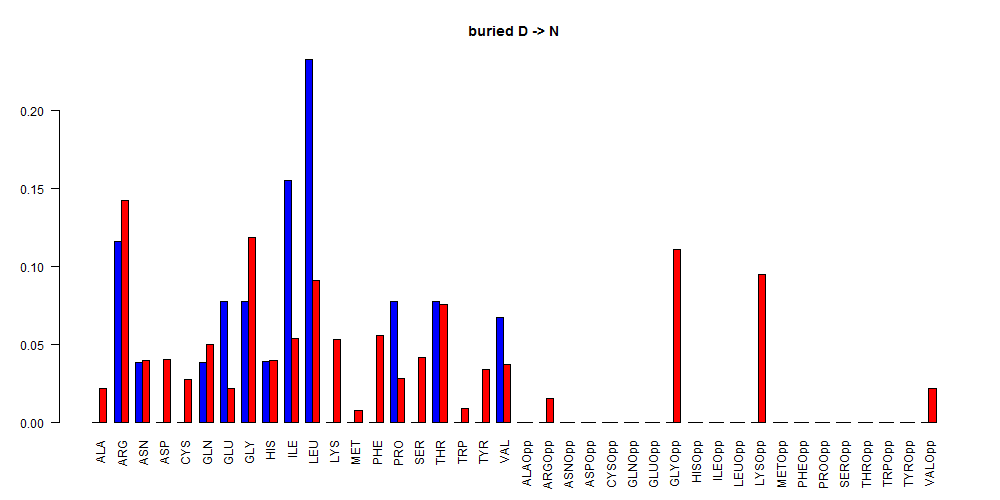

Supplement: Dataset S3 — Neighbouring residue profiles for mutations classed by substitution. (ZIP) [file pone.0084598.s003.zip › neighbour_2/buried_D_N.tif]

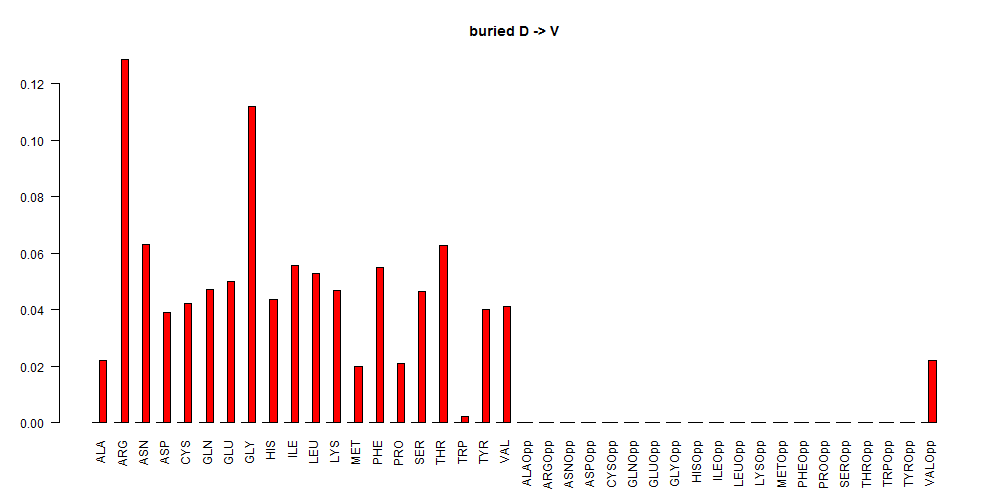

Supplement: Dataset S3 — Neighbouring residue profiles for mutations classed by substitution. (ZIP) [file pone.0084598.s003.zip › neighbour_2/buried_D_V.tif]

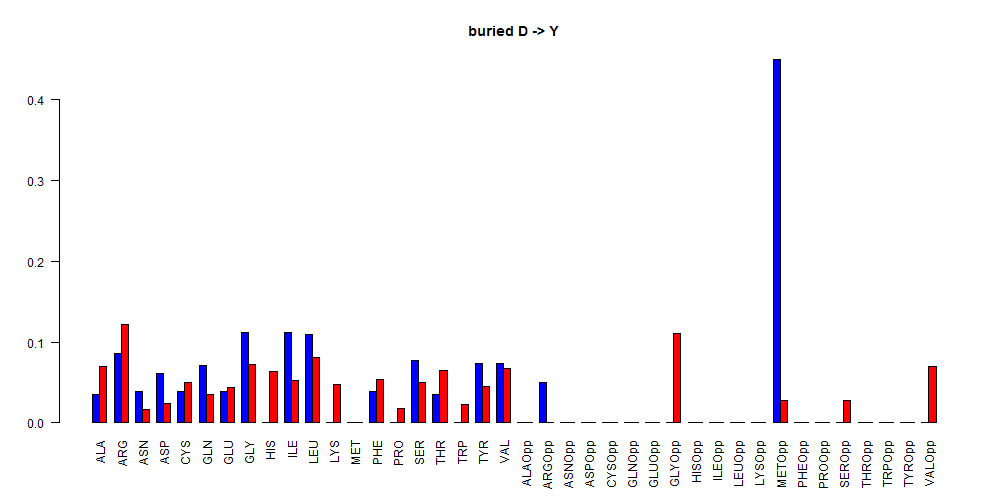

Supplement: Dataset S3 — Neighbouring residue profiles for mutations classed by substitution. (ZIP) [file pone.0084598.s003.zip › neighbour_2/buried_D_Y.tif]

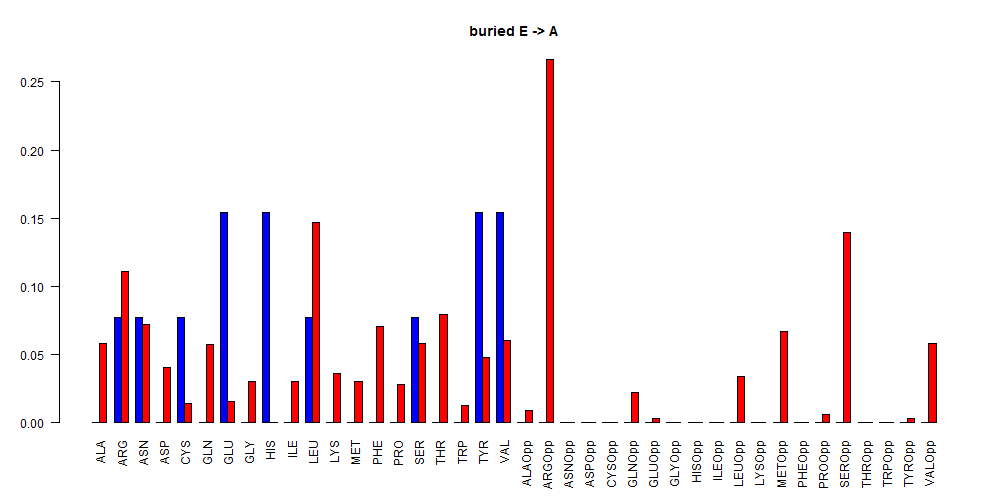

Supplement: Dataset S3 — Neighbouring residue profiles for mutations classed by substitution. (ZIP) [file pone.0084598.s003.zip › neighbour_2/buried_E_A.tif]

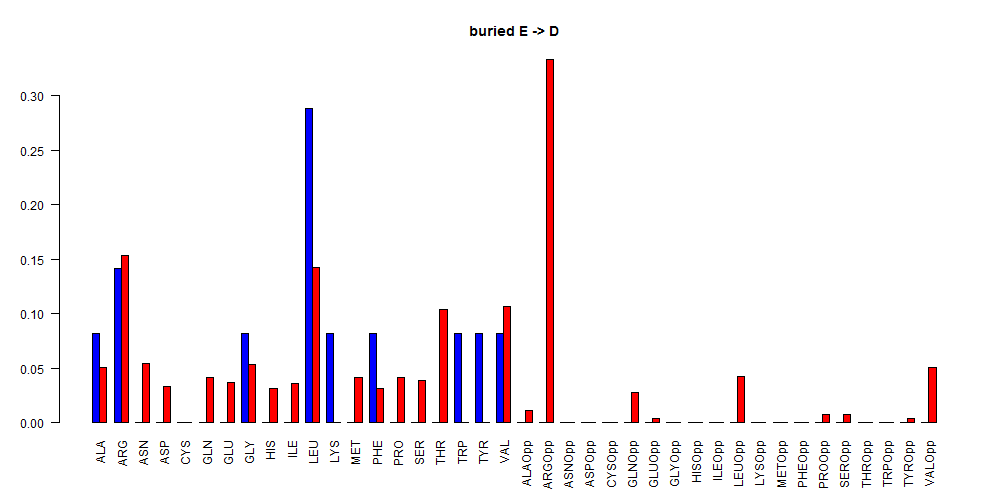

Supplement: Dataset S3 — Neighbouring residue profiles for mutations classed by substitution. (ZIP) [file pone.0084598.s003.zip › neighbour_2/buried_E_D.tif]

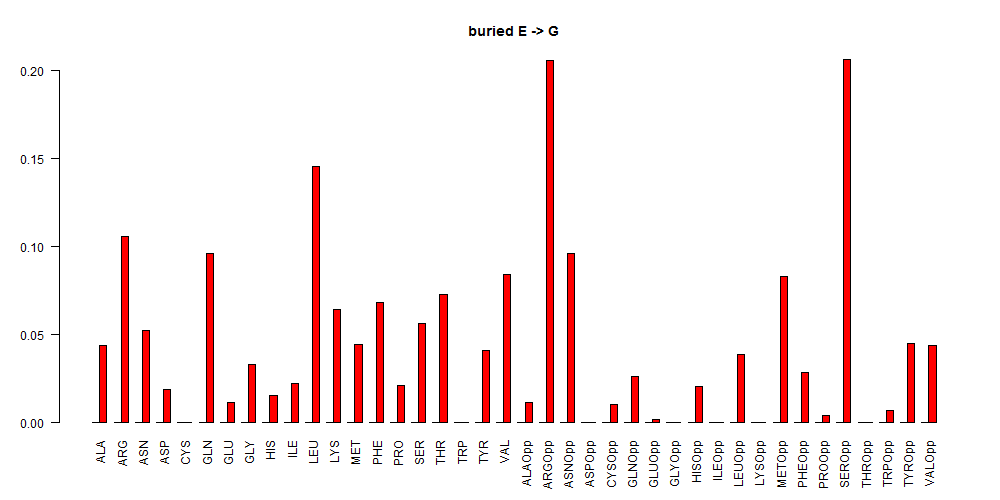

Supplement: Dataset S3 — Neighbouring residue profiles for mutations classed by substitution. (ZIP) [file pone.0084598.s003.zip › neighbour_2/buried_E_G.tif]

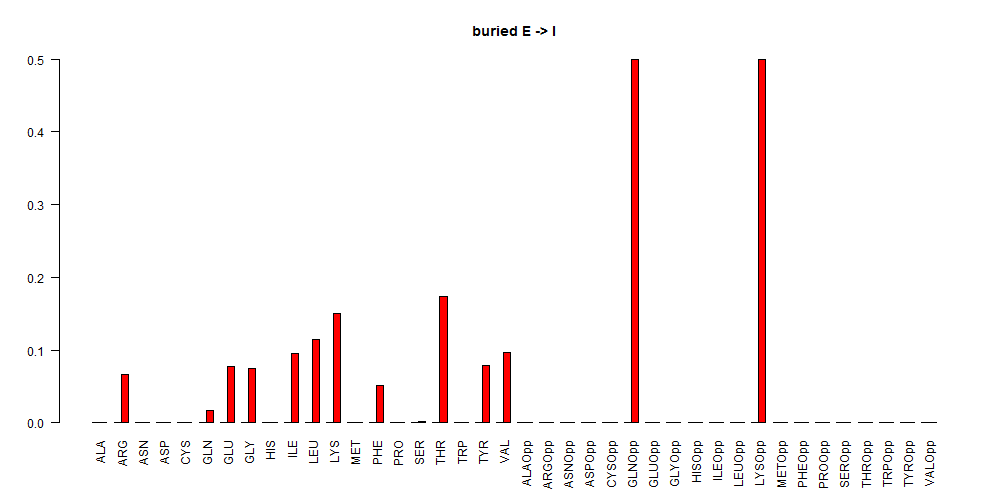

Supplement: Dataset S3 — Neighbouring residue profiles for mutations classed by substitution. (ZIP) [file pone.0084598.s003.zip › neighbour_2/buried_E_I.tif]

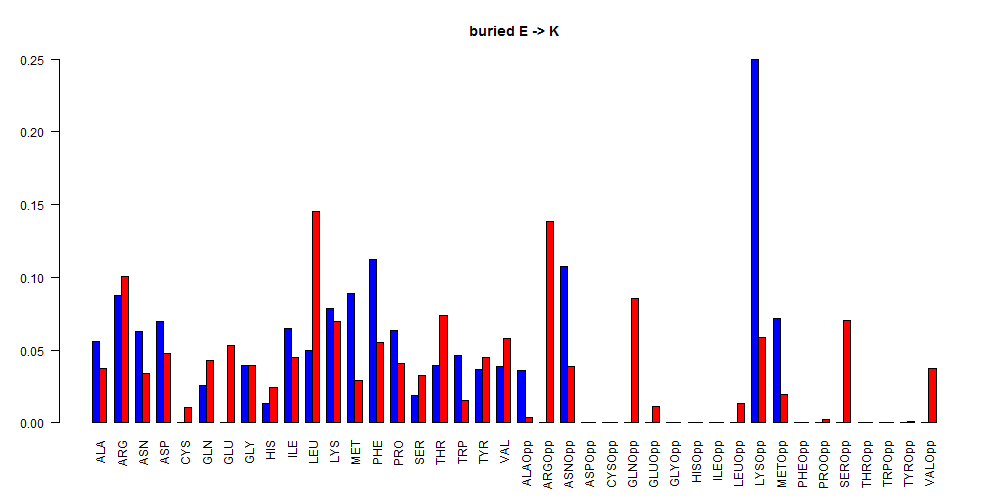

Supplement: Dataset S3 — Neighbouring residue profiles for mutations classed by substitution. (ZIP) [file pone.0084598.s003.zip › neighbour_2/buried_E_K.tif]

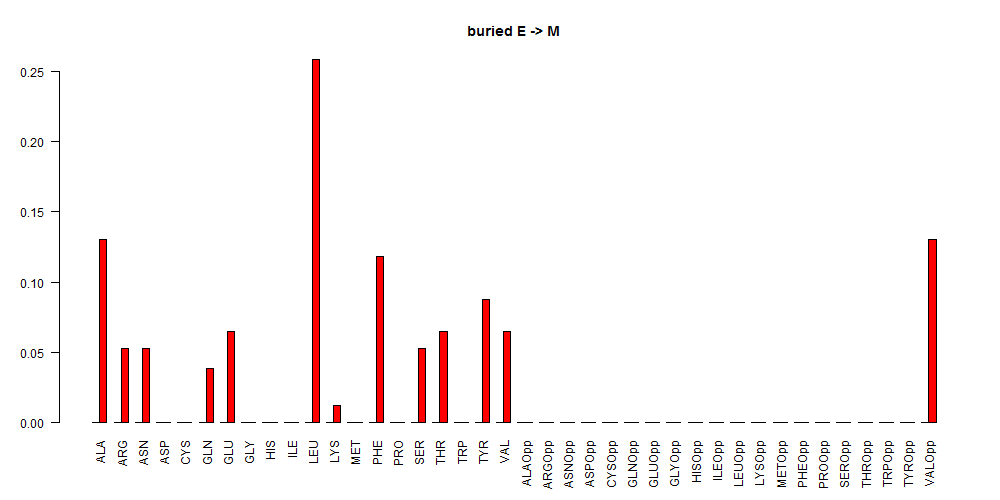

Supplement: Dataset S3 — Neighbouring residue profiles for mutations classed by substitution. (ZIP) [file pone.0084598.s003.zip › neighbour_2/buried_E_M.tif]

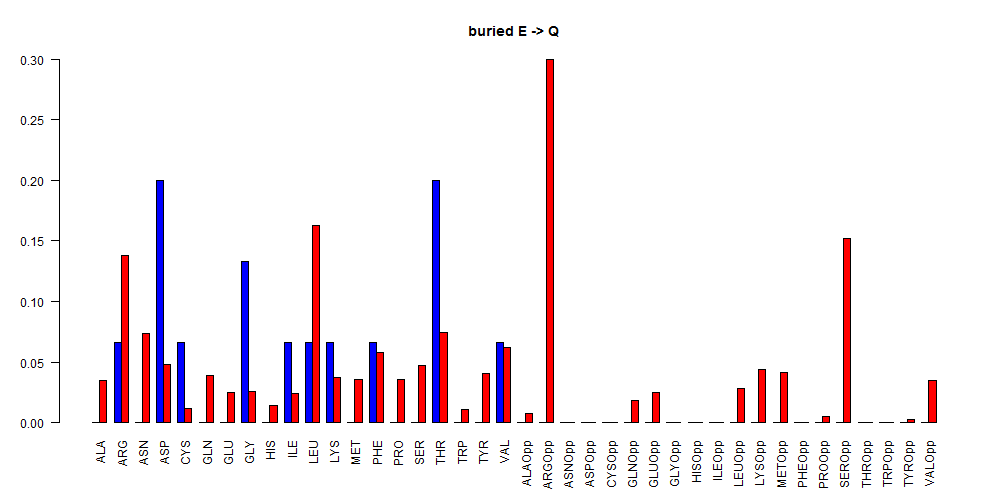

Supplement: Dataset S3 — Neighbouring residue profiles for mutations classed by substitution. (ZIP) [file pone.0084598.s003.zip › neighbour_2/buried_E_Q.tif]

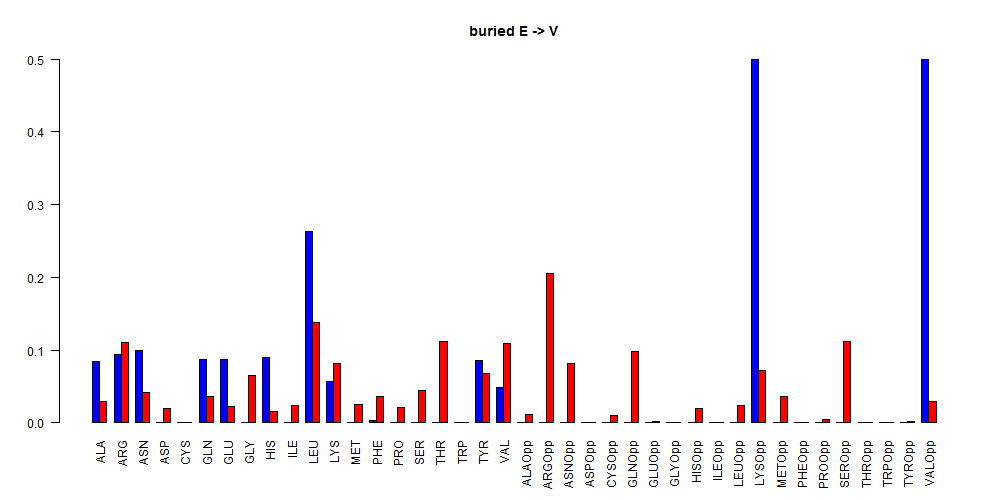

Supplement: Dataset S3 — Neighbouring residue profiles for mutations classed by substitution. (ZIP) [file pone.0084598.s003.zip › neighbour_2/buried_E_V.tif]

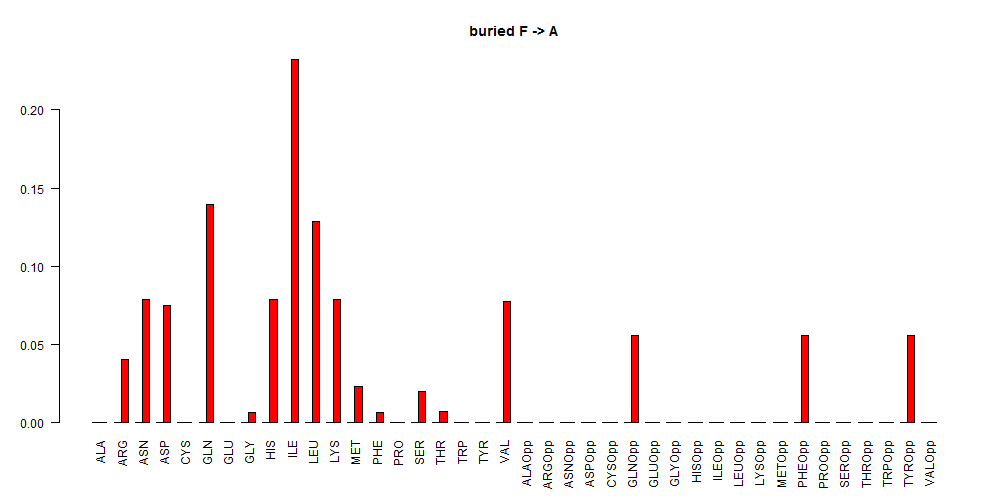

Supplement: Dataset S3 — Neighbouring residue profiles for mutations classed by substitution. (ZIP) [file pone.0084598.s003.zip › neighbour_2/buried_F_A.tif]

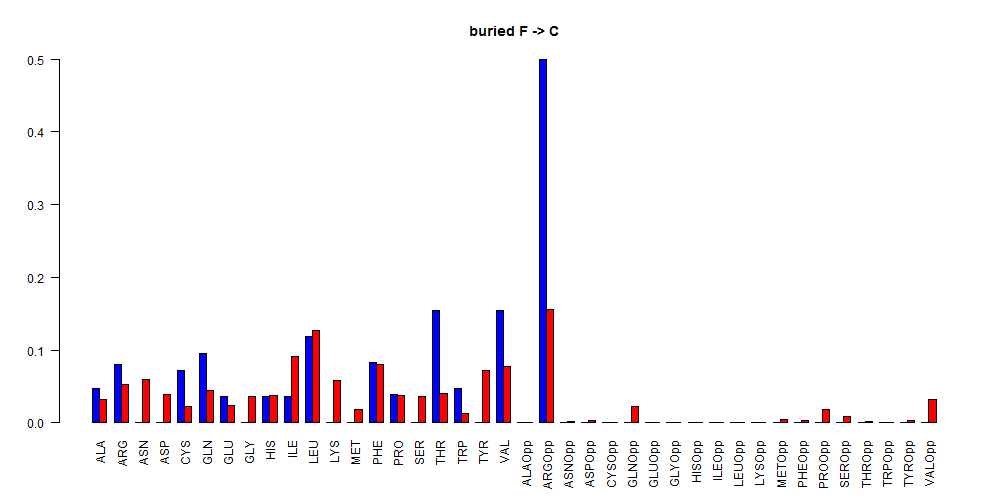

Supplement: Dataset S3 — Neighbouring residue profiles for mutations classed by substitution. (ZIP) [file pone.0084598.s003.zip › neighbour_2/buried_F_C.tif]

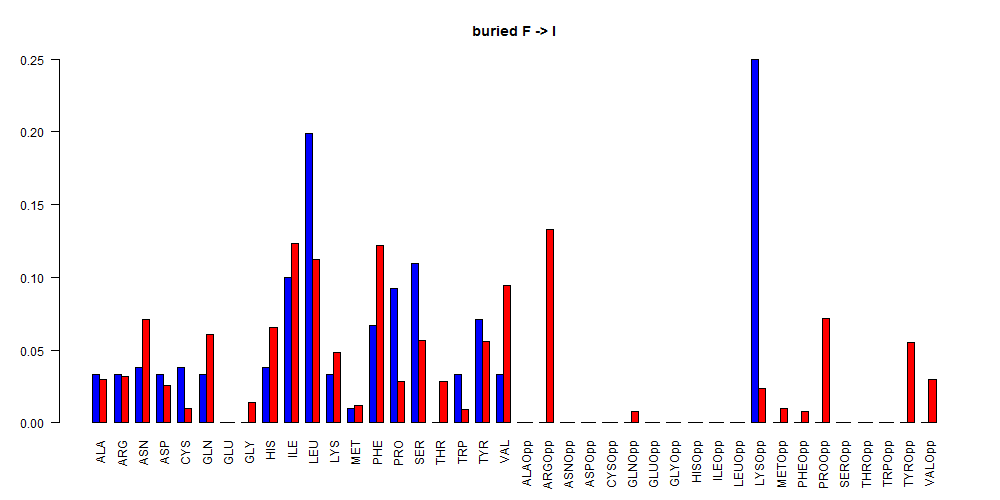

Supplement: Dataset S3 — Neighbouring residue profiles for mutations classed by substitution. (ZIP) [file pone.0084598.s003.zip › neighbour_2/buried_F_I.tif]

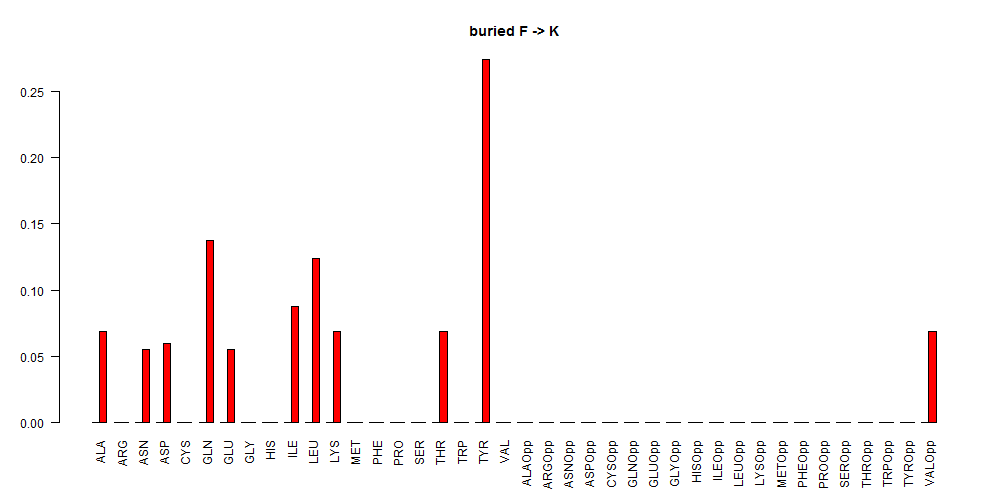

Supplement: Dataset S3 — Neighbouring residue profiles for mutations classed by substitution. (ZIP) [file pone.0084598.s003.zip › neighbour_2/buried_F_K.tif]

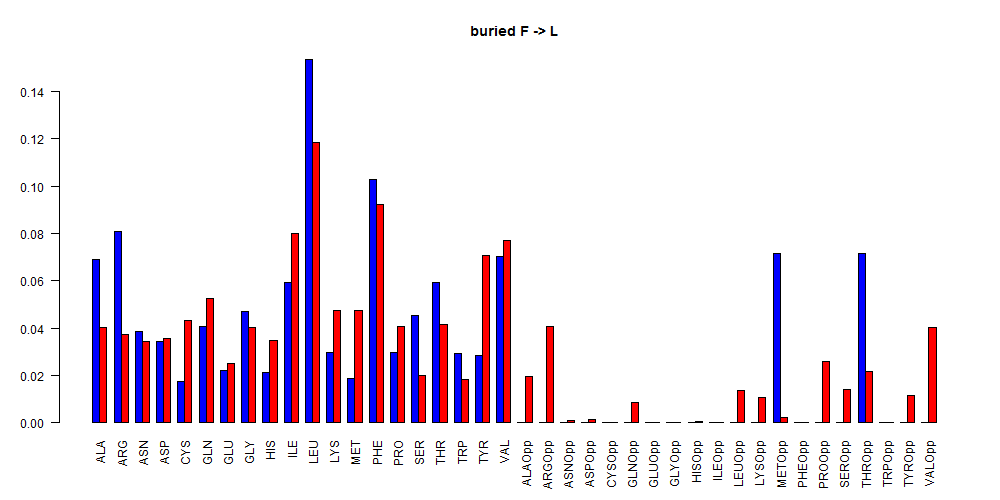

Supplement: Dataset S3 — Neighbouring residue profiles for mutations classed by substitution. (ZIP) [file pone.0084598.s003.zip › neighbour_2/buried_F_L.tif]

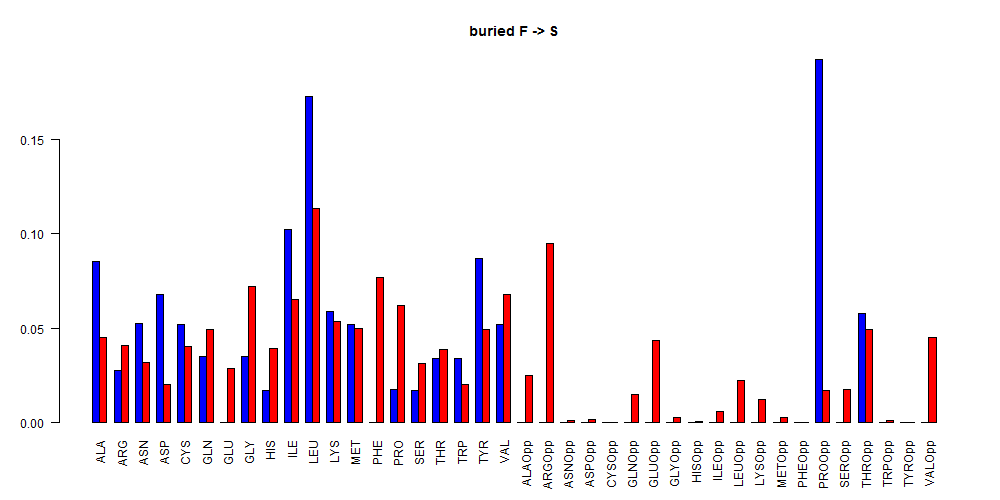

Supplement: Dataset S3 — Neighbouring residue profiles for mutations classed by substitution. (ZIP) [file pone.0084598.s003.zip › neighbour_2/buried_F_S.tif]

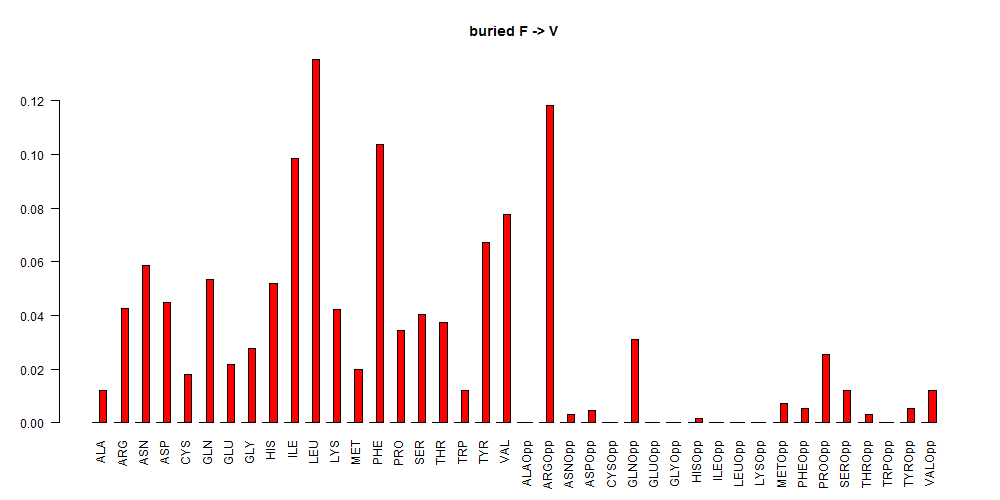

Supplement: Dataset S3 — Neighbouring residue profiles for mutations classed by substitution. (ZIP) [file pone.0084598.s003.zip › neighbour_2/buried_F_V.tif]

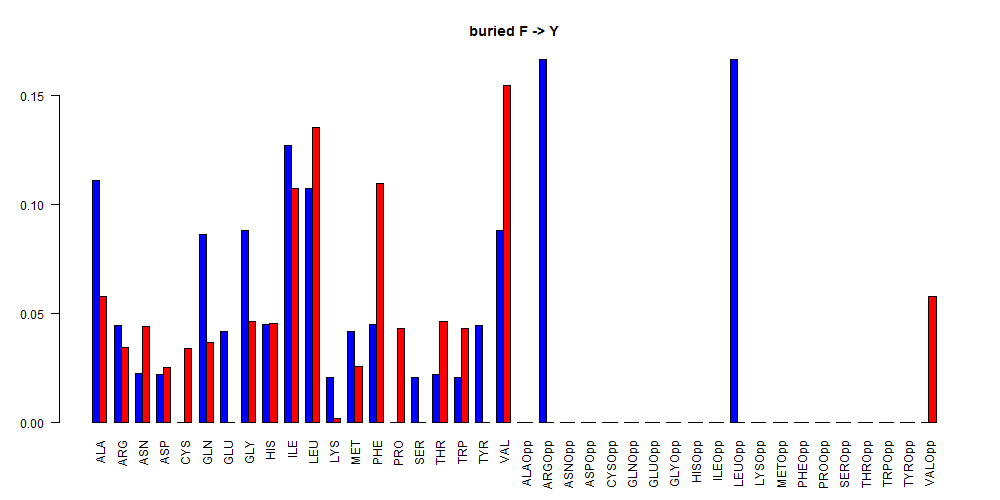

Supplement: Dataset S3 — Neighbouring residue profiles for mutations classed by substitution. (ZIP) [file pone.0084598.s003.zip › neighbour_2/buried_F_Y.tif]

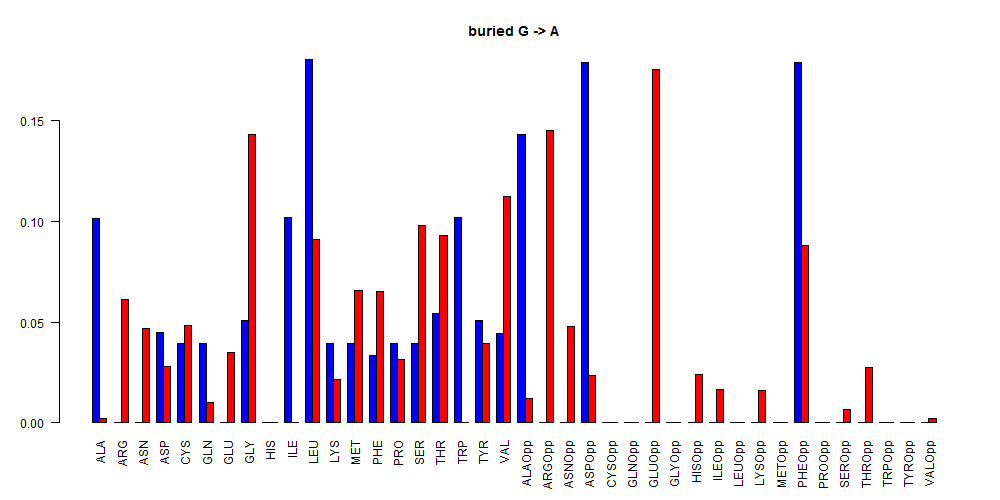

Supplement: Dataset S3 — Neighbouring residue profiles for mutations classed by substitution. (ZIP) [file pone.0084598.s003.zip › neighbour_2/buried_G_A.tif]

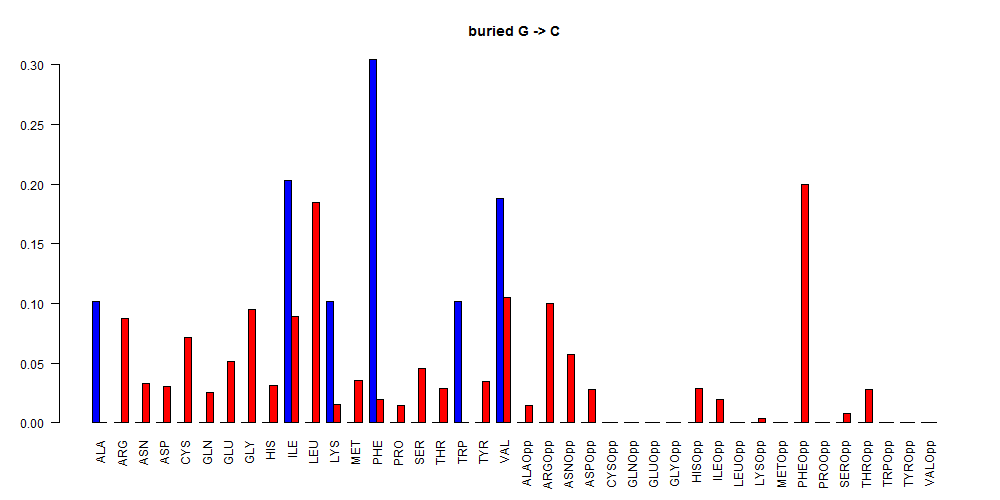

Supplement: Dataset S3 — Neighbouring residue profiles for mutations classed by substitution. (ZIP) [file pone.0084598.s003.zip › neighbour_2/buried_G_C.tif]
