# Supplementary figures and images for: Deriving a Mutation Index of Carcinogenicity Using Protein Structure and Protein Interfaces (part 3 of 3)
Source: PLoS One. 2014 Jan 15;9(1):e84598. doi: 10.1371/journal.pone.0084598 (PMC3893166; doi:10.1371/journal.pone.0084598)

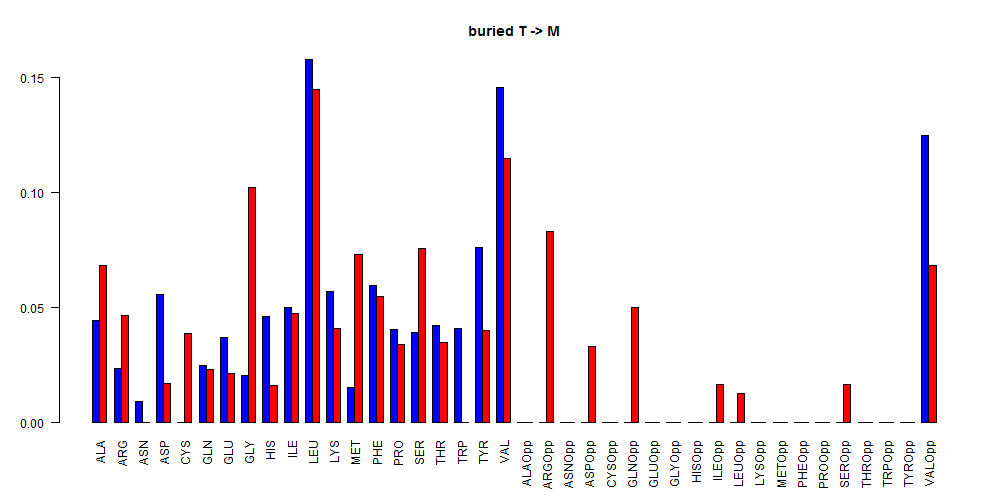

Supplement: Dataset S3 — Neighbouring residue profiles for mutations classed by substitution. (ZIP) [file pone.0084598.s003.zip › neighbour_2/buried_T_M.tif]

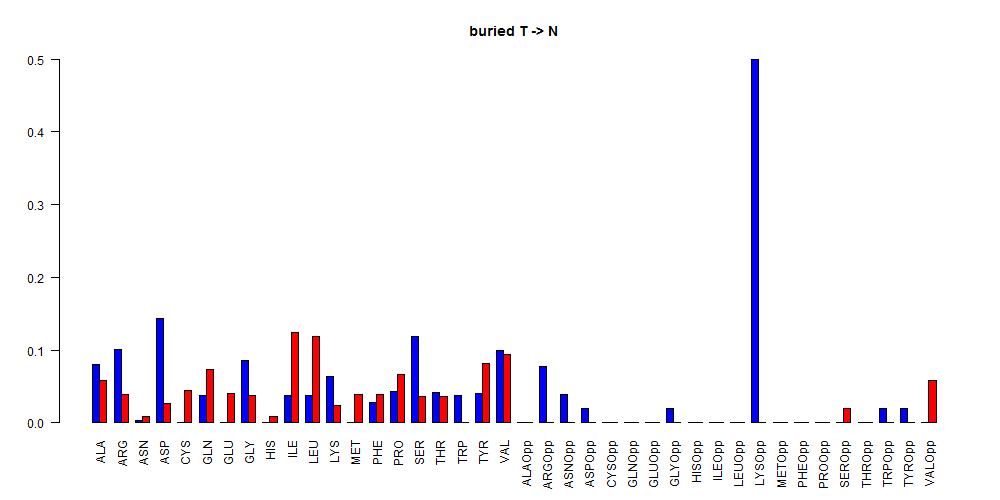

Supplement: Dataset S3 — Neighbouring residue profiles for mutations classed by substitution. (ZIP) [file pone.0084598.s003.zip › neighbour_2/buried_T_N.tif]

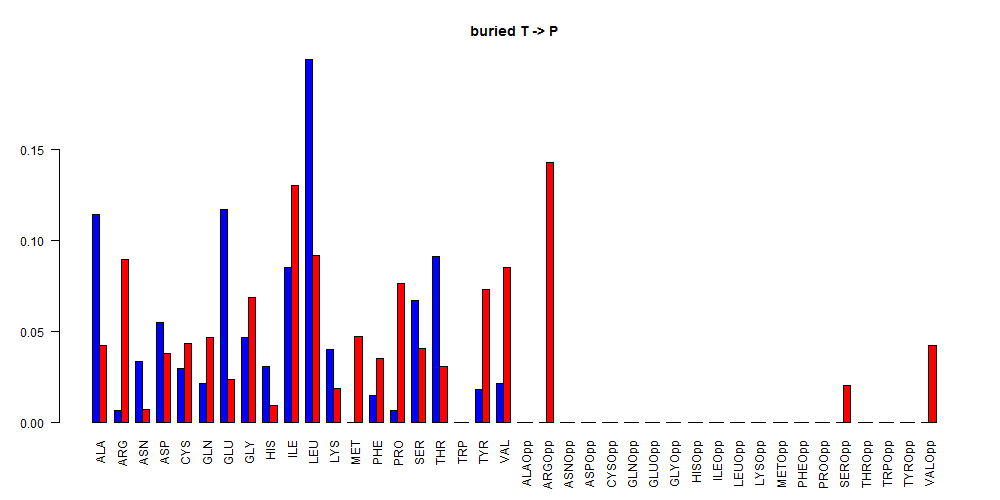

Supplement: Dataset S3 — Neighbouring residue profiles for mutations classed by substitution. (ZIP) [file pone.0084598.s003.zip › neighbour_2/buried_T_P.tif]

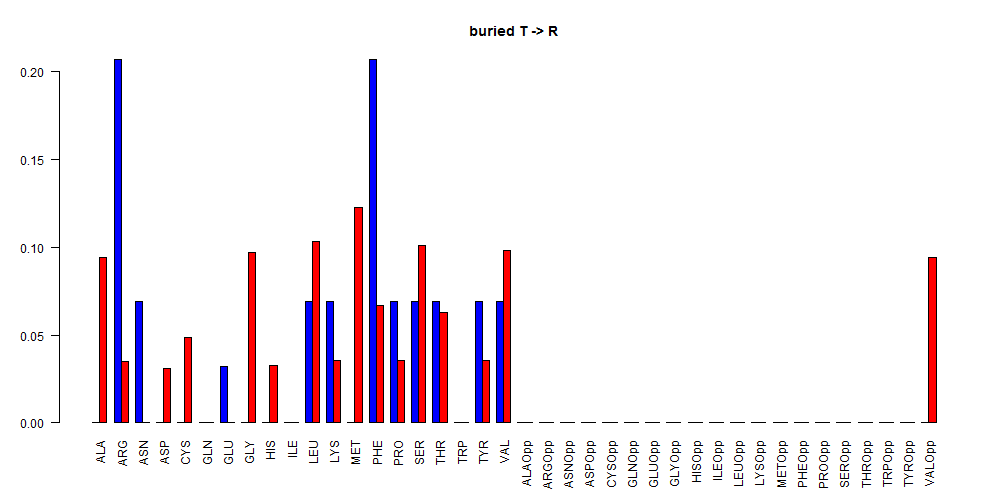

Supplement: Dataset S3 — Neighbouring residue profiles for mutations classed by substitution. (ZIP) [file pone.0084598.s003.zip › neighbour_2/buried_T_R.tif]

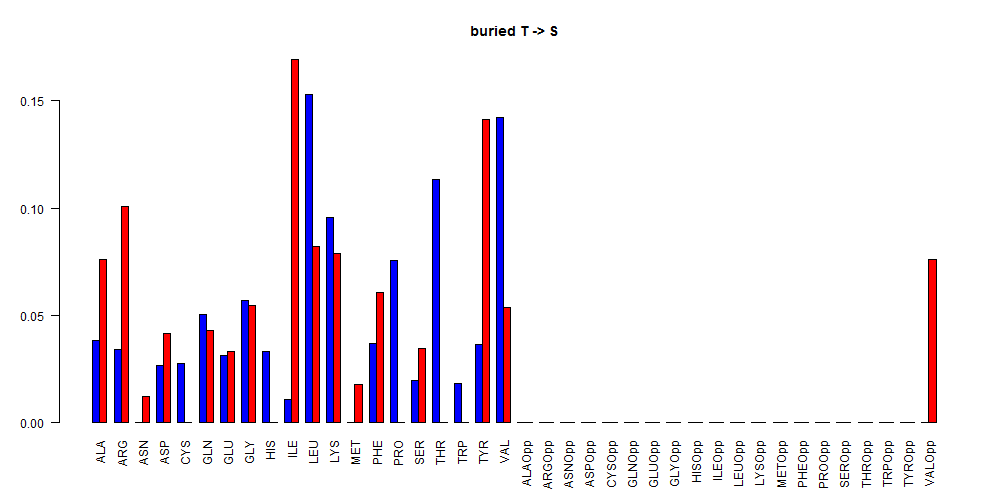

Supplement: Dataset S3 — Neighbouring residue profiles for mutations classed by substitution. (ZIP) [file pone.0084598.s003.zip › neighbour_2/buried_T_S.tif]

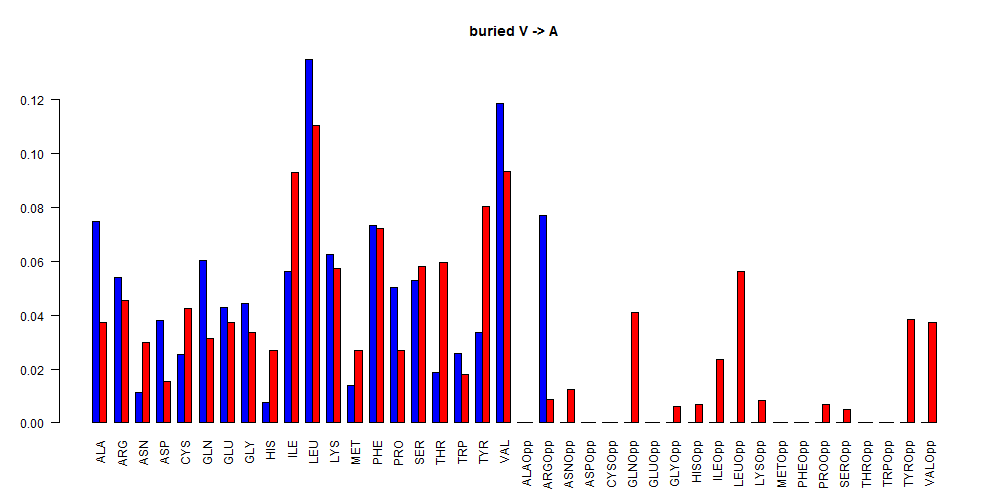

Supplement: Dataset S3 — Neighbouring residue profiles for mutations classed by substitution. (ZIP) [file pone.0084598.s003.zip › neighbour_2/buried_V_A.tif]

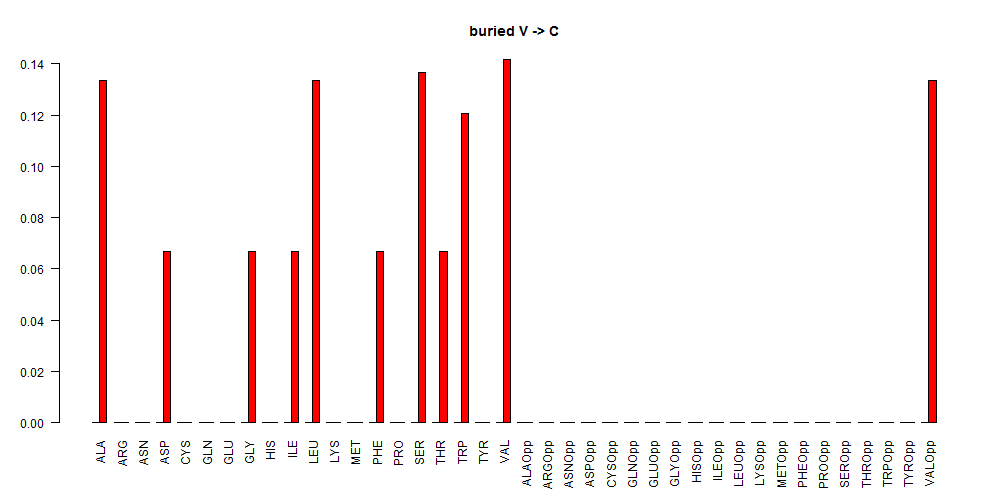

Supplement: Dataset S3 — Neighbouring residue profiles for mutations classed by substitution. (ZIP) [file pone.0084598.s003.zip › neighbour_2/buried_V_C.tif]

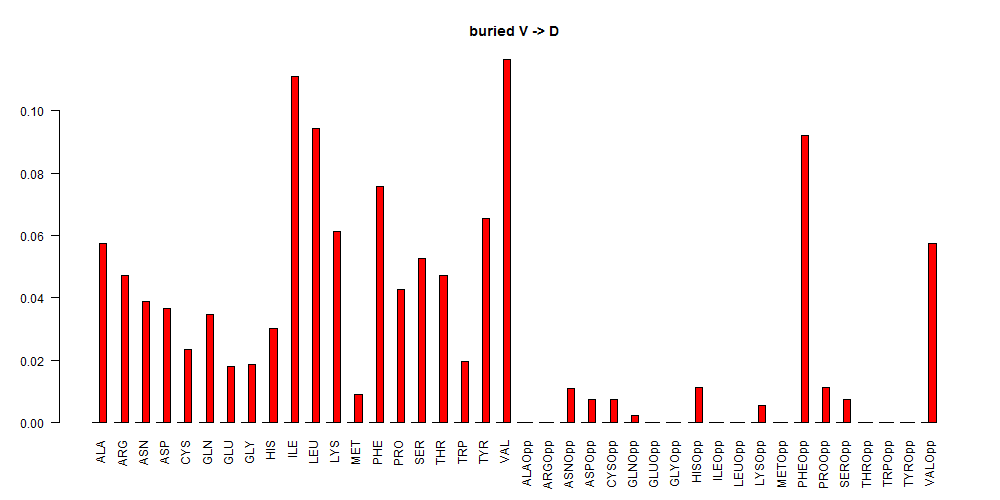

Supplement: Dataset S3 — Neighbouring residue profiles for mutations classed by substitution. (ZIP) [file pone.0084598.s003.zip › neighbour_2/buried_V_D.tif]

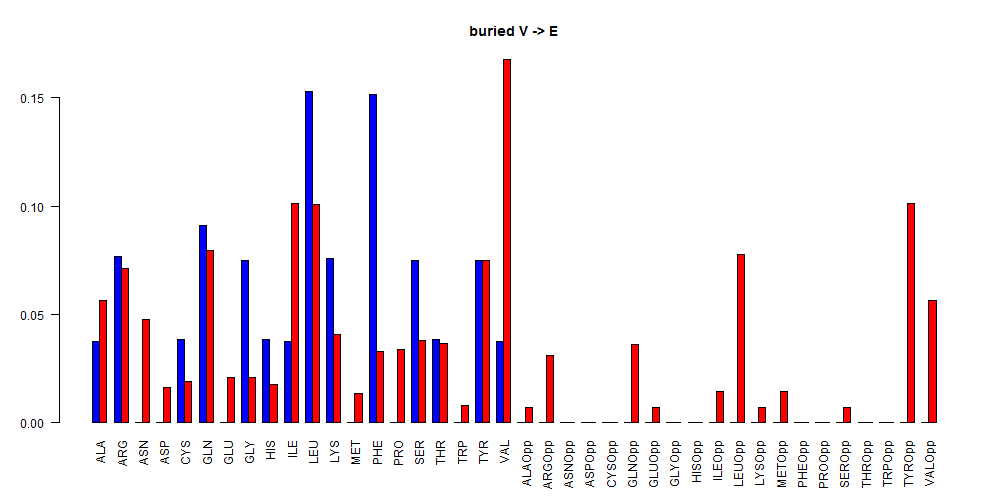

Supplement: Dataset S3 — Neighbouring residue profiles for mutations classed by substitution. (ZIP) [file pone.0084598.s003.zip › neighbour_2/buried_V_E.tif]

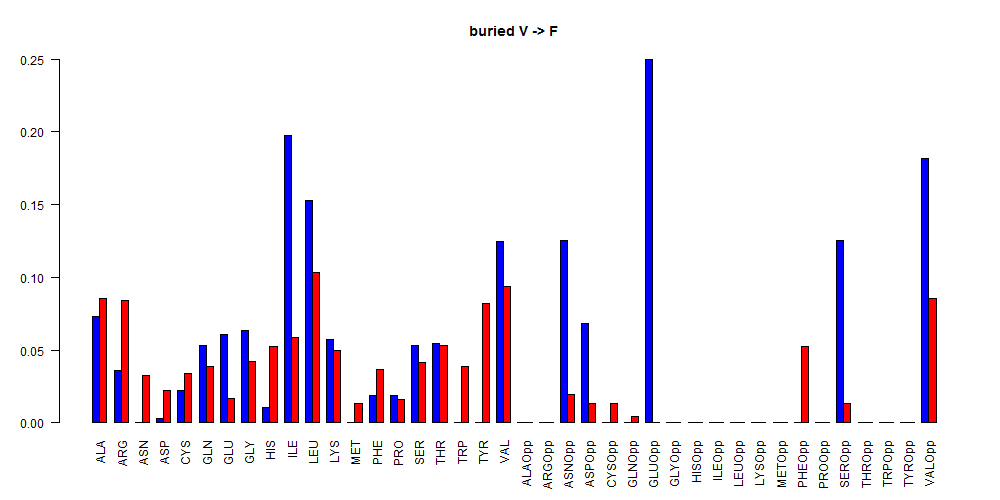

Supplement: Dataset S3 — Neighbouring residue profiles for mutations classed by substitution. (ZIP) [file pone.0084598.s003.zip › neighbour_2/buried_V_F.tif]

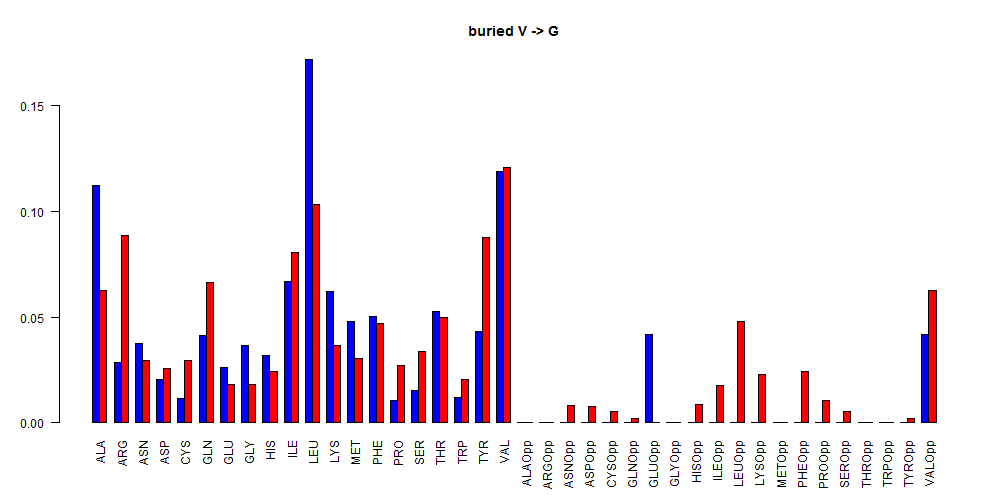

Supplement: Dataset S3 — Neighbouring residue profiles for mutations classed by substitution. (ZIP) [file pone.0084598.s003.zip › neighbour_2/buried_V_G.tif]

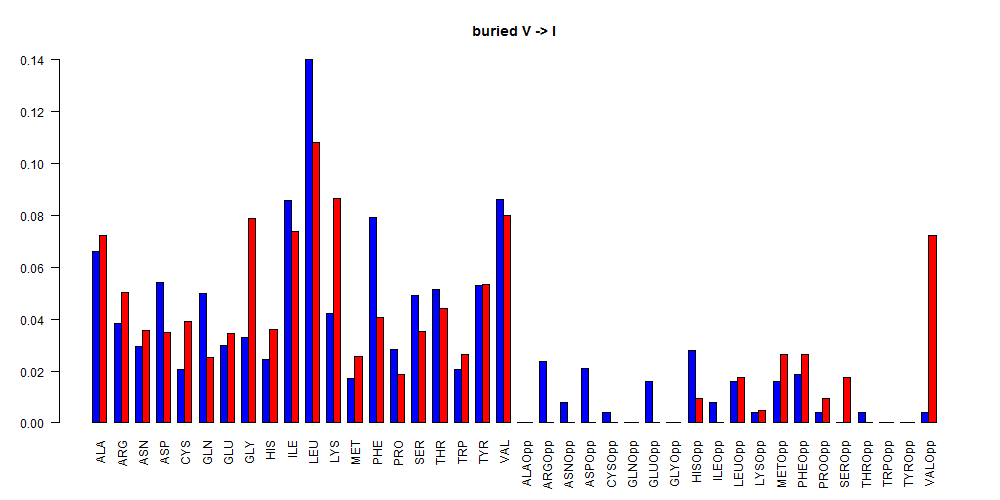

Supplement: Dataset S3 — Neighbouring residue profiles for mutations classed by substitution. (ZIP) [file pone.0084598.s003.zip › neighbour_2/buried_V_I.tif]

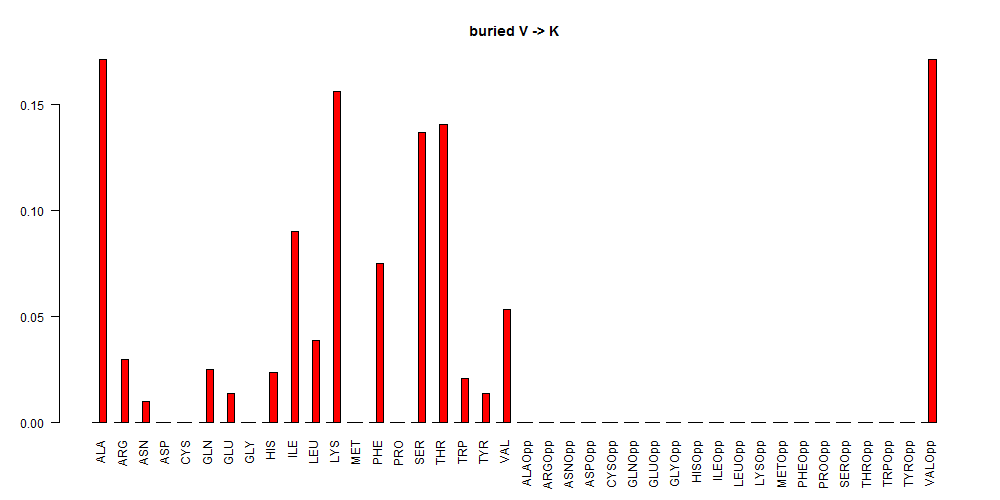

Supplement: Dataset S3 — Neighbouring residue profiles for mutations classed by substitution. (ZIP) [file pone.0084598.s003.zip › neighbour_2/buried_V_K.tif]

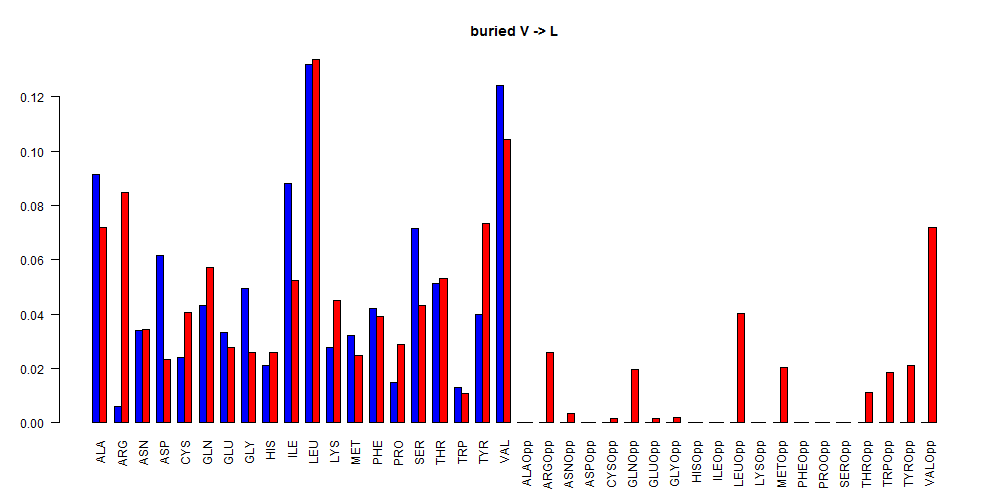

Supplement: Dataset S3 — Neighbouring residue profiles for mutations classed by substitution. (ZIP) [file pone.0084598.s003.zip › neighbour_2/buried_V_L.tif]

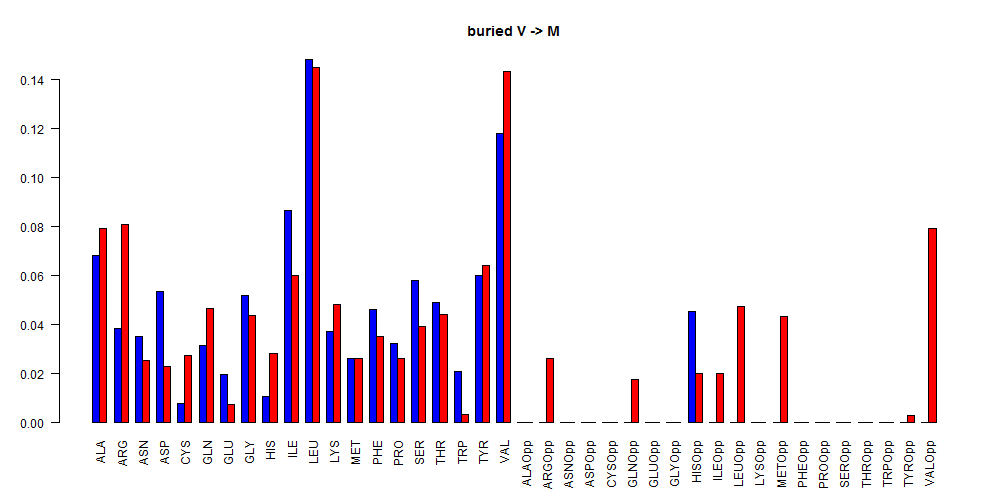

Supplement: Dataset S3 — Neighbouring residue profiles for mutations classed by substitution. (ZIP) [file pone.0084598.s003.zip › neighbour_2/buried_V_M.tif]

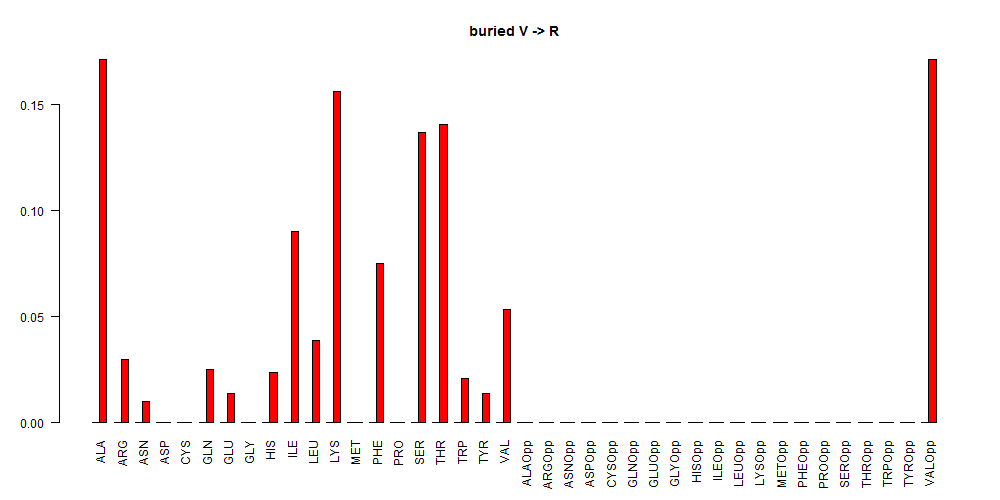

Supplement: Dataset S3 — Neighbouring residue profiles for mutations classed by substitution. (ZIP) [file pone.0084598.s003.zip › neighbour_2/buried_V_R.tif]

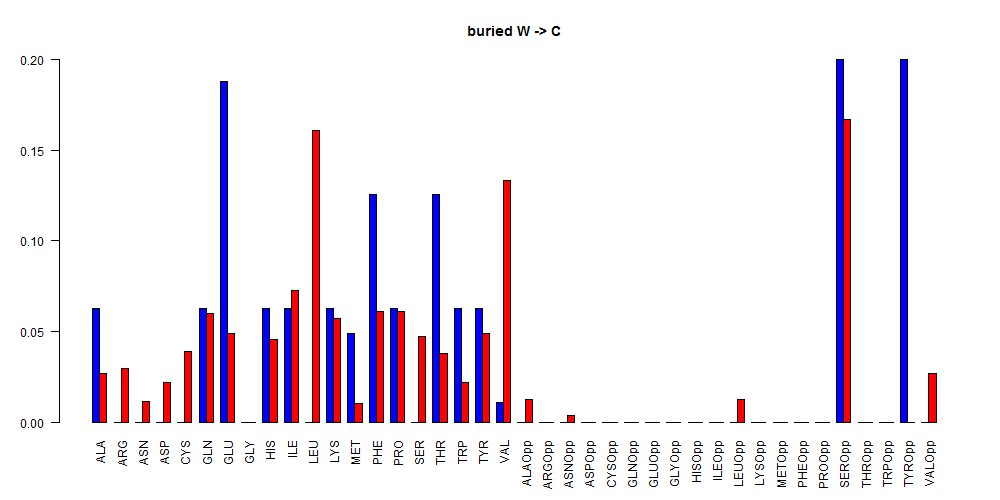

Supplement: Dataset S3 — Neighbouring residue profiles for mutations classed by substitution. (ZIP) [file pone.0084598.s003.zip › neighbour_2/buried_W_C.tif]

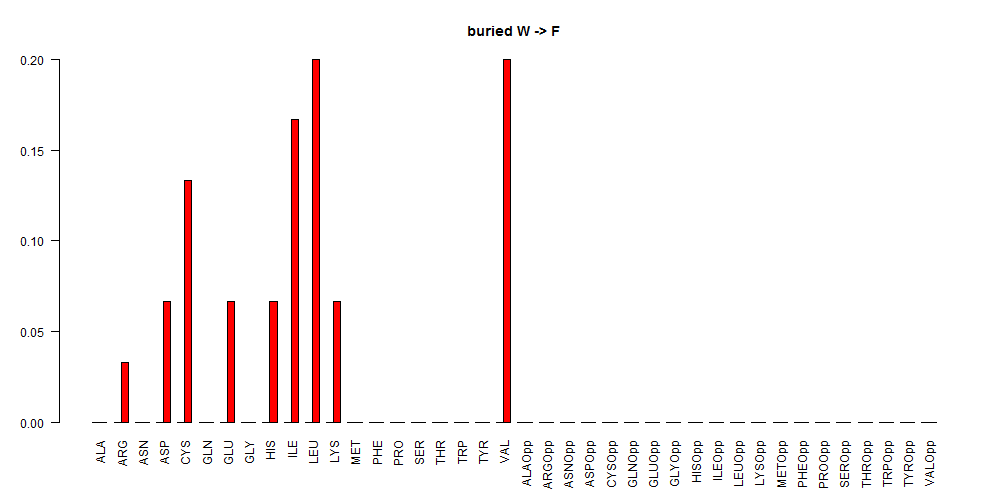

Supplement: Dataset S3 — Neighbouring residue profiles for mutations classed by substitution. (ZIP) [file pone.0084598.s003.zip › neighbour_2/buried_W_F.tif]

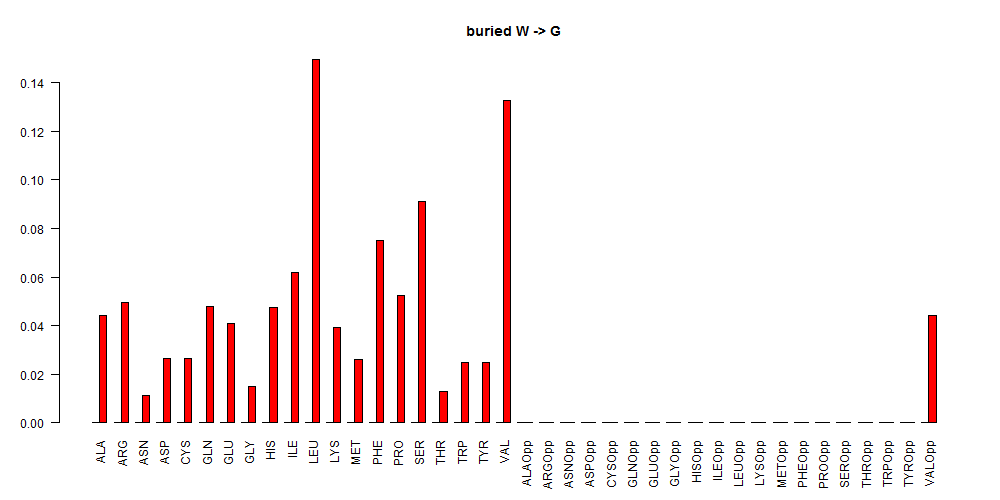

Supplement: Dataset S3 — Neighbouring residue profiles for mutations classed by substitution. (ZIP) [file pone.0084598.s003.zip › neighbour_2/buried_W_G.tif]

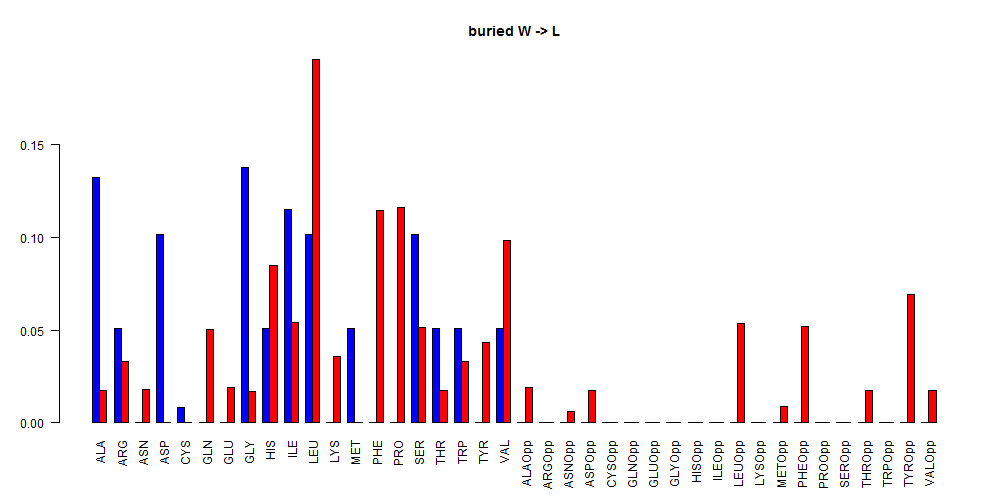

Supplement: Dataset S3 — Neighbouring residue profiles for mutations classed by substitution. (ZIP) [file pone.0084598.s003.zip › neighbour_2/buried_W_L.tif]

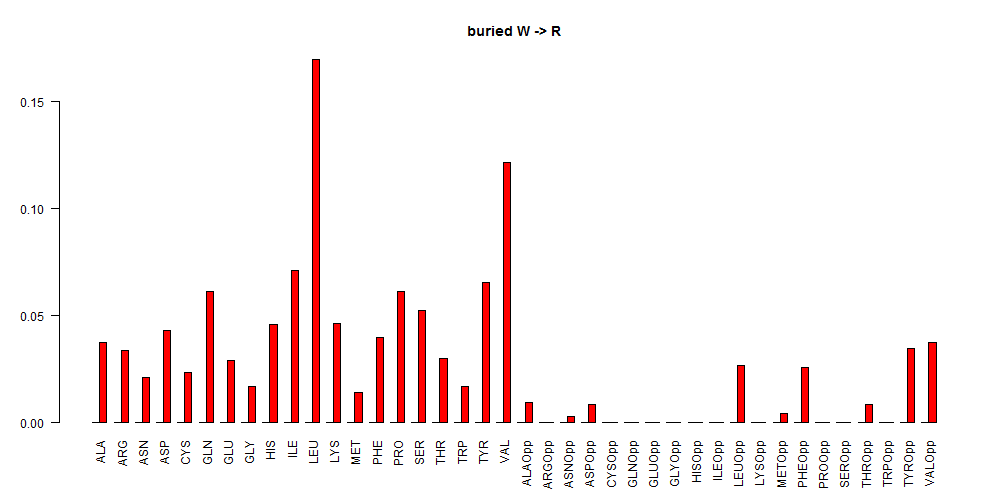

Supplement: Dataset S3 — Neighbouring residue profiles for mutations classed by substitution. (ZIP) [file pone.0084598.s003.zip › neighbour_2/buried_W_R.tif]

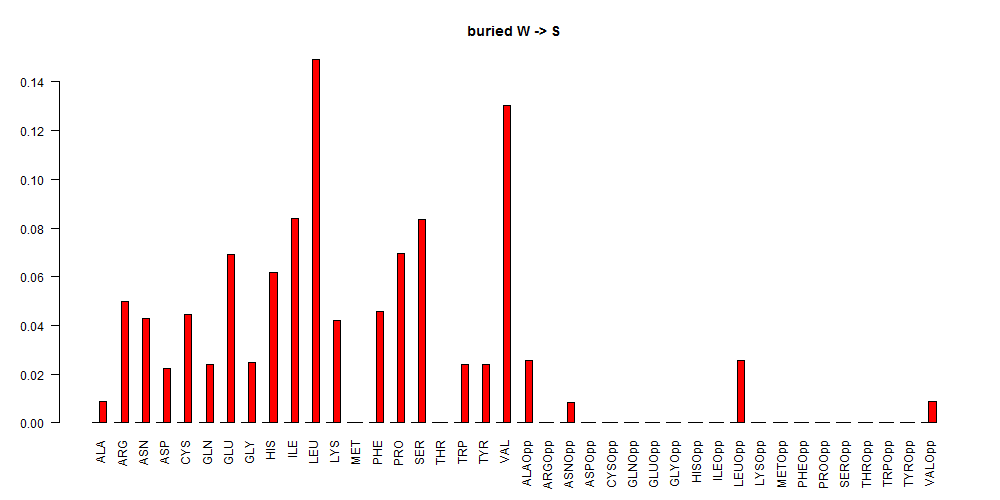

Supplement: Dataset S3 — Neighbouring residue profiles for mutations classed by substitution. (ZIP) [file pone.0084598.s003.zip › neighbour_2/buried_W_S.tif]

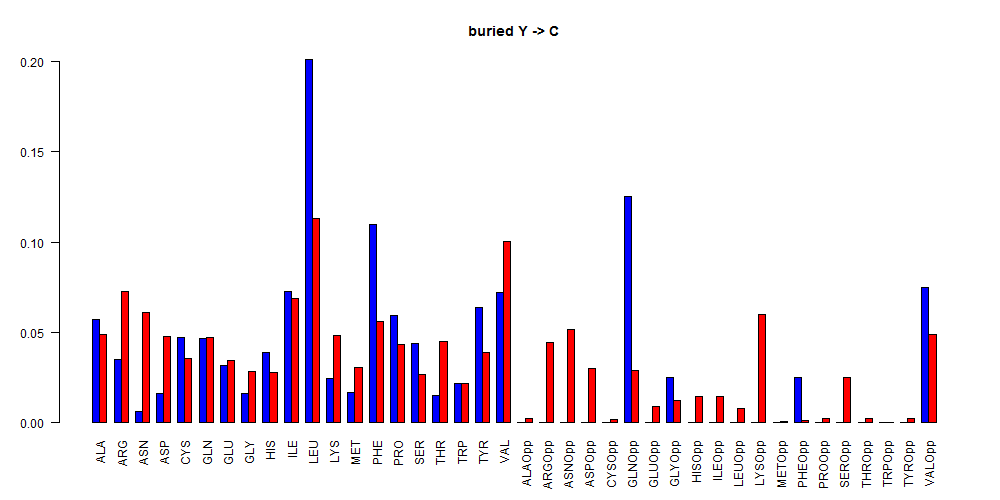

Supplement: Dataset S3 — Neighbouring residue profiles for mutations classed by substitution. (ZIP) [file pone.0084598.s003.zip › neighbour_2/buried_Y_C.tif]

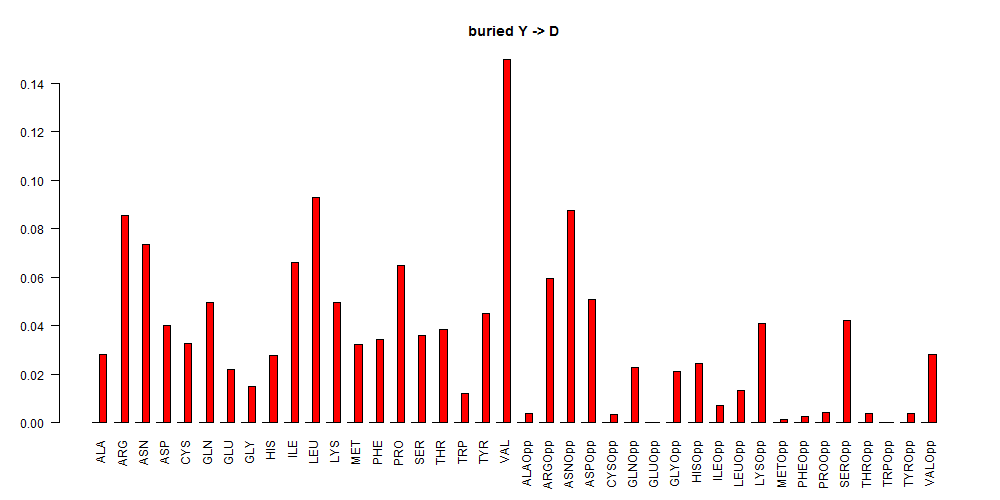

Supplement: Dataset S3 — Neighbouring residue profiles for mutations classed by substitution. (ZIP) [file pone.0084598.s003.zip › neighbour_2/buried_Y_D.tif]

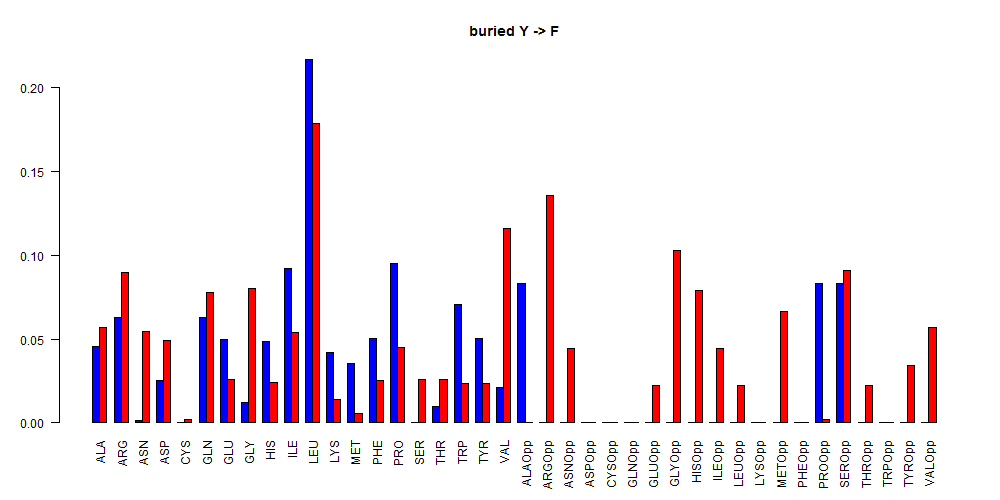

Supplement: Dataset S3 — Neighbouring residue profiles for mutations classed by substitution. (ZIP) [file pone.0084598.s003.zip › neighbour_2/buried_Y_F.tif]

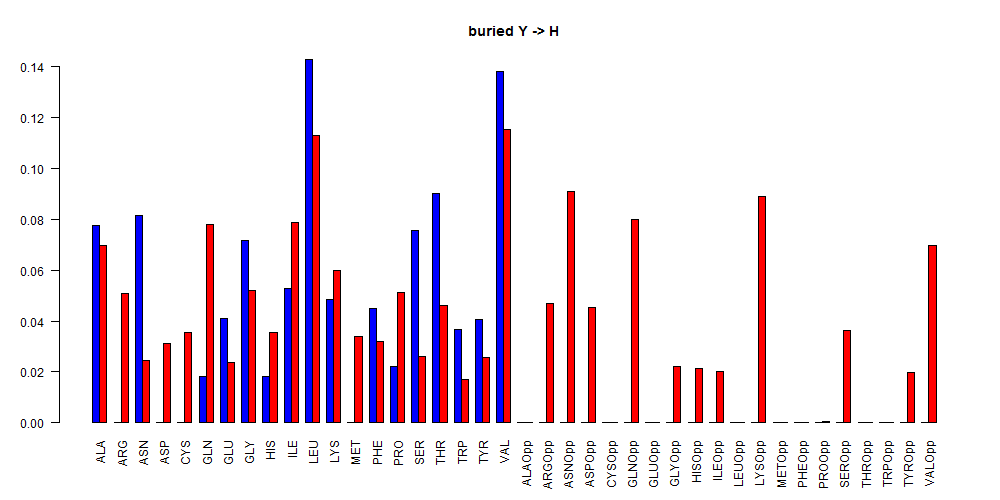

Supplement: Dataset S3 — Neighbouring residue profiles for mutations classed by substitution. (ZIP) [file pone.0084598.s003.zip › neighbour_2/buried_Y_H.tif]

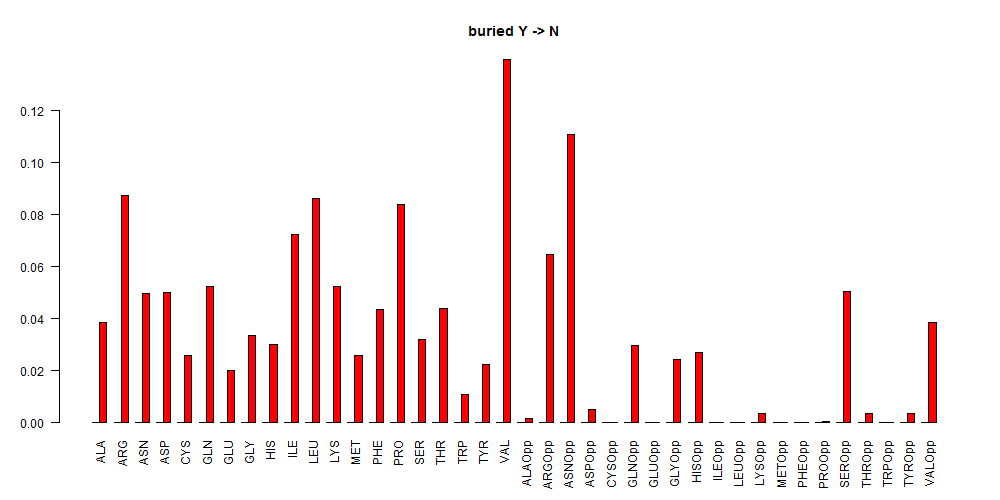

Supplement: Dataset S3 — Neighbouring residue profiles for mutations classed by substitution. (ZIP) [file pone.0084598.s003.zip › neighbour_2/buried_Y_N.tif]

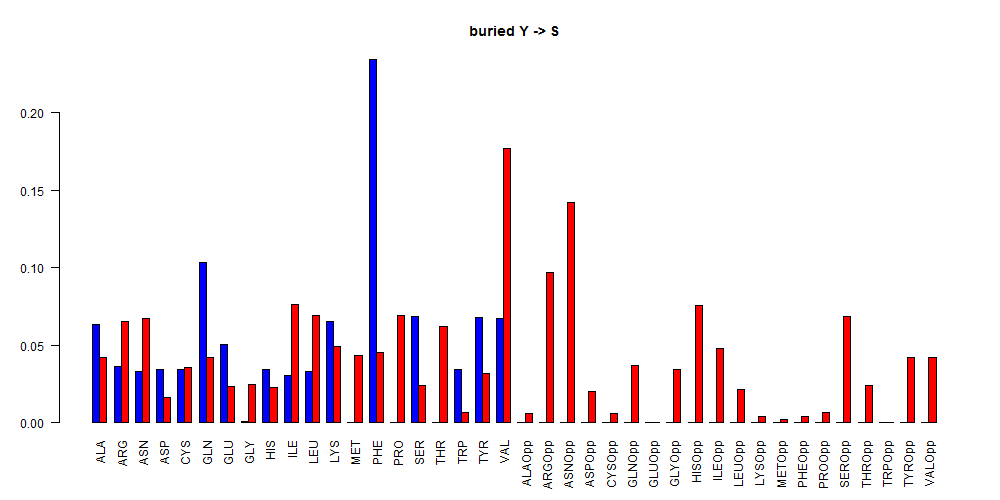

Supplement: Dataset S3 — Neighbouring residue profiles for mutations classed by substitution. (ZIP) [file pone.0084598.s003.zip › neighbour_2/buried_Y_S.tif]

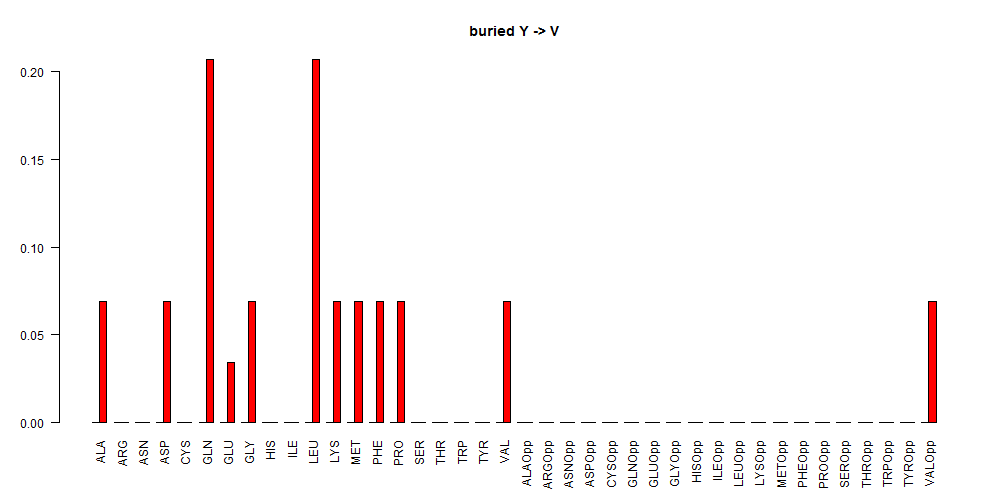

Supplement: Dataset S3 — Neighbouring residue profiles for mutations classed by substitution. (ZIP) [file pone.0084598.s003.zip › neighbour_2/buried_Y_V.tif]

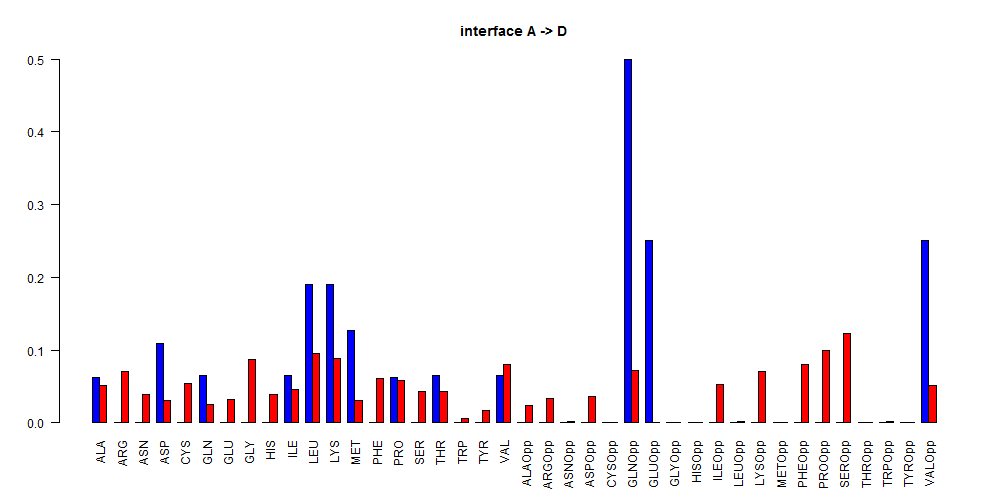

Supplement: Dataset S3 — Neighbouring residue profiles for mutations classed by substitution. (ZIP) [file pone.0084598.s003.zip › neighbour_2/interface_A_D.tif]

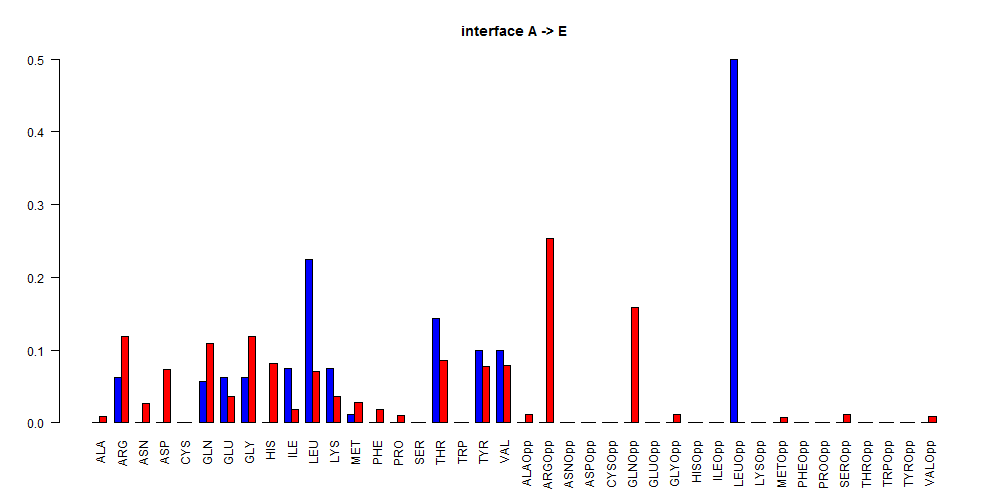

Supplement: Dataset S3 — Neighbouring residue profiles for mutations classed by substitution. (ZIP) [file pone.0084598.s003.zip › neighbour_2/interface_A_E.tif]

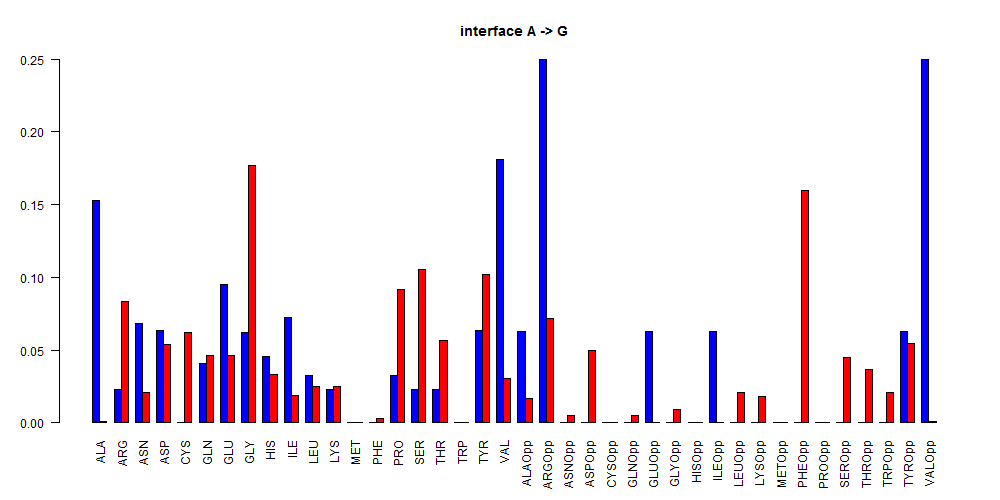

Supplement: Dataset S3 — Neighbouring residue profiles for mutations classed by substitution. (ZIP) [file pone.0084598.s003.zip › neighbour_2/interface_A_G.tif]

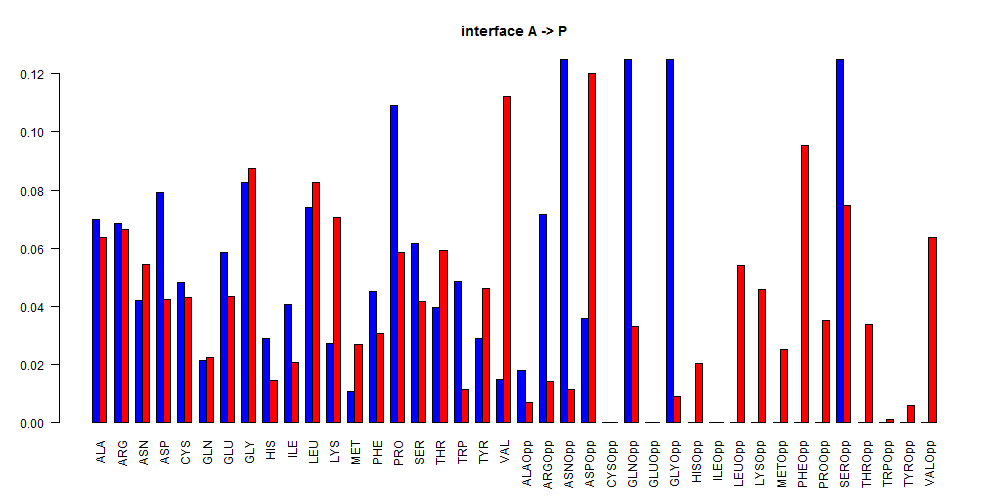

Supplement: Dataset S3 — Neighbouring residue profiles for mutations classed by substitution. (ZIP) [file pone.0084598.s003.zip › neighbour_2/interface_A_P.tif]

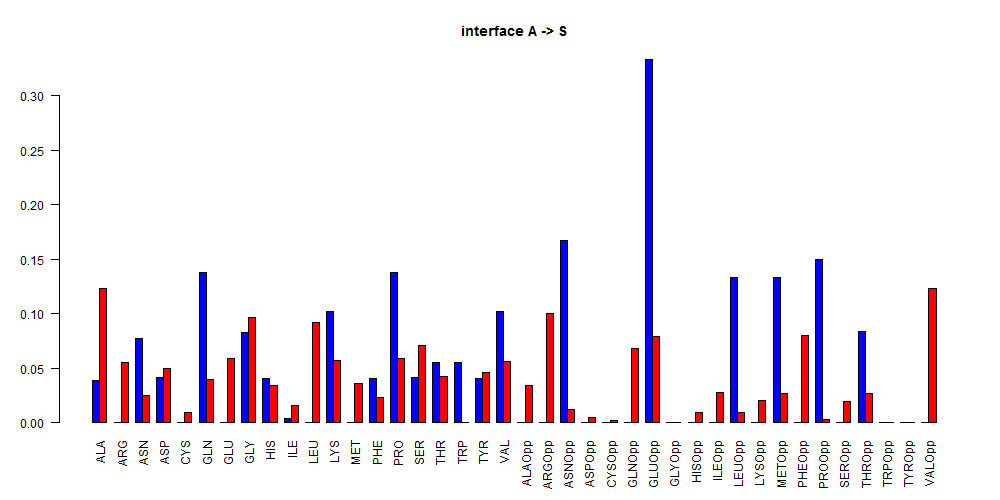

Supplement: Dataset S3 — Neighbouring residue profiles for mutations classed by substitution. (ZIP) [file pone.0084598.s003.zip › neighbour_2/interface_A_S.tif]

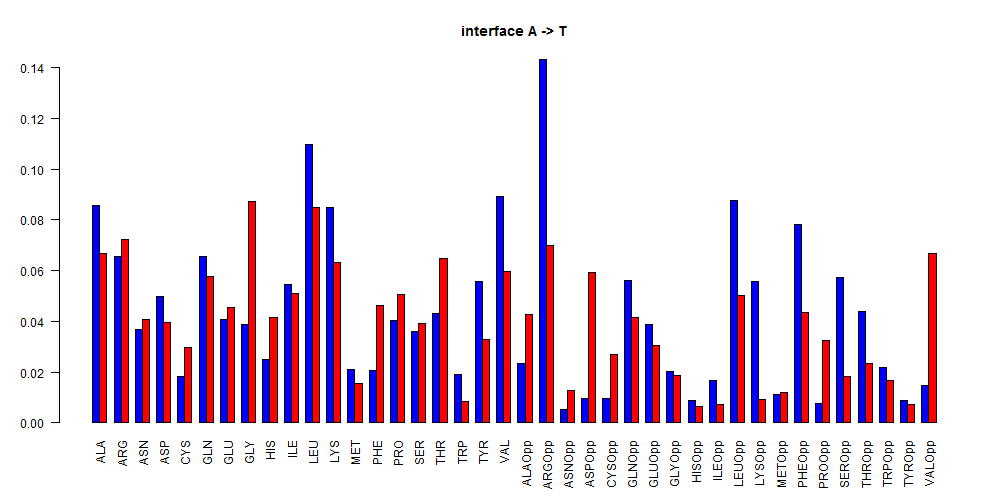

Supplement: Dataset S3 — Neighbouring residue profiles for mutations classed by substitution. (ZIP) [file pone.0084598.s003.zip › neighbour_2/interface_A_T.tif]

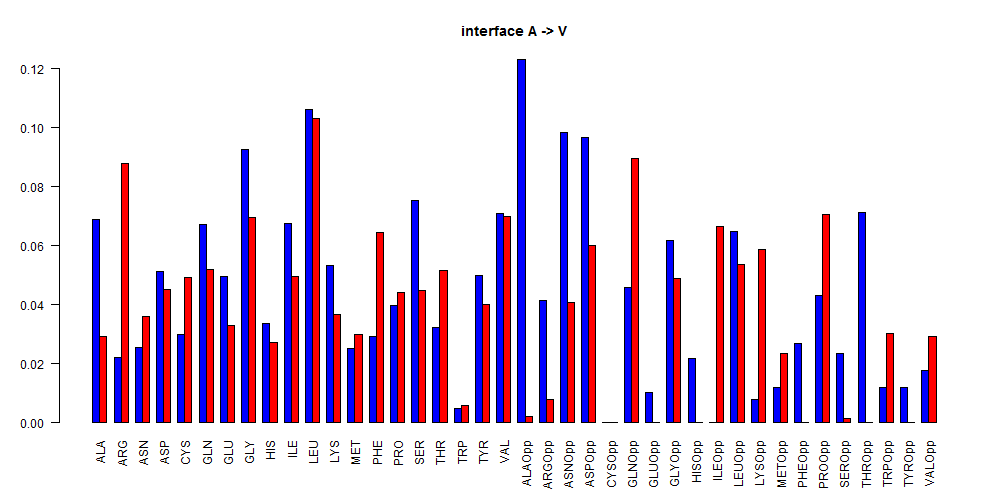

Supplement: Dataset S3 — Neighbouring residue profiles for mutations classed by substitution. (ZIP) [file pone.0084598.s003.zip › neighbour_2/interface_A_V.tif]

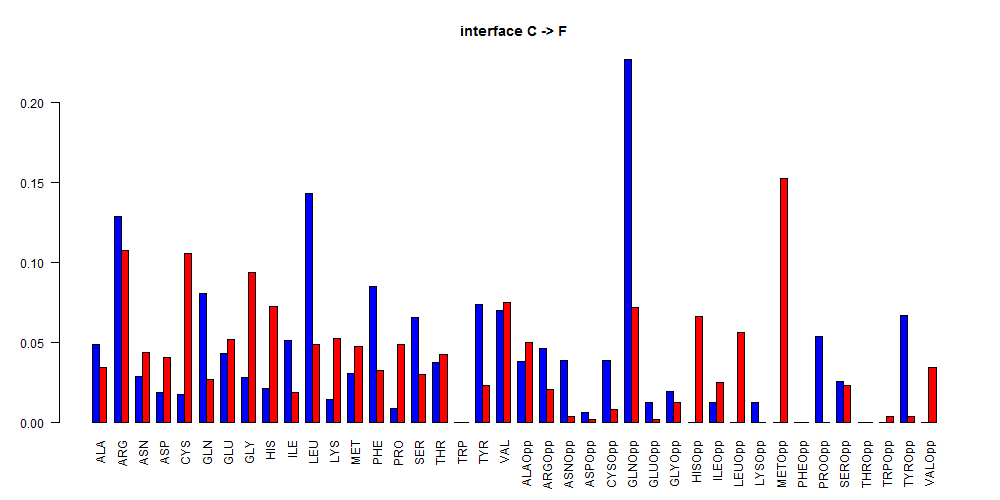

Supplement: Dataset S3 — Neighbouring residue profiles for mutations classed by substitution. (ZIP) [file pone.0084598.s003.zip › neighbour_2/interface_C_F.tif]

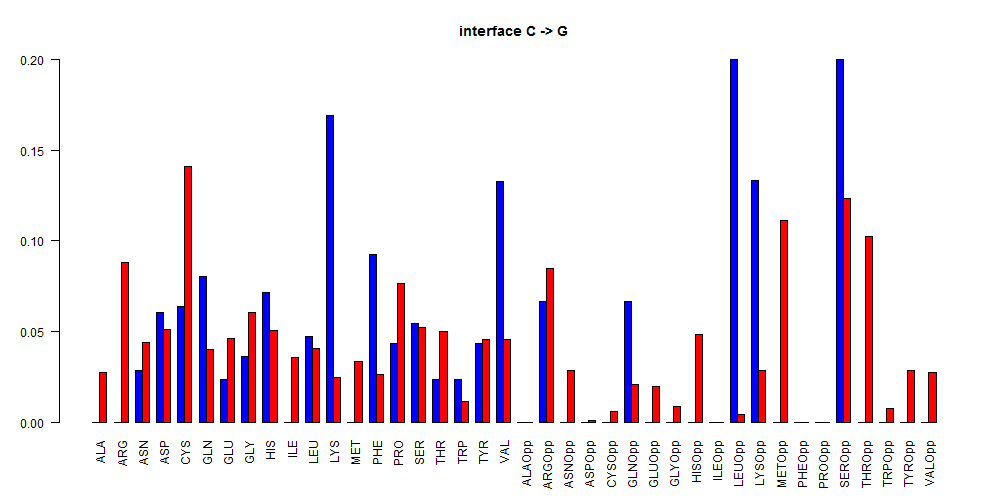

Supplement: Dataset S3 — Neighbouring residue profiles for mutations classed by substitution. (ZIP) [file pone.0084598.s003.zip › neighbour_2/interface_C_G.tif]

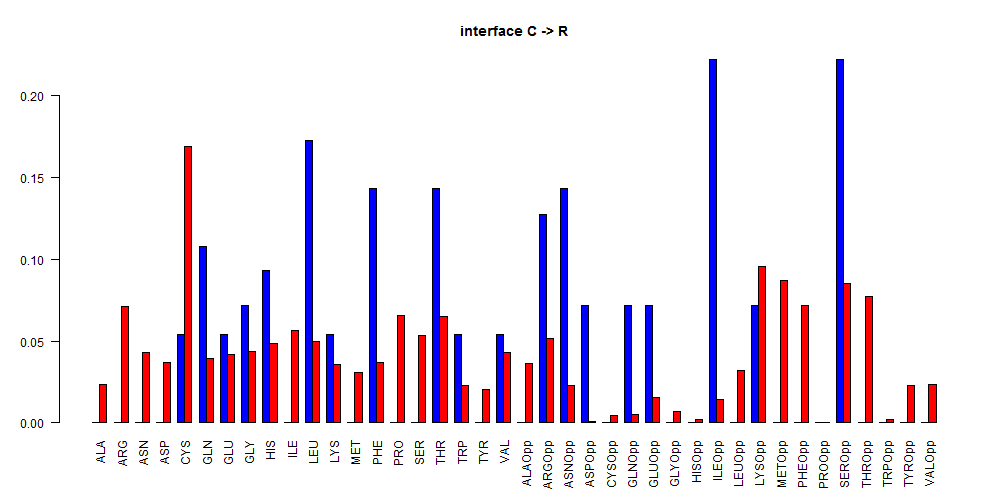

Supplement: Dataset S3 — Neighbouring residue profiles for mutations classed by substitution. (ZIP) [file pone.0084598.s003.zip › neighbour_2/interface_C_R.tif]

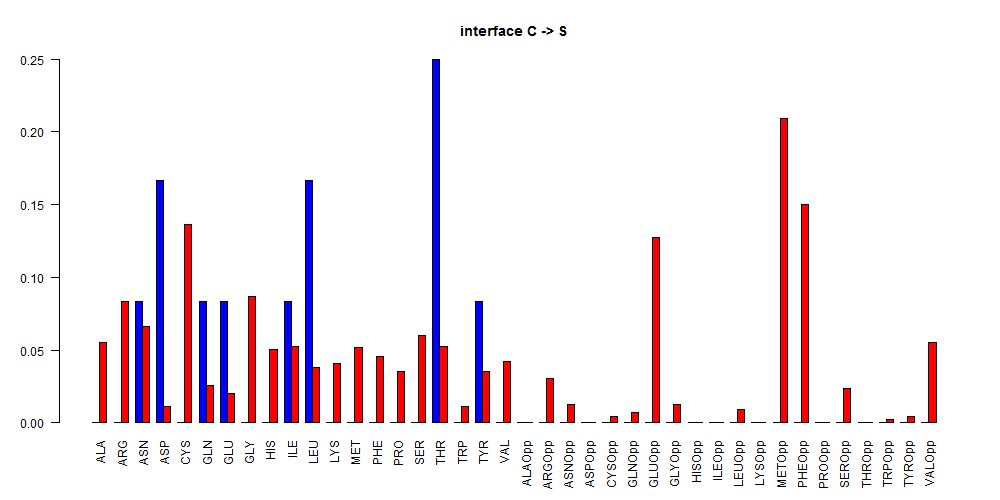

Supplement: Dataset S3 — Neighbouring residue profiles for mutations classed by substitution. (ZIP) [file pone.0084598.s003.zip › neighbour_2/interface_C_S.tif]

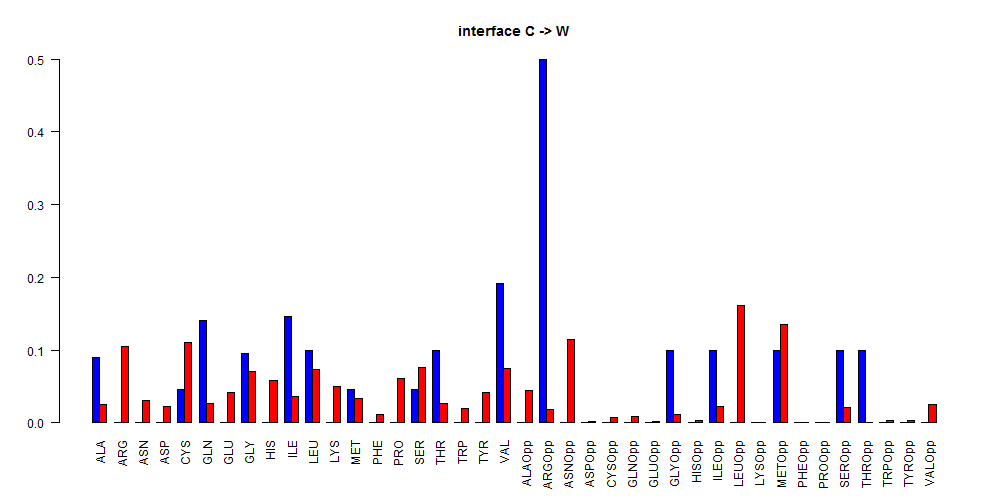

Supplement: Dataset S3 — Neighbouring residue profiles for mutations classed by substitution. (ZIP) [file pone.0084598.s003.zip › neighbour_2/interface_C_W.tif]

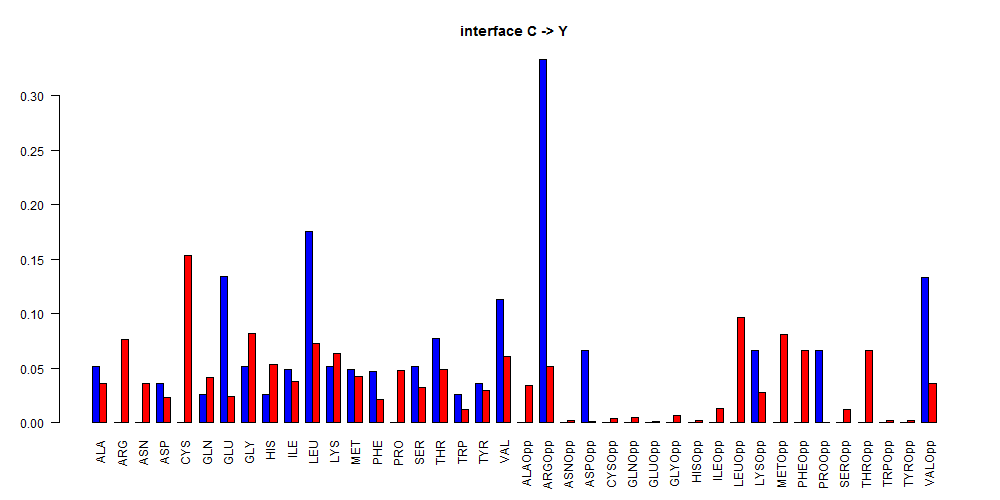

Supplement: Dataset S3 — Neighbouring residue profiles for mutations classed by substitution. (ZIP) [file pone.0084598.s003.zip › neighbour_2/interface_C_Y.tif]

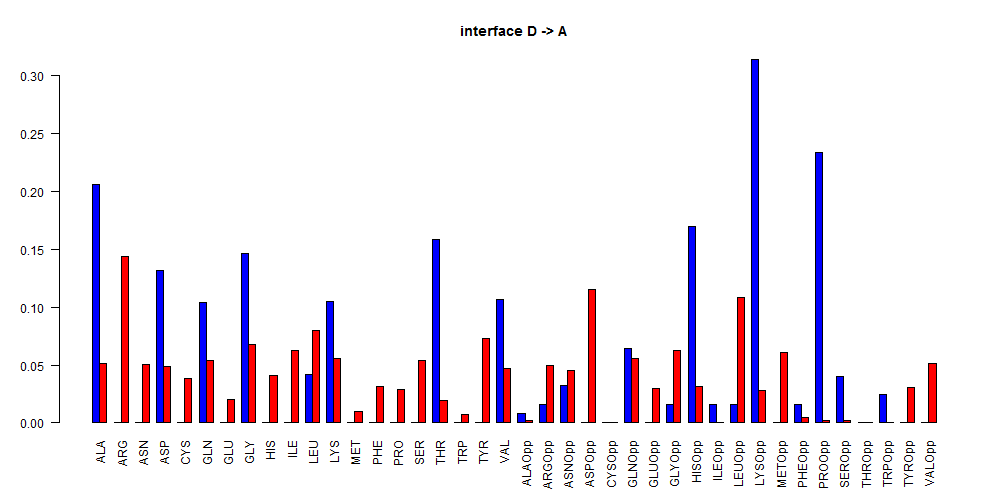

Supplement: Dataset S3 — Neighbouring residue profiles for mutations classed by substitution. (ZIP) [file pone.0084598.s003.zip › neighbour_2/interface_D_A.tif]

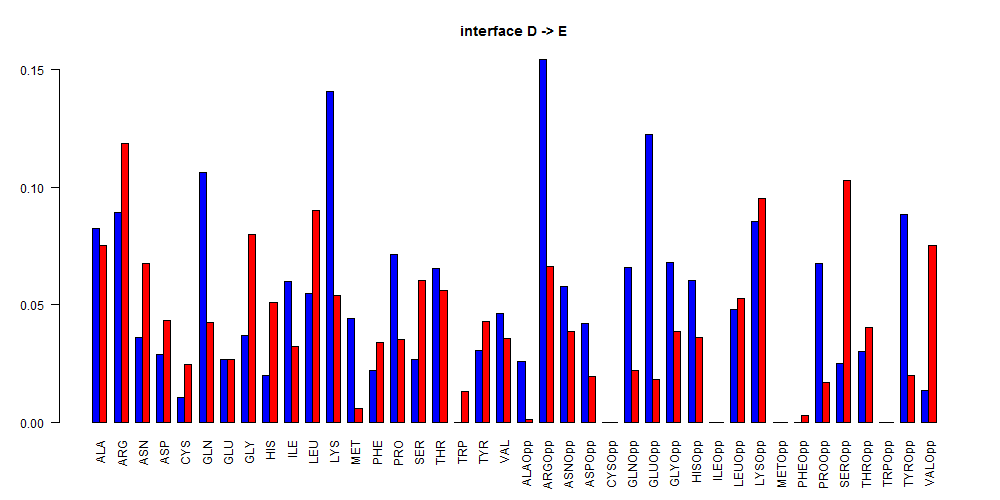

Supplement: Dataset S3 — Neighbouring residue profiles for mutations classed by substitution. (ZIP) [file pone.0084598.s003.zip › neighbour_2/interface_D_E.tif]

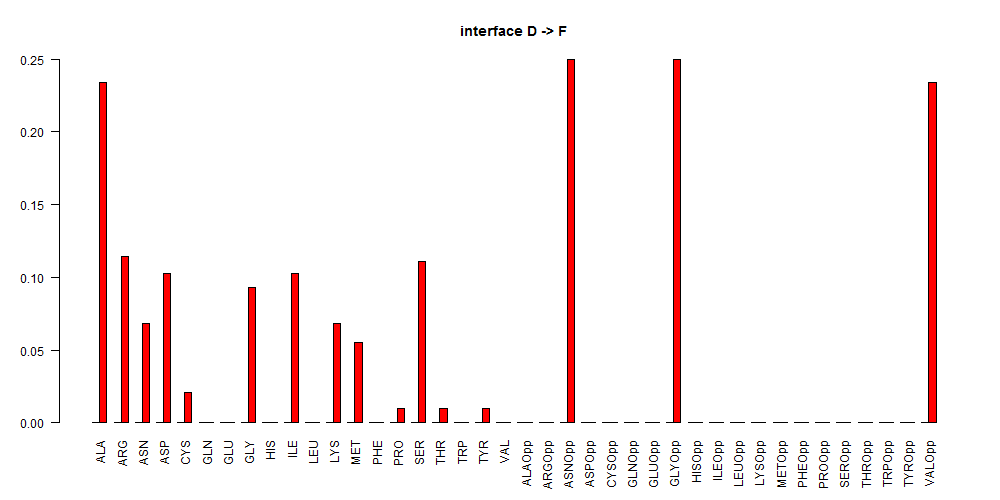

Supplement: Dataset S3 — Neighbouring residue profiles for mutations classed by substitution. (ZIP) [file pone.0084598.s003.zip › neighbour_2/interface_D_F.tif]

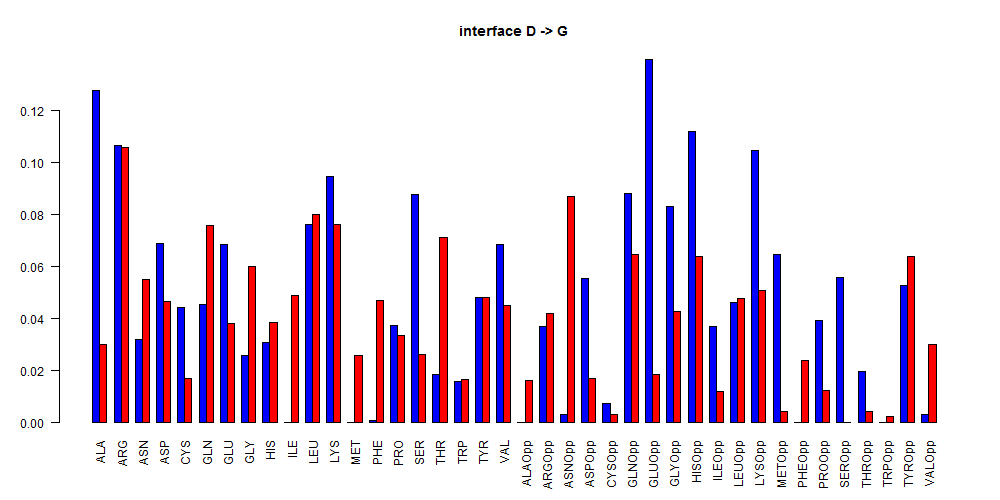

Supplement: Dataset S3 — Neighbouring residue profiles for mutations classed by substitution. (ZIP) [file pone.0084598.s003.zip › neighbour_2/interface_D_G.tif]

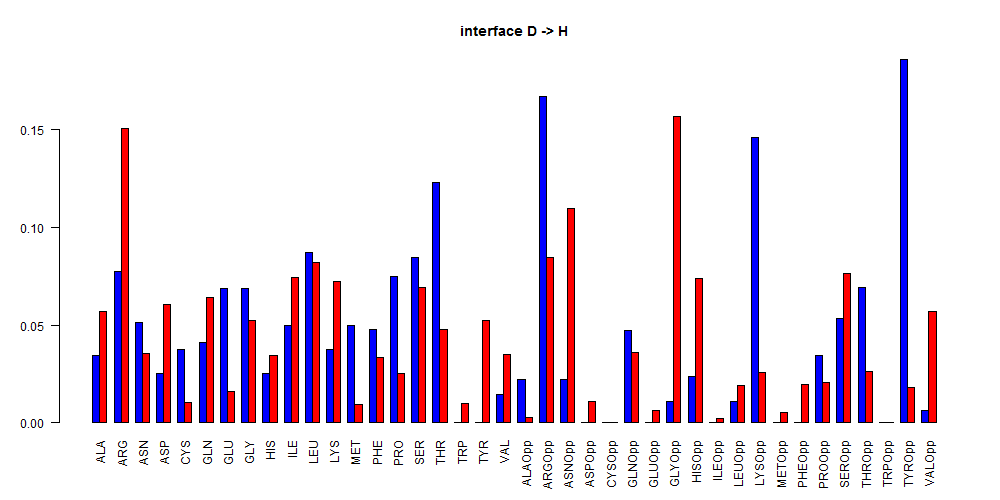

Supplement: Dataset S3 — Neighbouring residue profiles for mutations classed by substitution. (ZIP) [file pone.0084598.s003.zip › neighbour_2/interface_D_H.tif]

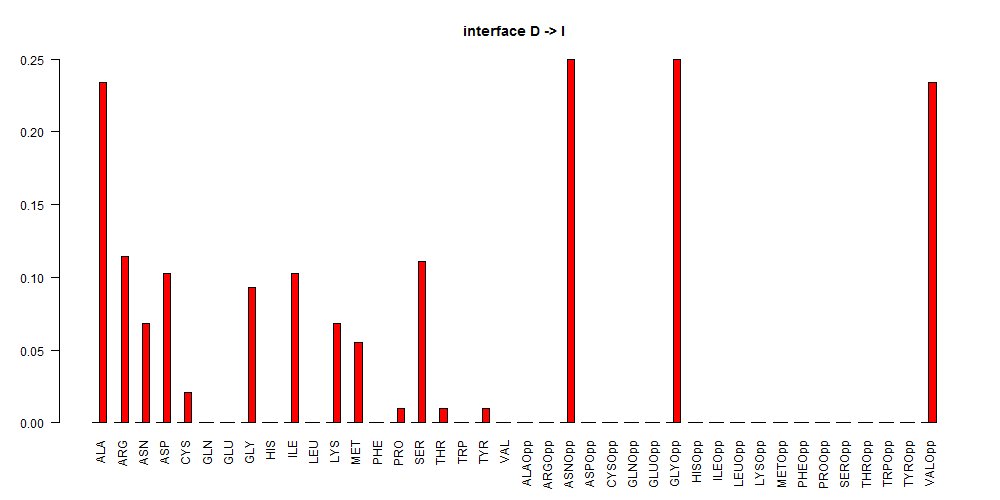

Supplement: Dataset S3 — Neighbouring residue profiles for mutations classed by substitution. (ZIP) [file pone.0084598.s003.zip › neighbour_2/interface_D_I.tif]

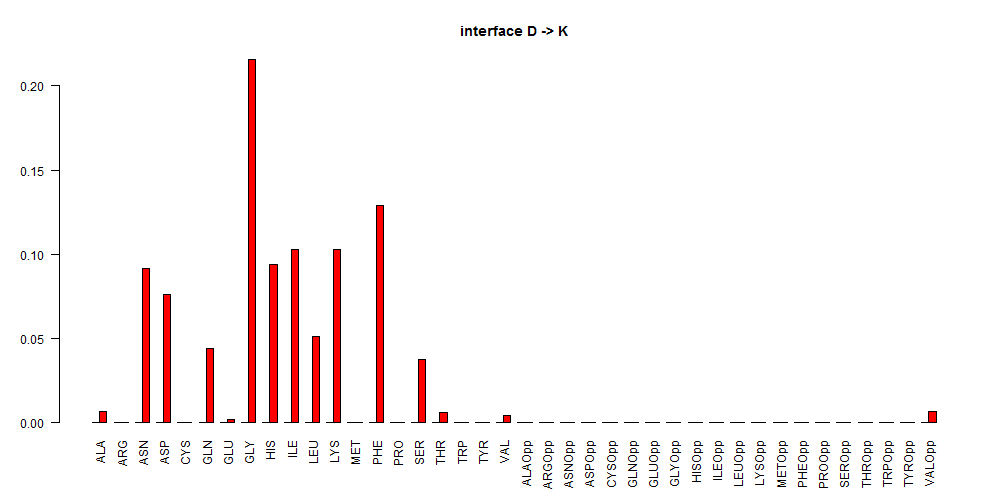

Supplement: Dataset S3 — Neighbouring residue profiles for mutations classed by substitution. (ZIP) [file pone.0084598.s003.zip › neighbour_2/interface_D_K.tif]

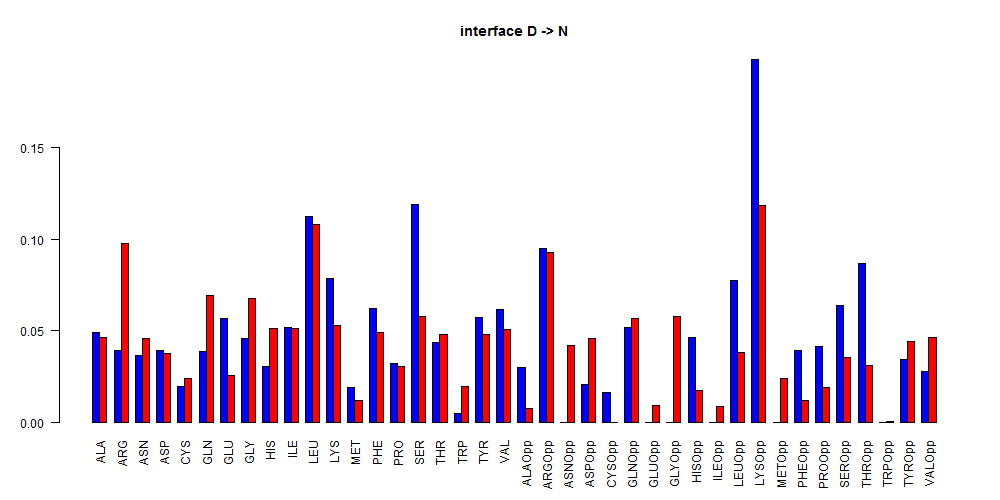

Supplement: Dataset S3 — Neighbouring residue profiles for mutations classed by substitution. (ZIP) [file pone.0084598.s003.zip › neighbour_2/interface_D_N.tif]

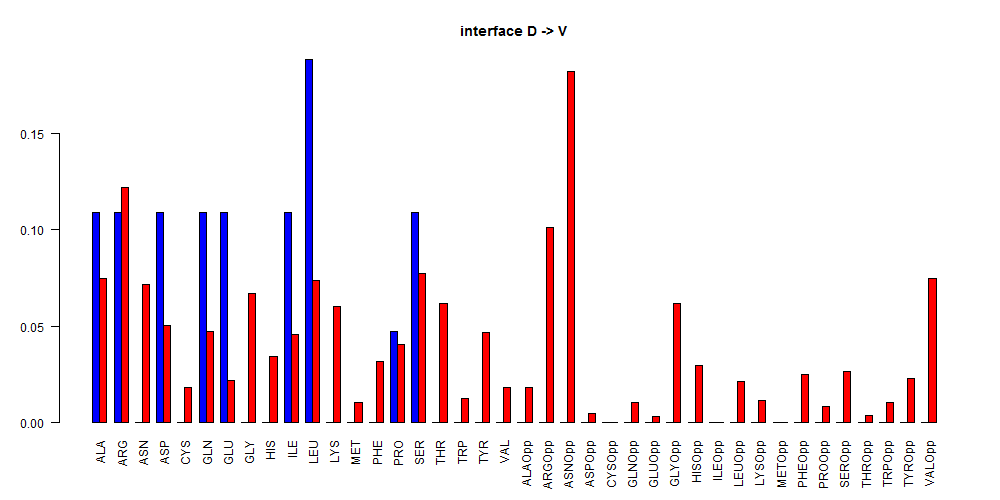

Supplement: Dataset S3 — Neighbouring residue profiles for mutations classed by substitution. (ZIP) [file pone.0084598.s003.zip › neighbour_2/interface_D_V.tif]

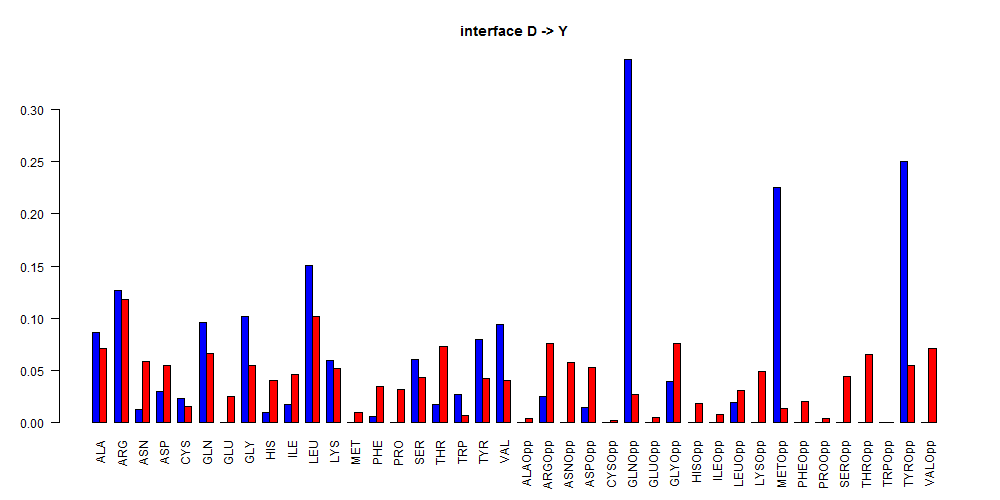

Supplement: Dataset S3 — Neighbouring residue profiles for mutations classed by substitution. (ZIP) [file pone.0084598.s003.zip › neighbour_2/interface_D_Y.tif]

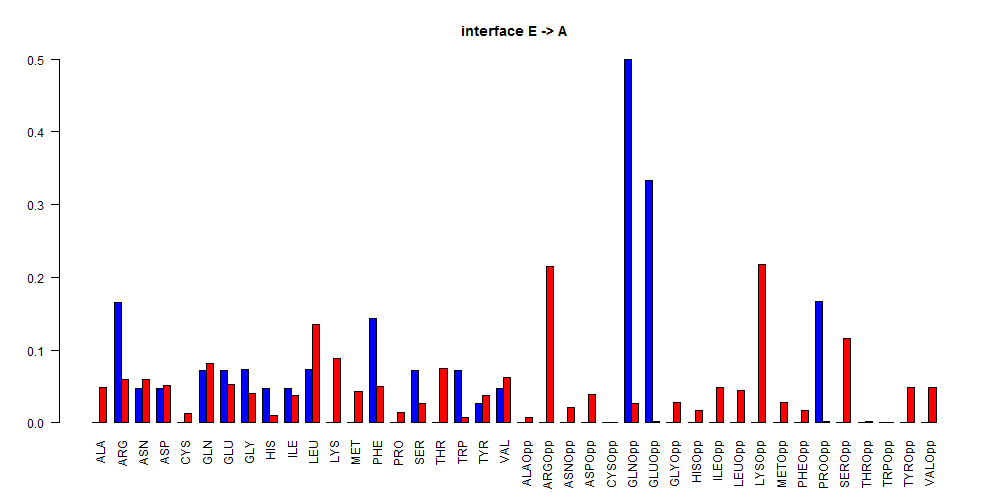

Supplement: Dataset S3 — Neighbouring residue profiles for mutations classed by substitution. (ZIP) [file pone.0084598.s003.zip › neighbour_2/interface_E_A.tif]

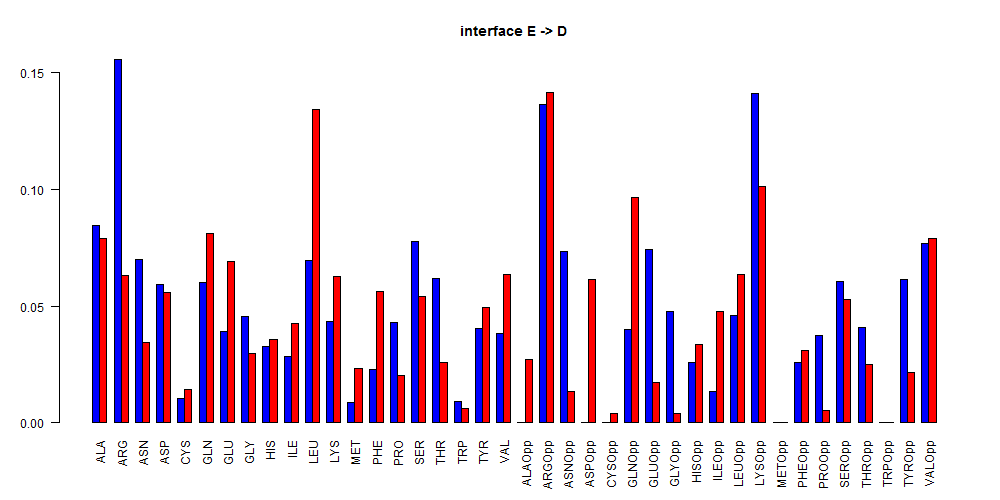

Supplement: Dataset S3 — Neighbouring residue profiles for mutations classed by substitution. (ZIP) [file pone.0084598.s003.zip › neighbour_2/interface_E_D.tif]

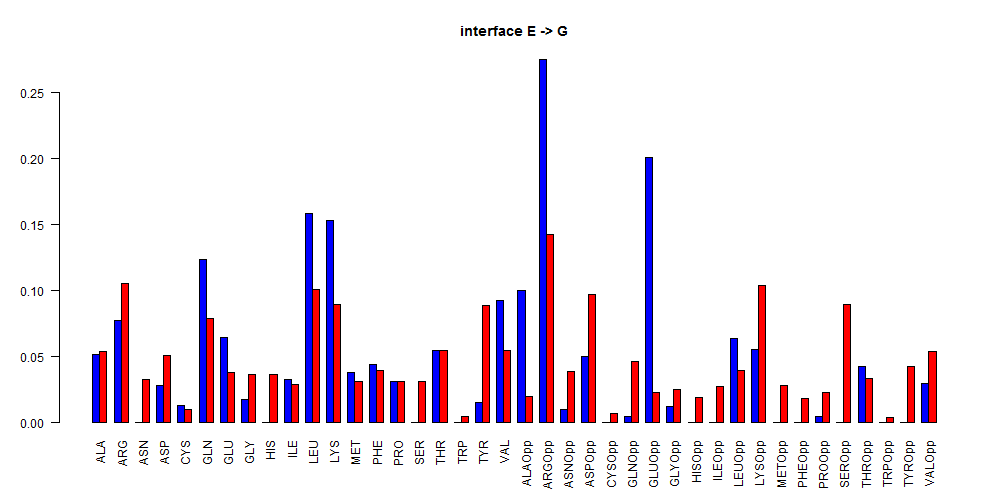

Supplement: Dataset S3 — Neighbouring residue profiles for mutations classed by substitution. (ZIP) [file pone.0084598.s003.zip › neighbour_2/interface_E_G.tif]

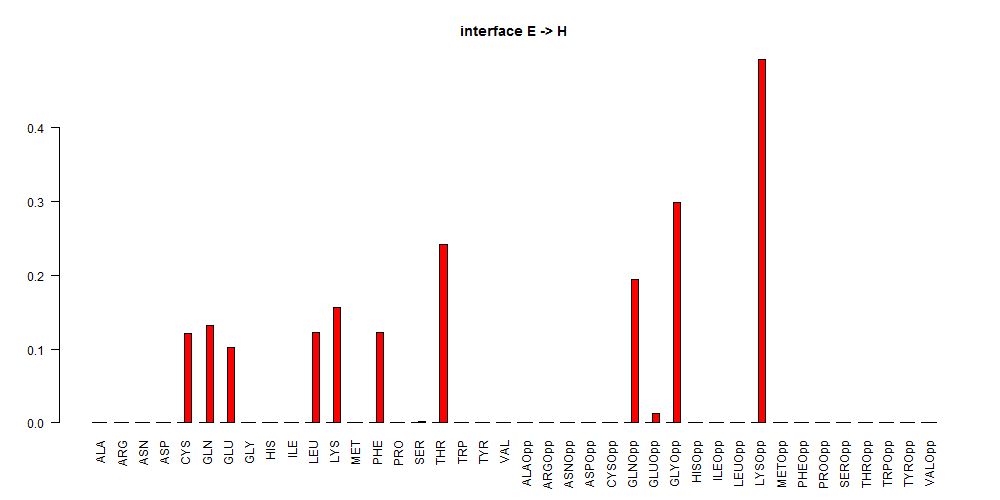

Supplement: Dataset S3 — Neighbouring residue profiles for mutations classed by substitution. (ZIP) [file pone.0084598.s003.zip › neighbour_2/interface_E_H.tif]

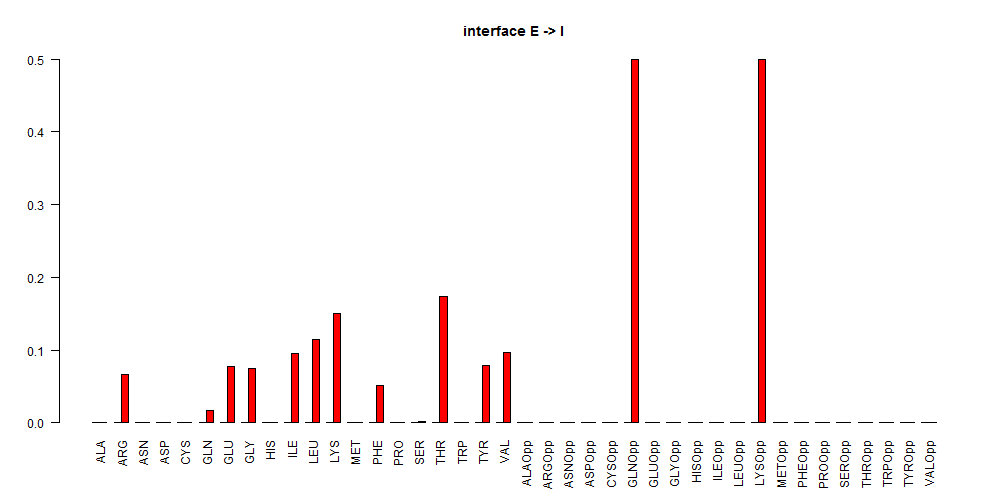

Supplement: Dataset S3 — Neighbouring residue profiles for mutations classed by substitution. (ZIP) [file pone.0084598.s003.zip › neighbour_2/interface_E_I.tif]

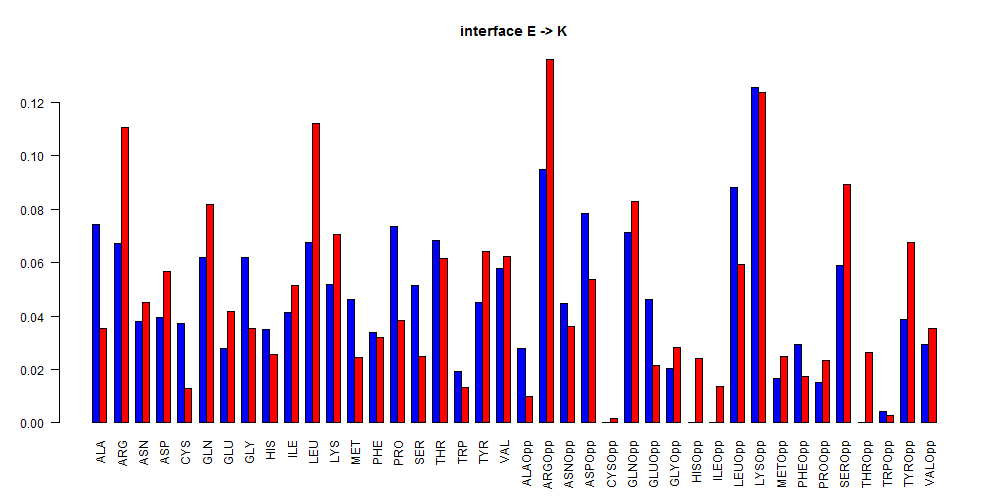

Supplement: Dataset S3 — Neighbouring residue profiles for mutations classed by substitution. (ZIP) [file pone.0084598.s003.zip › neighbour_2/interface_E_K.tif]

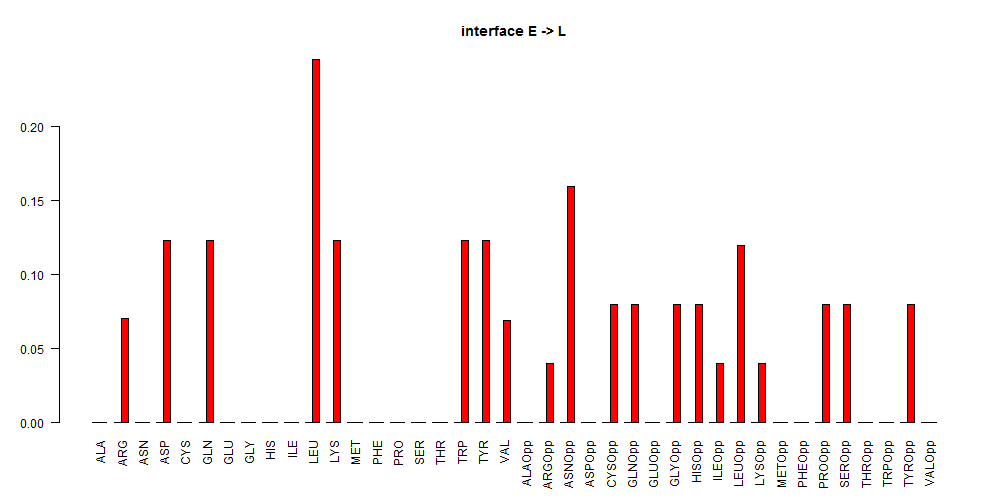

Supplement: Dataset S3 — Neighbouring residue profiles for mutations classed by substitution. (ZIP) [file pone.0084598.s003.zip › neighbour_2/interface_E_L.tif]

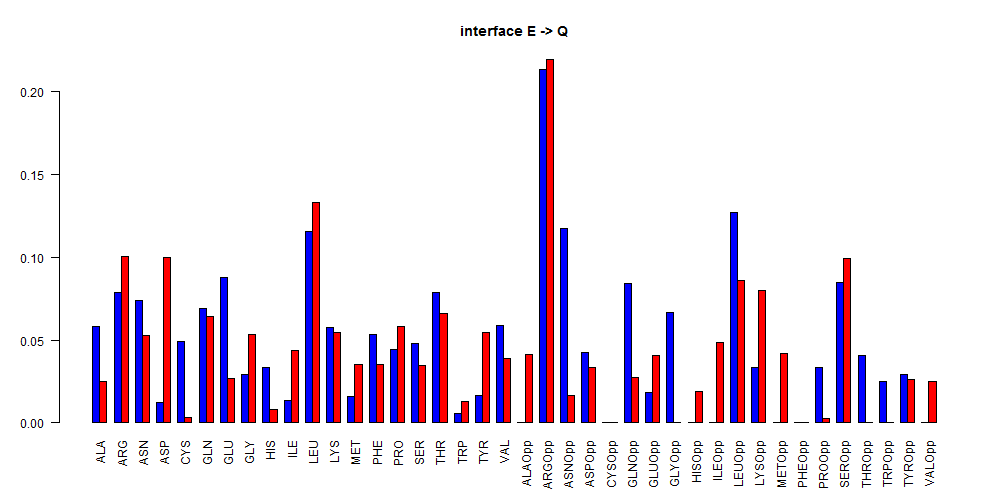

Supplement: Dataset S3 — Neighbouring residue profiles for mutations classed by substitution. (ZIP) [file pone.0084598.s003.zip › neighbour_2/interface_E_Q.tif]

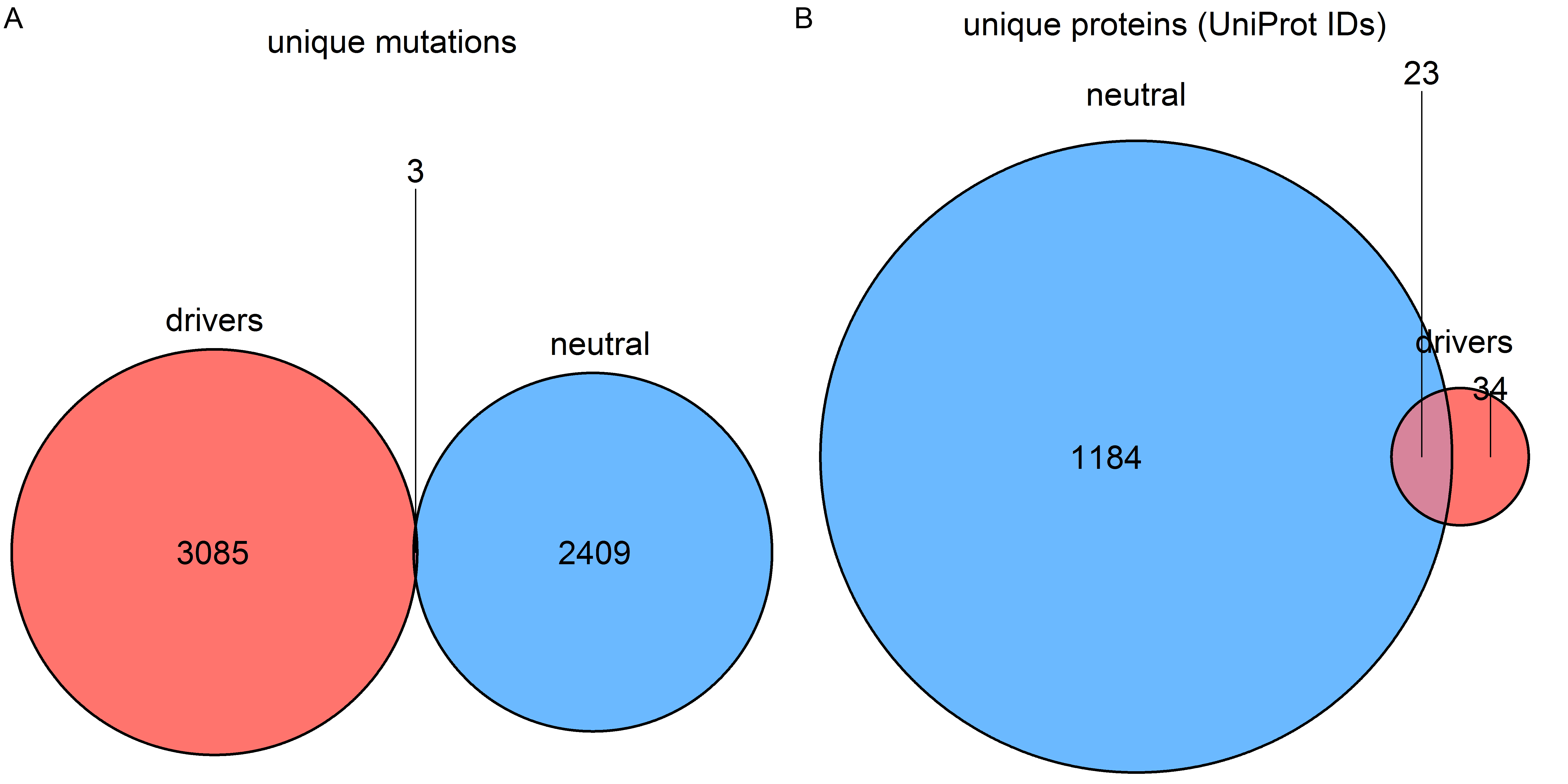

Supplement: Figure S1 — Unique mutations and proteins in the cancer driver and neutral datasets. (TIF) [file pone.0084598.s004.tif]

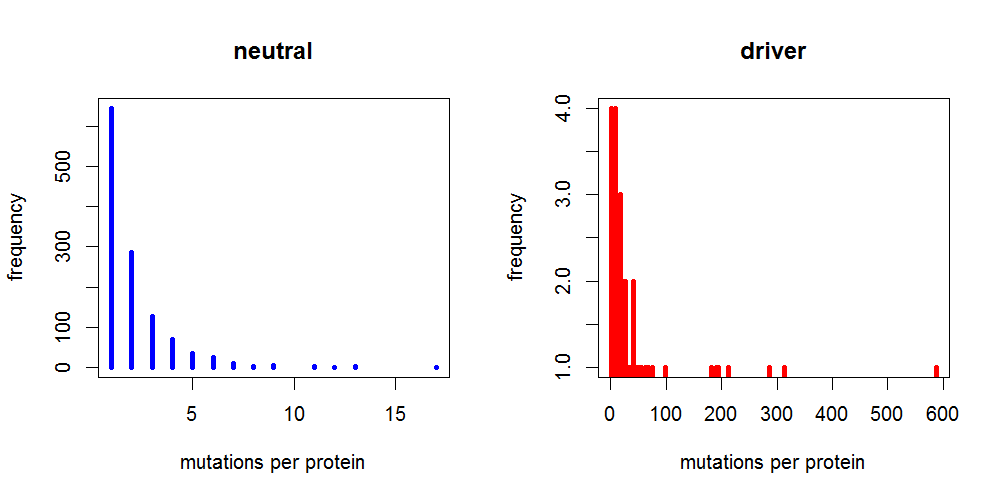

Supplement: Figure S2 — Distributions of amount of mutations per protein. (TIF) [file pone.0084598.s005.tif]

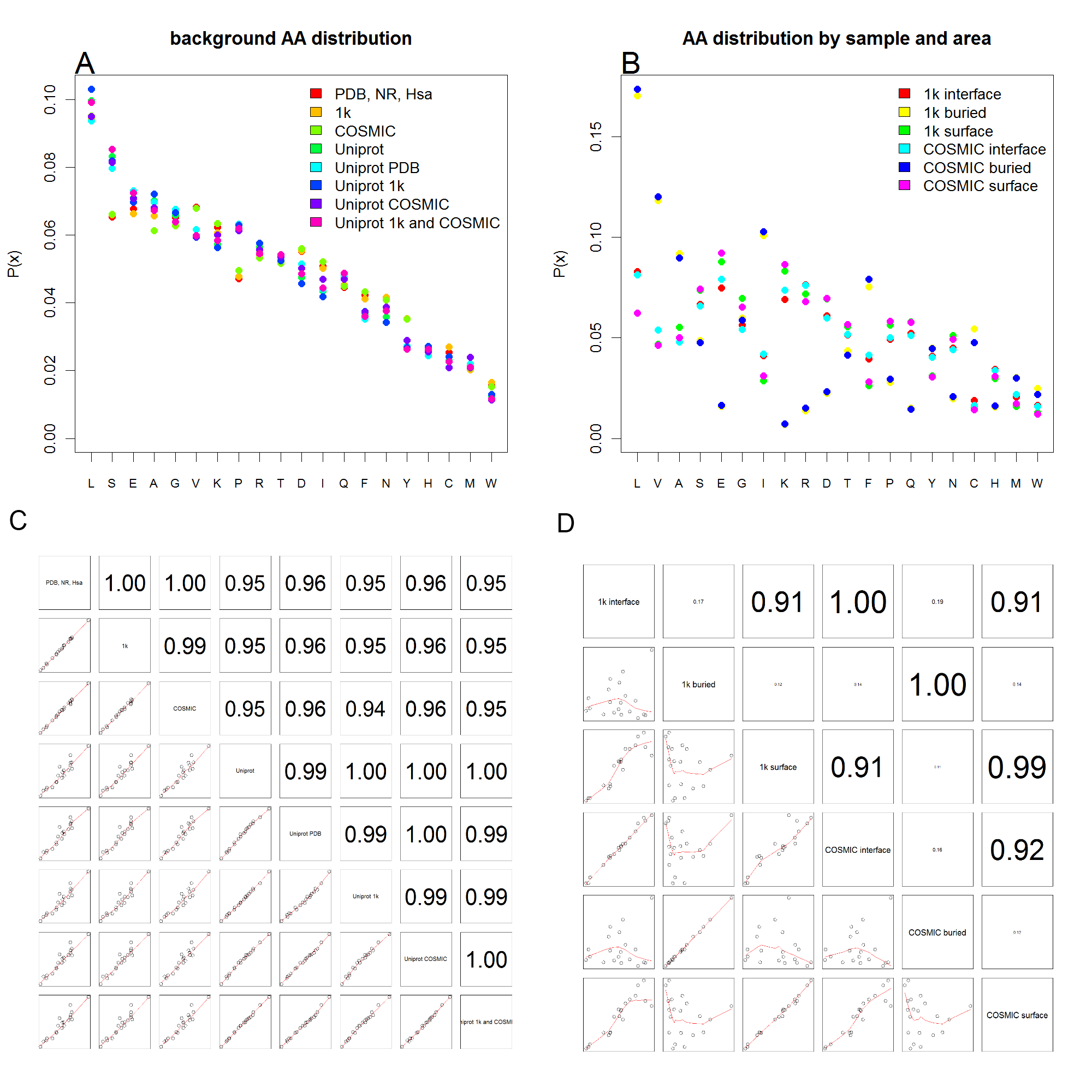

Supplement: Figure S3 — Background distribution of amino acid frequencies in 1000 Genomes and COSMIC. A) Normalised frequencies (denoted P(x) for density) of amino acids in each set. The frequencies are ordered according to average value. B) Normalised frequencies for each sample divided by area. C) Pearson correlation coefficients of each set pair. Smooth trendlines are overlaid in red on plots in the bottom left part of the panel. D) as C) for each sample divided by area. The dataset each series denotes is described below. PDB, NR, Hsa: All non-redundant human crystallised sequences 1k: 1000 Genomes set of non-redundant human crystallised sequences COSMIC: Cosmic set of non-redundant human crystallised sequences Uniprot: Entire Uniprot sequences Uniprot PDB: Entire Uniprot sequences which have PDB entries Uniprot 1k: Entire Uniprot sequences in the 1000 Genomes set Uniprot COSMIC: Entire Uniprot sequences in Cosmic Uniprot 1k and COSMIC: Entire Uniprot sequences in Cosmic and 1000 Genomes. (TIF) [file pone.0084598.s006.tif]

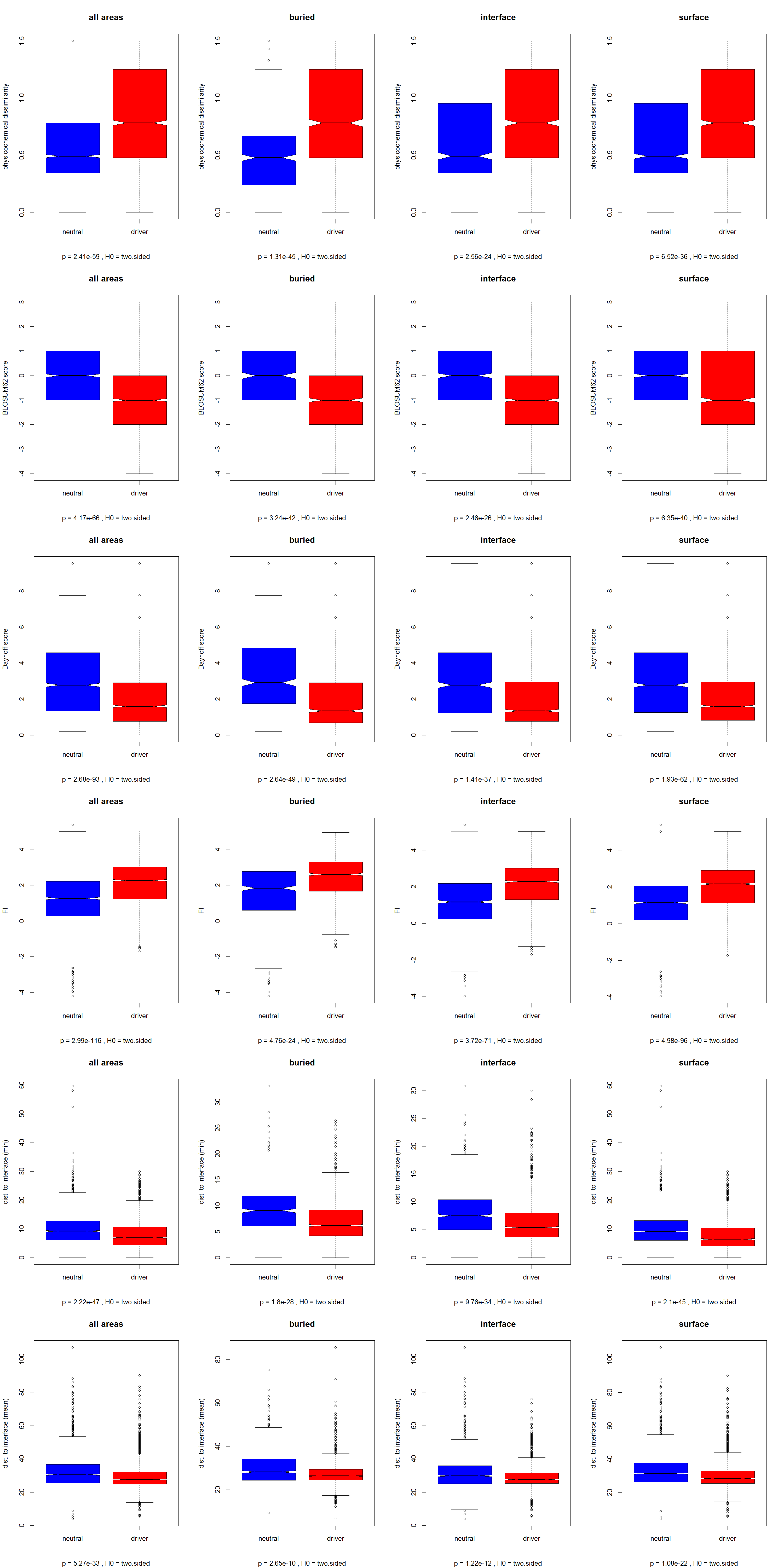

Supplement: Figure S4 — Mutation severity in neutral and driver mutations by physicochemical change of substitution, mutational permissiveness according to BLOSUM 62, Dayhoff, FI and distance to interface. The first row shows plots of change in amino acid physiochemical character incurred by the substitution. The driver mutations show a greater change in physiochemical character, thus presumably incurring a greater disruption to protein stability/function. The second row shows boxplots of mutation substitution severity according to the amino acid substitution values in BLOSUM 62 (EBI). The 1k mutations hover around 0, whereas the driver mutations have less permitted mutability. Rows 3 and 4 show that same using Dayhoff (EBI) (see text) and FI scores. Rows 5 and 6 show minimum and mean distances to interfaces. Because unique residues can have multiple PDB files and each PDB file can have many interfaces, there are several distances from each residue to each interface. The proximity of driver mutations to the interface suggests that cancer mutations tend to disrupt interfaces. (TIF) [file pone.0084598.s007.tif]

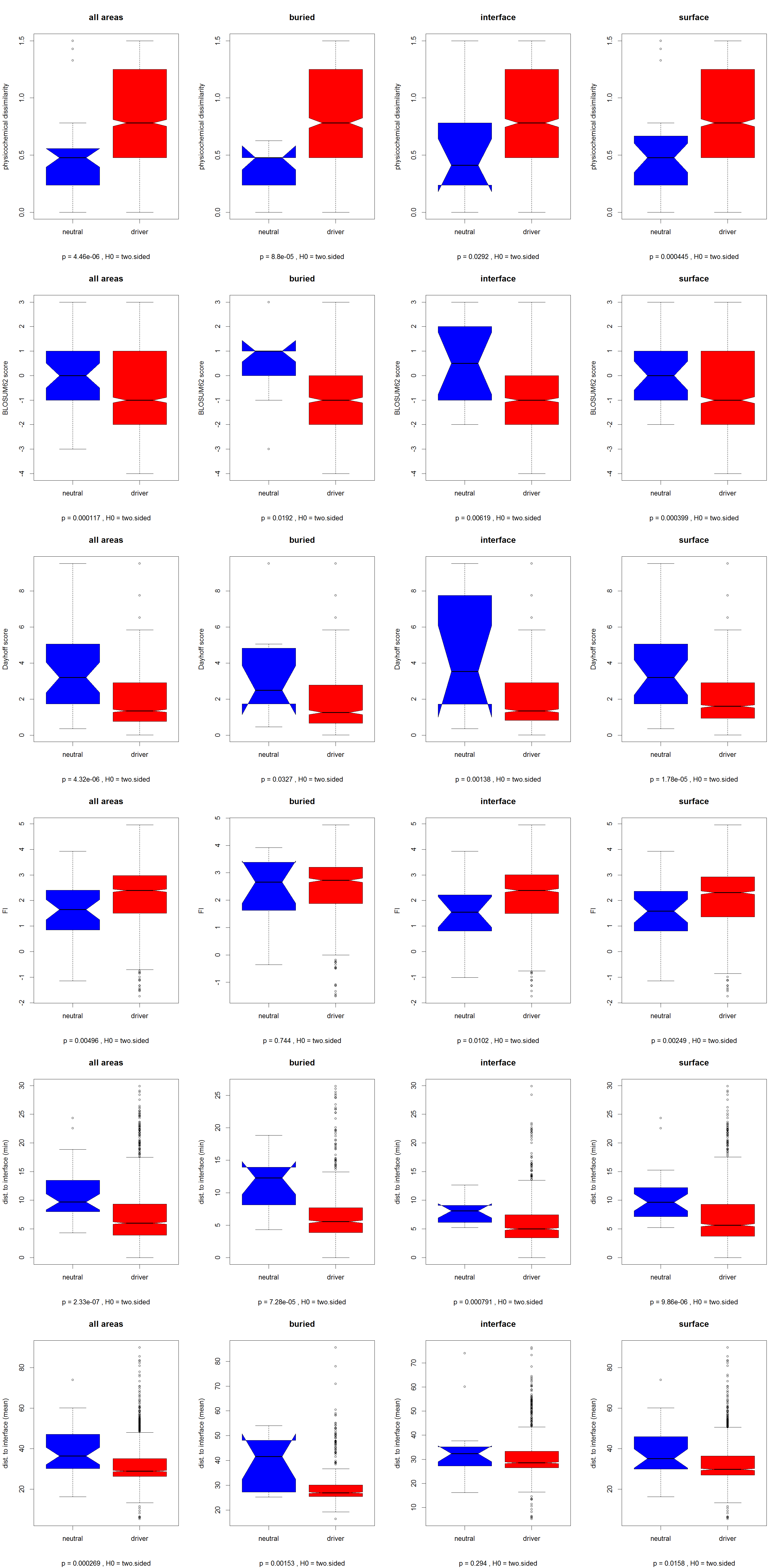

Supplement: Figure S5 — Mutation severity in neutral and driver mutations by physicochemical change of substitution, mutational permissiveness according to BLOSUM 62, Dayhoff, FI and distance to interface, using the reduced set of 23 proteins with both neutral and driver mutations. (TIF) [file pone.0084598.s008.tif]

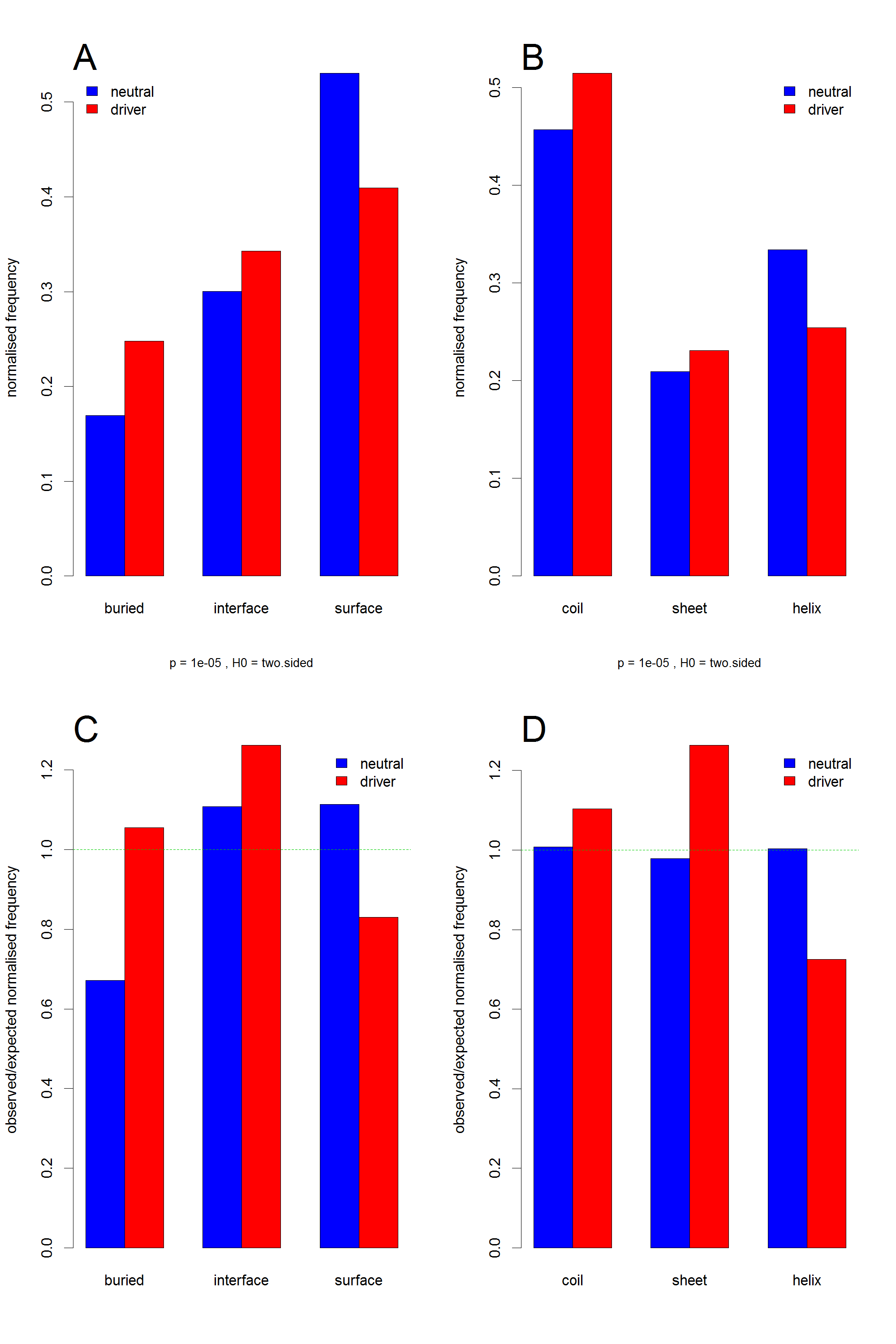

Supplement: Figure S6 — Propensities in mutations split by area and 2ry structure separately. A) Normalised frequency of occurrences of mutations in each area. Cancer mutations occur more frequently in buried and interface areas than neutral mutations. B) Normalised frequency of occurrences of mutations in secondary structures. Most carcinogenic mutations occur in coils and beta sheets and less in helices. There is a small but significant difference (Fisher's test with a two-sided alternative hypothesis) between the driver and 1k samples in both cases. C) Fractions of observed normalised frequency to expected normalised frequency (all residues in proteins) for each area. D) Fractions of observed normalised frequency to expected normalised frequency for each secondary structure. (TIF) [file pone.0084598.s009.tif]

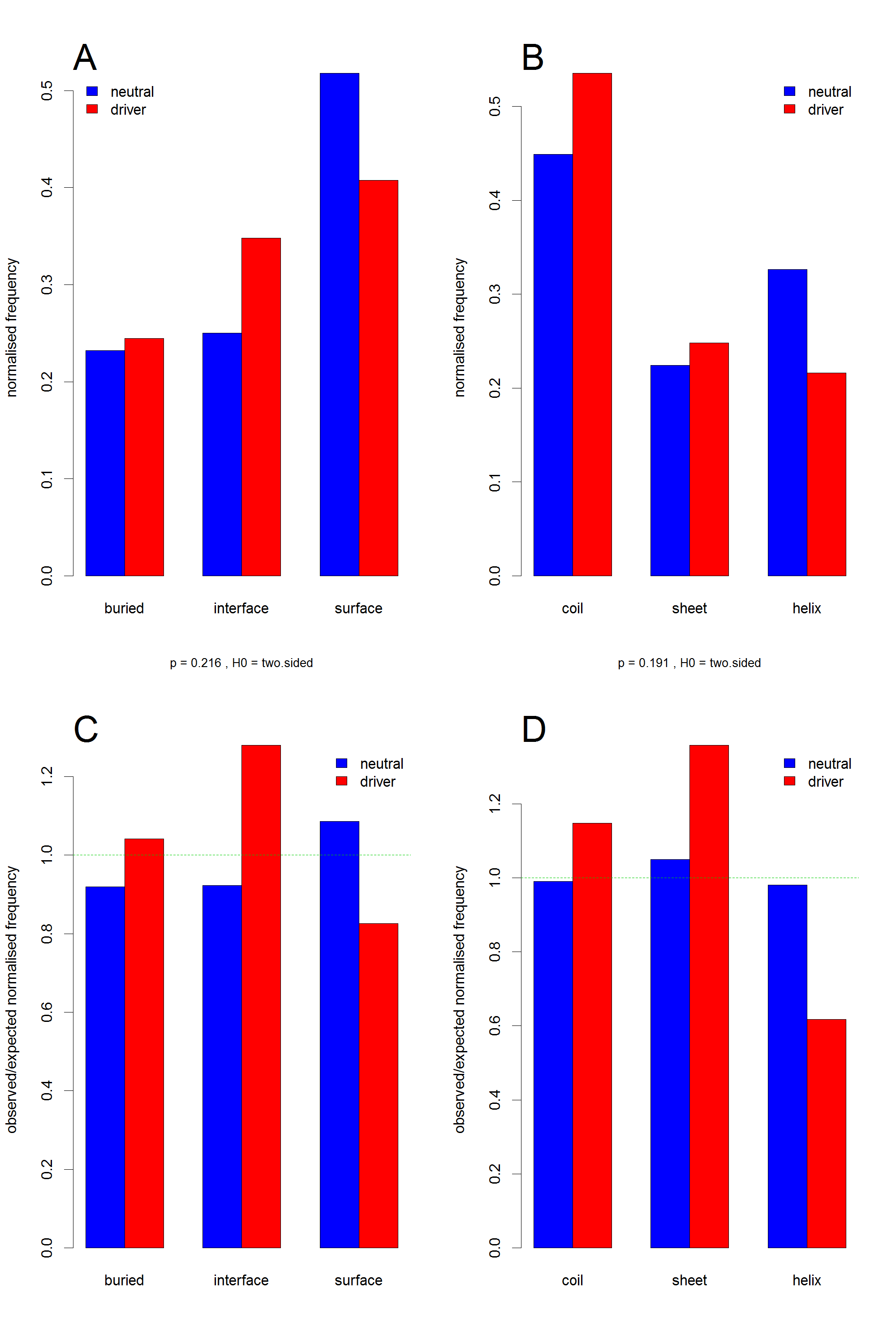

Supplement: Figure S7 — Propensities in mutations split by area and 2ry structure separately, using the reduced set of 23 proteins with both neutral and driver mutations. (TIF) [file pone.0084598.s010.tif]

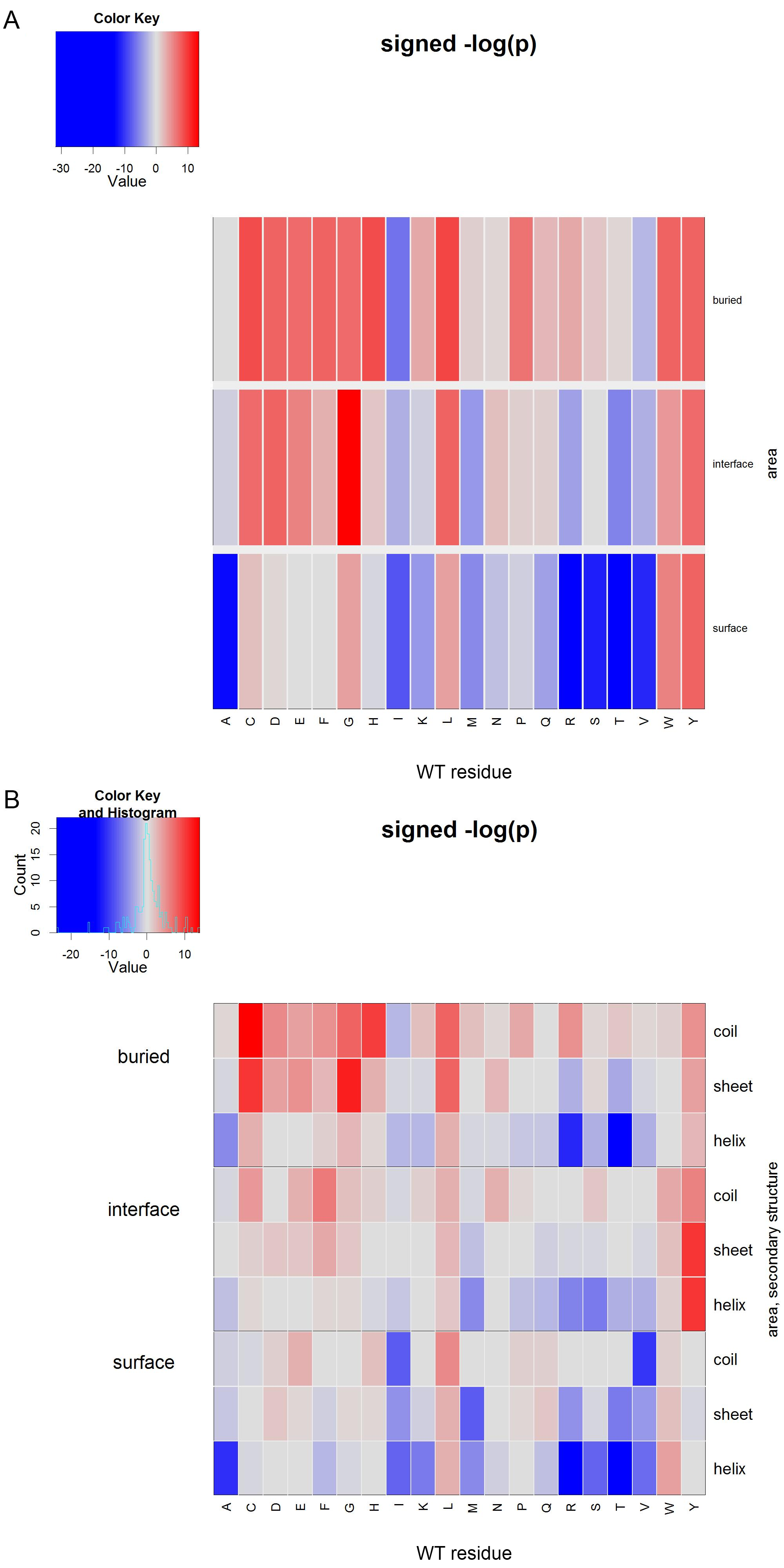

Supplement: Figure S8 — Enriched mutations in area, secondary structure and WT residue comparing neutral and driver mutations. Red denotes enriched classes in drivers and blue denotes enriched classes in neutral mutations. A) Enrichment in driver mutations divided by area and WT residue (s o). B) Enrichment in driver mutations divided by area, secondary structure and WT residue (s o). (TIF) [file pone.0084598.s011.tif]

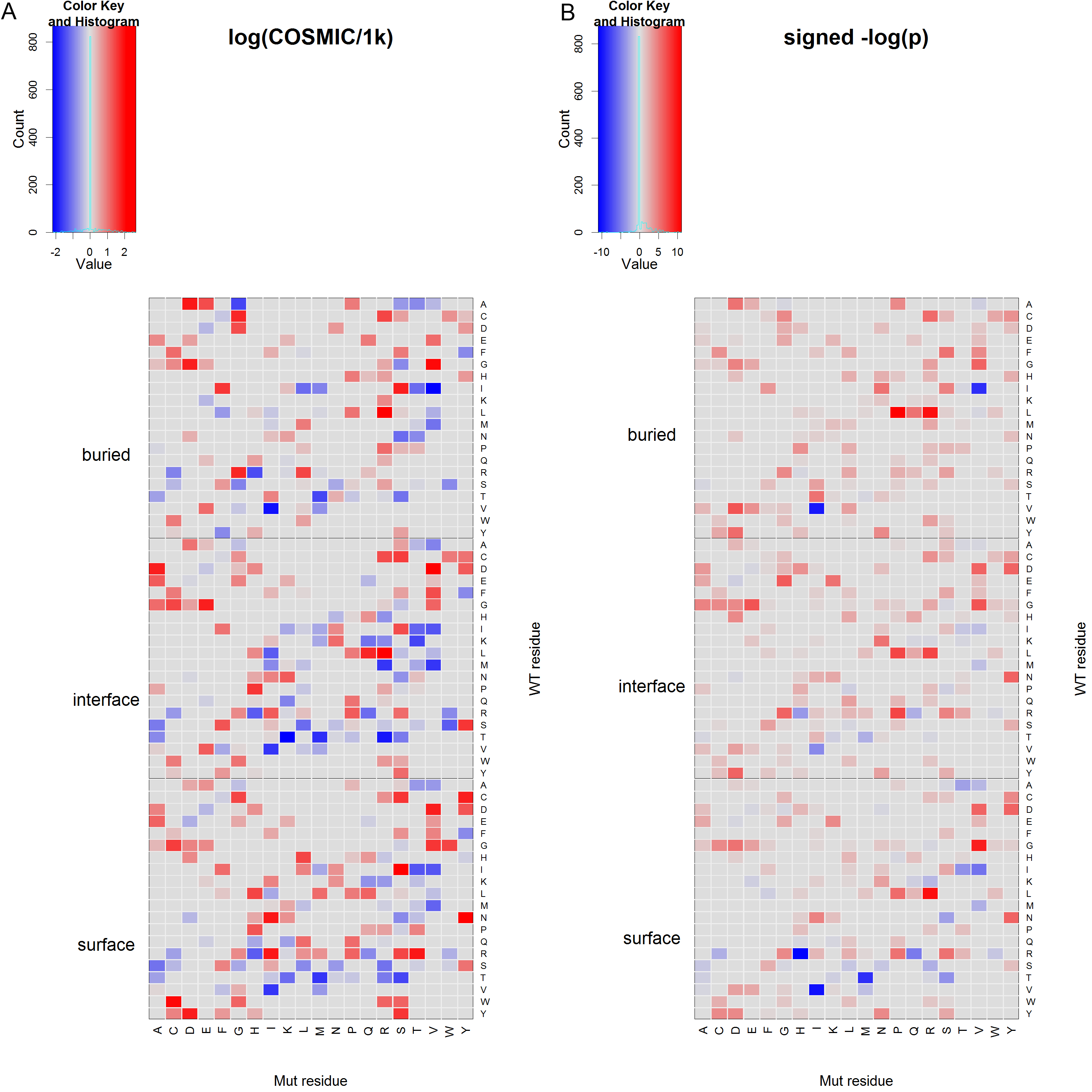

Supplement: Figure S9 — Heatmaps of normalised substitution frequencies and enrichment comparing neutral and driver mutations. Red denotes enriched classes in drivers and blue denotes enriched classes in neutral mutations. A) Driver/neutral fraction of normalised frequencies for mutations by area and substitution. B) Statistically overrepresented substitution frequencies by area (s o). (TIF) [file pone.0084598.s012.tif]

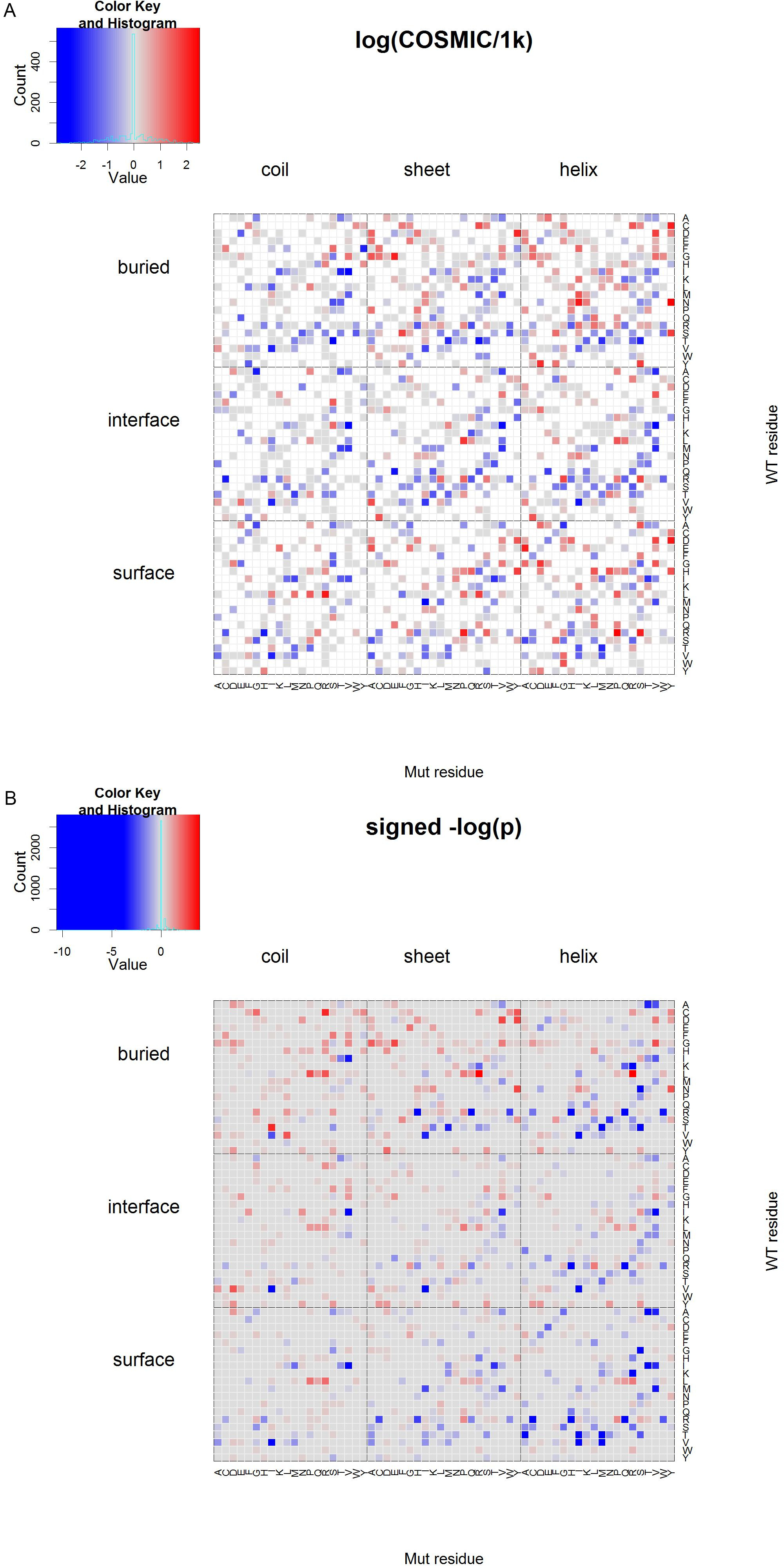

Supplement: Figure S10 — Heatmaps of normalised substitution frequencies and enrichment comparing neutral and driver mutations for mutation classes separated by area and secondary structure. Red denotes enriched classes in drivers and blue denotes enriched classes in neutral mutations. A) driver/neutral fraction of normalised frequencies for mutations by area and substitution. B) Statistically overrepresented substitution frequencies by area (s o). (TIF) [file pone.0084598.s013.tif]

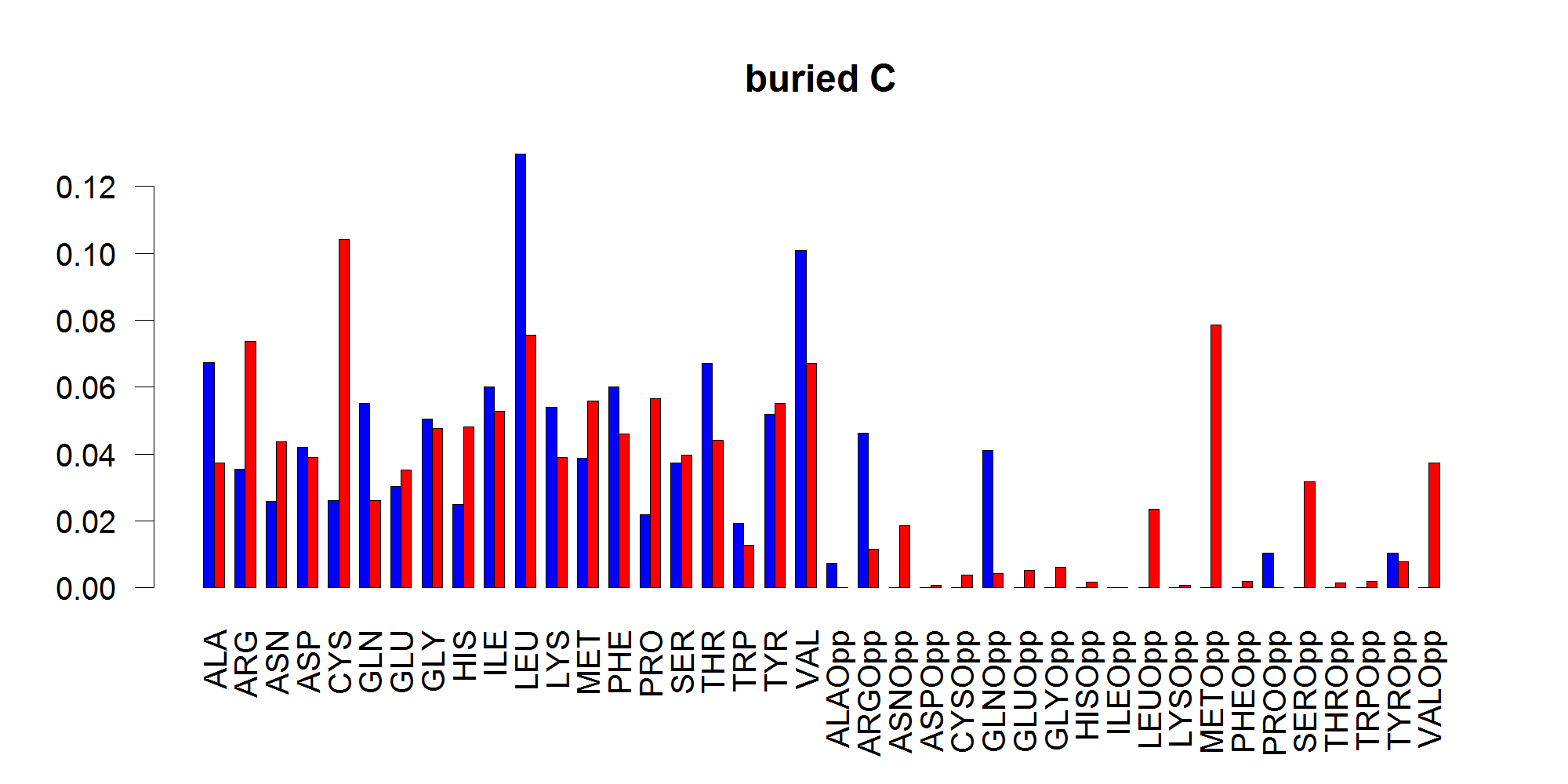

Supplement: Figure S11 — Neighbouring residue profile of targeted wild-type buried Cys mutations in the 5 Å vicinity. (TIF) [file pone.0084598.s014.tif]

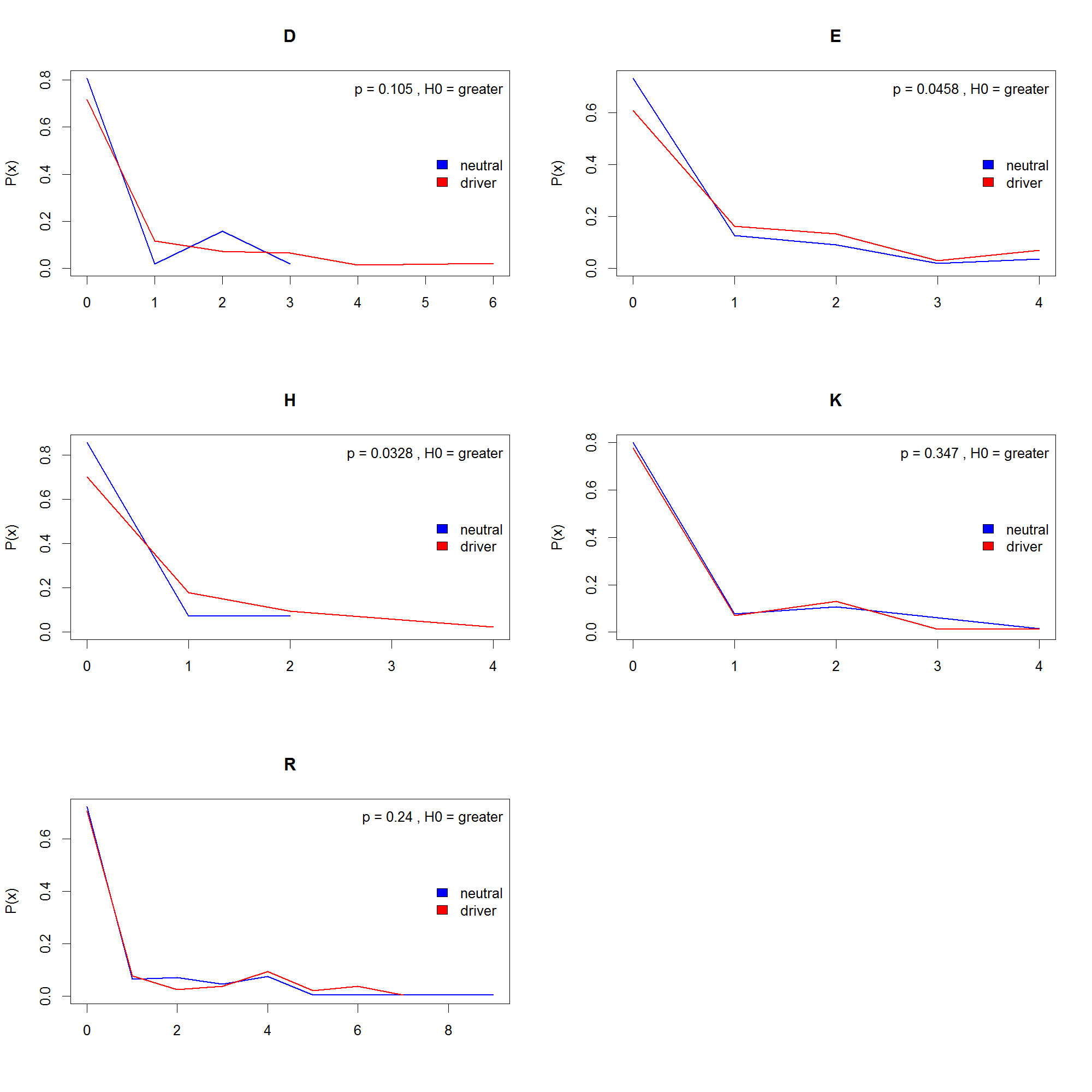

Supplement: Figure S12 — Salt bridge enrichment in interface residues for charged residues targeted by COSMIC mutations. Densities (denoted P(x)) are shown for mutations of each amino acid in both sets with their associated p-values comparing 1k and COSMIC with a two-sample Wilcoxon test using a one-sided alternative hypothesis. (TIF) [file pone.0084598.s015.tif]

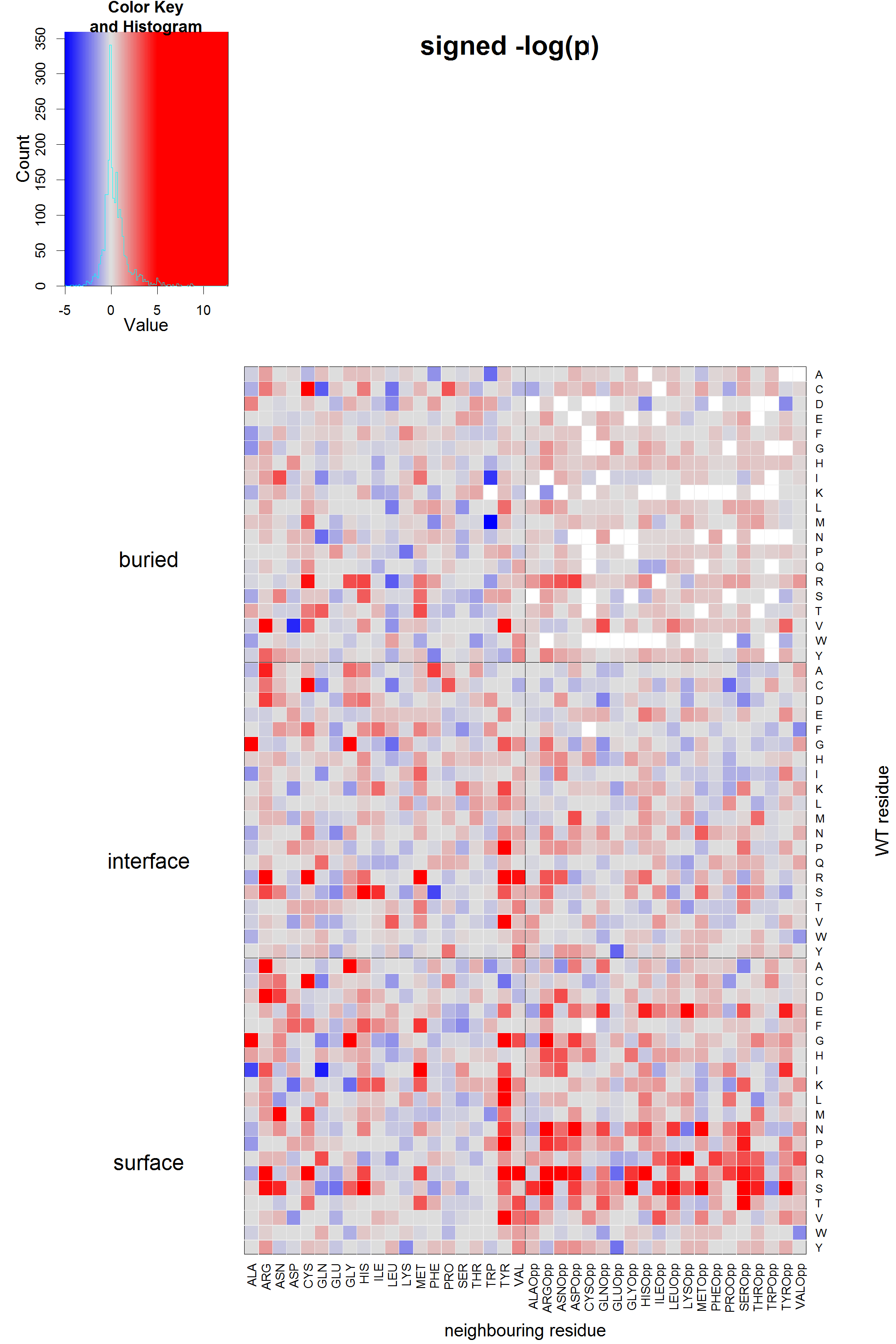

Supplement: Figure S13 — Neighbouring residue profiles of mutated interface residues in the 5 Å vicinity. The 5 Å neighbourhood profiles are shown grouped by mutated wild-type residue and by area. Shown are signed p-values using a two-sample Wilcoxon test. Red denotes enrichment in drivers and blue denotes enriched classes in neutral mutations. (TIF) [file pone.0084598.s016.tif]

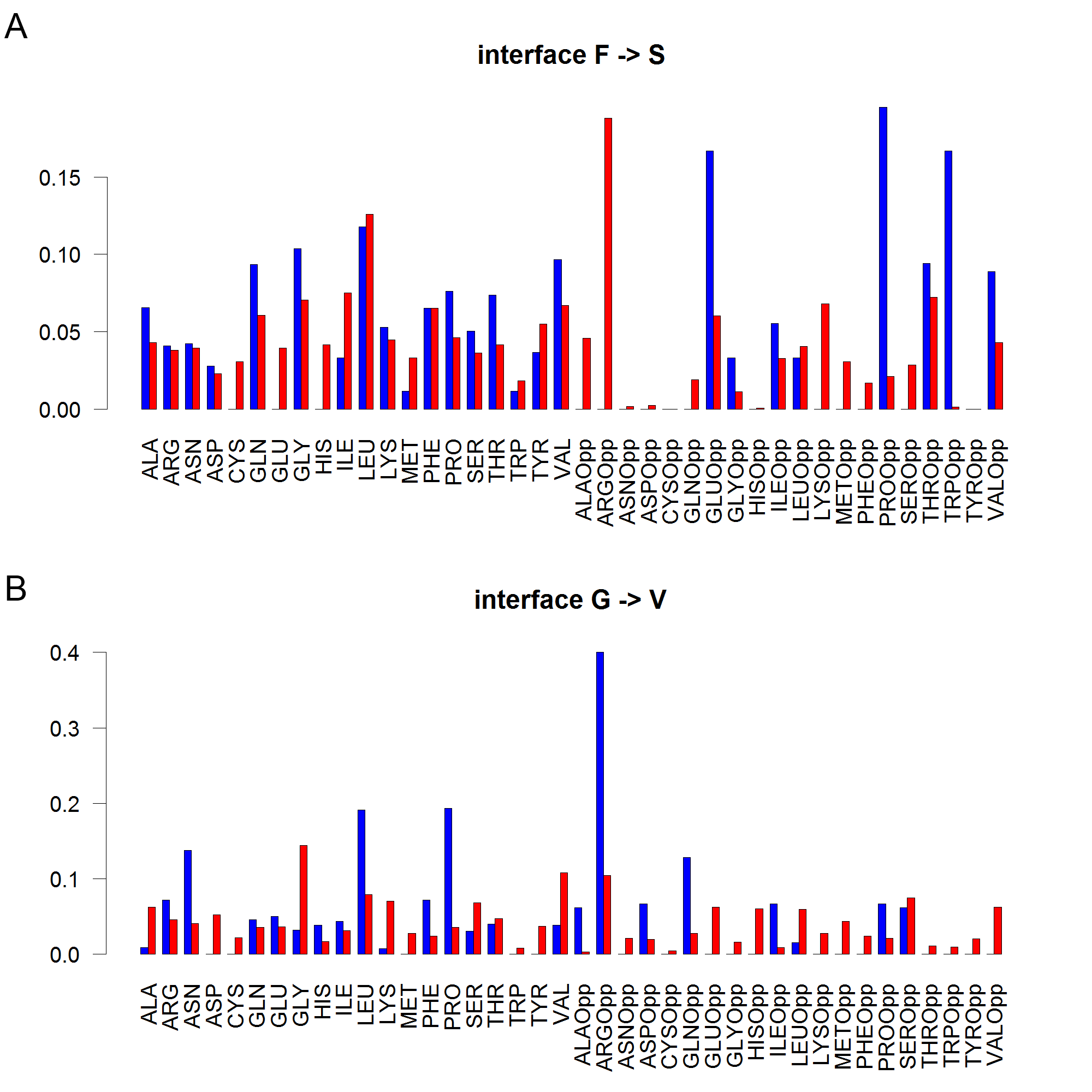

Supplement: Figure S14 — Neighbouring residue profiles for F→S mutations and G→V mutations in the 5 Å vicinity of the muatations. Profiles of neighbouring amino acid residues on the mutated side of the interface are denoted by their codes and those on the opposite side of the interface are denoted with “Opp”. Neighbouring residues of interface phenylalanine mutations in neutral mutations and drivers. Normalised (relative) frequencies (P(x)) are shown for each amino acid for 0–5 Å. The “Opp” suffix denotes the molecule on the opposite side of the interface to the mutated molecule. (TIF) [file pone.0084598.s017.tif]

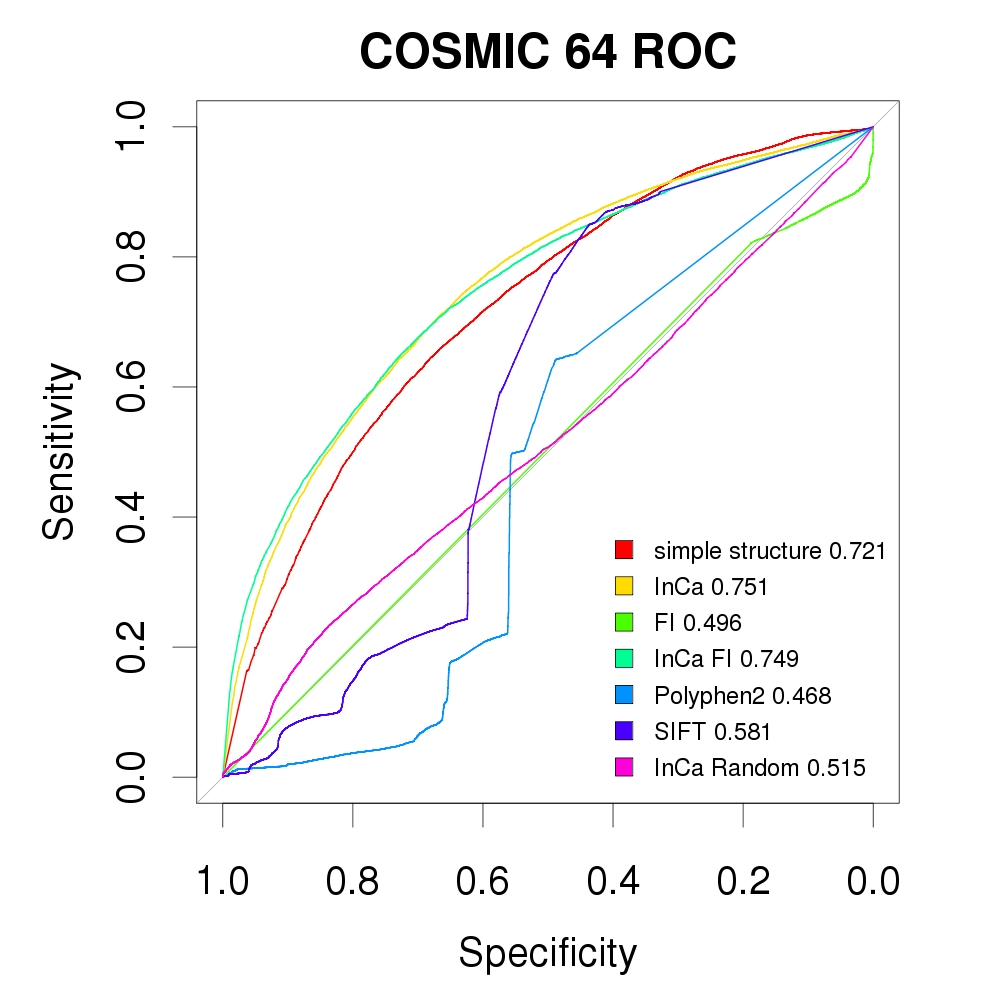

Supplement: Figure S15 — ROC curve for Inca and other mutation predictors using the reduced set of 23 proteins with both neutral and driver mutations. (TIF) [file pone.0084598.s018.tif]

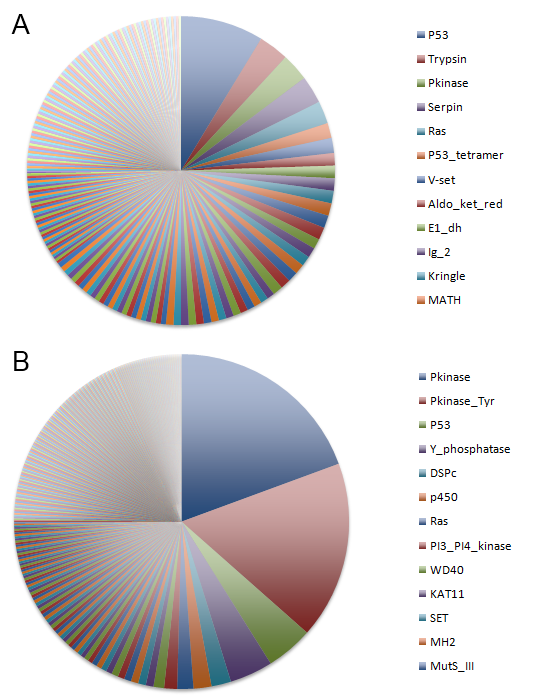

Supplement: Figure S16 — Pfam domain distribution in InCa and CHASM predicted driver mutations. A) InCa only. B) CHASM only. (TIF) [file pone.0084598.s019.tif]
