# Supplementary figures and images for: Deriving a Mutation Index of Carcinogenicity Using Protein Structure and Protein Interfaces (part 2 of 3)
Source: PLoS One. 2014 Jan 15;9(1):e84598. doi: 10.1371/journal.pone.0084598 (PMC3893166; doi:10.1371/journal.pone.0084598)

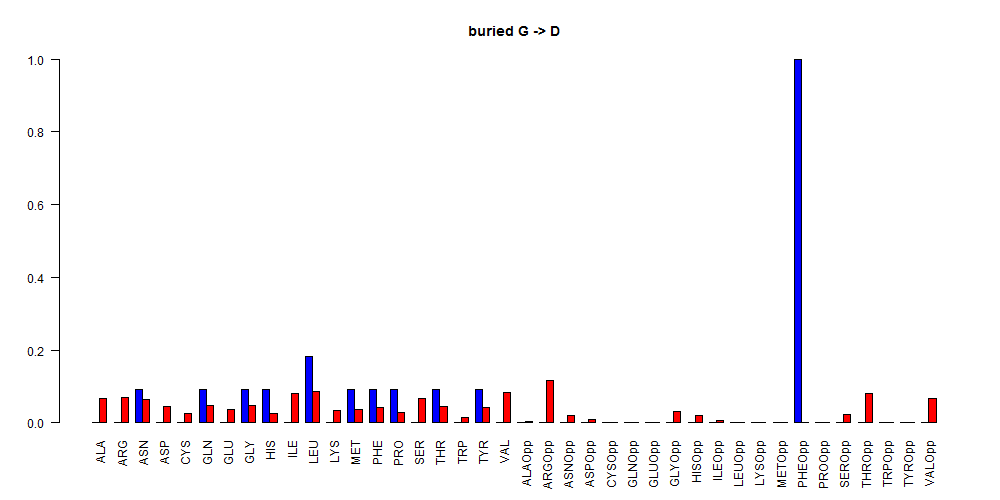

Supplement: Dataset S3 — Neighbouring residue profiles for mutations classed by substitution. (ZIP) [file pone.0084598.s003.zip › neighbour_2/buried_G_D.tif]

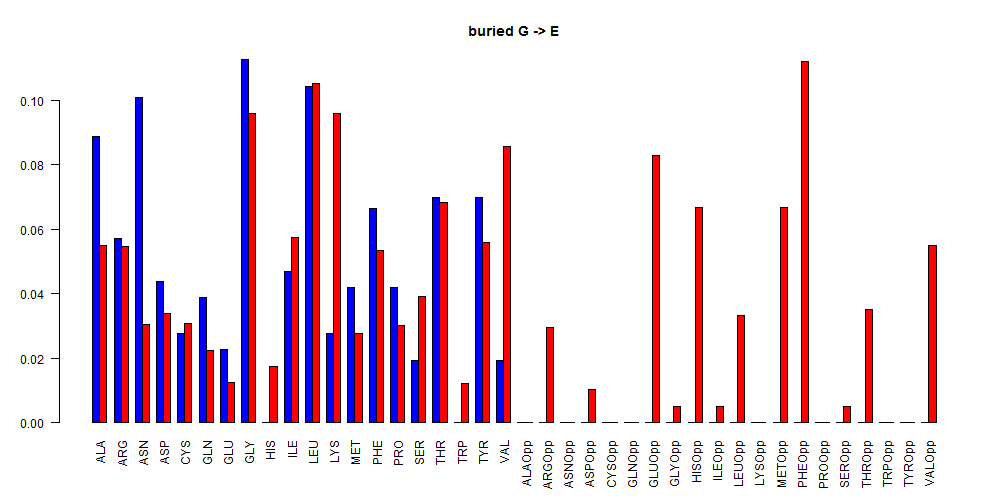

Supplement: Dataset S3 — Neighbouring residue profiles for mutations classed by substitution. (ZIP) [file pone.0084598.s003.zip › neighbour_2/buried_G_E.tif]

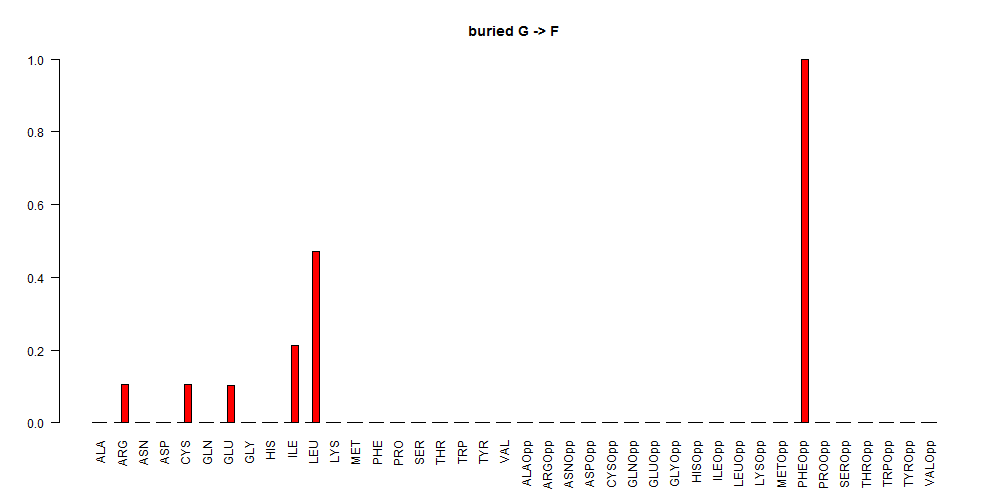

Supplement: Dataset S3 — Neighbouring residue profiles for mutations classed by substitution. (ZIP) [file pone.0084598.s003.zip › neighbour_2/buried_G_F.tif]

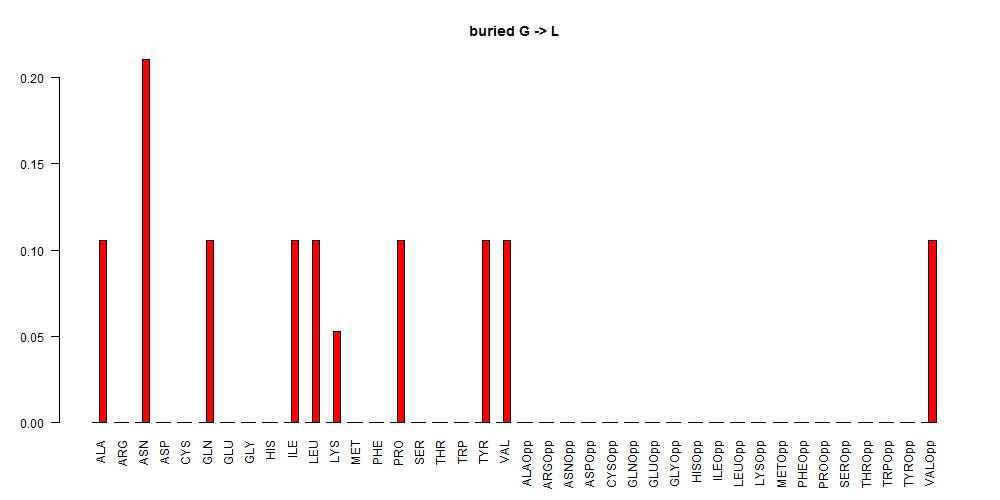

Supplement: Dataset S3 — Neighbouring residue profiles for mutations classed by substitution. (ZIP) [file pone.0084598.s003.zip › neighbour_2/buried_G_L.tif]

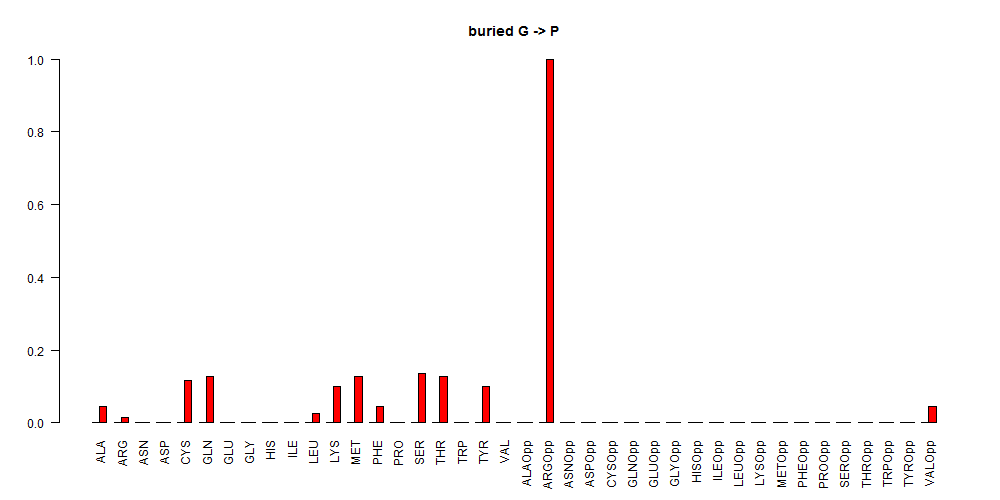

Supplement: Dataset S3 — Neighbouring residue profiles for mutations classed by substitution. (ZIP) [file pone.0084598.s003.zip › neighbour_2/buried_G_P.tif]

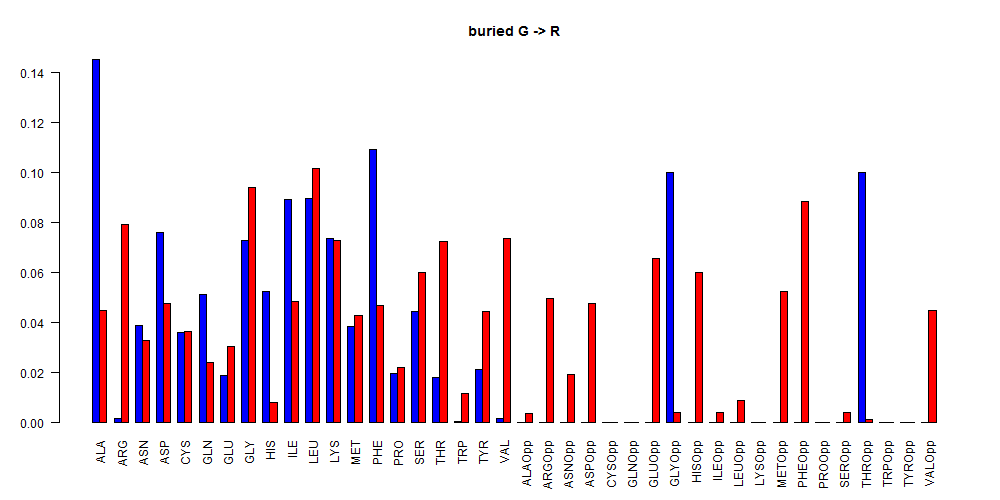

Supplement: Dataset S3 — Neighbouring residue profiles for mutations classed by substitution. (ZIP) [file pone.0084598.s003.zip › neighbour_2/buried_G_R.tif]

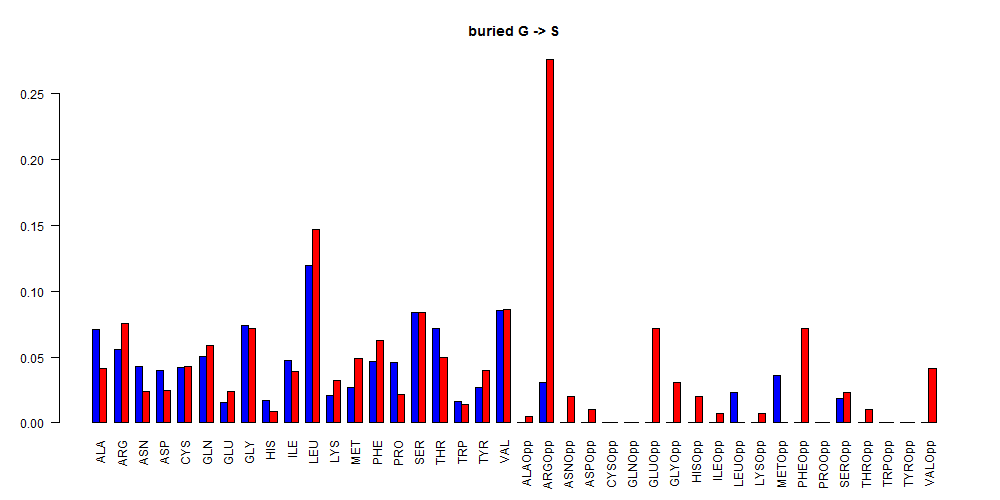

Supplement: Dataset S3 — Neighbouring residue profiles for mutations classed by substitution. (ZIP) [file pone.0084598.s003.zip › neighbour_2/buried_G_S.tif]

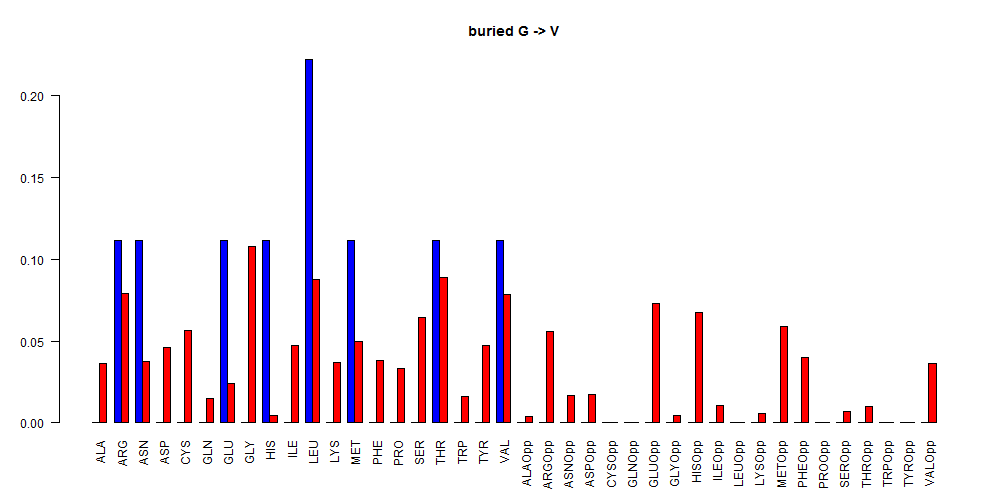

Supplement: Dataset S3 — Neighbouring residue profiles for mutations classed by substitution. (ZIP) [file pone.0084598.s003.zip › neighbour_2/buried_G_V.tif]

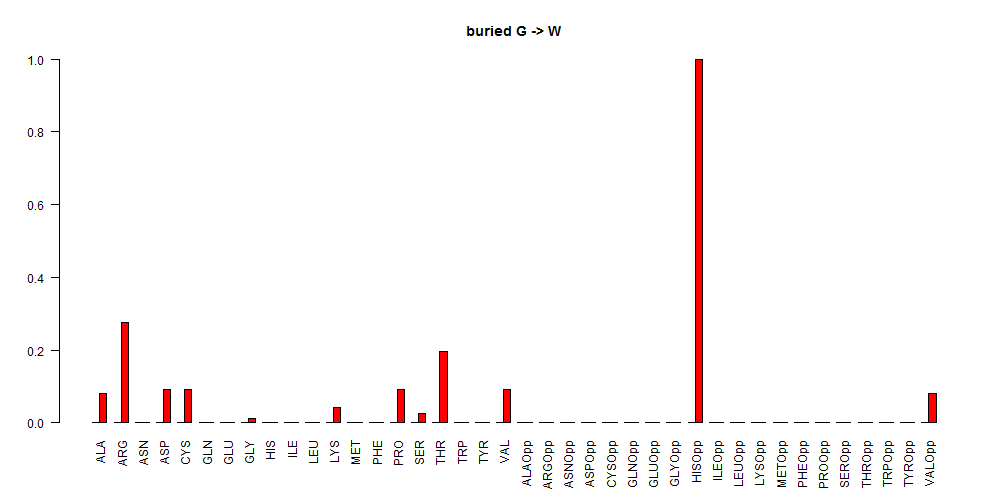

Supplement: Dataset S3 — Neighbouring residue profiles for mutations classed by substitution. (ZIP) [file pone.0084598.s003.zip › neighbour_2/buried_G_W.tif]

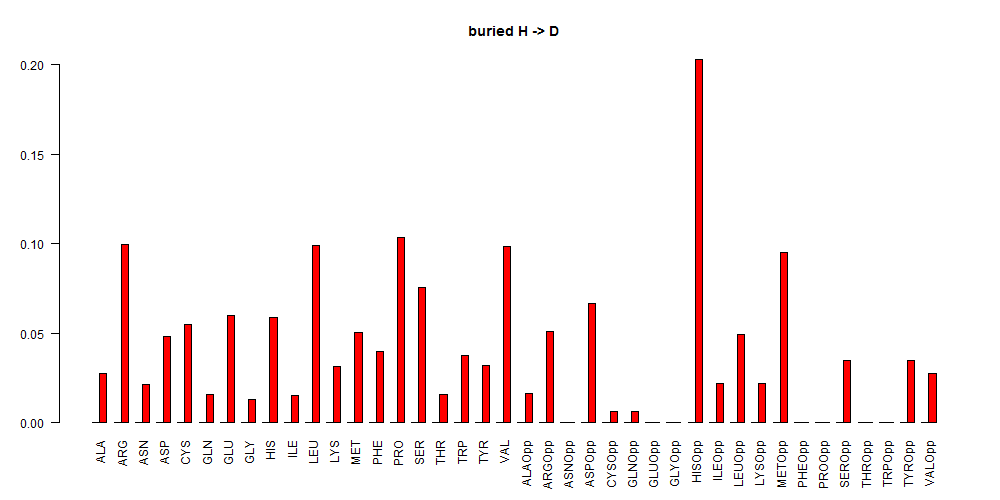

Supplement: Dataset S3 — Neighbouring residue profiles for mutations classed by substitution. (ZIP) [file pone.0084598.s003.zip › neighbour_2/buried_H_D.tif]

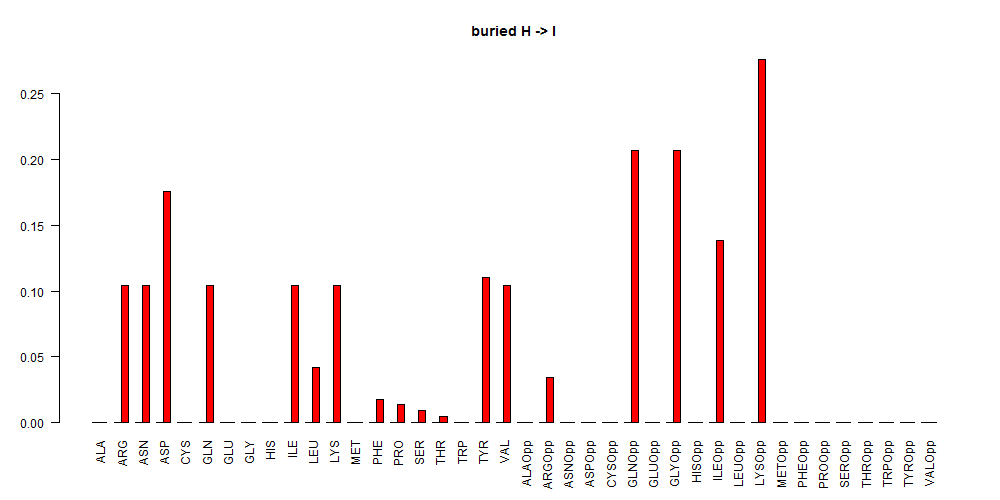

Supplement: Dataset S3 — Neighbouring residue profiles for mutations classed by substitution. (ZIP) [file pone.0084598.s003.zip › neighbour_2/buried_H_I.tif]

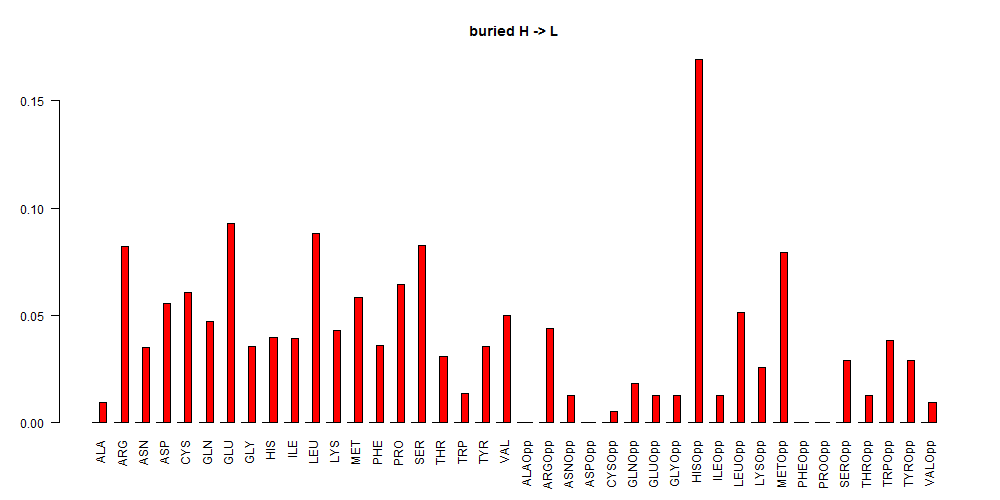

Supplement: Dataset S3 — Neighbouring residue profiles for mutations classed by substitution. (ZIP) [file pone.0084598.s003.zip › neighbour_2/buried_H_L.tif]

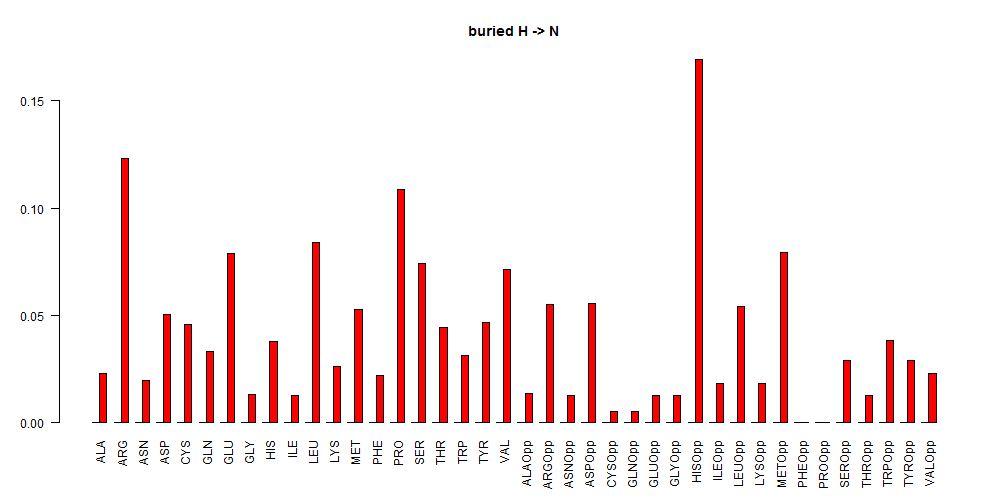

Supplement: Dataset S3 — Neighbouring residue profiles for mutations classed by substitution. (ZIP) [file pone.0084598.s003.zip › neighbour_2/buried_H_N.tif]

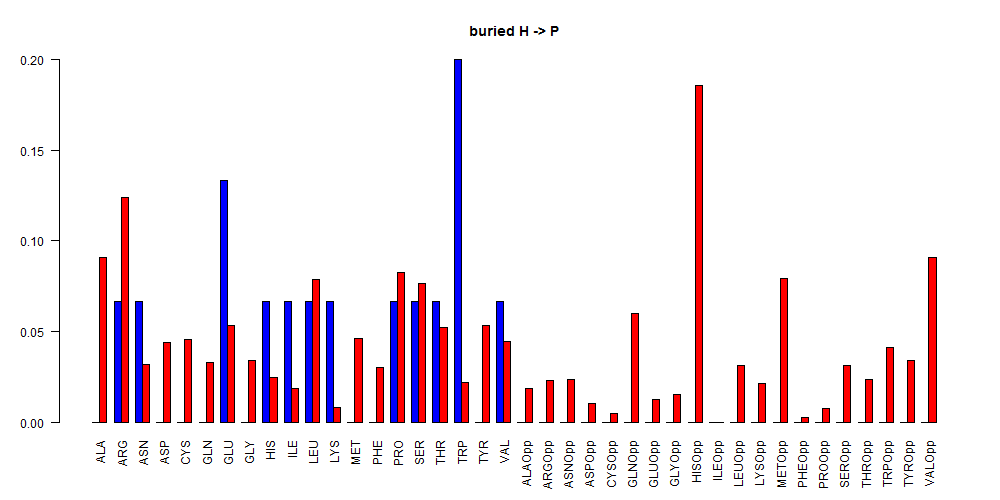

Supplement: Dataset S3 — Neighbouring residue profiles for mutations classed by substitution. (ZIP) [file pone.0084598.s003.zip › neighbour_2/buried_H_P.tif]

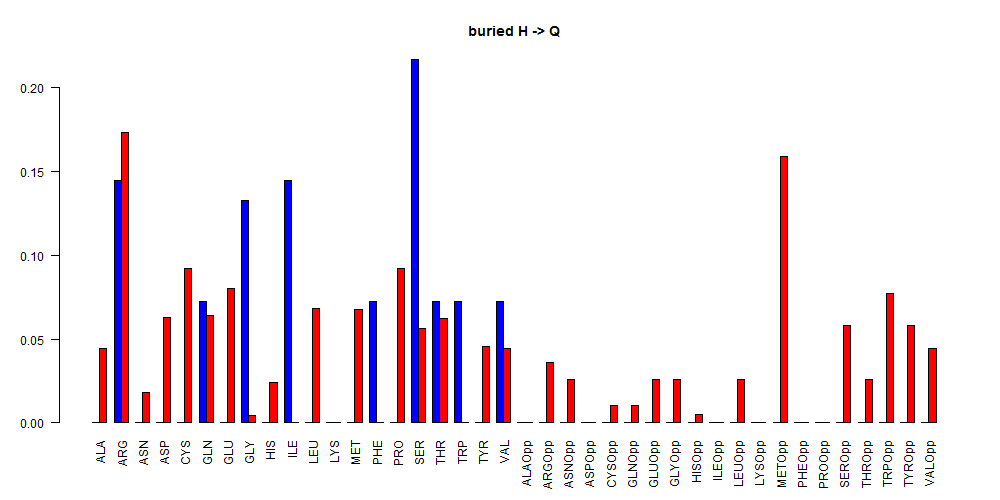

Supplement: Dataset S3 — Neighbouring residue profiles for mutations classed by substitution. (ZIP) [file pone.0084598.s003.zip › neighbour_2/buried_H_Q.tif]

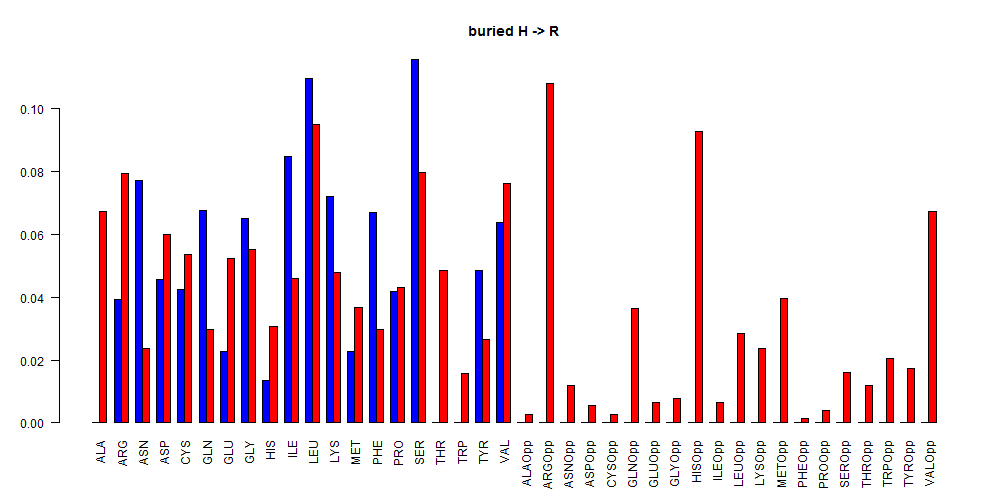

Supplement: Dataset S3 — Neighbouring residue profiles for mutations classed by substitution. (ZIP) [file pone.0084598.s003.zip › neighbour_2/buried_H_R.tif]

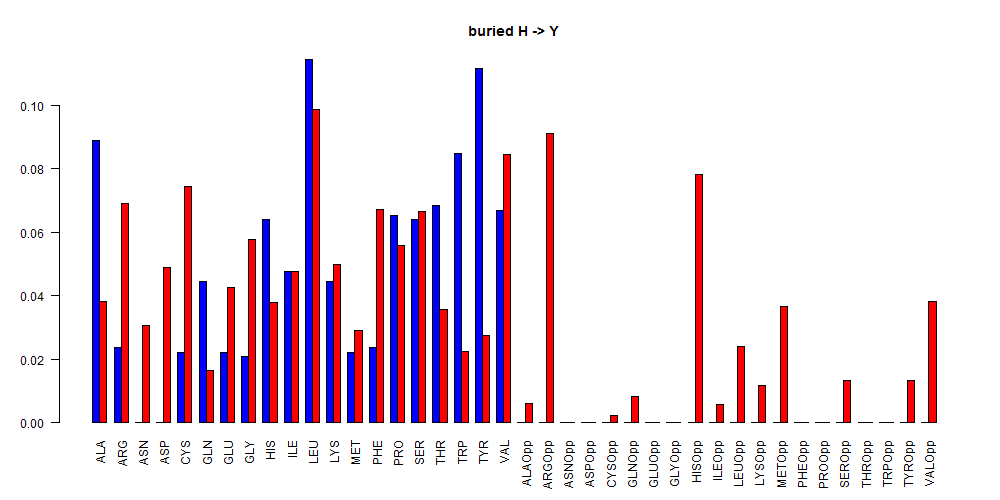

Supplement: Dataset S3 — Neighbouring residue profiles for mutations classed by substitution. (ZIP) [file pone.0084598.s003.zip › neighbour_2/buried_H_Y.tif]

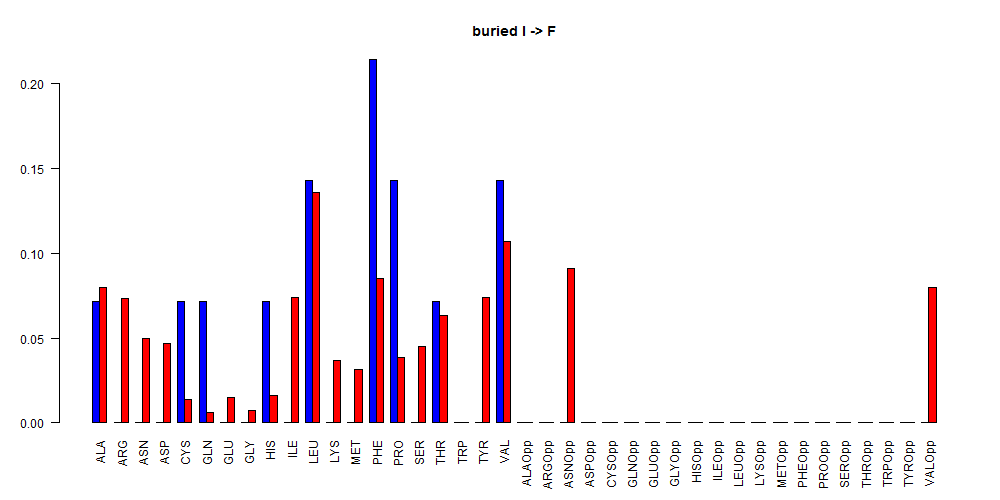

Supplement: Dataset S3 — Neighbouring residue profiles for mutations classed by substitution. (ZIP) [file pone.0084598.s003.zip › neighbour_2/buried_I_F.tif]

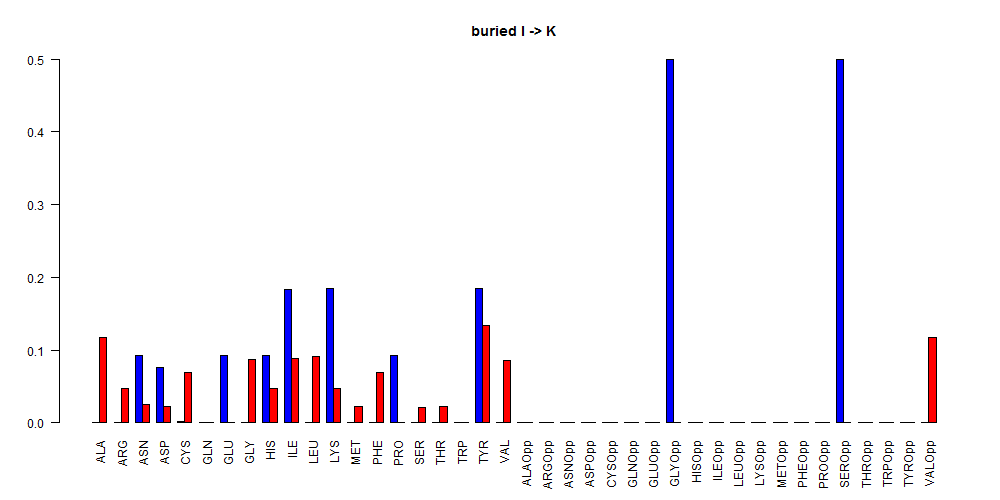

Supplement: Dataset S3 — Neighbouring residue profiles for mutations classed by substitution. (ZIP) [file pone.0084598.s003.zip › neighbour_2/buried_I_K.tif]

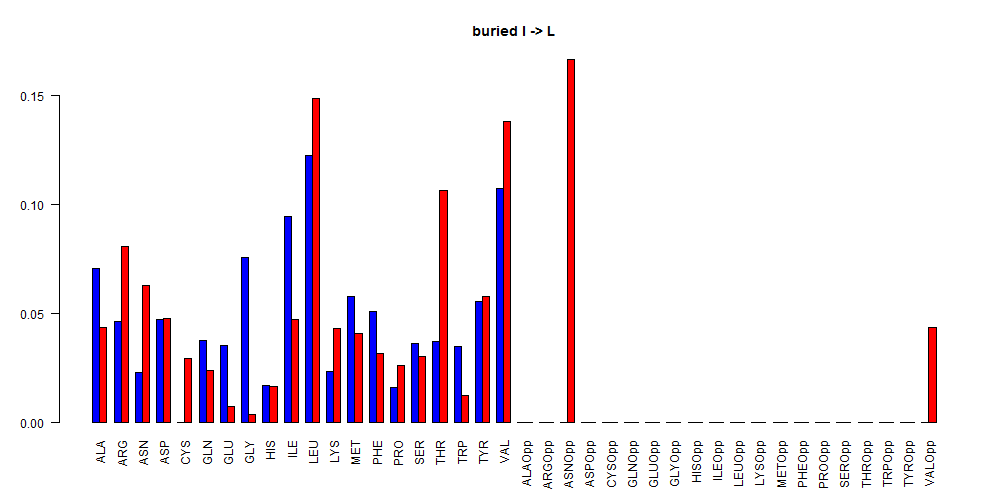

Supplement: Dataset S3 — Neighbouring residue profiles for mutations classed by substitution. (ZIP) [file pone.0084598.s003.zip › neighbour_2/buried_I_L.tif]

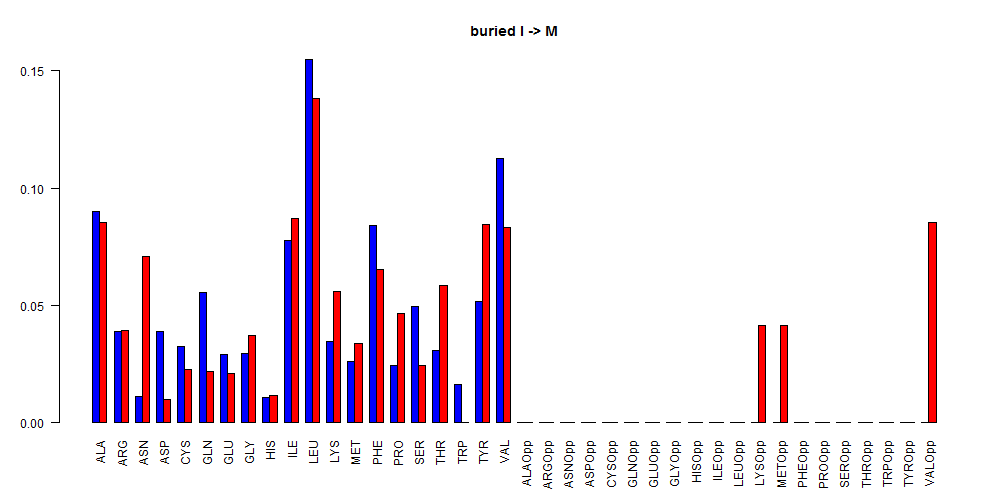

Supplement: Dataset S3 — Neighbouring residue profiles for mutations classed by substitution. (ZIP) [file pone.0084598.s003.zip › neighbour_2/buried_I_M.tif]

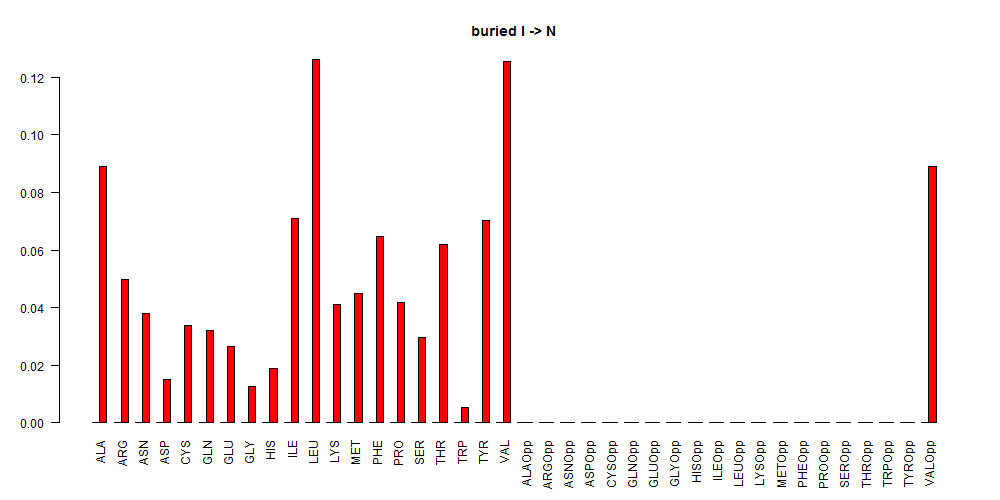

Supplement: Dataset S3 — Neighbouring residue profiles for mutations classed by substitution. (ZIP) [file pone.0084598.s003.zip › neighbour_2/buried_I_N.tif]

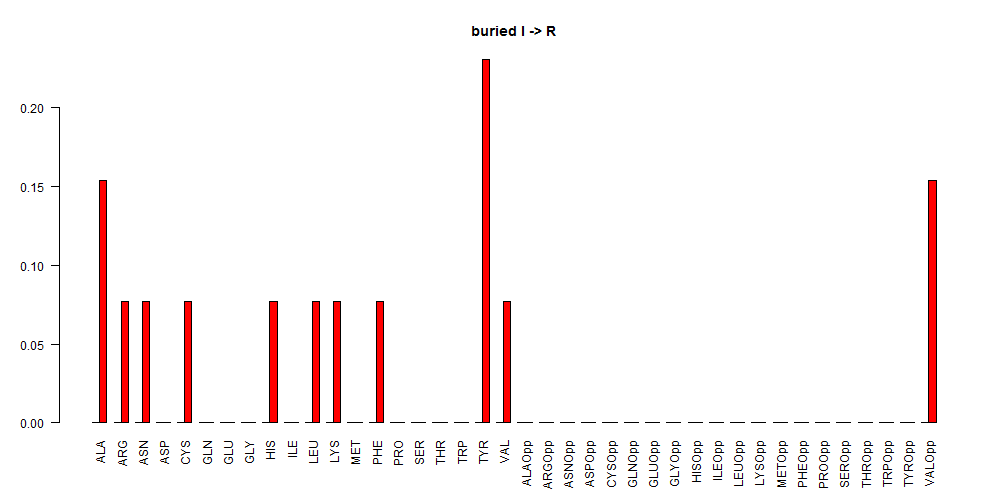

Supplement: Dataset S3 — Neighbouring residue profiles for mutations classed by substitution. (ZIP) [file pone.0084598.s003.zip › neighbour_2/buried_I_R.tif]

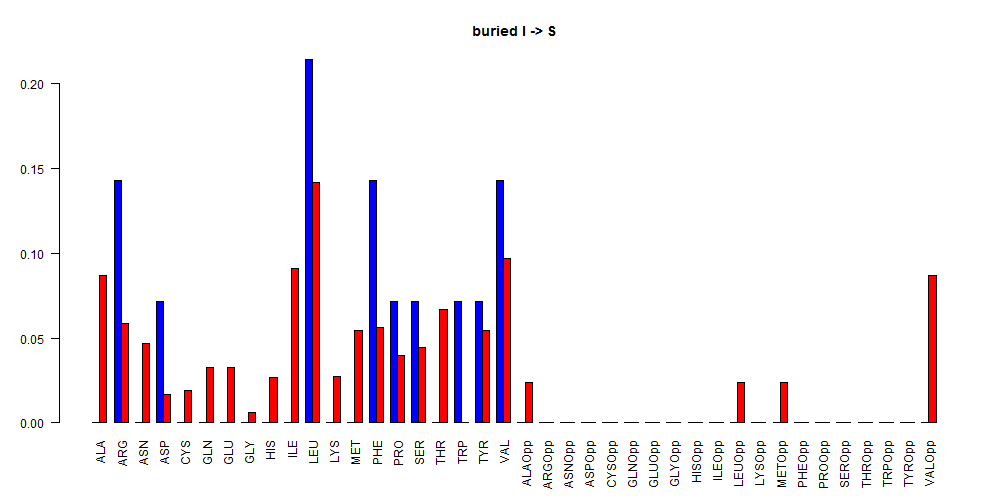

Supplement: Dataset S3 — Neighbouring residue profiles for mutations classed by substitution. (ZIP) [file pone.0084598.s003.zip › neighbour_2/buried_I_S.tif]

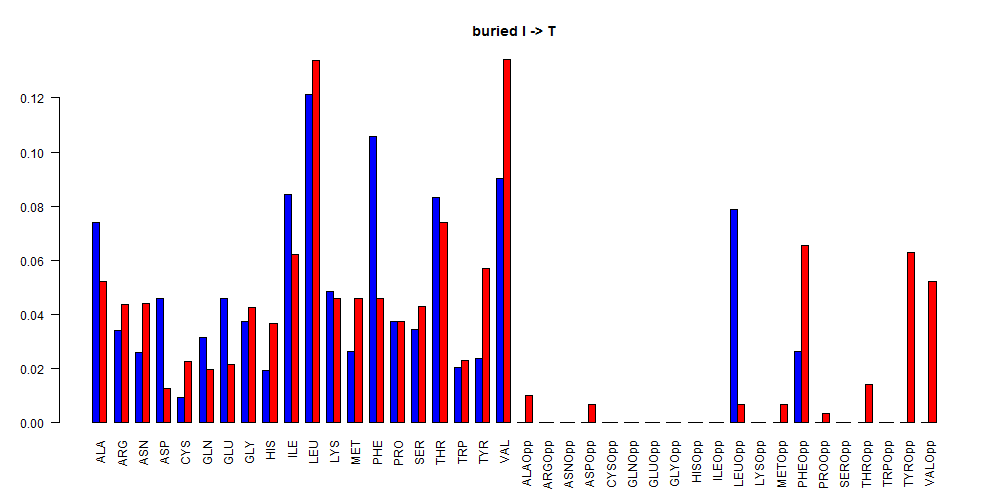

Supplement: Dataset S3 — Neighbouring residue profiles for mutations classed by substitution. (ZIP) [file pone.0084598.s003.zip › neighbour_2/buried_I_T.tif]

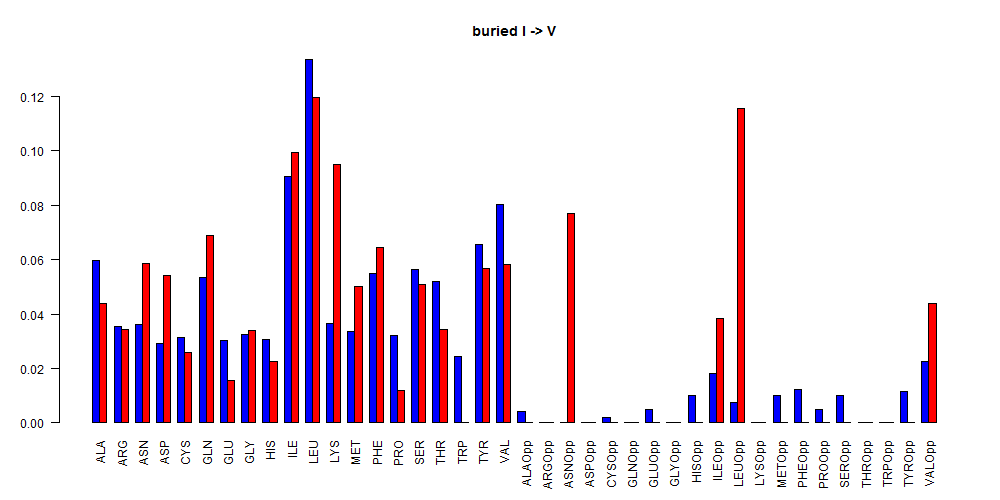

Supplement: Dataset S3 — Neighbouring residue profiles for mutations classed by substitution. (ZIP) [file pone.0084598.s003.zip › neighbour_2/buried_I_V.tif]

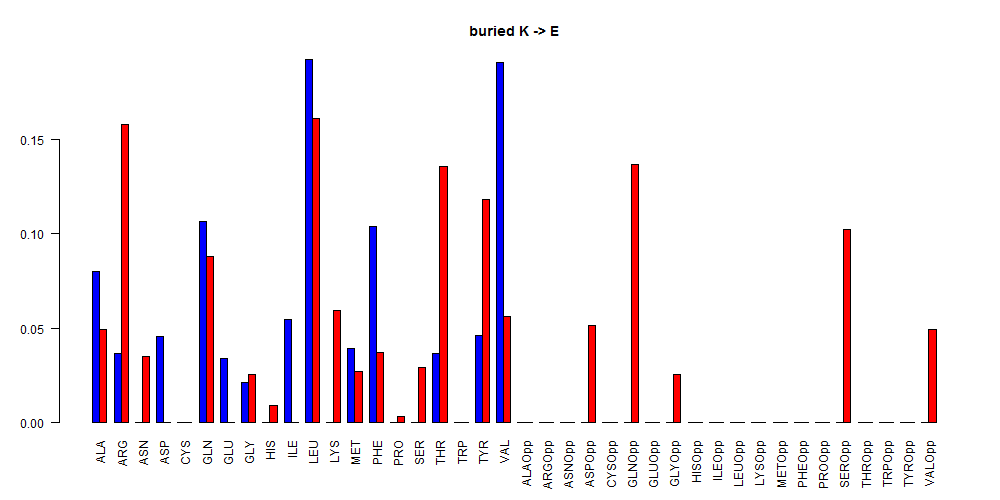

Supplement: Dataset S3 — Neighbouring residue profiles for mutations classed by substitution. (ZIP) [file pone.0084598.s003.zip › neighbour_2/buried_K_E.tif]

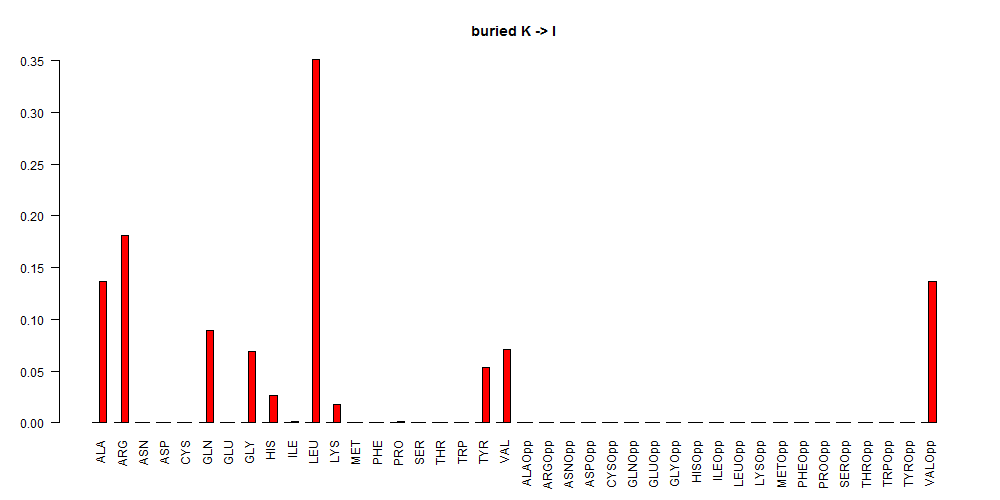

Supplement: Dataset S3 — Neighbouring residue profiles for mutations classed by substitution. (ZIP) [file pone.0084598.s003.zip › neighbour_2/buried_K_I.tif]

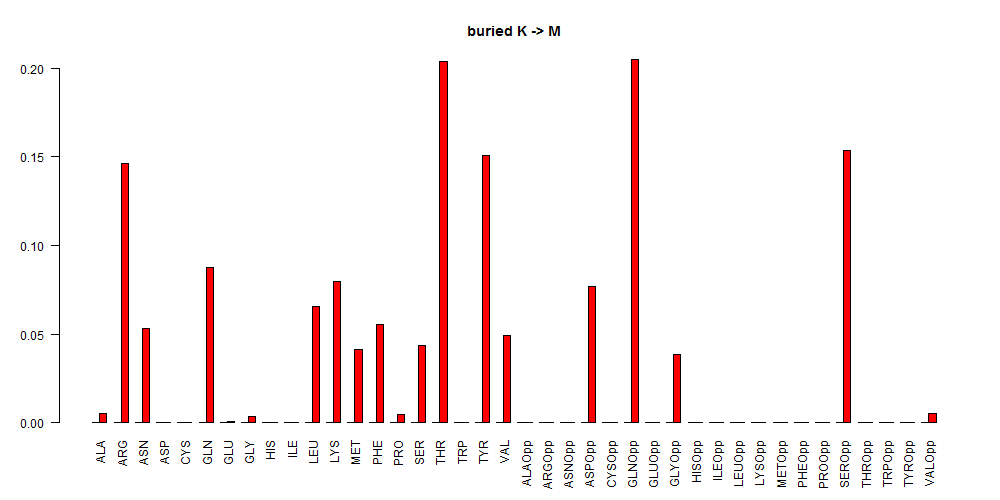

Supplement: Dataset S3 — Neighbouring residue profiles for mutations classed by substitution. (ZIP) [file pone.0084598.s003.zip › neighbour_2/buried_K_M.tif]

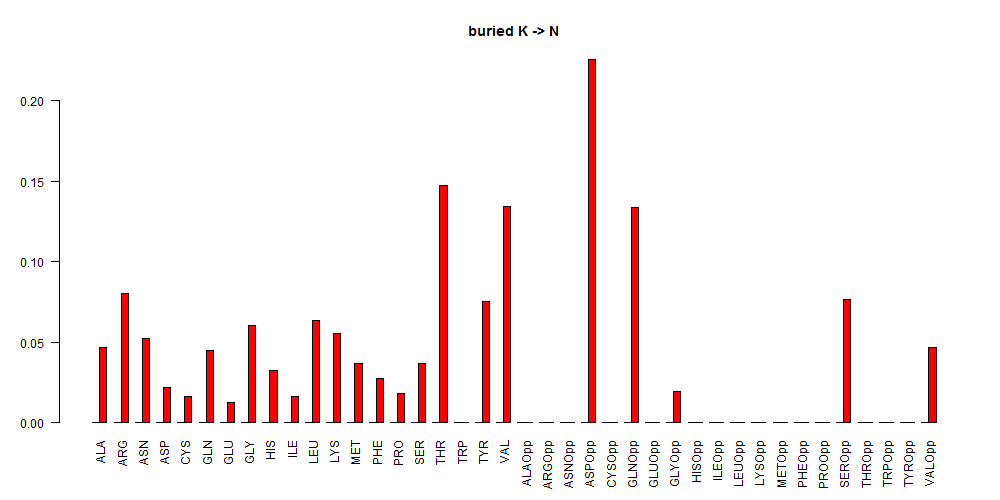

Supplement: Dataset S3 — Neighbouring residue profiles for mutations classed by substitution. (ZIP) [file pone.0084598.s003.zip › neighbour_2/buried_K_N.tif]

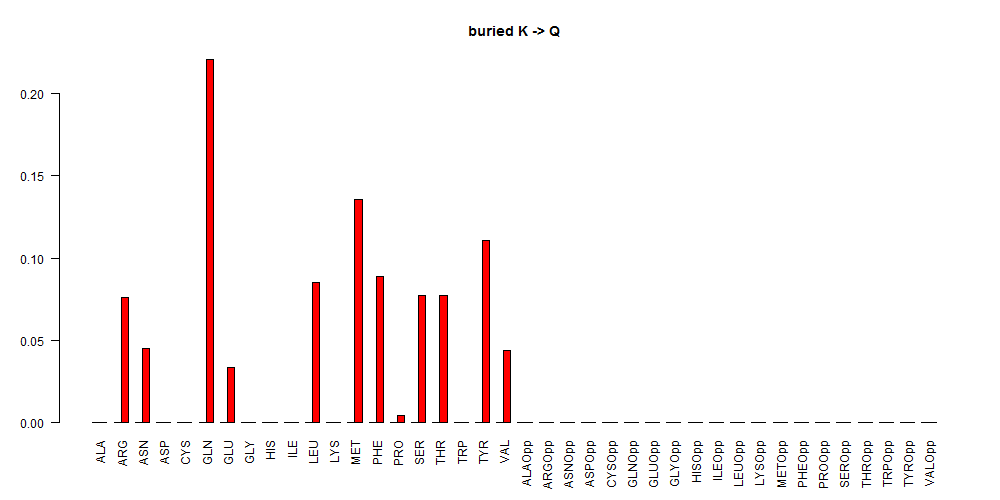

Supplement: Dataset S3 — Neighbouring residue profiles for mutations classed by substitution. (ZIP) [file pone.0084598.s003.zip › neighbour_2/buried_K_Q.tif]

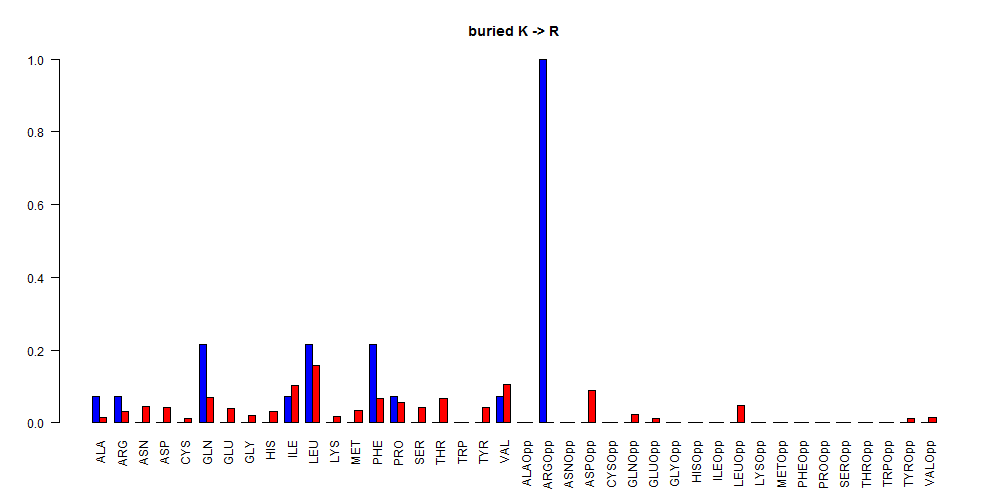

Supplement: Dataset S3 — Neighbouring residue profiles for mutations classed by substitution. (ZIP) [file pone.0084598.s003.zip › neighbour_2/buried_K_R.tif]

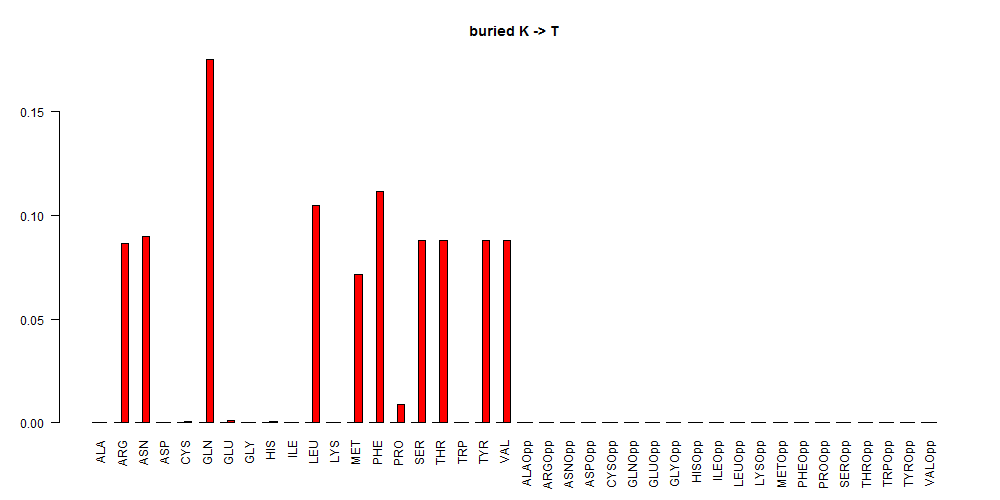

Supplement: Dataset S3 — Neighbouring residue profiles for mutations classed by substitution. (ZIP) [file pone.0084598.s003.zip › neighbour_2/buried_K_T.tif]

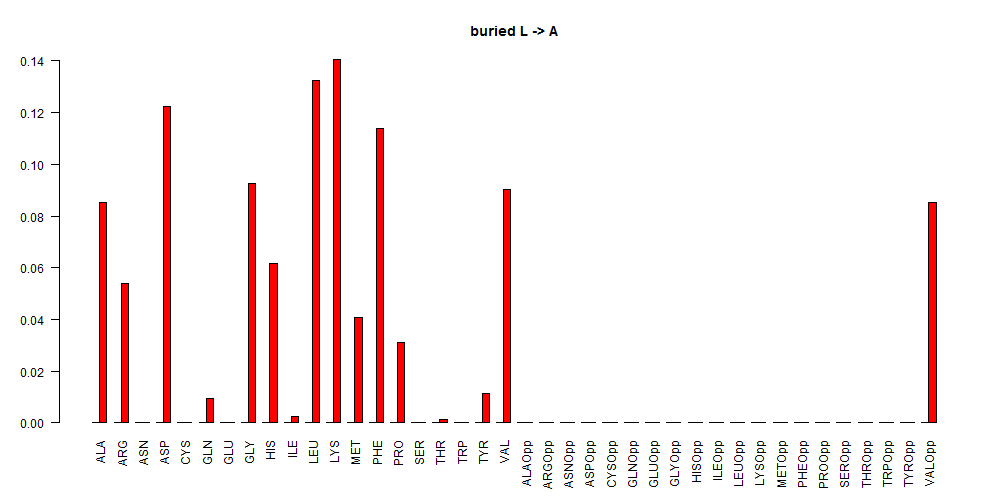

Supplement: Dataset S3 — Neighbouring residue profiles for mutations classed by substitution. (ZIP) [file pone.0084598.s003.zip › neighbour_2/buried_L_A.tif]

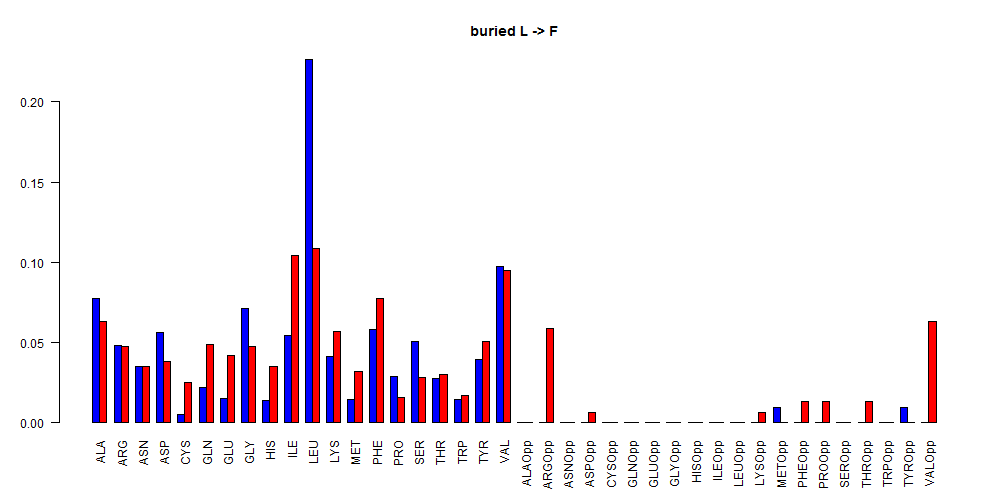

Supplement: Dataset S3 — Neighbouring residue profiles for mutations classed by substitution. (ZIP) [file pone.0084598.s003.zip › neighbour_2/buried_L_F.tif]

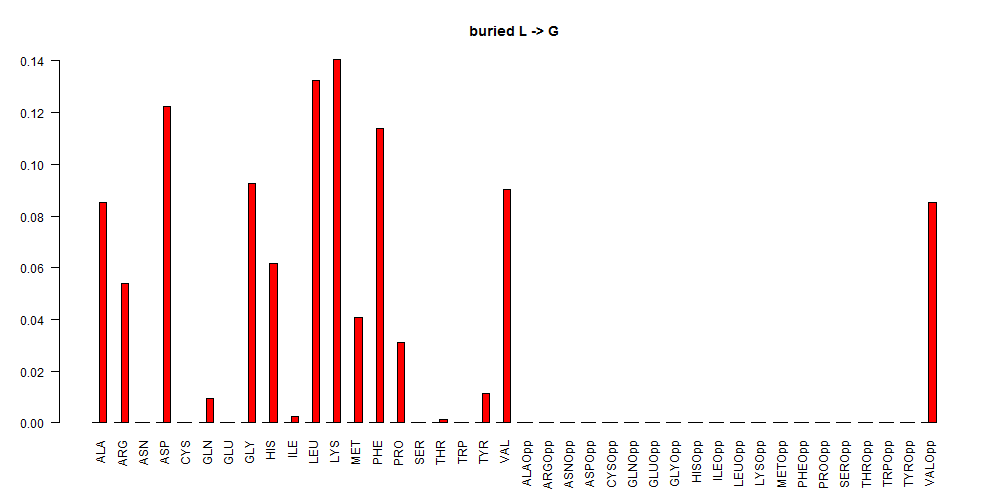

Supplement: Dataset S3 — Neighbouring residue profiles for mutations classed by substitution. (ZIP) [file pone.0084598.s003.zip › neighbour_2/buried_L_G.tif]

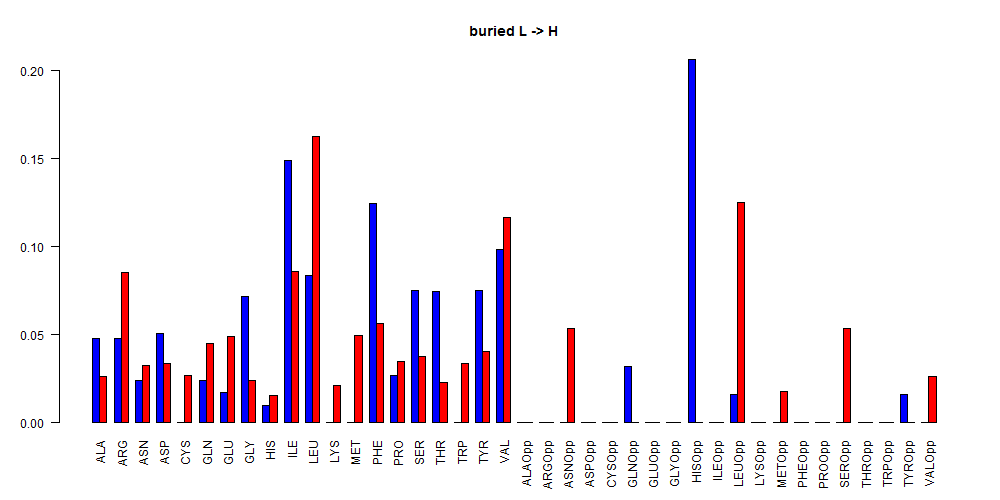

Supplement: Dataset S3 — Neighbouring residue profiles for mutations classed by substitution. (ZIP) [file pone.0084598.s003.zip › neighbour_2/buried_L_H.tif]

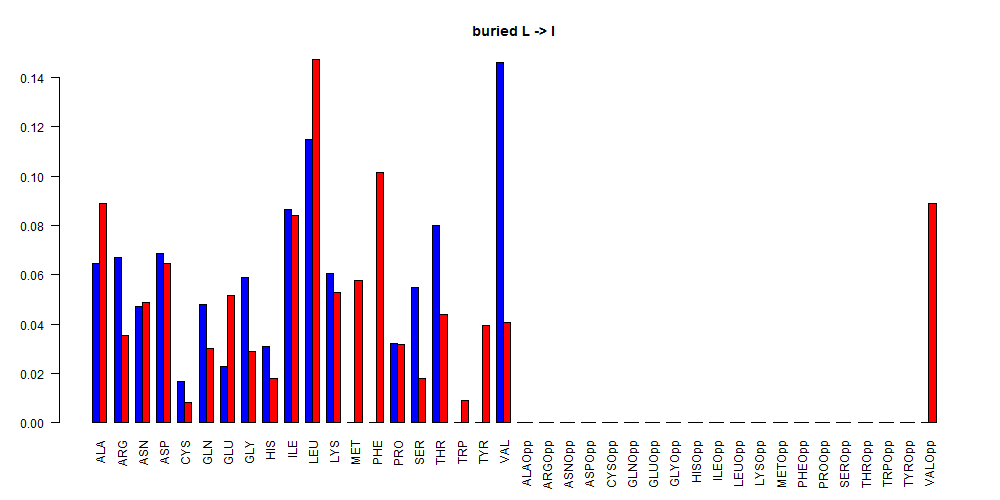

Supplement: Dataset S3 — Neighbouring residue profiles for mutations classed by substitution. (ZIP) [file pone.0084598.s003.zip › neighbour_2/buried_L_I.tif]

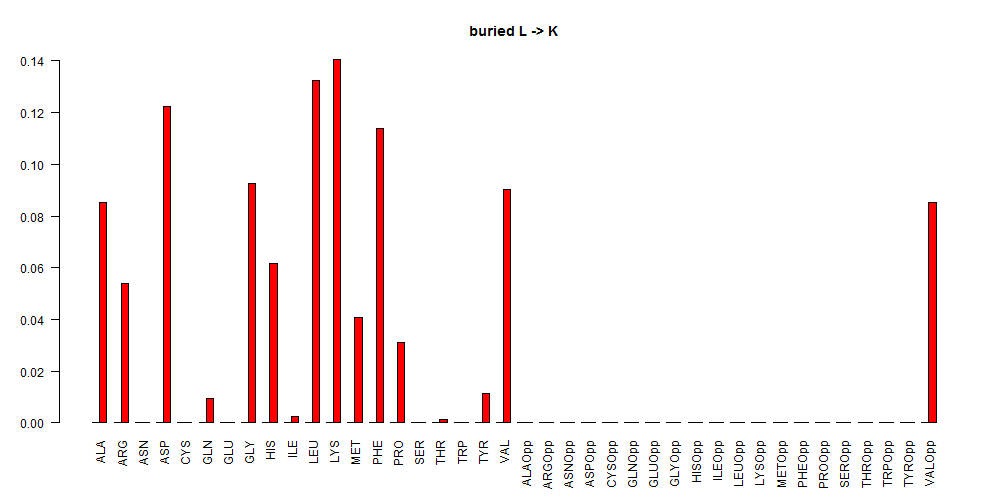

Supplement: Dataset S3 — Neighbouring residue profiles for mutations classed by substitution. (ZIP) [file pone.0084598.s003.zip › neighbour_2/buried_L_K.tif]

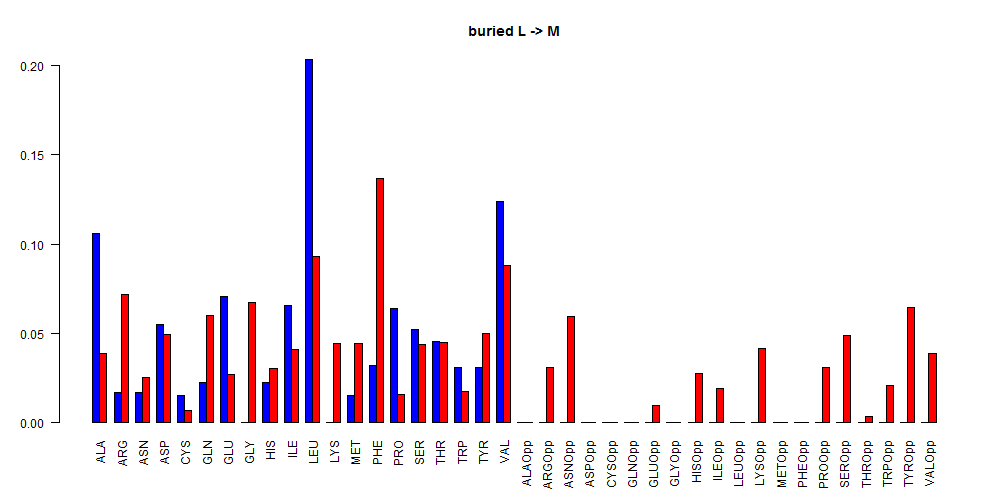

Supplement: Dataset S3 — Neighbouring residue profiles for mutations classed by substitution. (ZIP) [file pone.0084598.s003.zip › neighbour_2/buried_L_M.tif]

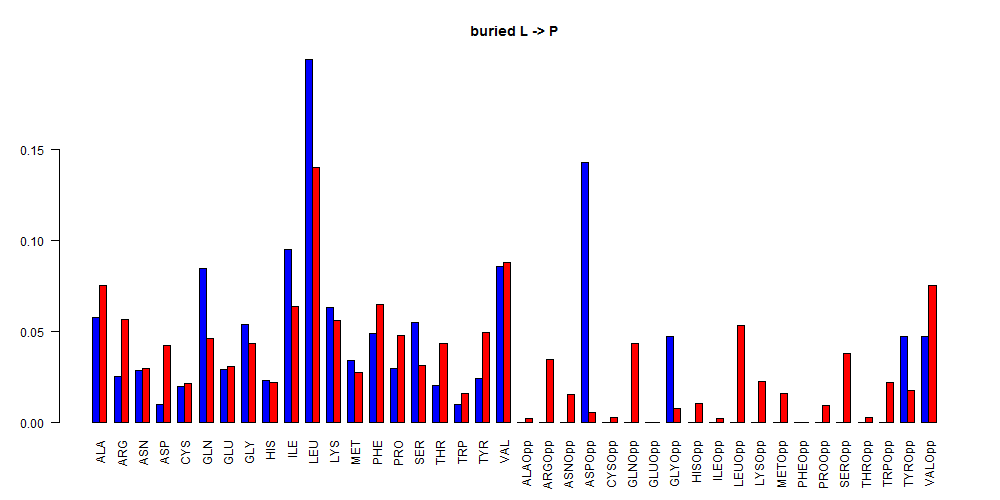

Supplement: Dataset S3 — Neighbouring residue profiles for mutations classed by substitution. (ZIP) [file pone.0084598.s003.zip › neighbour_2/buried_L_P.tif]

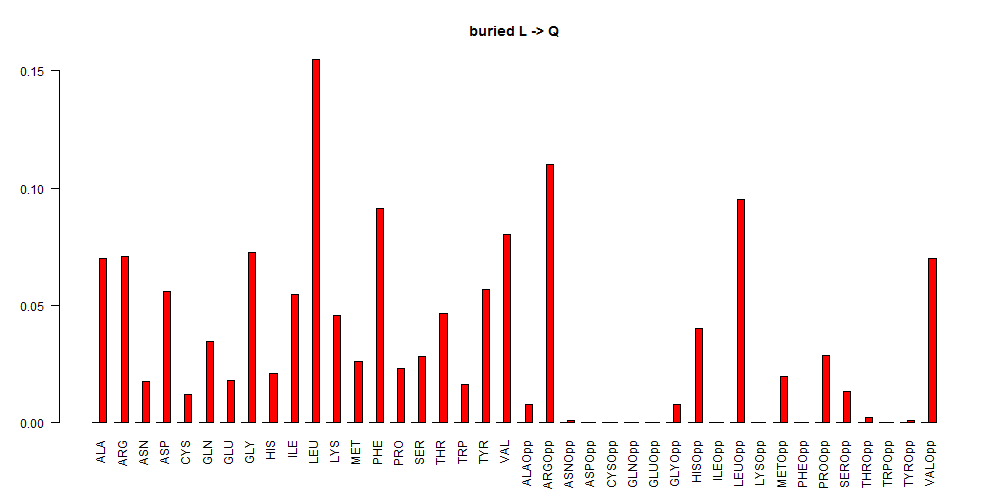

Supplement: Dataset S3 — Neighbouring residue profiles for mutations classed by substitution. (ZIP) [file pone.0084598.s003.zip › neighbour_2/buried_L_Q.tif]

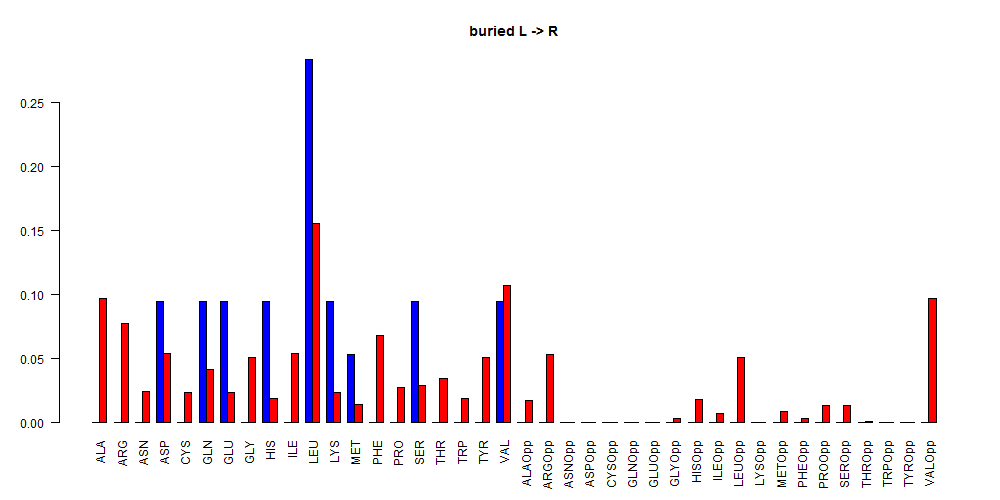

Supplement: Dataset S3 — Neighbouring residue profiles for mutations classed by substitution. (ZIP) [file pone.0084598.s003.zip › neighbour_2/buried_L_R.tif]

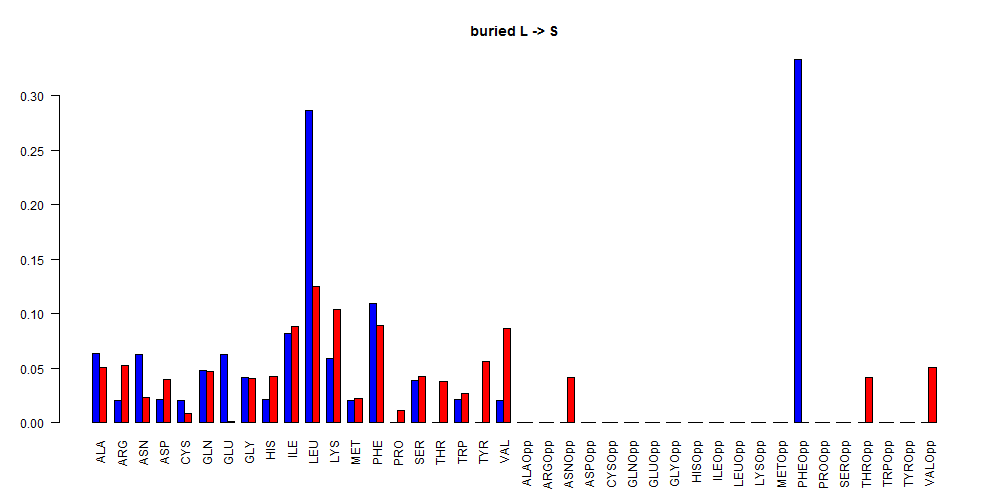

Supplement: Dataset S3 — Neighbouring residue profiles for mutations classed by substitution. (ZIP) [file pone.0084598.s003.zip › neighbour_2/buried_L_S.tif]

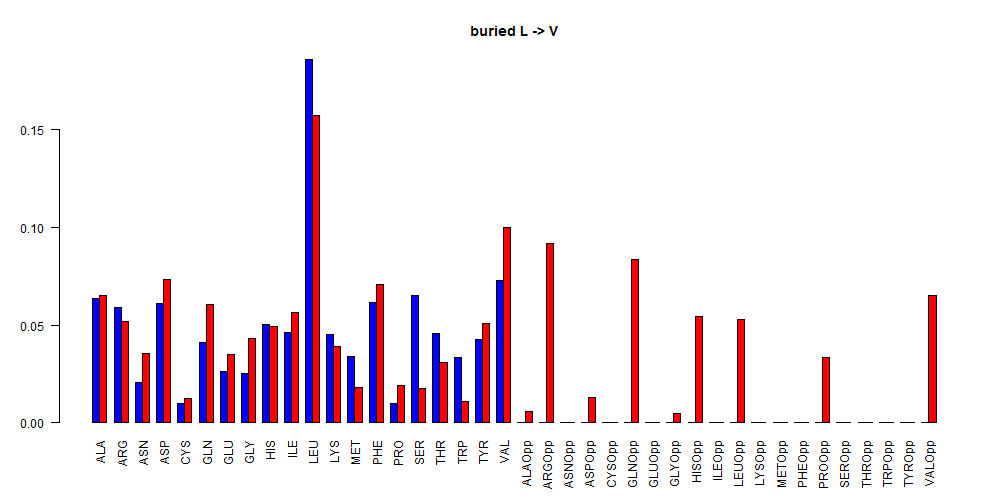

Supplement: Dataset S3 — Neighbouring residue profiles for mutations classed by substitution. (ZIP) [file pone.0084598.s003.zip › neighbour_2/buried_L_V.tif]

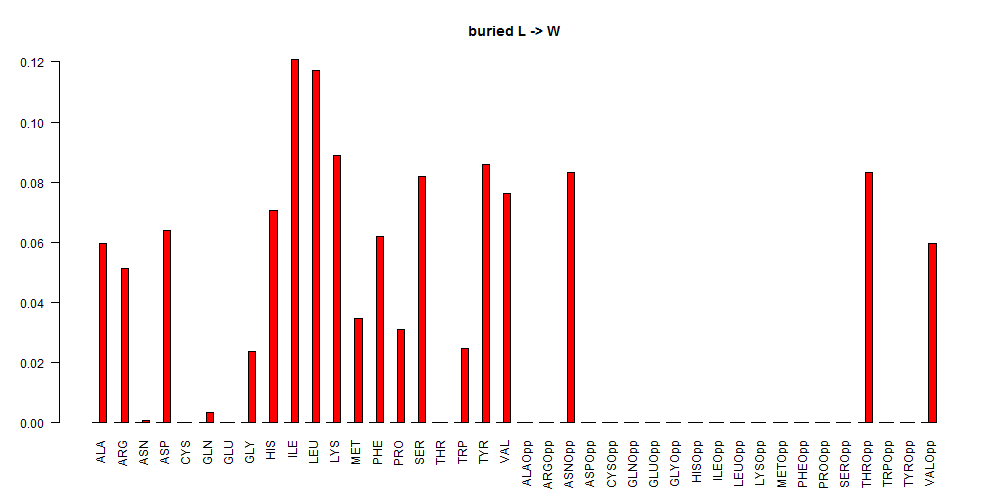

Supplement: Dataset S3 — Neighbouring residue profiles for mutations classed by substitution. (ZIP) [file pone.0084598.s003.zip › neighbour_2/buried_L_W.tif]

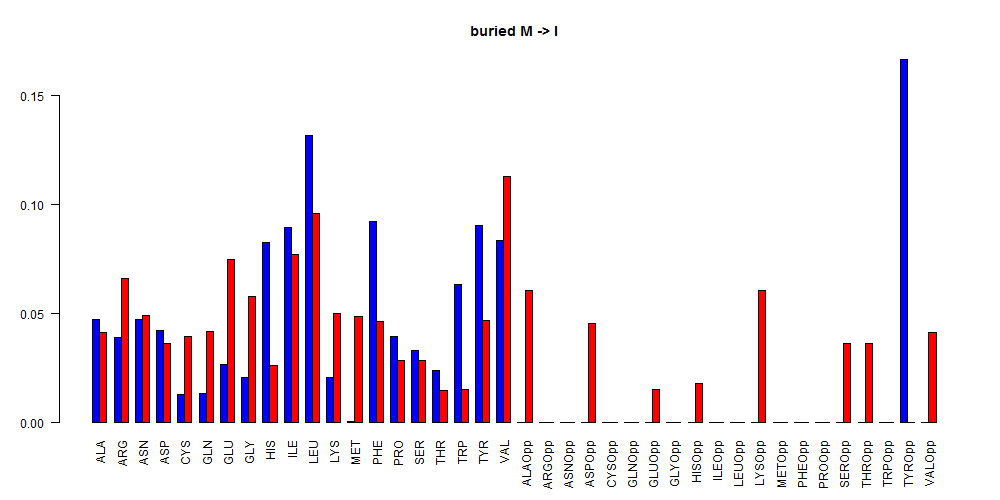

Supplement: Dataset S3 — Neighbouring residue profiles for mutations classed by substitution. (ZIP) [file pone.0084598.s003.zip › neighbour_2/buried_M_I.tif]

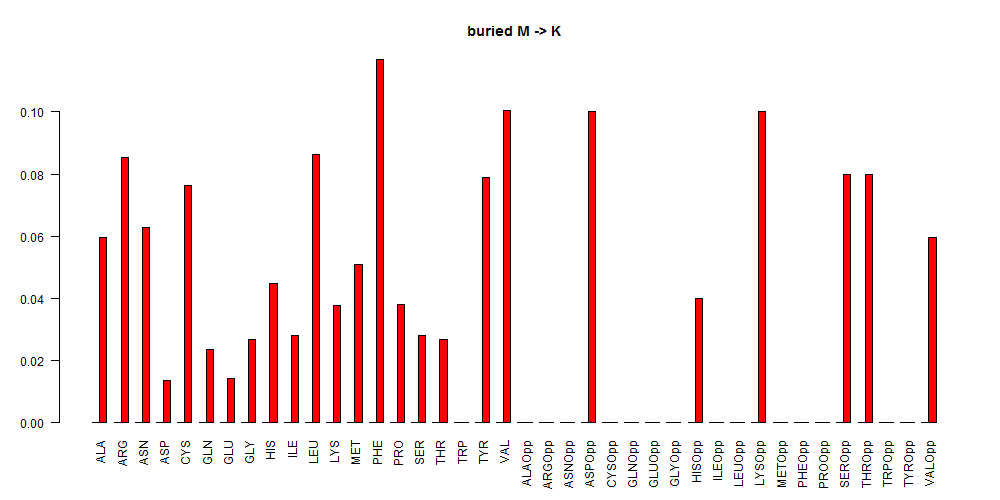

Supplement: Dataset S3 — Neighbouring residue profiles for mutations classed by substitution. (ZIP) [file pone.0084598.s003.zip › neighbour_2/buried_M_K.tif]

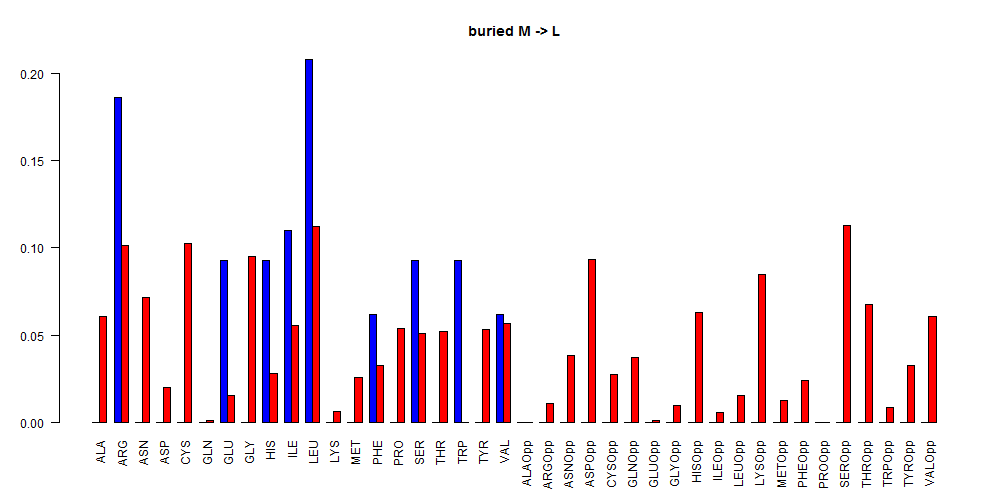

Supplement: Dataset S3 — Neighbouring residue profiles for mutations classed by substitution. (ZIP) [file pone.0084598.s003.zip › neighbour_2/buried_M_L.tif]

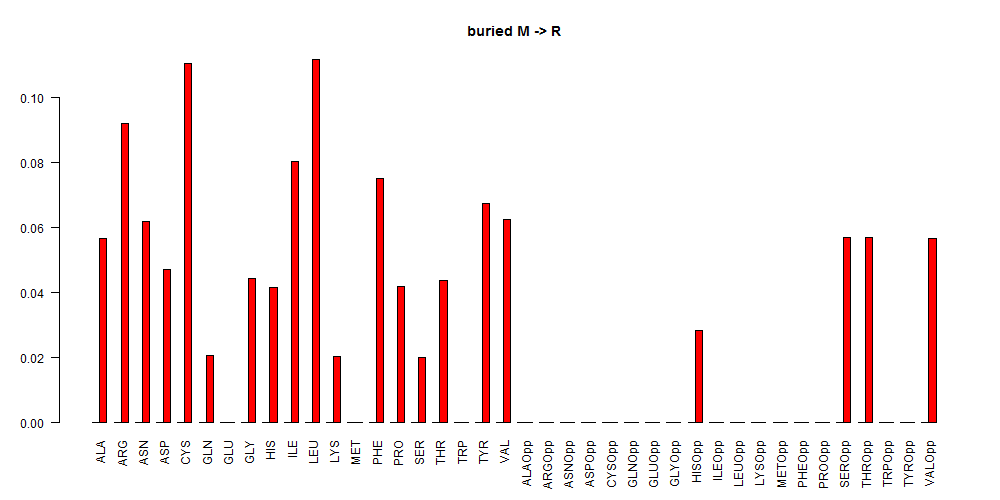

Supplement: Dataset S3 — Neighbouring residue profiles for mutations classed by substitution. (ZIP) [file pone.0084598.s003.zip › neighbour_2/buried_M_R.tif]

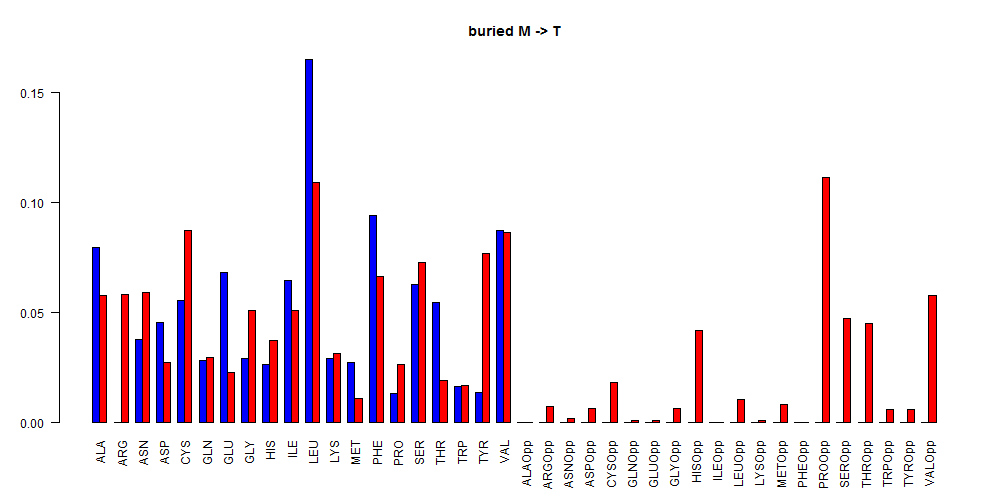

Supplement: Dataset S3 — Neighbouring residue profiles for mutations classed by substitution. (ZIP) [file pone.0084598.s003.zip › neighbour_2/buried_M_T.tif]

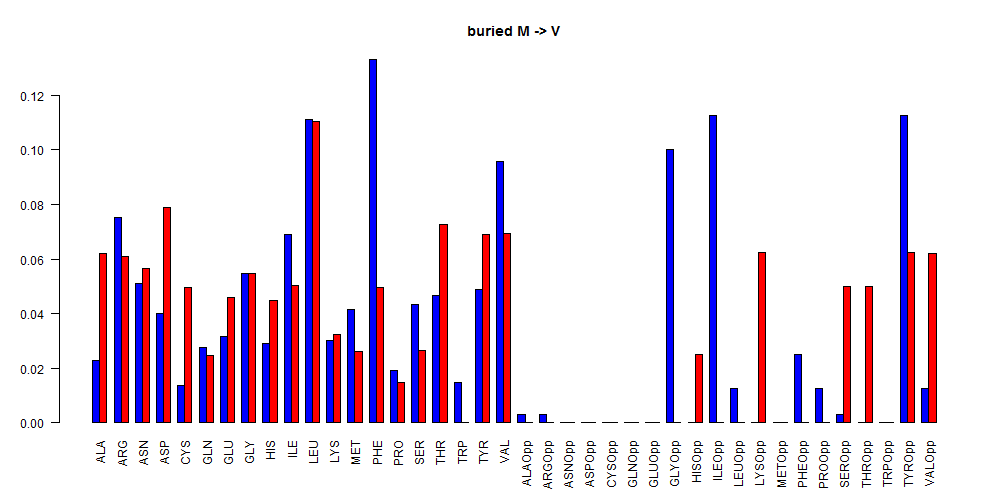

Supplement: Dataset S3 — Neighbouring residue profiles for mutations classed by substitution. (ZIP) [file pone.0084598.s003.zip › neighbour_2/buried_M_V.tif]

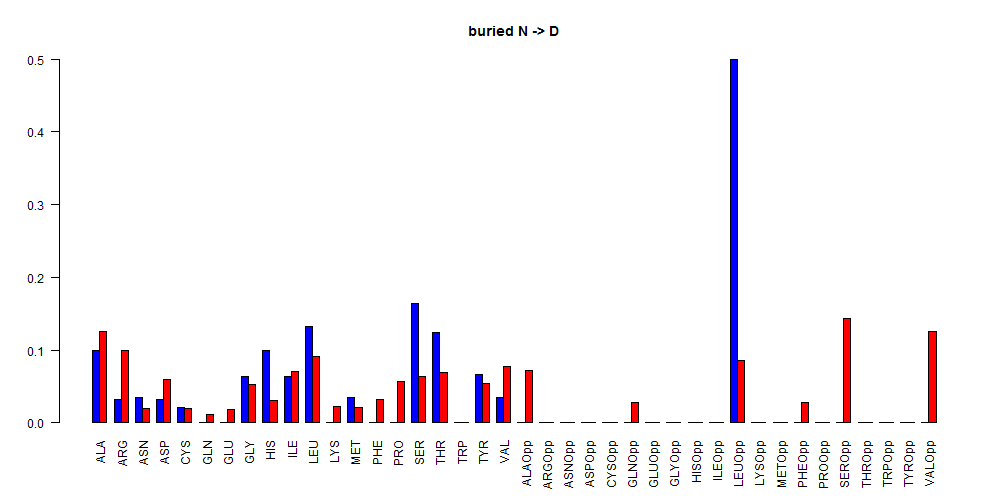

Supplement: Dataset S3 — Neighbouring residue profiles for mutations classed by substitution. (ZIP) [file pone.0084598.s003.zip › neighbour_2/buried_N_D.tif]

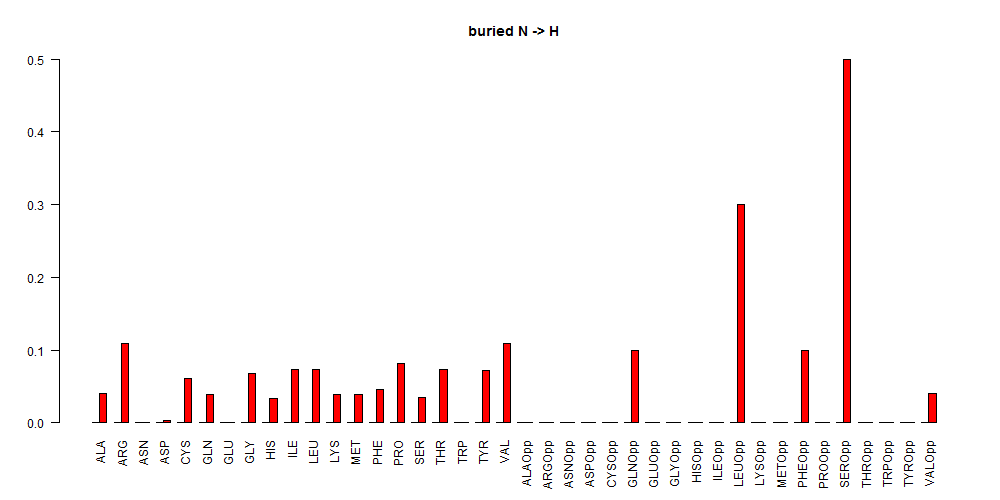

Supplement: Dataset S3 — Neighbouring residue profiles for mutations classed by substitution. (ZIP) [file pone.0084598.s003.zip › neighbour_2/buried_N_H.tif]

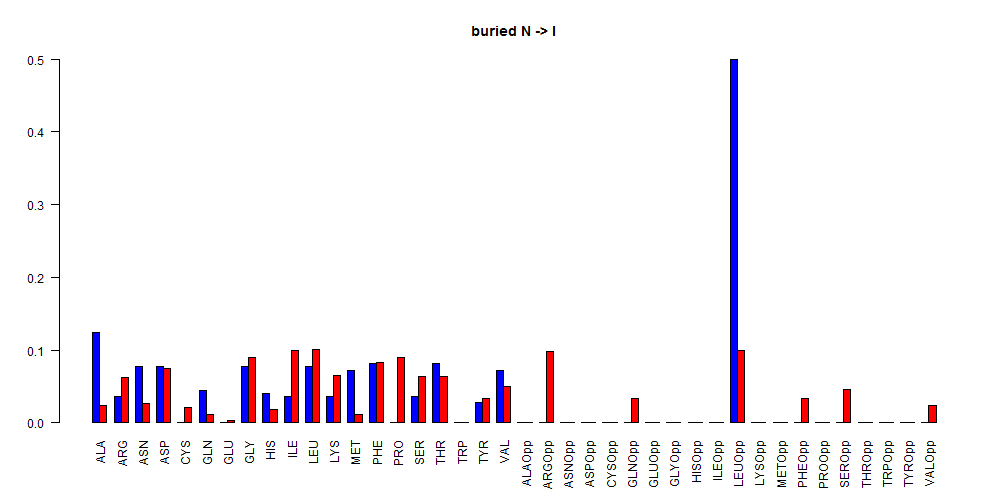

Supplement: Dataset S3 — Neighbouring residue profiles for mutations classed by substitution. (ZIP) [file pone.0084598.s003.zip › neighbour_2/buried_N_I.tif]

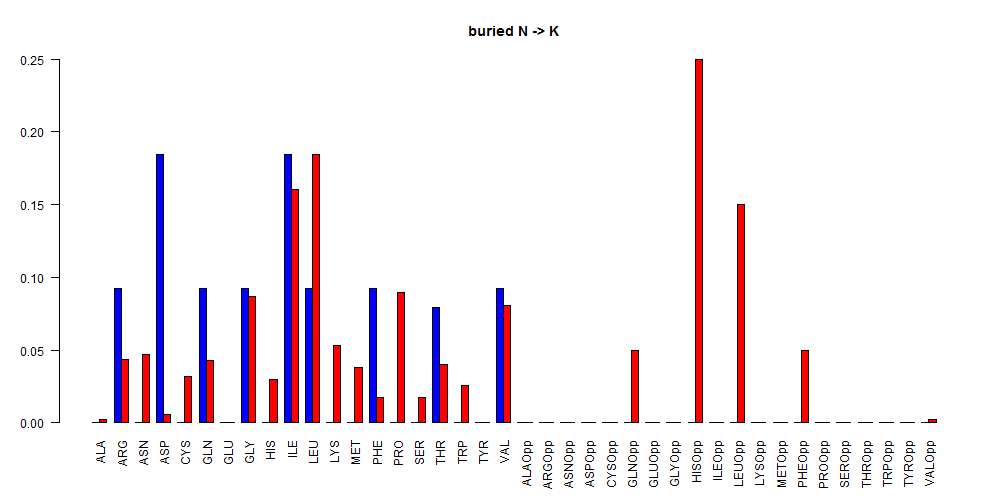

Supplement: Dataset S3 — Neighbouring residue profiles for mutations classed by substitution. (ZIP) [file pone.0084598.s003.zip › neighbour_2/buried_N_K.tif]

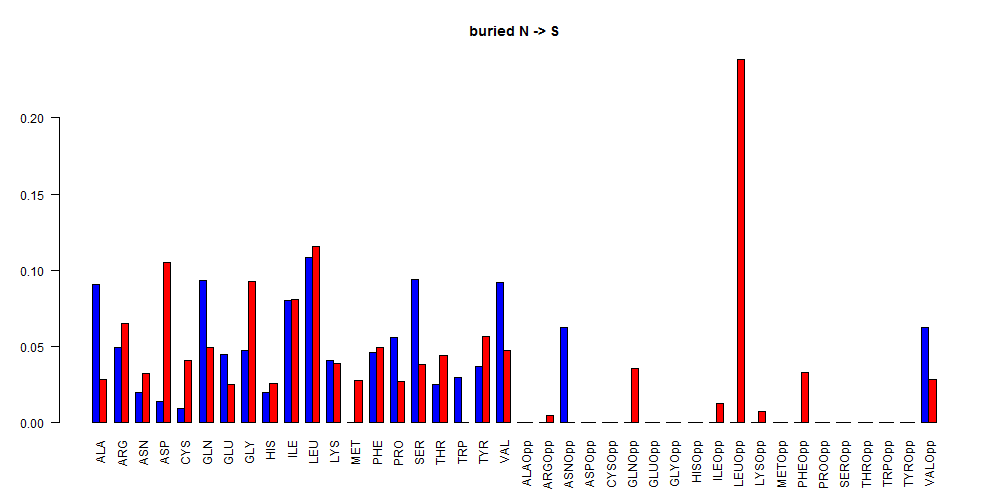

Supplement: Dataset S3 — Neighbouring residue profiles for mutations classed by substitution. (ZIP) [file pone.0084598.s003.zip › neighbour_2/buried_N_S.tif]

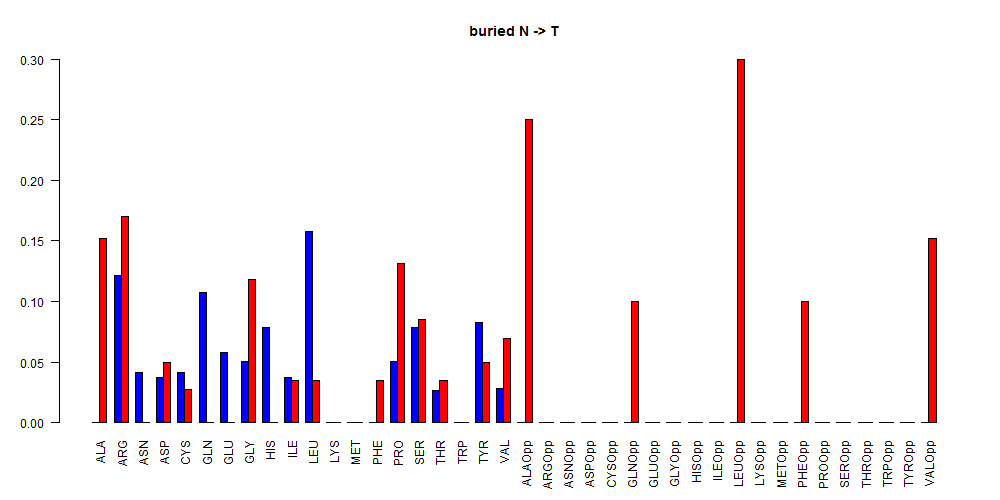

Supplement: Dataset S3 — Neighbouring residue profiles for mutations classed by substitution. (ZIP) [file pone.0084598.s003.zip › neighbour_2/buried_N_T.tif]

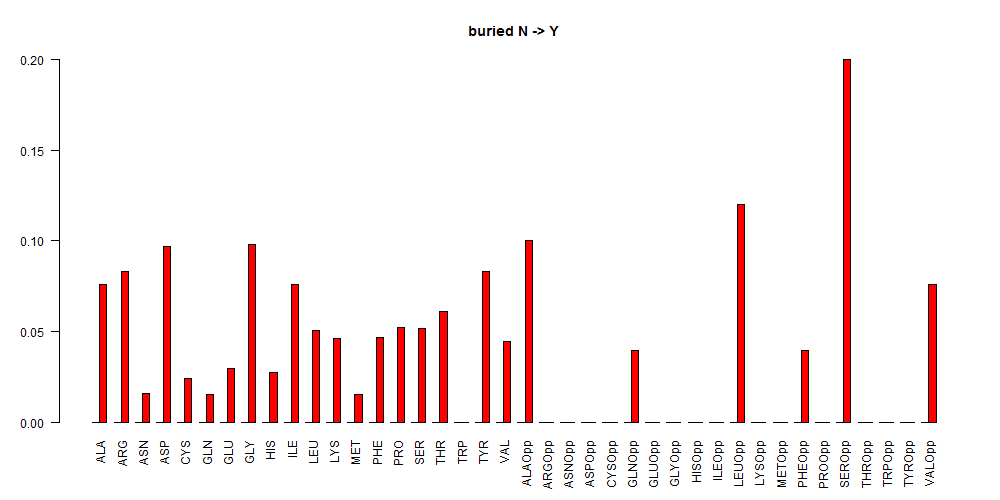

Supplement: Dataset S3 — Neighbouring residue profiles for mutations classed by substitution. (ZIP) [file pone.0084598.s003.zip › neighbour_2/buried_N_Y.tif]

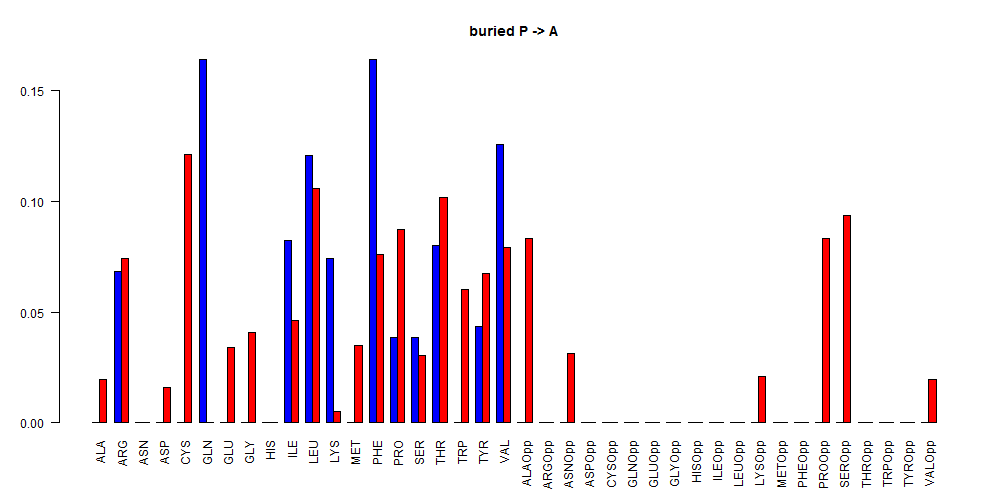

Supplement: Dataset S3 — Neighbouring residue profiles for mutations classed by substitution. (ZIP) [file pone.0084598.s003.zip › neighbour_2/buried_P_A.tif]

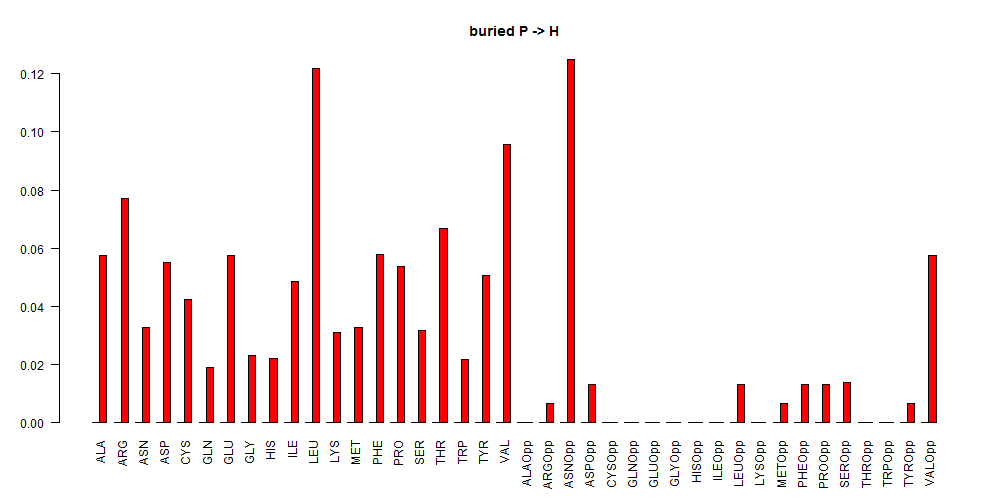

Supplement: Dataset S3 — Neighbouring residue profiles for mutations classed by substitution. (ZIP) [file pone.0084598.s003.zip › neighbour_2/buried_P_H.tif]

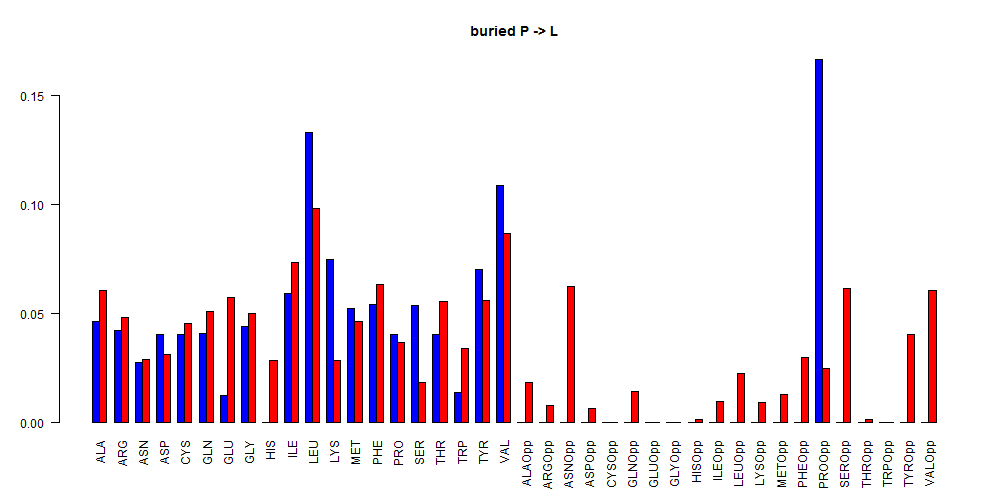

Supplement: Dataset S3 — Neighbouring residue profiles for mutations classed by substitution. (ZIP) [file pone.0084598.s003.zip › neighbour_2/buried_P_L.tif]

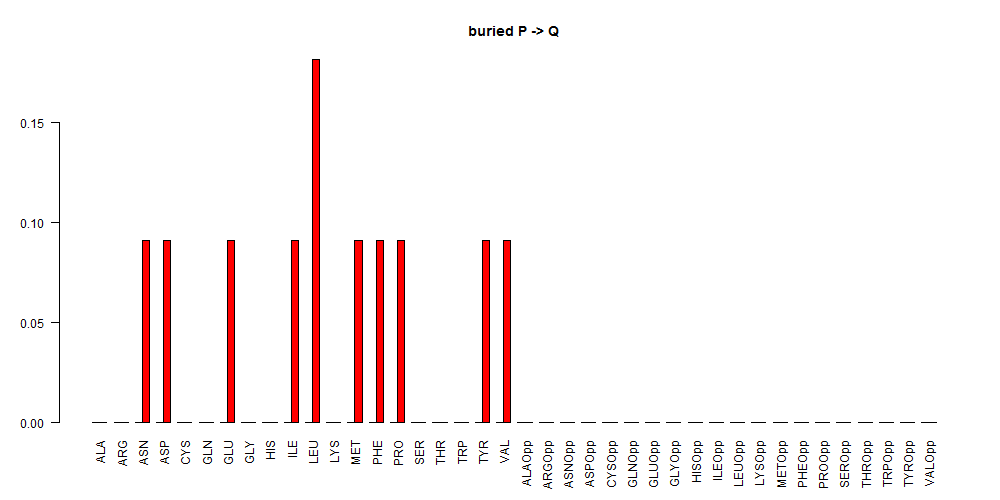

Supplement: Dataset S3 — Neighbouring residue profiles for mutations classed by substitution. (ZIP) [file pone.0084598.s003.zip › neighbour_2/buried_P_Q.tif]

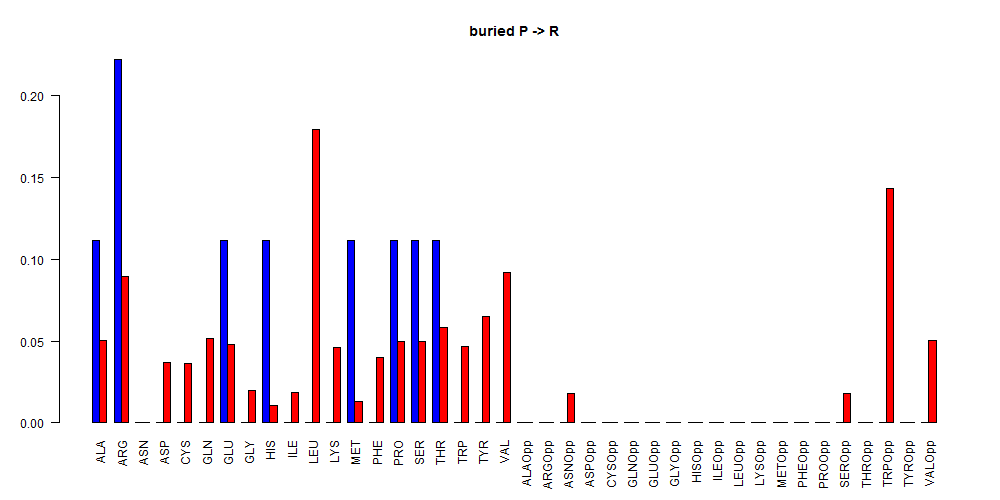

Supplement: Dataset S3 — Neighbouring residue profiles for mutations classed by substitution. (ZIP) [file pone.0084598.s003.zip › neighbour_2/buried_P_R.tif]

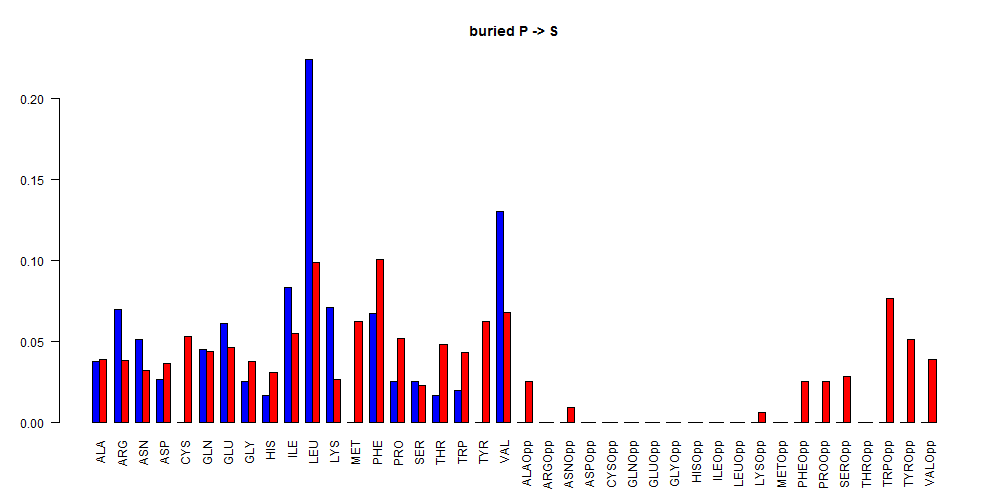

Supplement: Dataset S3 — Neighbouring residue profiles for mutations classed by substitution. (ZIP) [file pone.0084598.s003.zip › neighbour_2/buried_P_S.tif]

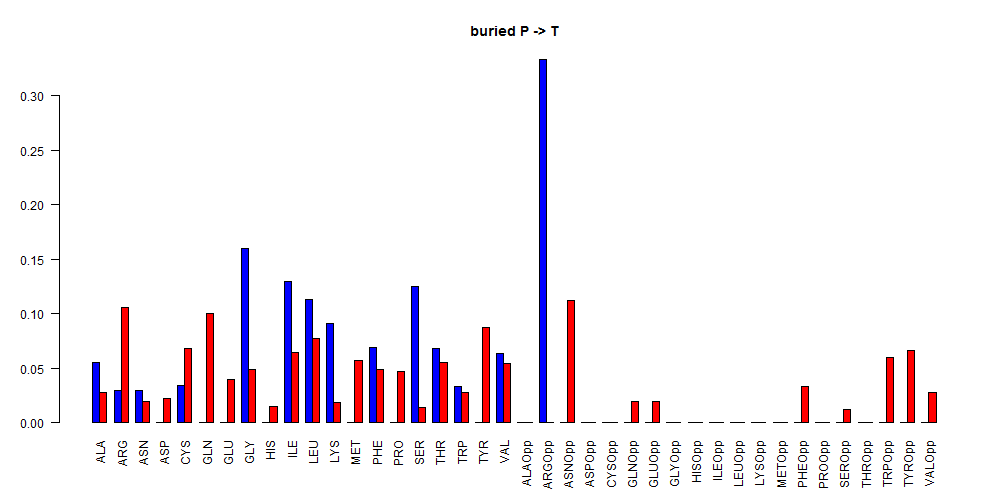

Supplement: Dataset S3 — Neighbouring residue profiles for mutations classed by substitution. (ZIP) [file pone.0084598.s003.zip › neighbour_2/buried_P_T.tif]

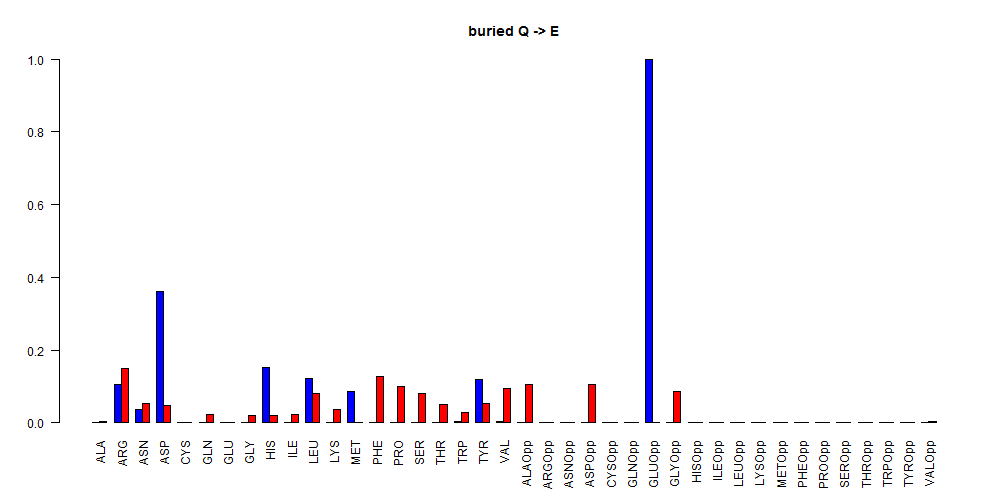

Supplement: Dataset S3 — Neighbouring residue profiles for mutations classed by substitution. (ZIP) [file pone.0084598.s003.zip › neighbour_2/buried_Q_E.tif]

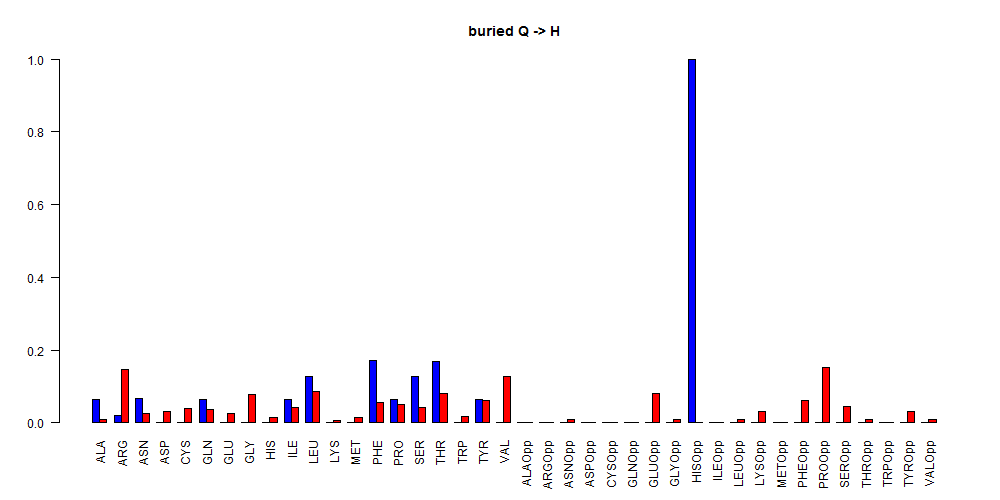

Supplement: Dataset S3 — Neighbouring residue profiles for mutations classed by substitution. (ZIP) [file pone.0084598.s003.zip › neighbour_2/buried_Q_H.tif]

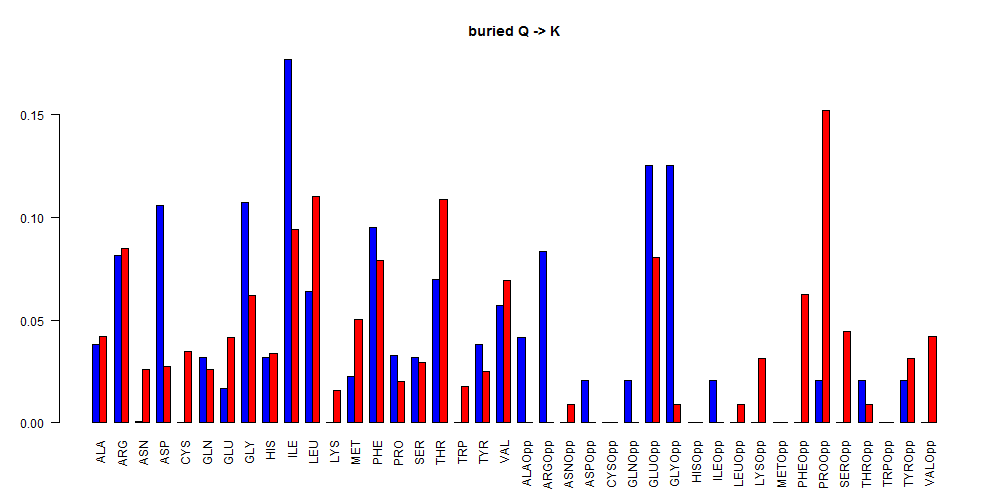

Supplement: Dataset S3 — Neighbouring residue profiles for mutations classed by substitution. (ZIP) [file pone.0084598.s003.zip › neighbour_2/buried_Q_K.tif]

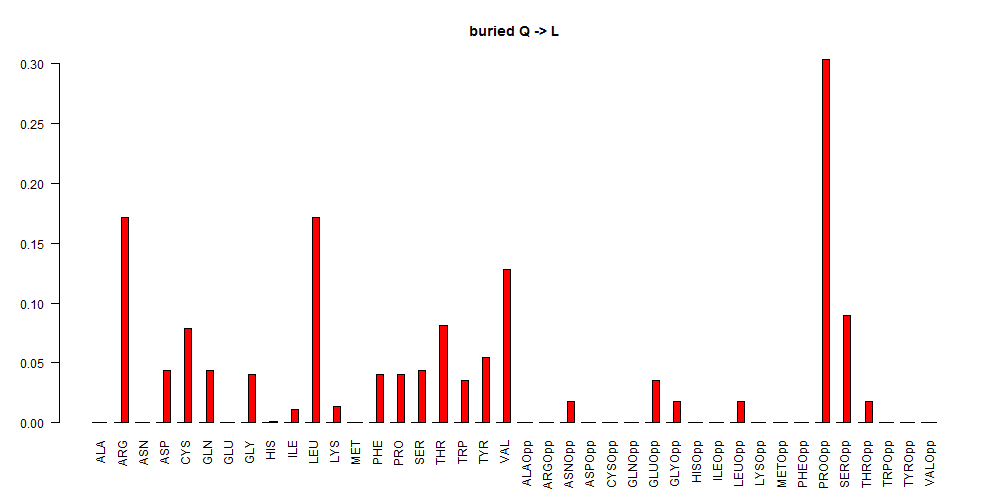

Supplement: Dataset S3 — Neighbouring residue profiles for mutations classed by substitution. (ZIP) [file pone.0084598.s003.zip › neighbour_2/buried_Q_L.tif]

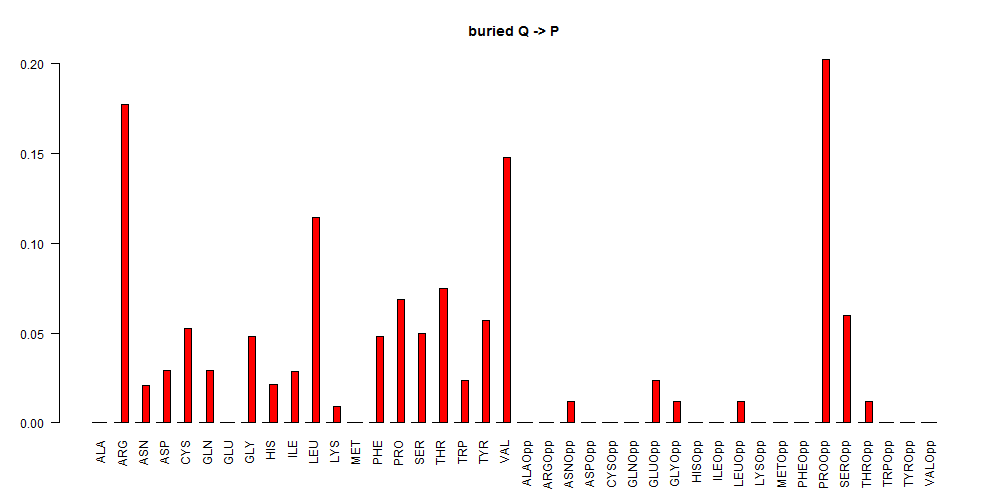

Supplement: Dataset S3 — Neighbouring residue profiles for mutations classed by substitution. (ZIP) [file pone.0084598.s003.zip › neighbour_2/buried_Q_P.tif]

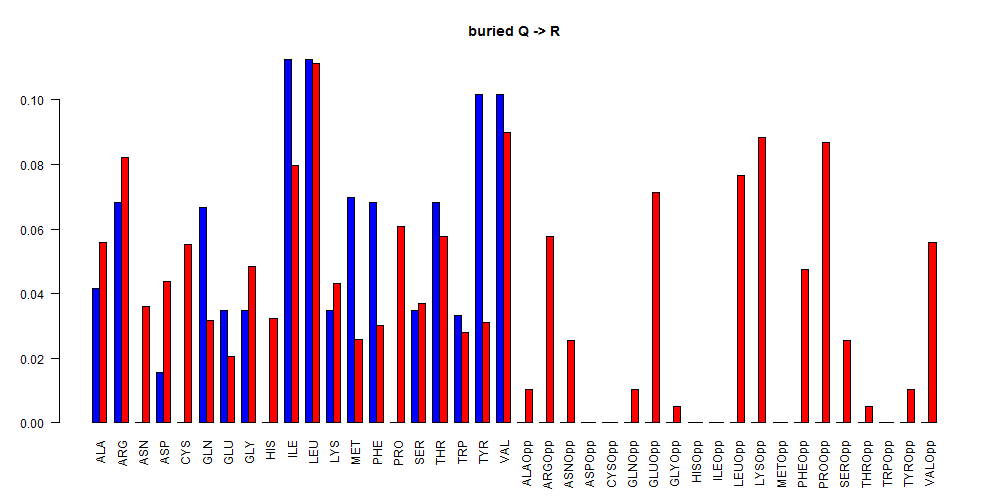

Supplement: Dataset S3 — Neighbouring residue profiles for mutations classed by substitution. (ZIP) [file pone.0084598.s003.zip › neighbour_2/buried_Q_R.tif]

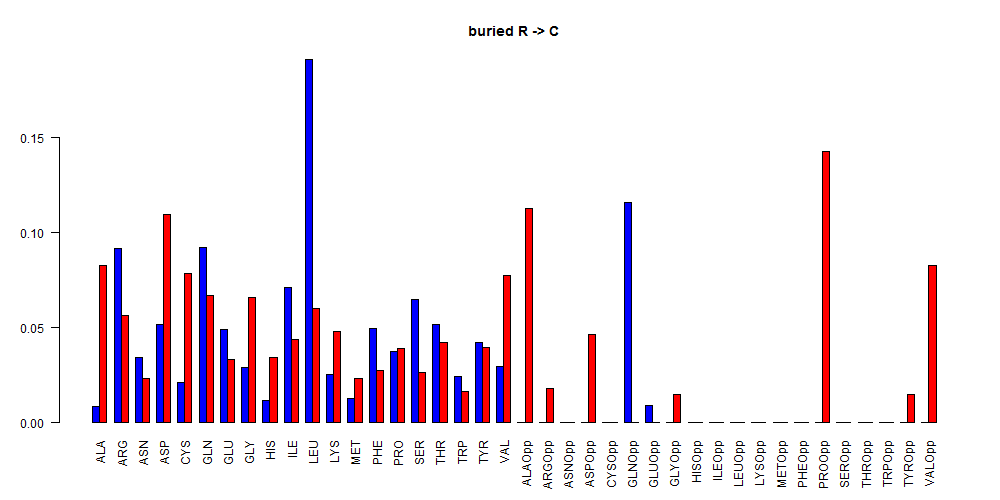

Supplement: Dataset S3 — Neighbouring residue profiles for mutations classed by substitution. (ZIP) [file pone.0084598.s003.zip › neighbour_2/buried_R_C.tif]

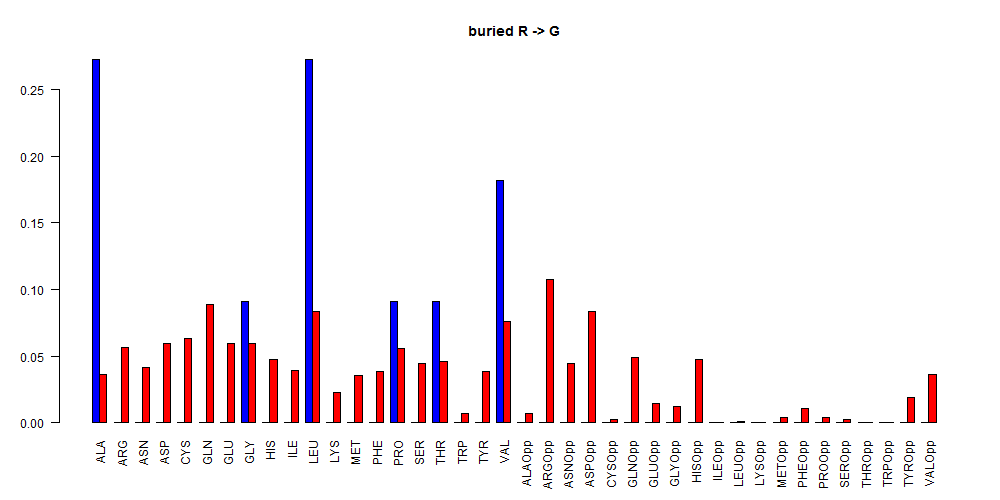

Supplement: Dataset S3 — Neighbouring residue profiles for mutations classed by substitution. (ZIP) [file pone.0084598.s003.zip › neighbour_2/buried_R_G.tif]

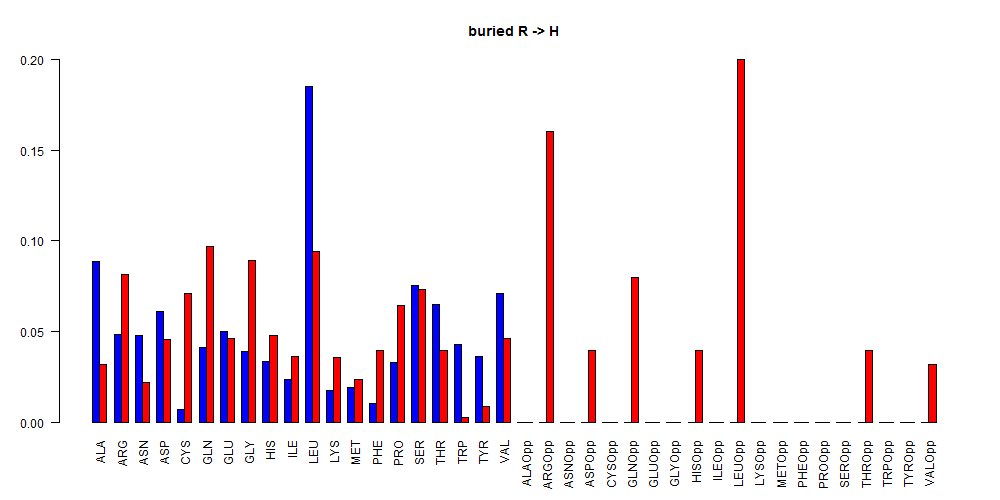

Supplement: Dataset S3 — Neighbouring residue profiles for mutations classed by substitution. (ZIP) [file pone.0084598.s003.zip › neighbour_2/buried_R_H.tif]

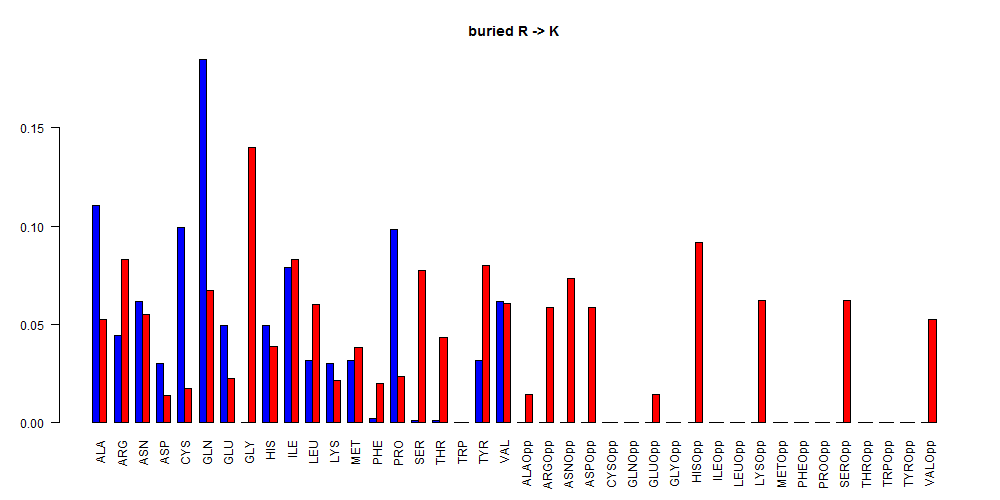

Supplement: Dataset S3 — Neighbouring residue profiles for mutations classed by substitution. (ZIP) [file pone.0084598.s003.zip › neighbour_2/buried_R_K.tif]

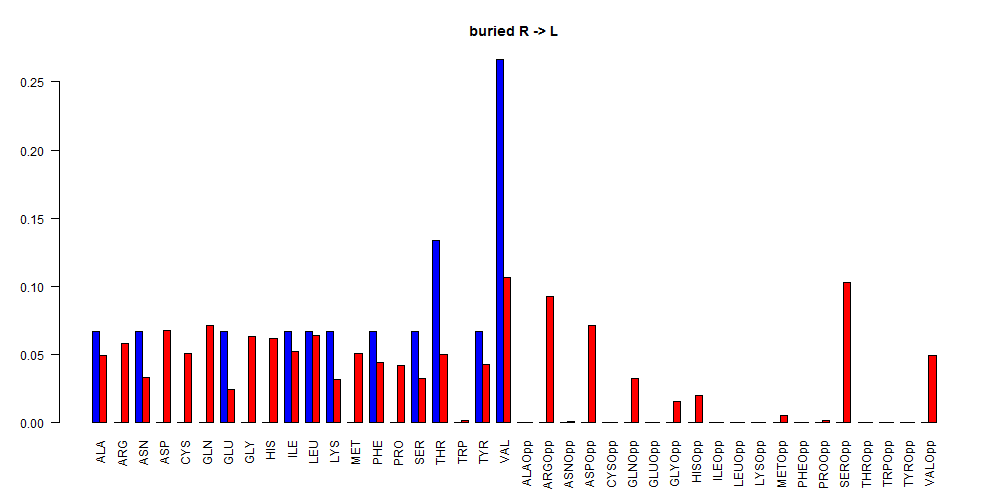

Supplement: Dataset S3 — Neighbouring residue profiles for mutations classed by substitution. (ZIP) [file pone.0084598.s003.zip › neighbour_2/buried_R_L.tif]

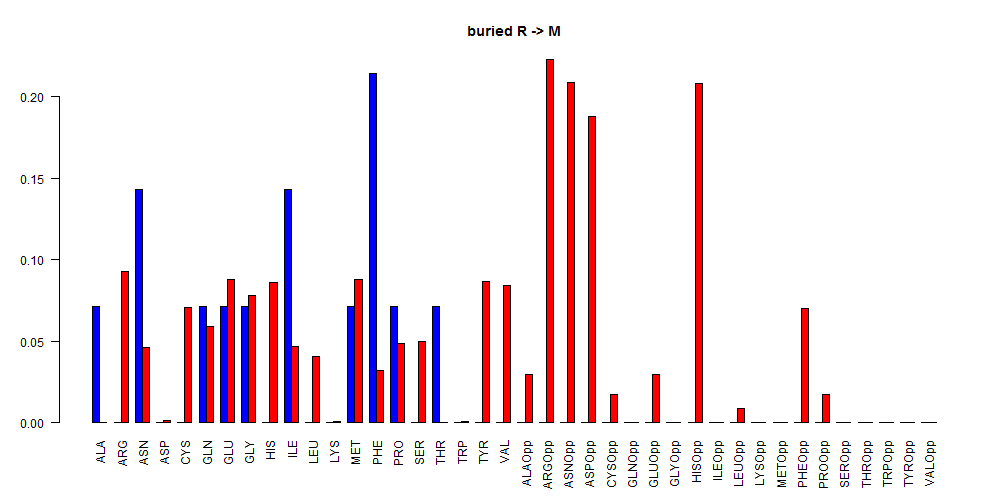

Supplement: Dataset S3 — Neighbouring residue profiles for mutations classed by substitution. (ZIP) [file pone.0084598.s003.zip › neighbour_2/buried_R_M.tif]

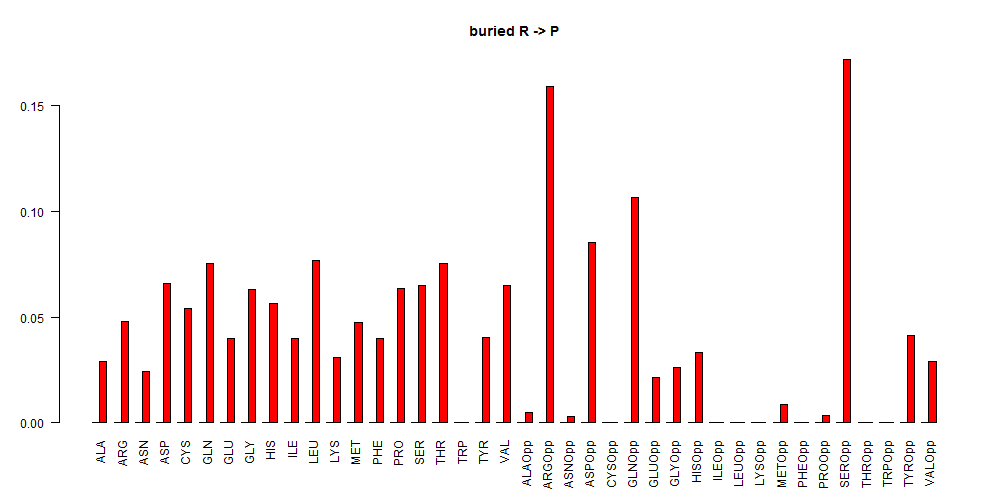

Supplement: Dataset S3 — Neighbouring residue profiles for mutations classed by substitution. (ZIP) [file pone.0084598.s003.zip › neighbour_2/buried_R_P.tif]

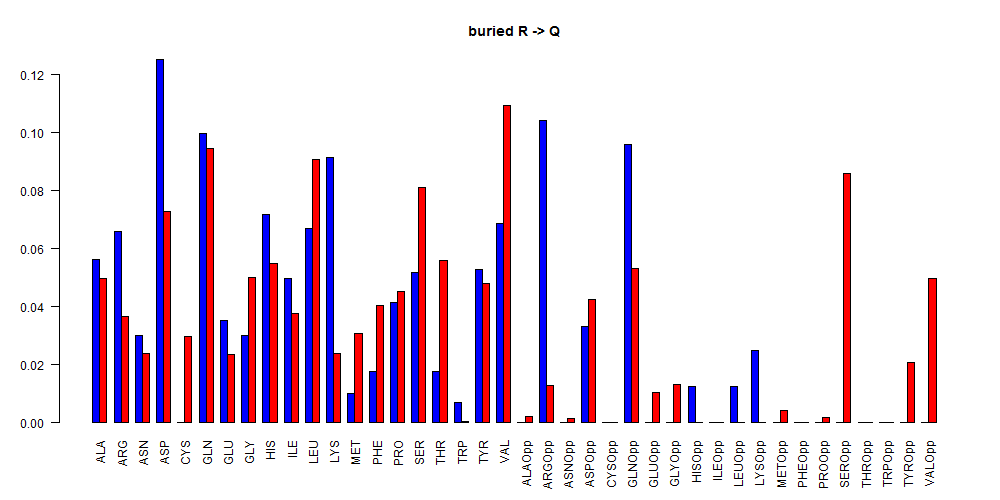

Supplement: Dataset S3 — Neighbouring residue profiles for mutations classed by substitution. (ZIP) [file pone.0084598.s003.zip › neighbour_2/buried_R_Q.tif]

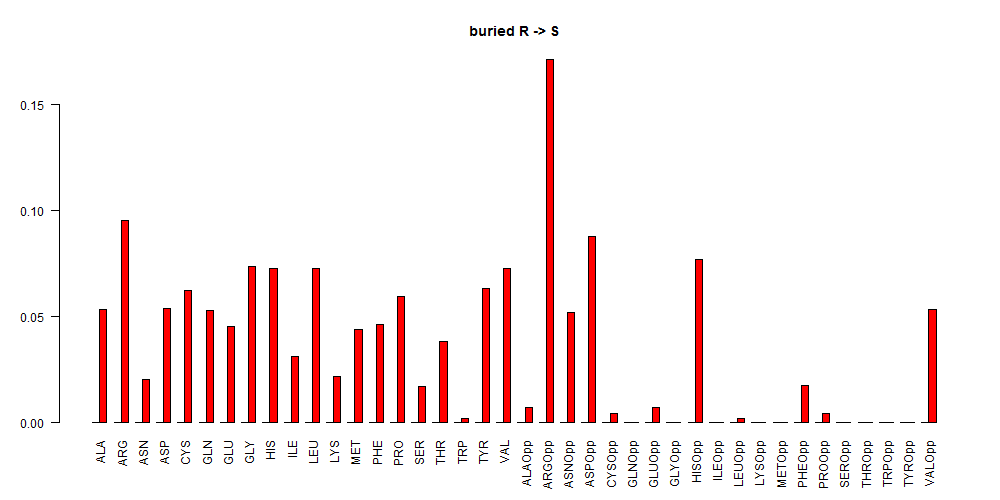

Supplement: Dataset S3 — Neighbouring residue profiles for mutations classed by substitution. (ZIP) [file pone.0084598.s003.zip › neighbour_2/buried_R_S.tif]

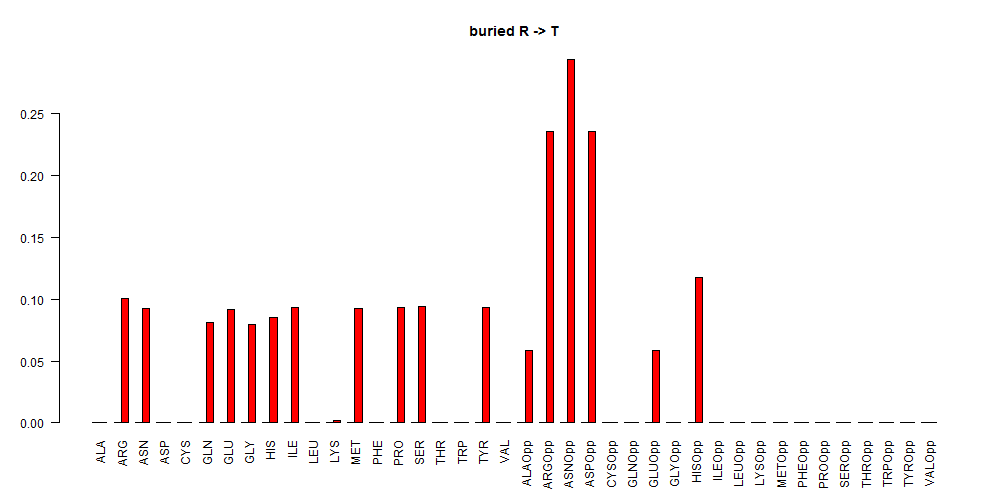

Supplement: Dataset S3 — Neighbouring residue profiles for mutations classed by substitution. (ZIP) [file pone.0084598.s003.zip › neighbour_2/buried_R_T.tif]

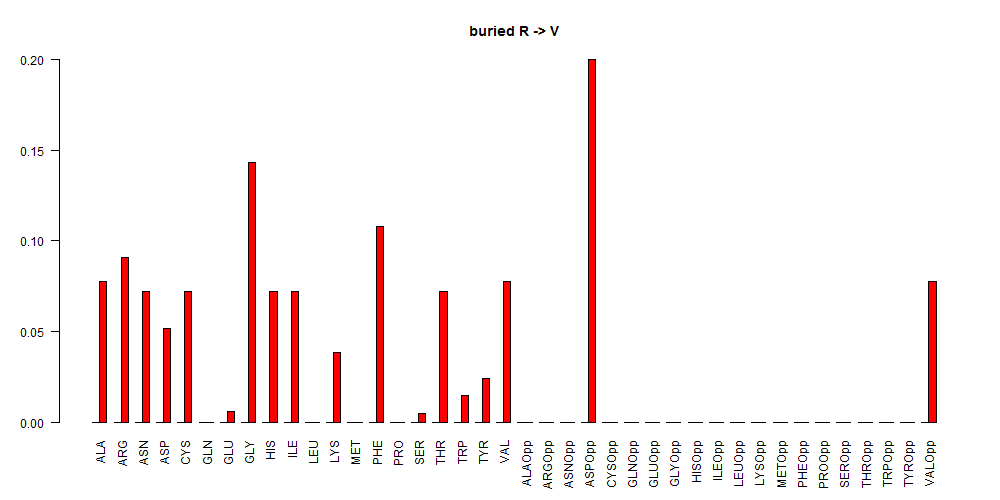

Supplement: Dataset S3 — Neighbouring residue profiles for mutations classed by substitution. (ZIP) [file pone.0084598.s003.zip › neighbour_2/buried_R_V.tif]

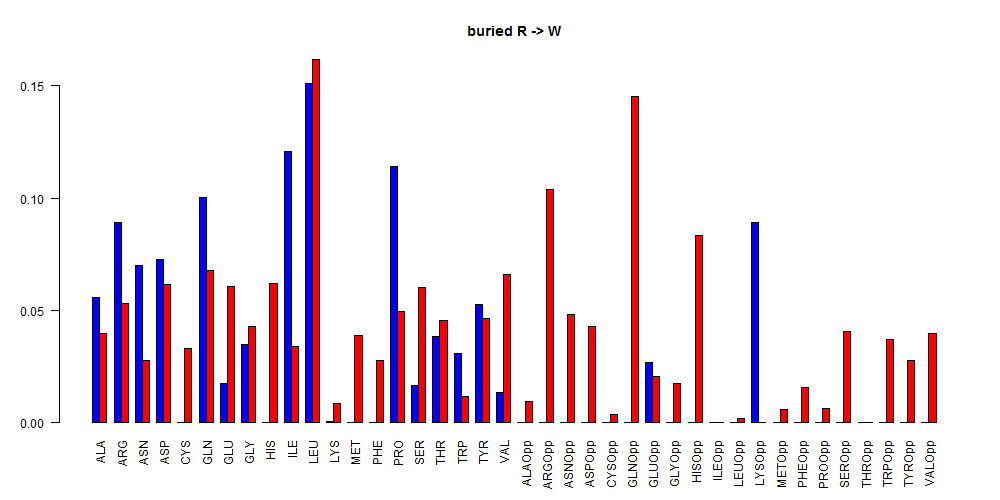

Supplement: Dataset S3 — Neighbouring residue profiles for mutations classed by substitution. (ZIP) [file pone.0084598.s003.zip › neighbour_2/buried_R_W.tif]

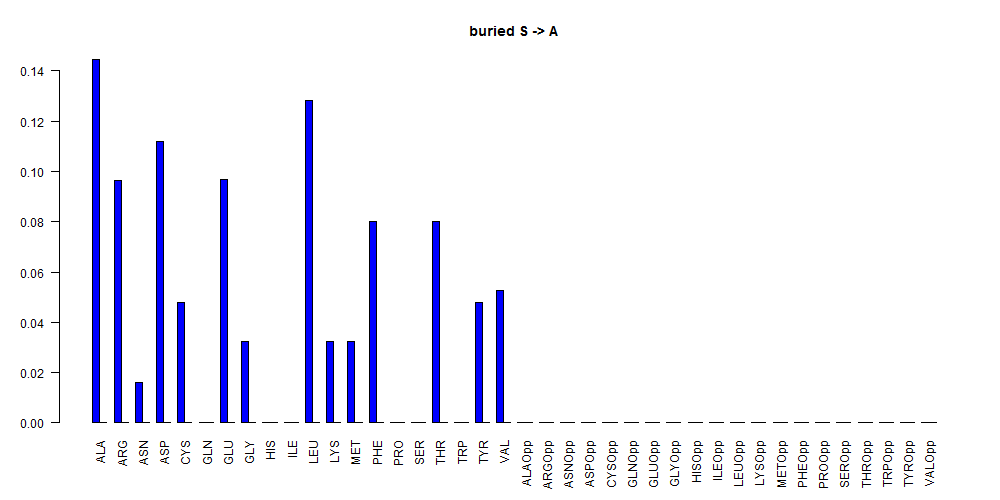

Supplement: Dataset S3 — Neighbouring residue profiles for mutations classed by substitution. (ZIP) [file pone.0084598.s003.zip › neighbour_2/buried_S_A.tif]

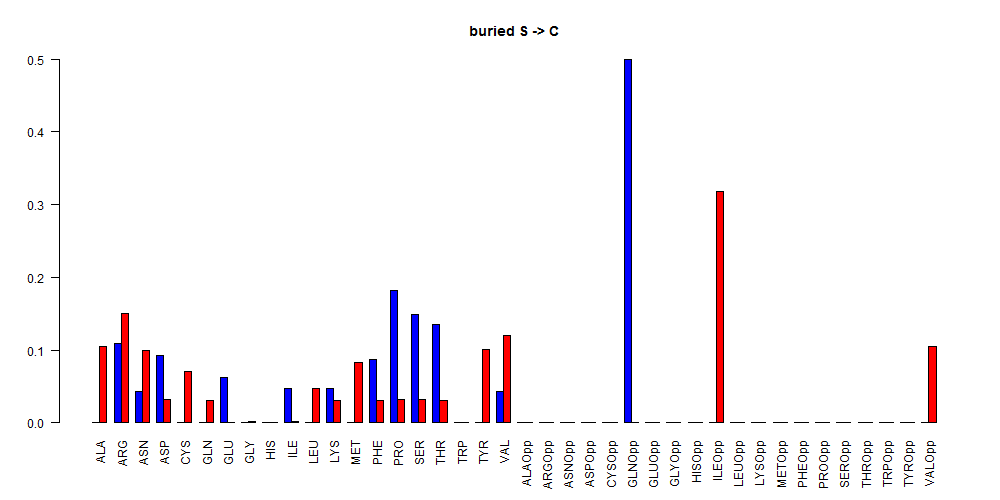

Supplement: Dataset S3 — Neighbouring residue profiles for mutations classed by substitution. (ZIP) [file pone.0084598.s003.zip › neighbour_2/buried_S_C.tif]

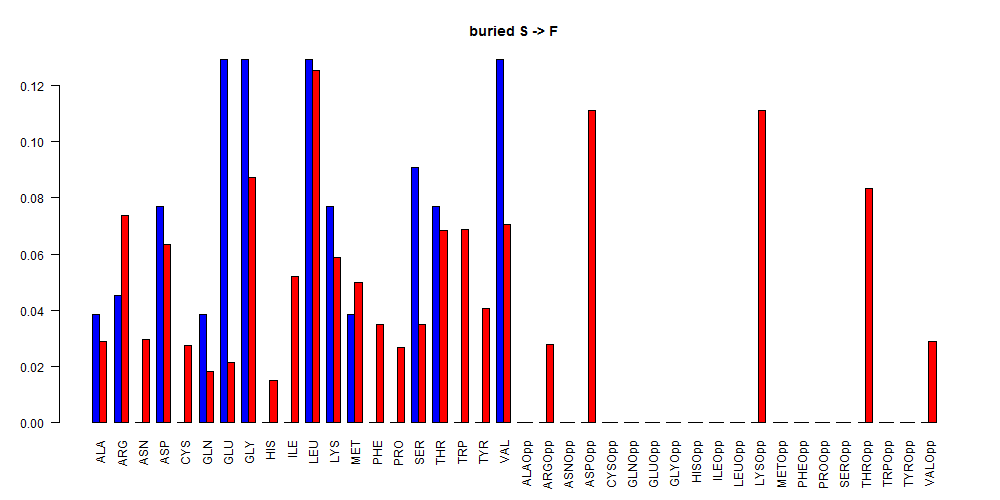

Supplement: Dataset S3 — Neighbouring residue profiles for mutations classed by substitution. (ZIP) [file pone.0084598.s003.zip › neighbour_2/buried_S_F.tif]

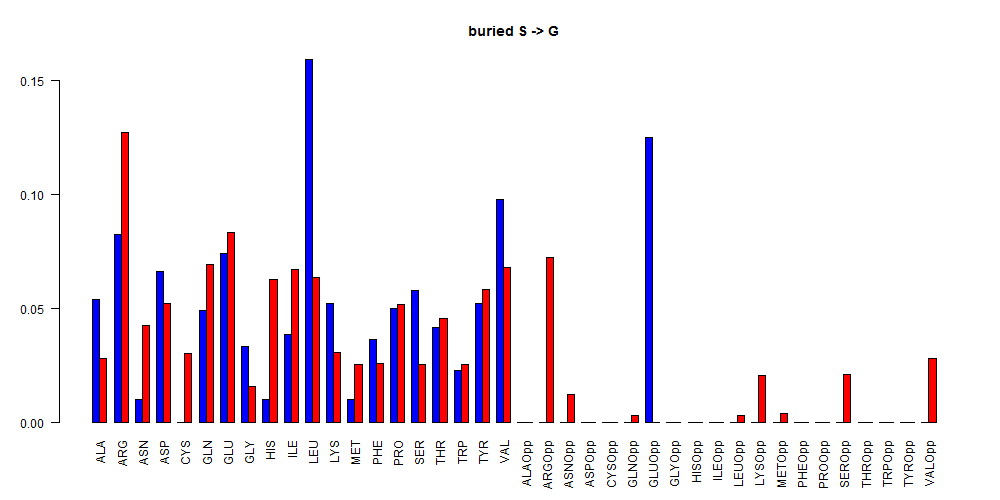

Supplement: Dataset S3 — Neighbouring residue profiles for mutations classed by substitution. (ZIP) [file pone.0084598.s003.zip › neighbour_2/buried_S_G.tif]

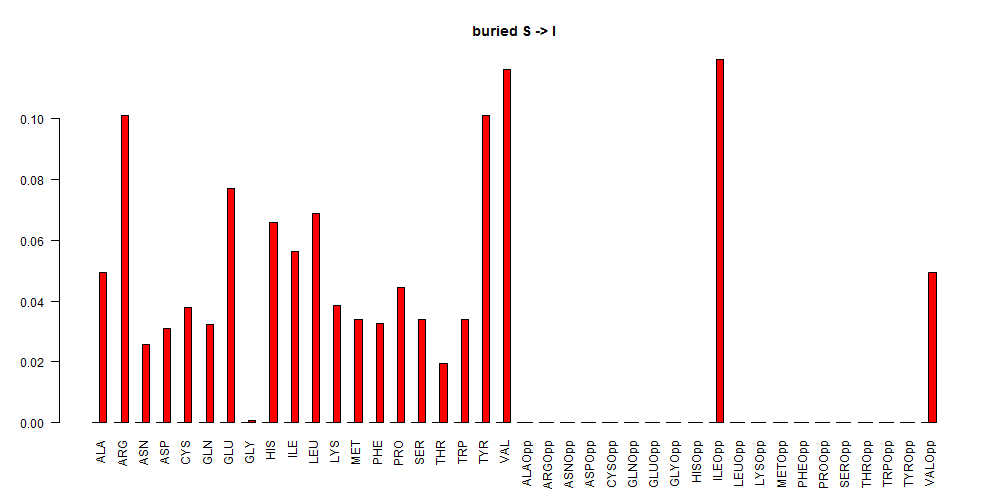

Supplement: Dataset S3 — Neighbouring residue profiles for mutations classed by substitution. (ZIP) [file pone.0084598.s003.zip › neighbour_2/buried_S_I.tif]

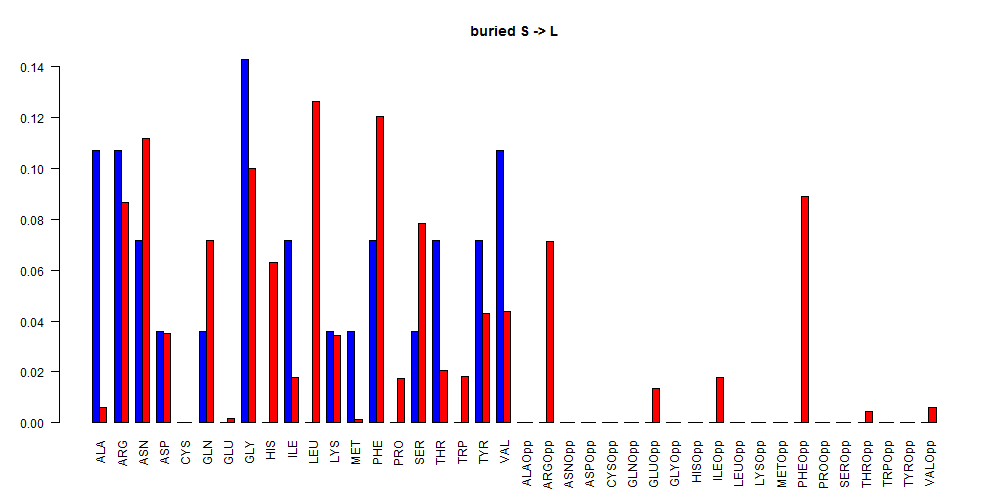

Supplement: Dataset S3 — Neighbouring residue profiles for mutations classed by substitution. (ZIP) [file pone.0084598.s003.zip › neighbour_2/buried_S_L.tif]

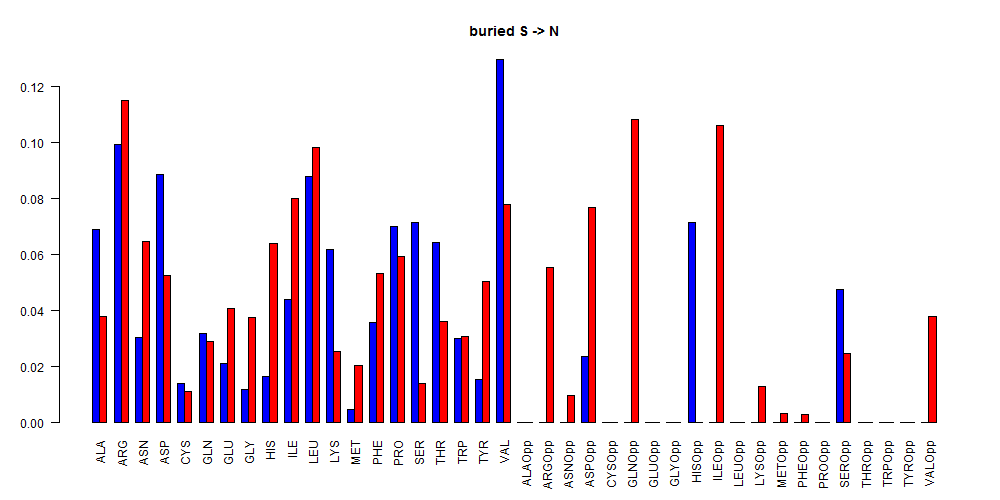

Supplement: Dataset S3 — Neighbouring residue profiles for mutations classed by substitution. (ZIP) [file pone.0084598.s003.zip › neighbour_2/buried_S_N.tif]

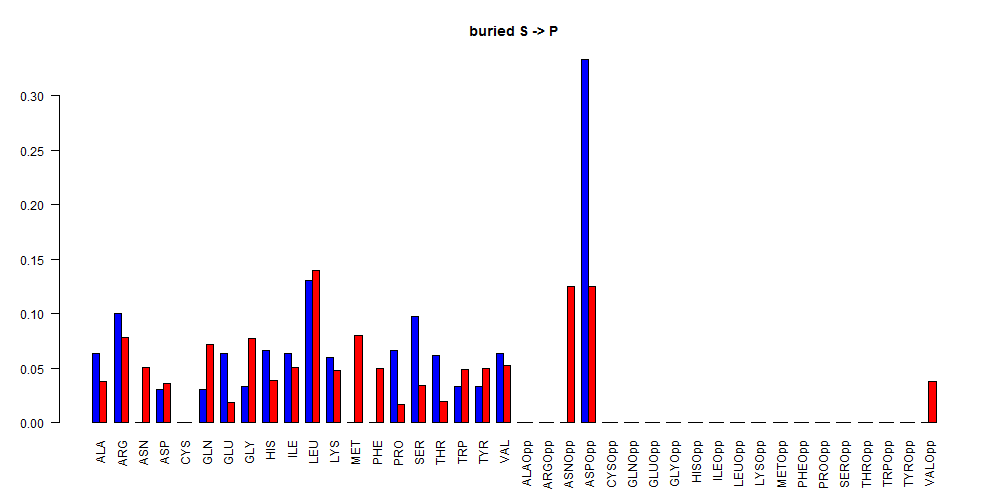

Supplement: Dataset S3 — Neighbouring residue profiles for mutations classed by substitution. (ZIP) [file pone.0084598.s003.zip › neighbour_2/buried_S_P.tif]

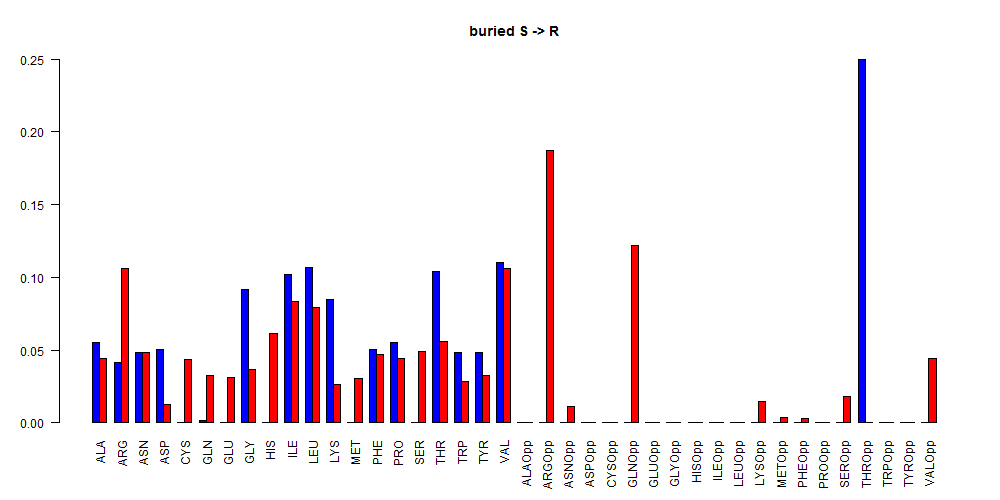

Supplement: Dataset S3 — Neighbouring residue profiles for mutations classed by substitution. (ZIP) [file pone.0084598.s003.zip › neighbour_2/buried_S_R.tif]

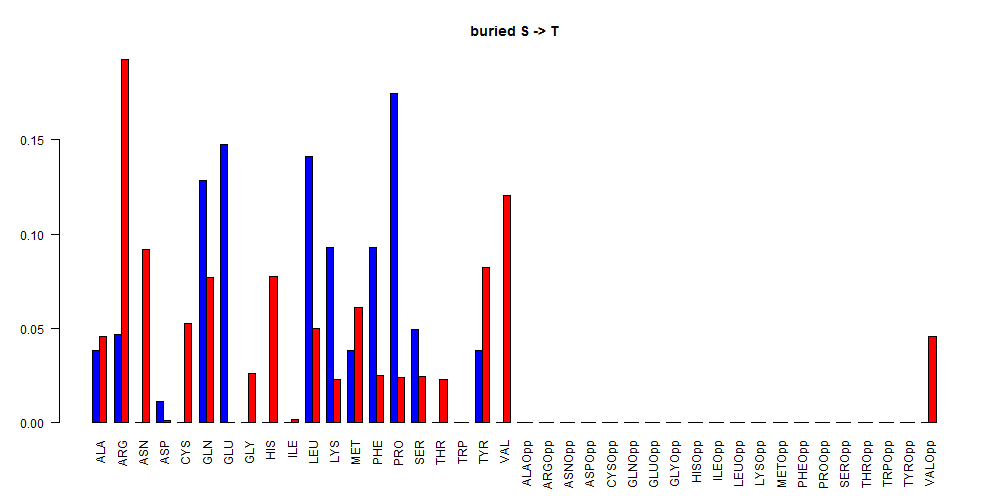

Supplement: Dataset S3 — Neighbouring residue profiles for mutations classed by substitution. (ZIP) [file pone.0084598.s003.zip › neighbour_2/buried_S_T.tif]

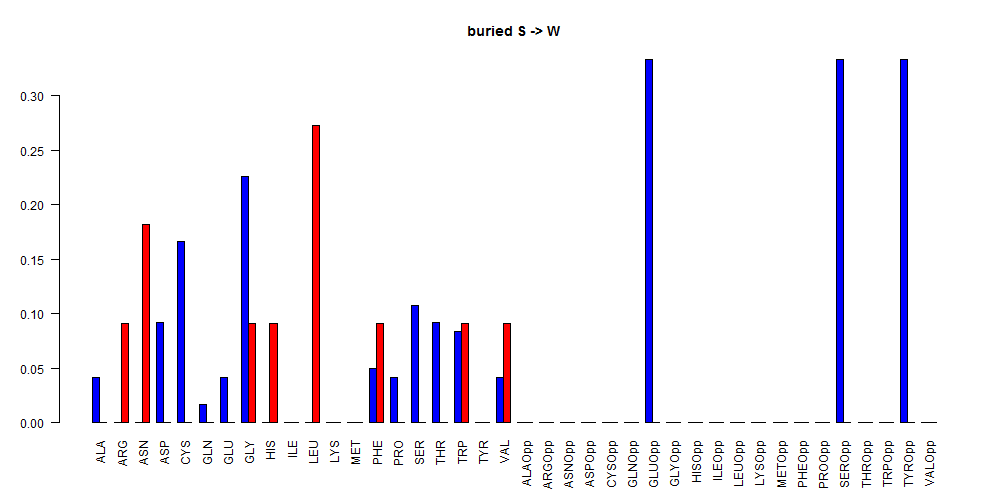

Supplement: Dataset S3 — Neighbouring residue profiles for mutations classed by substitution. (ZIP) [file pone.0084598.s003.zip › neighbour_2/buried_S_W.tif]

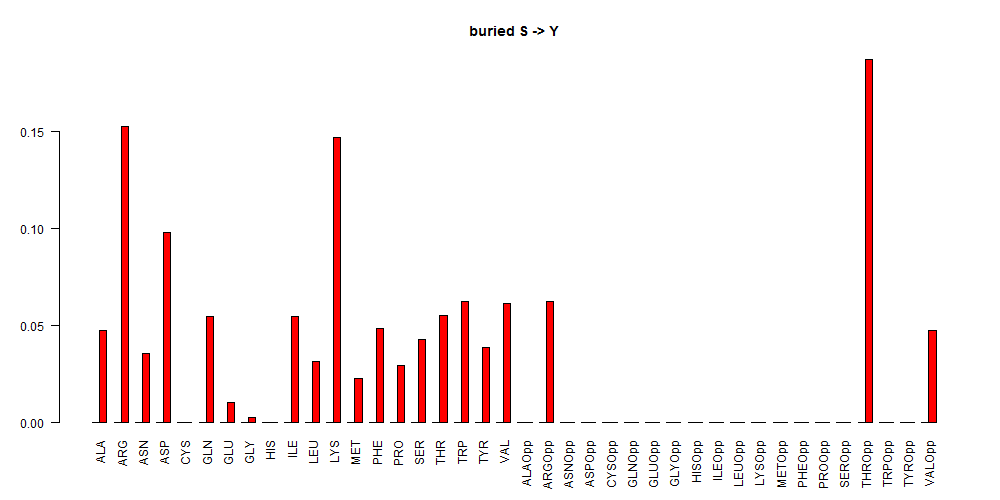

Supplement: Dataset S3 — Neighbouring residue profiles for mutations classed by substitution. (ZIP) [file pone.0084598.s003.zip › neighbour_2/buried_S_Y.tif]

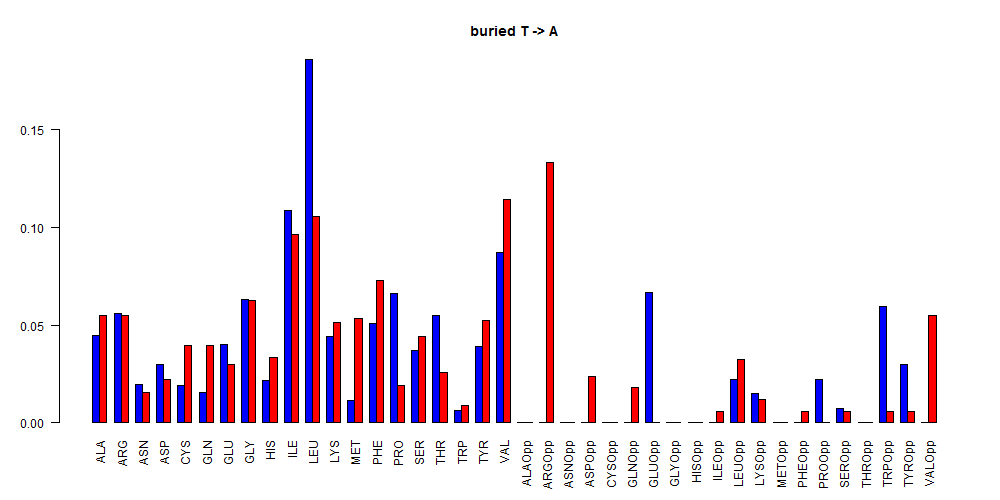

Supplement: Dataset S3 — Neighbouring residue profiles for mutations classed by substitution. (ZIP) [file pone.0084598.s003.zip › neighbour_2/buried_T_A.tif]

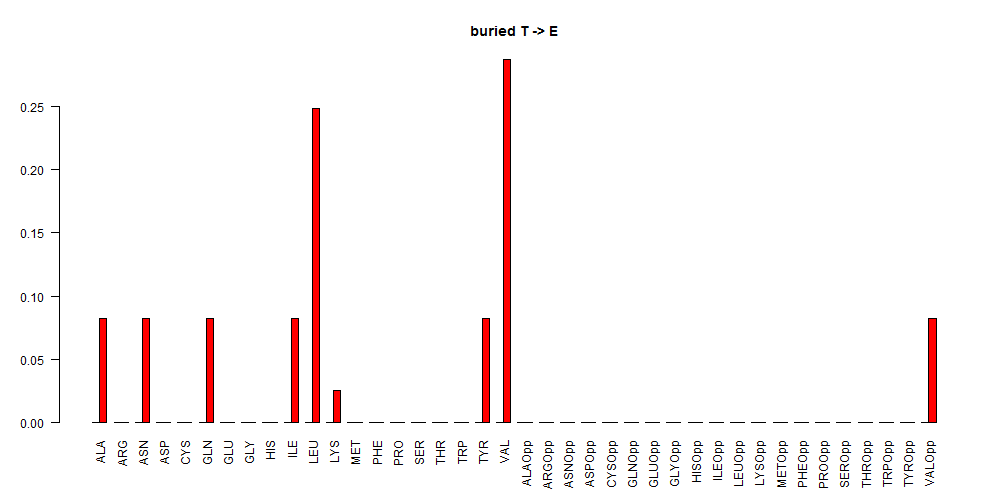

Supplement: Dataset S3 — Neighbouring residue profiles for mutations classed by substitution. (ZIP) [file pone.0084598.s003.zip › neighbour_2/buried_T_E.tif]

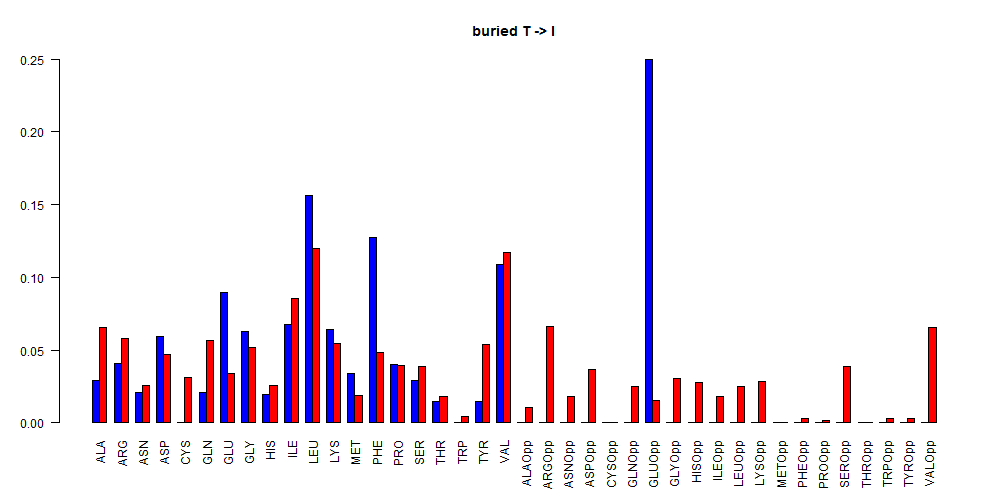

Supplement: Dataset S3 — Neighbouring residue profiles for mutations classed by substitution. (ZIP) [file pone.0084598.s003.zip › neighbour_2/buried_T_I.tif]

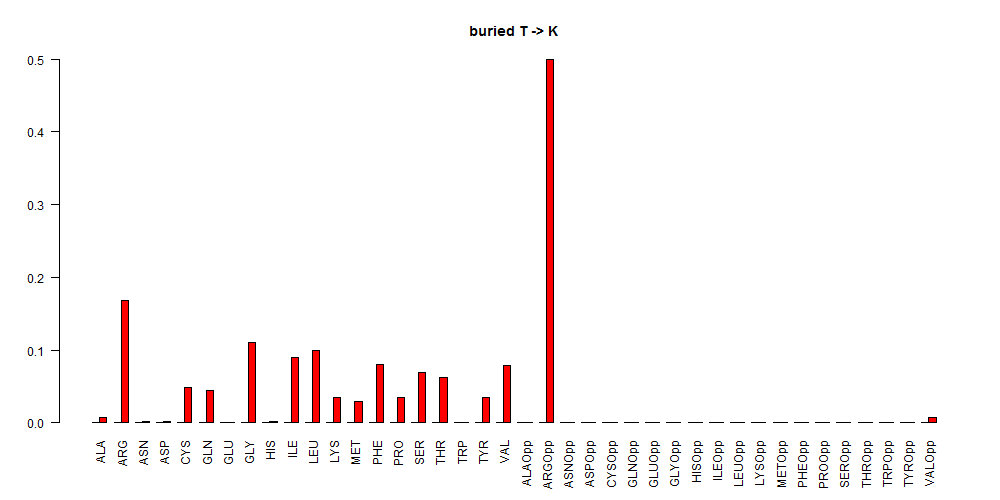

Supplement: Dataset S3 — Neighbouring residue profiles for mutations classed by substitution. (ZIP) [file pone.0084598.s003.zip › neighbour_2/buried_T_K.tif]
