# Supplementary material for: Deriving a Mutation Index of Carcinogenicity Using Protein Structure and Protein Interfaces
Source: PLoS One. 2014 Jan 15;9(1):e84598. doi: 10.1371/journal.pone.0084598 (PMC3893166; doi:10.1371/journal.pone.0084598)
Supplement: Text S1 — Supporting methods. (DOC) [file pone.0084598.s029.doc]

**Deriving a Mutation Index of Carcinogenicity Using Protein Structure and Protein Interfaces**

### Octavio Espinosa, Costas Mitsopoulos, Jarle Hakas, Frances M G Pearl and Marketa Zvelebil

# **Supporting Text**

# Supporting methods

**Mutation severity analysis data and propensity analysis**

We constructed a matrix of amino acid physiochemical properties adapted from [1] (Table S2) and derived a correlation matrix used to calculate distance and hierarchical clustering with the average linkage method. Distance (**D**_c_) was calculated as

**D**_c_ = 1 – cor(**A**^T^)

where **A** denotes the binary amino acid × physicochemical property matrix. Mutation substitutions were scored according to this matrix and also according to their mutability according to the BLOSUM 62 EBI matrix, a variant of the matrix described in [2] and the Dayhoff mutability matrix [3].

**Comparison of predictors**

We calculated areas under curve (AUCs) of receiver operating characteristic (ROC) curves using the pROC package [4].

## Analysis of biases in area, secondary structure and amino acid composition

We computed the driver/neutral fractions of normalised frequencies as follows:

$$f_{driver/neutral}=\frac{\frac{n_{i}^{driver}}{t^{driver}}}{\frac{n_{i}^{neutral}}{t^{neutral}}}$$

Where *n_i_*^driver^ denotes the number of driver mutations in the category and *t*^driver^ denotes the total driver mutations in drivers and *n_i_*^neutral^ denotes the number of driver mutations in the category and *t*^neutral^ denotes the total neutral.

## Statistical overrepresentation in mutation classes

We calculated the statistical overrepresentation in various classes of mutations divided by wild-type (WT) residue, mutated residue (Mut), area and secondary structure.

To assess the overrepresentation in drivers compared to neutral mutations we performed a two-sided Fisher’s exact test on the contingency tables of counts of mutations in each category.

Our overrepresentation score *s_o_* was

*s*_0_ = sign(Δ_driver,neutral_)(-log(*p*_driver,neutral_))

Where Δ_driver,neutral_ is the difference in normalised counts (normalised by total in the whole sample (1000 Genomes or drivers) and *p*_driver,neutral_ denotes the p-value for the category from the Fisher’s exact test (two tailed) of the contingency table. P-values were corrected for multiple testing using false discovery rate [5,6].

## Functional impact scores

To obtain functional impact (FI) scores, we used the web interface to the Mutationassessor server from [7] for all the nsSNP mutations in both samples (COSMIC and 1000 Genomes). The FI score is based on alignments where conservation of each residue in each protein is scored. To assess the difference between 1k and COSMIC with respect to FI scores, we compared the FI distributions for the same categories as above (splitting the data by area, and amino acid substitution and splitting by area, secondary structure and amino acid substitution).

## PolyPhen predictions

We obtained PolyPhen predictions for all our mutations using the PolyPhen-2 sever from [8].

**CHASM predictions**

CHASM predictions were obtained from the CRAVAT webserver described in [9].

**Random forest model**

We used the randomForest package in R to construct the random forest model, based on the algorithm by [10].

**References**

1. Zvelebil MJ, Barton GJ, Taylor WR, Sternberg MJ (1987) Prediction of protein secondary structure and active sites using the alignment of homologous sequences. J Mol Biol 195: 957-961.

2. Henikoff S, Henikoff JG (1992) Amino acid substitution matrices from protein blocks. Proc Natl Acad Sci U S A 89: 10915-10919.

3. Kosiol C, Goldman N (2005) Different versions of the Dayhoff rate matrix. Mol Biol Evol 22: 193-199.

4. Robin X, Turck N, Hainard A, Tiberti N, Lisacek F, et al. (2011) pROC: an open-source package for R and S+ to analyze and compare ROC curves. BMC Bioinformatics 12: 77.

5. Benjamini Y, and Hochberg, Y. (1995) Controlling the false discovery rate: a practical and powerful approach to multiple testing. Journal of the Royal Statistical Society Series B 57: 289–300.

6. Benjamini Y, and Yekutieli, D. (2001) The control of the false discovery rate in multiple testing under dependency. Annals of Statistics 29: 1165–1188.

7. Reva B, Antipin Y, Sander C (2011) Predicting the functional impact of protein mutations: application to cancer genomics. Nucleic Acids Res 39: e118.

8. Adzhubei IA, Schmidt S, Peshkin L, Ramensky VE, Gerasimova A, et al. (2010) A method and server for predicting damaging missense mutations. Nat Methods 7: 248-249.

9. Douville C, Carter H, Kim R, Niknafs N, Diekhans M, et al. (2013) CRAVAT: cancer-related analysis of variants toolkit. Bioinformatics 29: 647-648.

10. Breiman L (2001) Random forests. Machine Learning 45: 5-32.
